# Spatial phylogenetics of the native woody plant species in Hainan, China

**DOI:** 10.1002/ece3.7180

**Published:** 2021-02-01

**Authors:** Zhi‐Xin Zhu, AJ Harris, Mir Muhammad Nizamani, Andrew H. Thornhill, Rosa A. Scherson, Hua‐Feng Wang

**Affiliations:** ^1^ Hainan Key Laboratory for Sustainable Utilization of Tropical Bioresources College of Tropical Crops Hainan University Haikou China; ^2^ Key Laboratory of Plant Resources Conservation and Sustainable Utilization South China Botanical Garden Chinese Academy of Science Guangzhou China; ^3^ Department for Environment and Water State Herbarium of South Australia Botanic Gardens and State Herbarium Adelaide SA Australia; ^4^ Departamento de Silvicultura y Conservación de la Naturaleza Facultad de Ciencias Forestales y Conservación de la Naturaleza Universidad de Chile Santiago Chile

**Keywords:** community assembly, hotspots, Island, phylogenetic diversity

## Abstract

To better identify biodiversity hotspots for conservation on Hainan Island, a tropical island in southern China, we assessed spatial variation in phylogenetic diversity and species richness using 18,976 georeferenced specimen records and a newly reconstructed molecular phylogeny of 957 native woody plants. Within this framework, we delineated bioregions based on vegetation composition and mapped areas of neoendemism and paleoendemism to identify areas of priority for conservation. Our results reveal that the southwest of Hainan is the most important hot spot for endemism and plant diversity followed by the southeast area. The distribution of endemic species showed a scattered, rather than clustered, pattern on the island. Based on phylogenetic range‐weighted turnover metrics, we delineated three major vegetational zones in Hainan. These largely correspond to natural secondary growth and managed forests (e.g., rubber and timber forests) in central Hainan, old‐growth forests and natural secondary growth forest at the margins of Hainan, and nature reserves on the island (e.g., Jianfeng and Diaoluo National Nature Reserves). Our study helps to elucidate potential botanical conservation priorities for Hainan within an evolutionary, phylogenetic framework.

## Graphical abstract

Our results reveal that the southwest of Hainan is the most important hot spot for endemism and plant diversity followed by the southeast area. The distribution of endemic species showed a scattered, rather than clustered, pattern on the island. Based on phylogenetic range‐weighted turnover metrics, we delineated four major vegetational zones in Hainan. These largely correspond to natural secondary growth and managed forests (e.g., rubber and timber forests) in central Hainan, old‐growth forests, natural secondary growth forest at the margins of Hainan, and the nature reserves on the island (e.g., Jianfeng and Diaoluo National Nature Reserves).

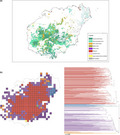

## 1 INTRODUCTION

Understanding patterns of biodiversity distribution is a primary goal of macroecological research and essential for conservation, particularly in the global context of increasing habitat loss (Thornton et al., 2020) and anthropogenic climate change (Marchioro et al., 2020). The fundamental unit of biodiversity assessment is the species. Traditionally, species richness (sometimes also evenness) comprises the primary metric to determine the diversity of a region or to compare the diversity among regions.

More recently, the biodiversity of a region is often considered within a phylogenetic framework, which, very generally, uses evolutionary distances as a measurement parameter (Mishler et al., 2014). Such methods are broadly regarded as spatial phylogenetics. In the way of using spatial phylogenetics, phylogenetic approaches add an evolutionary dimension to understanding spatial patterns of endemism and diversity (Mishler et al., 2014; Scherson et al., 2017; Thornhill et al., 2016).

Spatial phylogenetics can delineate unique bioregions and identify those with high alpha and beta phylogenetic diversity (Kling et al., 2018; Thornhill et al., 2016, 2017). While alpha diversity comprises a measure of local diversity within a sampling area, beta diversity facilitates scaling up to the full extent of a study region and represents changes in the composition of species across the landscape. Whittaker (1960) defined it as the total regional diversity minus the average diversity of sites in the area, but has since been measured in many other ways (Luna et al., 2020). As one key measure of biodiversity, beta diversity greatly helps us to understand the factors driving diversity and protecting ecosystems (Tan et al., 2019). Despite the importance of beta diversity assessments to conservation, there are few empirical studies for tropical Asia using this biodiversity proxy within the context of either traditional or spatial phylogenetic approaches (Ibanez, 2018; Li & Yue, 2020).

Hainan is a tropical Asian island located in southern China. The island is home to ca. 4,800–5,800 vascular plant species, of which 397 species are endemic (Francisco‐Ortega et al., 2010; Ren et al., 2018; Xing et al., 2012; Yang, 2013). Although the percentage of endemic plant species found in Hainan (10.5%) is lower than on some other tropical islands, such as Taiwan (19.3%), the Philippines (62.8%), and Madagascar (82.4%), Hainan possesses a high density of endemic species: 0.014 species/km^2^, comparable to Madagascar, which has 0.015 species/km^2^. Within China, Hainan harbors the nation's most extensive and well‐preserved tropical forest (Francisco‐Ortega et al., 2010). However, the island is facing pressure for continued economic development, which may lead to loss of biodiversity. Thus, conducting a biodiversity assessment of Hainan using modern, phylogenetic methods is of critical importance.

In this study, we investigated the spatial phylogenetic patterns of diversity of woody plants on Hainan Island using a species‐level phylogeny and distributional data of 957 species as a framework. Our primary objective was to delineate biodiversity hotspots within Hainan based on phylogenetic information. Our methods facilitate the assessment of both alpha diversity and beta diversity using numbers of species, and also reveal the number of different, independent phylogenetic lineages present per area unit. This is the first spatial phylogenetic study of Hainan using this comprehensive approach. We expect that our findings can be used to enhance biodiversity conservation strategies for the island in order to reduce biodiversity loss due to increasing anthropogenic activities.

## 2 METHODS

**FIGURE 1:**
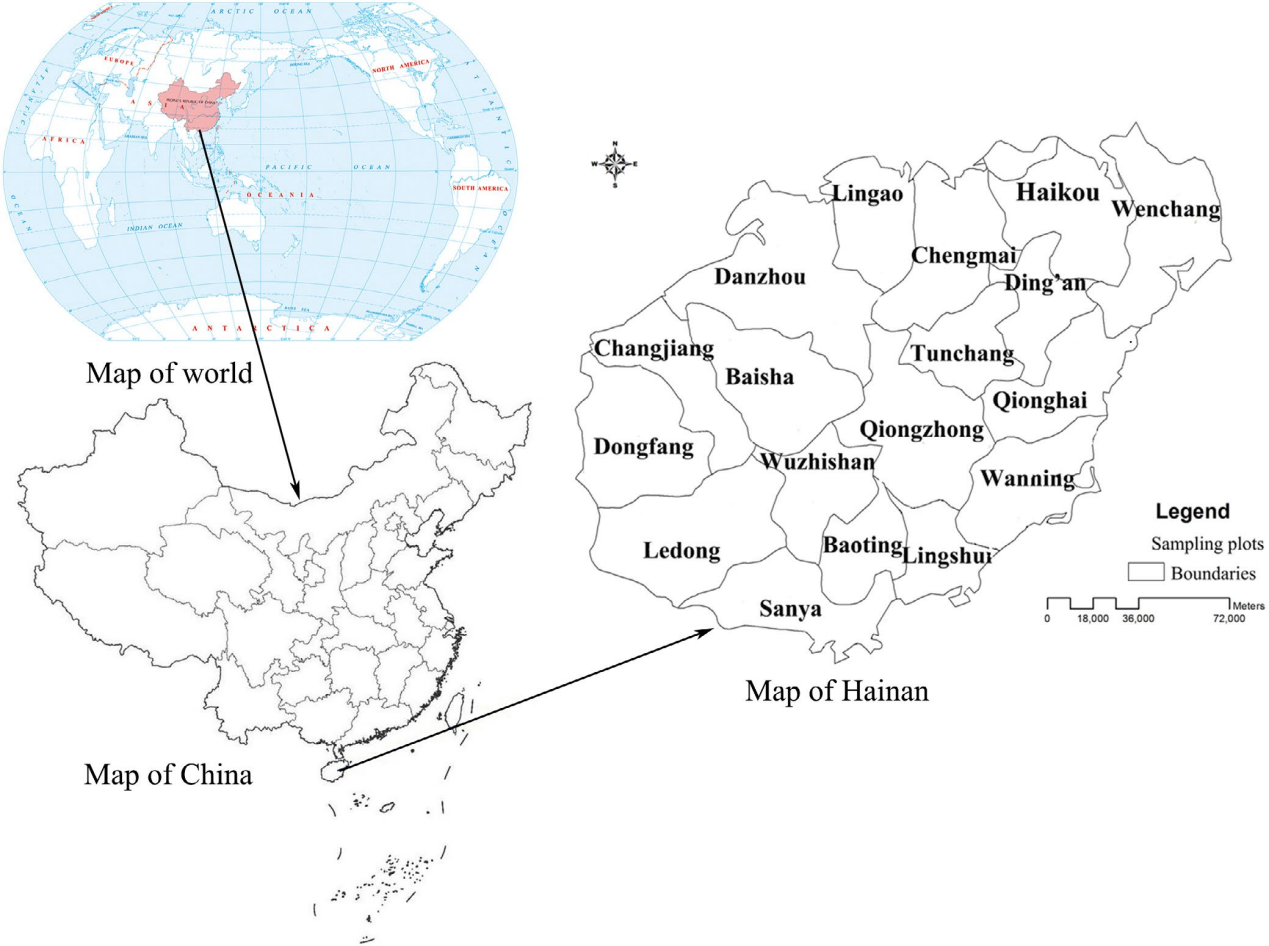
Geographic location of Hainan. The island is located in the monsoon climatic zone on the northern edge of tropical Asia 2019

The island of Hainan is located in the monsoon climate zone on the northern edge of tropical Asia (Figure 1). Therefore, the ecological characteristics of its vegetation are tropical but different from those in the equatorial zone, and its flora is characteristic of monsoon‐affected tropical vegetation. The natural tropical forest of Hainan can be divided into low‐lying rain forest, montane rain forest, and montane evergreen broad‐leaved forest. These natural vegetation types are mainly distributed in the present time in southwest Hainan, while there is extensive tropical silviculture (e.g., *Hevea brasiliensis* (Willd. ex A. Juss.) Müll. Arg. and *Areca catechu* Linn.) in the northeast of Hainan Island (Jiang, 1988; Zhu, 2016).

### 2.1 Sampling design

To determine the taxonomic composition of Hainan, we followed the checklist of plant species of Xing et al., (2012) and Yang (2013). Based on this, we initially found 4,234 plant species native to the main island of Hainan (not including subspecies, varieties, or forms). However, we determined that it was challenging to obtain GPS data for herbaceous species mainly because they show patchy (and sometimes seasonal) distributional patterns, so we removed herbs from the plant list and retained only the native woody plant species. In this study, woody plant species were defined as nonherbaceous spermatophytes with secondary xylem, including palms and lianas. In total, we identified 957 native woody plant species belonging to 118 families and 477 genera (Appendix S1), representing ca. 35.87% of the total plant species of Hainan and 25.44% total plant genera on the island (Yang, 2013).

### 2.2 Phylogenetic reconstruction

We used five loci to reconstruct the phylogeny: the nuclear internal transcribed spacer (ITS) and the chloroplast loci *trn*L, *mat*K, *ndh*F, and *rbc*L. We extracted gene sequences from GenBank (Benson et al., 2017) following the methodology of Smith et al., (2011). For the 477 genera of woody species, we checked available, published phylogenies (e.g., Li et al., 2019) in order to verify the monophyly of genera to the extent possible. When a genus was not monophyletic, we endeavored to select a sequence belonging to its main clade. We aligned the sequences with Muscle using default settings (Edgar, 2004). We conducted a maximum‐likelihood phylogenetic analysis (ML) in RAxML (Stamatakis, 2006) HPC2 within the CIPRES Portal (Miller et al., 2010) on XSEDE, we performed 1,000 bootstrap replicates with automatic termination, and we used the frequency‐based criterion to assess whether 1,000 bootstrap was enough for each node (i.e., ‐I autoFC). Following the analyses, we compared the resulting topology with that of Li et al. (2019) to verify congruence at the family level (i.e., classification of families into orders) and used FigTree 1.4.4 (Rambaut, 2018) for visualization and file format conversion.

Based on a preliminary phylogenetic reconstruction using the methods described above, we removed species that did not cluster with their closest relatives in the topology based on their taxonomic identities or recent literature (Li et al., 2019), because we assumed that such species may not have a clearly resolved taxonomy and could confound our analyses based on taxonomic identities (Figures S1 and S2). We performed a final phylogenetic analysis with the problematic species removed from our alignment.

### 2.3 Species occurrence data

In order to include as many localities as possible, we combined lists of specimens with geolocations from Xing et al., (2012) and Yang (2016) with occurrence records obtained from GBIF. We removed all occurrence records with “0” or “NA” values for latitude and longitude, as well as those that had coordinates outside the main island of Hainan (using a spatial overlay function in the raster package for R 3.6.1; Thornhill et al., 2016).

### 2.4 Biodiversity analyses

To conduct analyses of biodiversity, we created a 10 × 10 km grid fully covering the main island of Hainan, excluding small adjacent islands. The grid comprised 322 grid squares, and based on these, we used Biodiverse v3.1 (Laffan et al., 2010) to calculate taxonomic richness (TR), weighted endemism (WE), phylogenetic diversity (PD), phylogenetic endemism (PE), relative phylogenetic diversity (RPD), and relative phylogenetic endemism (RPE). RPD refers to PD on the original tree/PD on a comparison tree with all branch lengths equal, while RPE comprises PD on the original tree/PD on a comparison tree with all branch lengths equal (Mishler et al., 2014).

For Phylogenetic Diversity (PD), Relative Phylogenetic Diversity (RPD) and Relative Phylogenetic Endemism (RPE), significance was assessed by reshuffling of the identities of the taxa found in each grid cell from an island‐wide taxonomic pool to obtain null distributions, holding constant the total number of cells per taxon and the total taxa per cell. The null model assumes that the occurrences of a taxon display no spatial autocorrelation. We performed the random reshufflings 999 times using the Biodiverse pipeline for R ("rand_structured"; https://github.com/NunzioKnerr/biodiverse_pipeline).

We also conducted a range‐weighted phylo‐Sorenson analysis in Biodiverse using all 322 cells. This analysis identifies areas of lineage similarity based on a range‐weighted tree (Mishler et al., 2014; Thornhill et al., 2016, 2017). This approach has shown relatively high accuracy (compared to similar methods) in identifying unique biotic zones (Laffan et al., 2016). To perform the phylo‐Sorenson analysis, we used Biodiverse v3.1 (Laffan et al., 2010) to automatically map observed patterns of taxon richness (TR), phylogenetic diversity (PD), weighted endemism of taxa (WE), and phylogenetic endemism (PE) of plant species within the 322 10 × 10 km grid cells. In order to test the randomization test for PE and RP, we performed categorical analysis of neo‐ and paleoendemism (CANAPE) to identify significant concentrations of neoendemism (i.e., range‐restricted short branches) or paleoendemism (i.e., range‐restricted long branches), as well as mixtures of the two as Mishler et al., (2014).

## 3 RESULTS

### 3.1 Metrics of biodiversity

**FIGURE 2:**
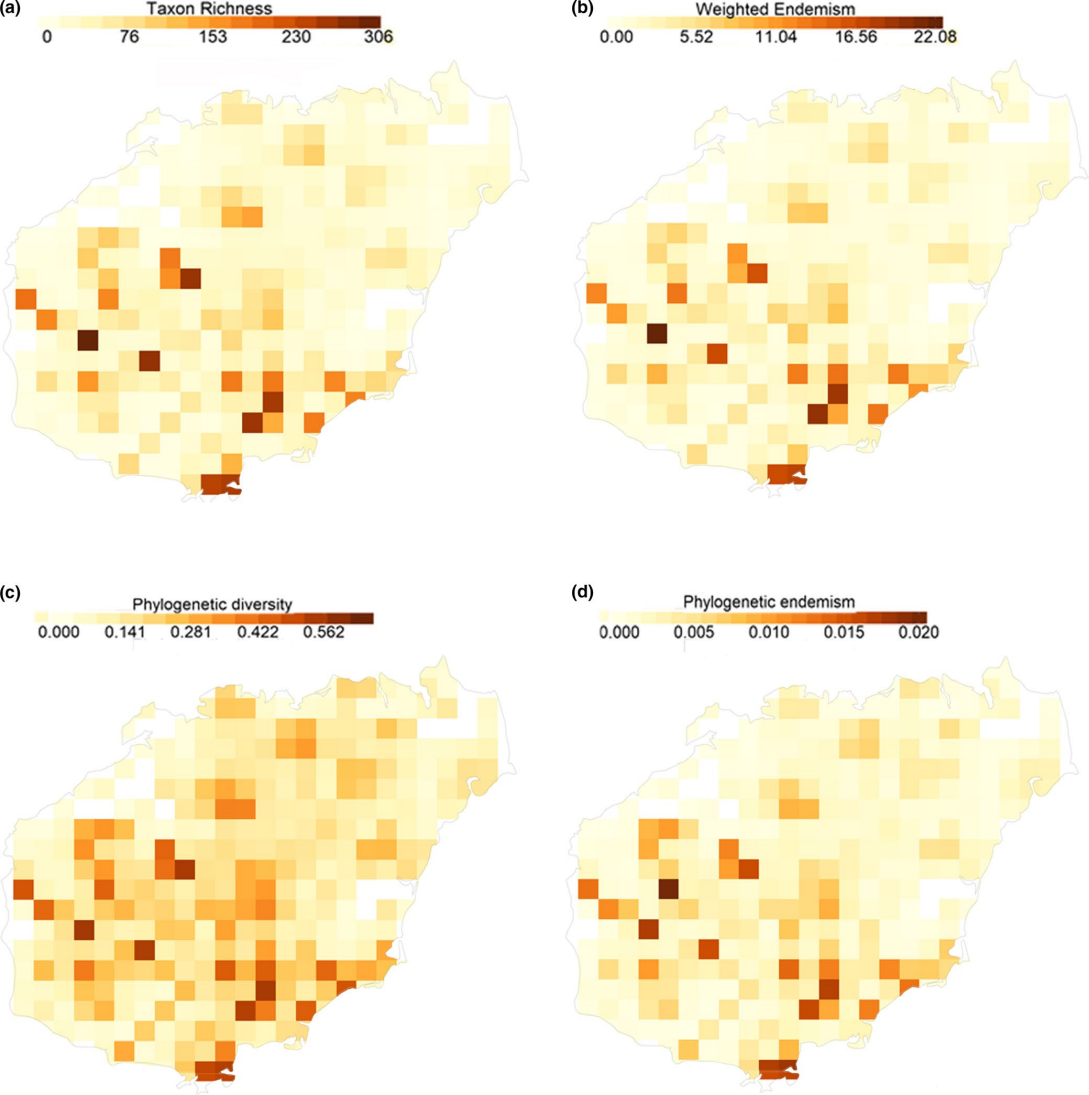
Maps showing the observed diversity and endemism of native woody species of Hainan. (a) genus richness (TR); (b) weighted endemism of taxa (WE); (c) phylogenetic diversity (PD); and (d) phylogenetic endemism (PE)

**FIGURE 3:**
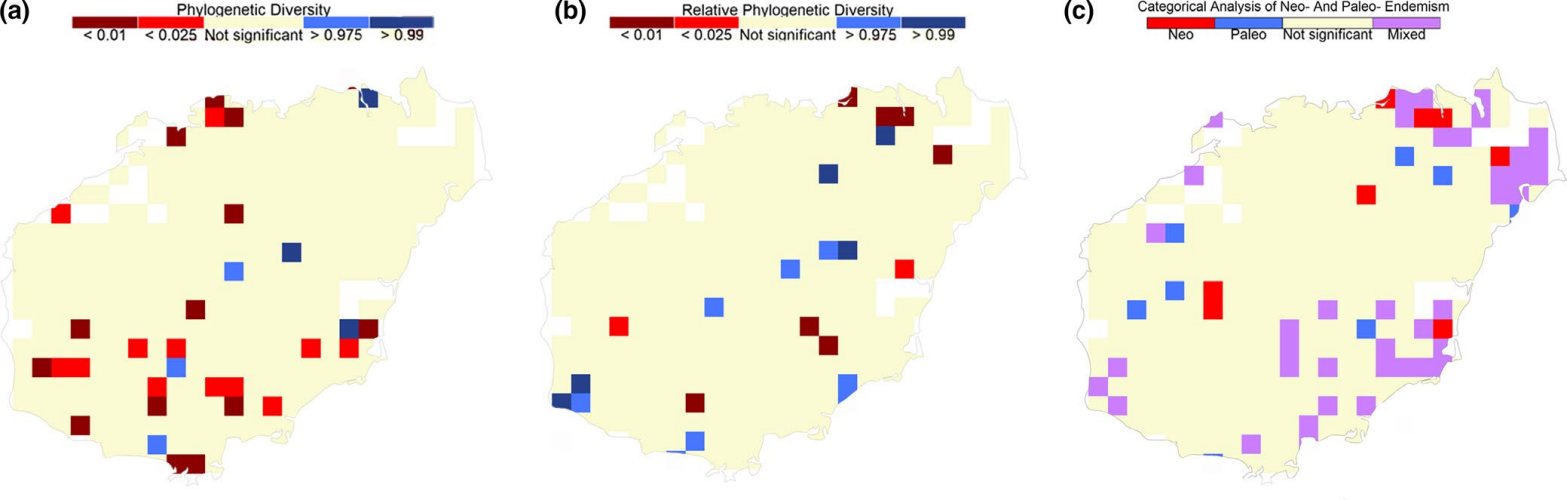
Maps showing significant results for distributional patterns of woody plants of Hainan based on permutation tests. (a) Phylogenetic diversity (PD): Red cells contain significantly less PD than expected; blue cells contain significantly more PD than expected. (b) Relative phylogenetic diversity (RPD): Red cells contain significantly less RPD than expected, meaning that the branch lengths for subtrees present in that area are shorter than expected. Blue cells contain significantly more RPD than expected, meaning that the branch lengths for subtrees present in that area are longer than expected. White cells contain no records, and results for beige cells are significant

Our results showed that the southwestern and southeastern parts of Hainan had the greatest plant diversity and uniqueness based on traditional taxonomic and phylogenetic metrics, that is, species (or taxon) richness (TR), weighted endermism (WE), phylogenetic diversity (PD), and phylogenetic endemism (PE) (Figure 2). Overall, TR (Figure 2a), PD (Figure 2c), WE (Figure 2b), and PE (Figure 2d; 3) showed similar patterns and were higher in the southwest and southeast and lower in the north of Hainan. Additionally, based on traditional taxonomic measures, the highest rates of endemism are found in cells concentrated within the southeast and southwest of the island (Figure 2, Appendix S3).

**FIGURE 4:**
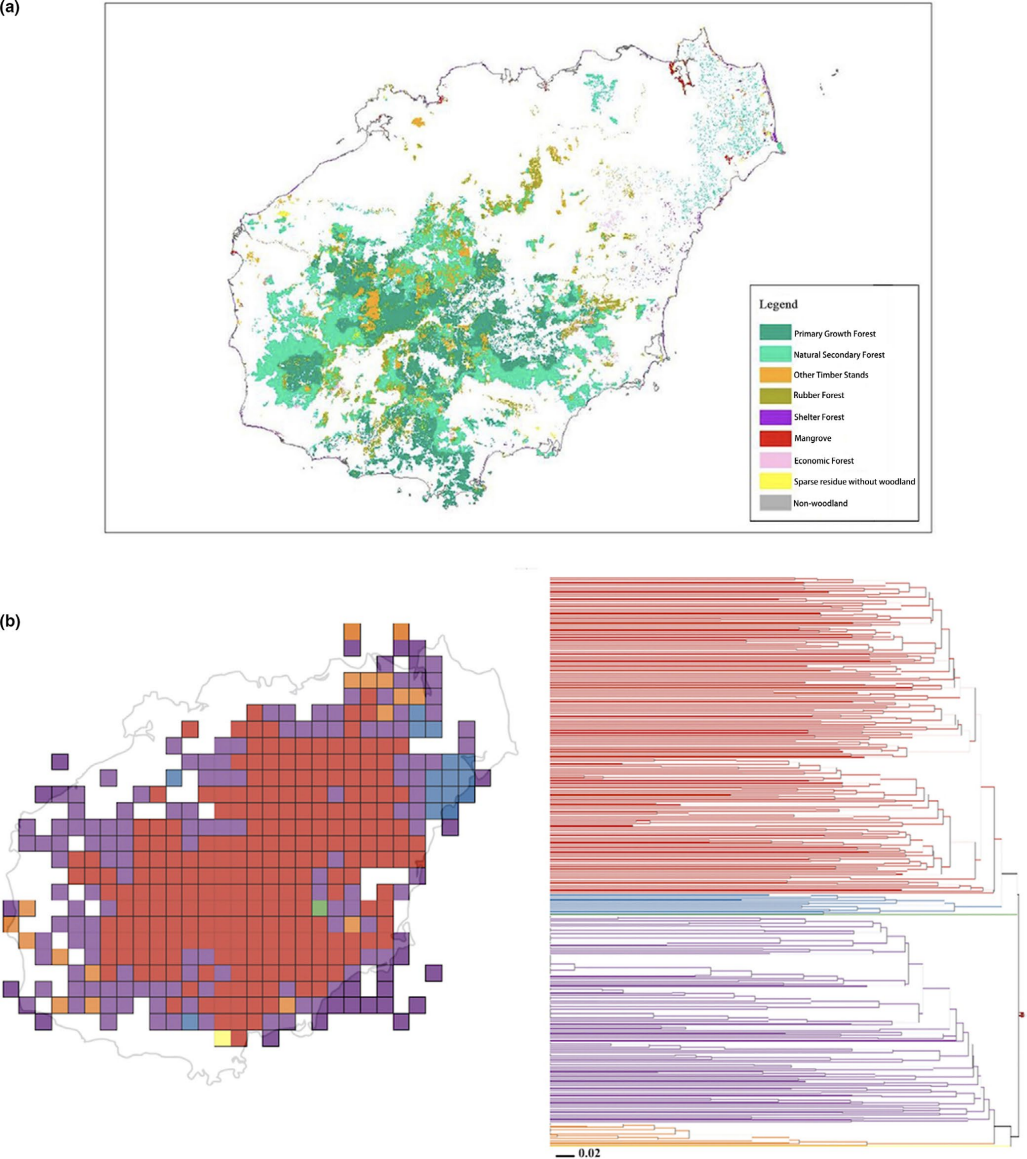
(a) The main vegetation types within Hainan island. (b) Map of centers of phylogenetic endemism. The colors of cells represent the different vegetation types as obtained with the range‐weighted turnover analysis. The color of the areas are the same as the colors in the cluster tree to the right

**FIGURE 5:**
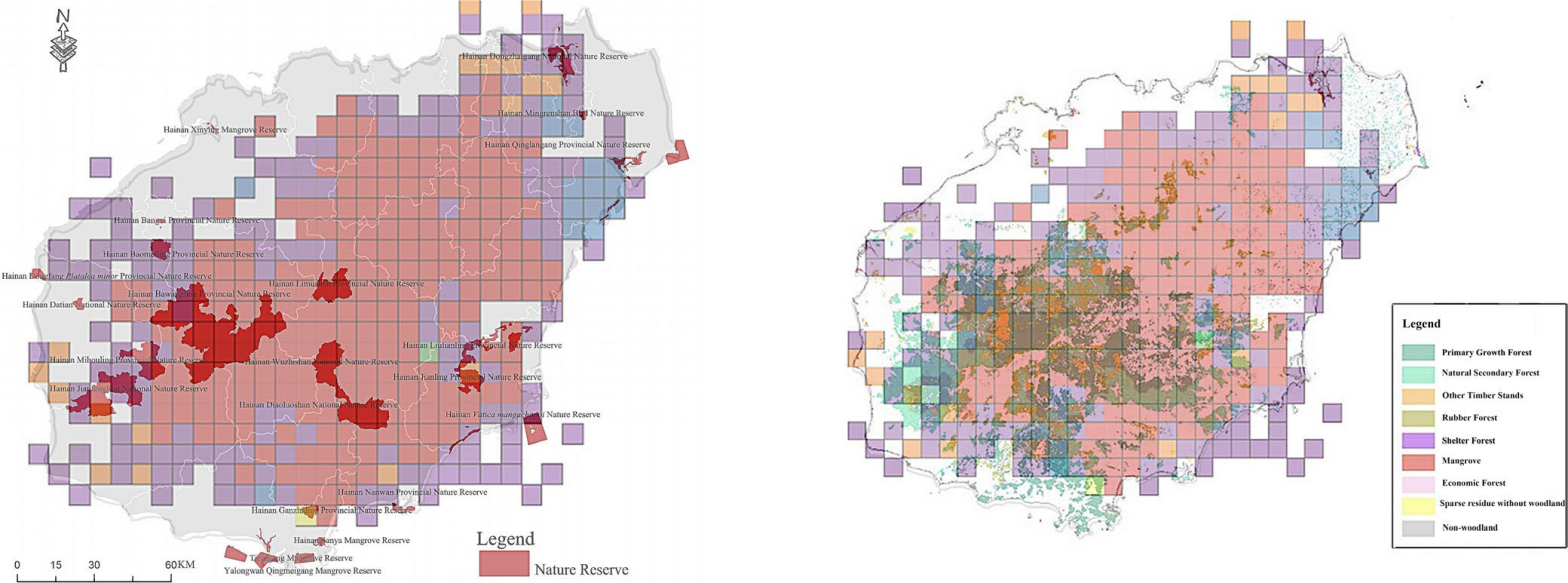
Overlayed maps of the range‐weighted turnover analysis of native woody plants onto the national/provincial reserves in Hainan, China

Of the 322 grid cells representing Hainan, 30 harbored significant PD (red, blue or purple cells in Figure 3a) and 21 have significant RPD (red, blue, or purple cells in Figure 3b). More cells show significant PD in the south of Hainan (<0.025, Figure 3a), while less cells in southern Hainan show significant RPD (<0.025, Figure 3b). There were 54 cells with significant PE based on the categorical analysis of neo‐ and paleoendemism (CANAPE) analysis (Figure 3c). Among these, areas of mixed endemism are most common, that is, showing a combination of significant neo‐ and paleoendemism (Figure 3c). Areas of paleoendemism and neoendemism were scattered and less common (Figure 3c). There are few significant paleo‐ or neoendemic areas in the north and south of the island. However, we also observed high endemism in Hainan national or provincial nature reserves, particularly in the southwest and southeast of the island (Figures 4 and 5).

### 3.2 Biotic transitions with phylogenetic turnover as a measurement

Based on range‐weighted turnover analysis, three major clusters of grid cells were detected, representing three vegetational zones: (a) natural secondary forests and regions of intense silviculture activities, such as rubber and timber forests, in central Hainan (Figure 4a); (b) old‐growth forests of northeastern Hainan (Figure 4b); and (c) natural secondary growth forests at the margins of Hainan, including the nature reserves in the southwest of the island (e.g., Limushan, Jianfengling, and Diaoluoshan National Nature Reserves; see the orange cluster, Figures 4b and 5).

## 4 DISCUSSION

### 4.1 Phylogenetic diversity and unique patterns associated with Hainan biomes

We obtained 18,976 georeferenced records and an alignment of 32,227 nucleotides (37.85% missing data) representing 957 native woody plant species of Hainan to assess the taxonomic and phylogenetic biodiversity of the island. These data represent 25.44% woody plant genera and 35.87% woody plant species on the island (Yang, 2013).

From a global perspective, Hainan is a continental island that belongs to the Paleotropical region (Jiang, 1988) and, overall, possesses a tropical evergreen broad‐leaved forest vegetation (Takhtajian, 1980; Ye et al., 2020). In general, its vegetation is more similar to that in Vietnam and Guangxi Province of China, than to Guangdong Province of China, which is geographically closer (Ye et al., 2020; Zhu, 2016). The island has likely had connections to the mainland in the past, allowing for an overland migration corridor, and at other times, including in the present, it has been isolated by shallow seas, causing it to be subject to basic principles of island biogeography (MacArthur & Wilson, 1963; Wilson & MacArthur, 1967) in terms of species colonization. Thus, prior connections to the mainland followed by vicariance and long‐distance dispersals from the mainland or other regional areas are both likely to have occurred, affecting the vegetational assembly of Hainan (Ye et al., 2020; Zhu, 2016). The mixed origins of floristic elements probably caused the unique spatial patterns found in Hainan, and this is reflected in our analyses, which revealed that most areas with significant endemism are mixed in terms of paleo‐ and neoendemism. Mixed endemism on islands of all kinds may be more common than previously thought, while earlier conventional wisdom suggested that oceanic islands should harbor more neoendemics, while continental islands should be sites of greater paleoendemism (Veron et al., 2019).

Although most significant centers of endemism in Hainan are mixed, there are also critical areas dominated by neoendemics. Neoendemism often represents an important source of new plant lineages that have evolved since their arrival from the regional species pool (Huang et al., 2016; Scherson et al., 2017; Spalink et al., 2018; Thornhill et al., 2016) and may be rarer than once thought on islands (Veron et al., 2019). Neoendemics may comprise valuable, unique genetic resources to help facilitate our response to global change. On Hainan, some examples of conservation targets could be species such as *Firmiana pulcherrima* H. H. Hsue (Malvaceae) (Wang et al., 2018) and *Symplocos ovatilobata* Noot. (Symplocaceae) (Zhu et al., 2018), which are listed as critically endangered (CR) and endangered (EN), respectively, in China and are distributed in the Diaoluoshan Mountain Nature Reserve of southwest Hainan.

### 4.2 Phylogenetic turnover in relation to biomes of Hainan

Hainan represents a unique assemblage of vegetation from diverse, regional species pools influenced by long‐term Earth history and evolutionary and historical biogeographic processes. Its flora belongs to the Indian–Malaysian flora based on its ecology, climate, geography, and vegetation (Slik et al., 2018; Jiang, 1988; Zhu, 2016). Overall, the plant community of Hainan island is far less diverse than the southeastern Asian mainland and the Malayan Islands, but it still possesses characteristic tropical southeastern Asian plant communities (Jiang, 1988). In particular, the monsoon tropical forests of Hainan are typical of this vegetation type, which is found throughout the Indian–Malaysian biogeographic realm. The monsoon climate has largely arisen within the Quaternary and comprises rainy and dry seasons (Jiang, 1988; Yang, 2016; Slik et al., 2018; Zhu, 2016). Similarly, the mountain valleys of Hainan possess elements from the Paleotropics and tropical Asia, reflecting aspects of Cenozoic (i.e., pre‐Quaternary) plant diversification across southeastern Asia. The flora of these valleys is broadly akin to that found within the Indochina Peninsula, which is also a part of the Indian–Malaysian biogeographic realm (Slik et al., 2018; Jiang, 1988).

The fact that the regions of high PD and PE that we uncovered coincided with areas recognized as biodiversity hotspots on the basis of other criteria suggests that large‐scale analyses such as this, despite issues of data availability or sampling bias, may be valuable for detecting hotspots that merit protection (Mishler et al., 2020). In Hainan, we found that TR, WE, and PE are higher in the southwest than in northeastern areas. This is not surprising because the northern part of the island is more densely populated (Yang, 2016; Zhu, 2016) and is the location of the capital, Haikou, while several large national nature reserves are located in the southwestern part of the island (i.e., Bawangling, Diaoluoshan national forest parks). This suggests that relatively recent anthropogenic disturbance and extirpation may have reduced biodiversity in the north of Hainan and also highlights that China's extensive national and provincial park system is critical for conserving biodiversity.

Numerous studies have been conducted on the relationships between species richness and environmental variables (Fitzpatrick et al., 2013; König et al., 2017). However, how contemporary and historical processes are intertwined to control the observed patterns remains uncertain (Ibanez, 2018; Jiménez‐Alfaro et al., 2018). In the tropics, water‐related aspects of the environment are widely known to be important in governing species distributions (Hawkins et al., 2003; Kreft & Jetz, 2007), and this appears to be true in Hainan, where temperature plays a secondary role in the organization of floristic bioregions (He et al., 2020). Notably, we found that the humid parts of Hainan have higher biodiversity than less humid areas, supporting the importance of water in the organization of biodiversity on the island.

Human activities are also known to play important roles in shaping patterns of species richness (Kareiva & Wennergren, 1995; Lenzen et al., 2009). There is a 6,000‐year history of human activities in Hainan involving the use of plant resources for wood, medicine, dyes, and other raw and refined products, and this has resulted in loss of many local endemic species and many hectares of primary tropical rain forest (Yang, 2016; Zhu, 2016). Moreover, recent anthropogenic activities have had profound effects on plant diversity in Hainan. For example, presently, the habitats of plants with extremely small population sizes are increasingly fragmented by development and agriculture (Chen et al., 2014). Nevertheless, the area surrounding the southern city of Sanya has a long history of human occupation but retains high levels of diversity and endemism (Figures 2 and 3). This suggests that the relationship of the people of Hainan to plant communities presently and historically is complex and merits further study.

In Hainan, tropical rainforest national parks (http://www.hntrnp.com/) were established since 2019 and create stable habitats and broad ecosystem integrity for areas of conservation priority on the island. These parks are especially crucial for range‐restricted taxa, which often require long‐term, stable habitats to persist. Specifically, range‐restricted taxa follow a more densely aggregated biogeographic pattern that is more difficult to explain based solely on environmental factors, although topography and habitat diversity probably play some role (De Groot et al., 2002; Kandziora et al., 2013; Odum, 1962).

### 4.3 Potential limitations and conservation implications

The paucity of georeferenced records for plants is a well‐known problem (Peterson et al., 2018; Scherson et al., 2018; Thornhill et al., 2016). For example, there are many specimens stored in Chinese herbaria without geographic coordinates for the collections, and some possess only coarse locality information (e.g., at the county level or above). Such specimens are often not suitable for spatially dependent analyses, such as in spatial phylogenetics. Thus, spatial phylogenetics can be limited by the quality of the occurrence data represented by the world's vast biodiversity collections. This highlights the need to continue to make extensive collections in the field and to ensure precise records of collection localities.

Spatial phylogenetic analyses can also be constrained by geographic and/or taxonomic collection bias. Issues of collection bias have sometimes been addressed using species distribution models (Fuentes‐Castillo et al., 2019). These models yield estimated occurrences, to which PD can be applied (Fuentes‐Castillo et al., 2019; Thornhill et al., 2017). However, CANAPE analyses have not been tested using estimated occurrences. Moreover, the distributions of narrowly endemic species can be challenging to estimate using species distribution models because there are inherently few occurrences available for building the models to begin with (Papeş & Gaubert, 2007), and their distributions may be controlled by factors other than the variables used for modeling. These factors may include specific requirements of the species in terms of soil, or microtopography, presence of symbionts, and others. Thus, performing CANAPE analyses from species distribution models may yield inaccurate results (Patsiou et al., 2014).

Geographic sampling biases yield heterogeneity of sampling density across a region of study (Hernández‐León et al., 2013; Scherson et al., 2017). In Hainan, there are several national forest parks, such as Diaoluoshan, Bawangling, and Jiafengling, that occupy a number of grid cells in the southwest of the island. Within the parks, there have been extensive government‐funded efforts to map plant diversity compared with surrounding areas. Thus, this yielded denser, perhaps more complete, sampling for these areas, and this could have increased our estimations of TR and PD (see Figure 2). However, our analyses using randomizations are robust to the heterogeneity of sampling so that the patterns of significance that we detected in the poorly sampled grid cells should have equal confidence as those for the well‐sampled grid cells surrounding them (Appendix S2).

The woody flora of Hainan has been extensively studied (Xing et al., 2012; Yang, 2013; 2016) and collected, and this has enabled us to conduct a biodiversity assessment using traditional or spatial phylogenetic methods at a fine, species‐level taxonomic scale. Consequently, we have been able to detect spatial patterns that would likely not be evident when working at coarser scales. As more collections‐based and field‐based data become available for Hainan, we expect to expand our study to include herbaceous species, in order to have a more complete perspective of the spatial patterns of phylodiversity in this complex continental island system.

## 5 CONCLUSIONS

Species richness remains generally accepted as a basic metric for conservation priority actions, but increasingly evolutionary factors underlying the assembly of vegetation are also regarded as important for assessing the level of need for and value of protecting an area. Spatial phylogenetic methods allow us to develop approaches for conserving the diversity of rare lineages from an evolutionary standpoint. Our results showed that the majority of the conservation gaps occur in the central or southern montane areas of Hainan, whereas there are already extensive nature reserves under protection in the southeast and southwest. These conservation gaps include extensive areas harboring plants of Hainan that are more xeromorphic and adapted to the dry season of the monsoon climate. We argue that it is vital to provide more protection to these areas with conservation gaps, especially by reducing human disturbance.

## CONFLICT OF INTEREST

None declared.

## AUTHOR CONTRIBUTIONS

**Zhi‐Xin Zhu:** Investigation (supporting); writing–original draft (supporting); writing–review and editing (equal). **AJ Harris:** Conceptualization (equal); data curation (supporting); formal analysis (supporting); investigation (supporting); methodology (equal); resources (equal); software (supporting); supervision (equal); validation (supporting); visualization (supporting); writing–original draft (supporting); writing–review and editing (equal). **Mir Muhammad Nizamani:** Writing–original draft (supporting); writing–review and editing (equal). **Andrew H. Thornhill:** Methodology (equal); resources (equal); software (supporting); supervision (equal); validation (supporting); visualization (supporting); writing–original draft (supporting); writing–review and editing (equal). **Rosa A. Schersona:** Methodology (equal); software (supporting); validation (supporting); visualization (supporting); writing–original draft (supporting); writing–review and editing (equal). **Hua‐Feng Wang:** Conceptualization (equal); data curation (lead); formal analysis (lead); funding acquisition (supporting); investigation (equal); methodology (equal); project administration (supporting); resources (equal); software (lead); supervision (equal); validation (lead); visualization (lead); writing–original draft (lead); writing–review and editing (equal).

## Supporting information

Fig S1

Fig S2

Appendix S1

Appendix S2

Appendix S3

## Data Availability

All of the data used in this paper are included as Appendix.
