# Supplementary material for: Spatial phylogenetics of the native woody plant species in Hainan, China
Source: Ecol Evol. 2021 Feb 1;11(5):2100–9. doi: 10.1002/ece3.7180 (PMC7920777; doi:10.1002/ece3.7180)
Supplement: Supplementary file 4 — Appendix S2 [file ECE3-11-2100-s004.docx]

| Latin_name | Longitude | Latitude | x_epsg_341 | y_epsg_341 |
| --- | --- | --- | --- | --- |
| Abroma augusta | 109.5104 | 19.51803 | 29281.71 | 342750.8 |
| Abroma augusta | 109.7747 | 18.5641 |  |  |
| Abroma augusta | 109.8139 | 18.58248 |  |  |
| Abroma augusta | 109.8716 | 18.60381 |  |  |
| Abroma augusta | 109.6559 | 18.25764 |  |  |
| Abroma augusta | 109.5944 | 18.26027 |  |  |
| Abroma augusta | 109.5975 | 18.22565 |  |  |
| Abroma augusta | 108.7438 | 19.05099 |  |  |
| Abroma augusta | 108.7472 | 19.06683 |  |  |
| Abroma augusta | 108.7999 | 18.97076 |  |  |
| Abroma augusta | 109.471 | 19.21812 |  |  |
| Abroma augusta | 109.392 | 19.23083 |  |  |
| Abroma augusta | 109.3459 | 19.21473 |  |  |
| Abroma augusta | 109.6755 | 19.95354 |  |  |
| Abroma augusta | 109.7417 | 19.89798 |  |  |
| Abroma augusta | 109.5963 | 19.88359 |  |  |
| Abroma augusta | 109.0387 | 18.69876 |  |  |
| Abroma augusta | 109.2936 | 18.77968 |  |  |
| Abroma augusta | 109.1333 | 18.62271 |  |  |
| Abroma augusta | 109.74 | 19.88827 |  |  |
| Abroma augusta | 109.6113 | 19.90022 |  |  |
| Abrus precatorius | 108.9515 | 18.90932 | -31419.3 | 277210.7 |
| Abrus precatorius | 109.1191 | 19.05985 | -13271 | 293304.6 |
| Abrus precatorius | 109.1215 | 19.14164 | -12747.8 | 302339.7 |
| Abrus precatorius | 110.2942 | 20.01217 | 112706.7 | 395269.1 |
| Abrus precatorius | 110.5245 | 19.23187 | 134898.9 | 308461.6 |
| Abrus precatorius | 109.5 | 18.2 |  |  |
| Abrus precatorius | 108.7428 | 19.04489 |  |  |
| Abrus precatorius | 108.716 | 19.16136 |  |  |
| Abrus precatorius | 109.1527 | 18.73549 |  |  |
| Abrus precatorius | 109.5273 | 18.23845 |  |  |
| Abrus precatorius | 109.5767 | 18.17714 |  |  |
| Abrus precatorius | 109.5097 | 18.34179 |  |  |
| Abrus precatorius | 109.1331 | 18.75093 |  |  |
| Abrus precatorius | 108.7619 | 19.09883 |  |  |
| Abrus precatorius | 110.4409 | 18.79725 |  |  |
| Abrus pulchellus | 109.0333 | 18.86036 | -22982.8 | 271527.7 |
| Abrus pulchellus | 109.4044 | 18.88006 | 16149.68 | 272536.9 |
| Abrus pulchellus | 109.414 | 19.57115 | 19347.16 | 348910 |
| Abrus pulchellus | 110.2728 | 19.59653 | 109405.7 | 349374.1 |
| Abrus pulchellus | 109.6807 | 18.37829 | 43729.57 | 216234.2 |
| Abrus pulchellus | 110.3 | 20 |  |  |
| Abrus pulchellus | 109.4 | 18.3 |  |  |
| Abrus pulchellus | 109.2 | 18.7 |  |  |
| Abrus pulchellus | 108.7099 | 19.08943 |  |  |
| Abrus pulchellus | 108.7604 | 19.15106 |  |  |
| Abrus pulchellus | 109.2853 | 18.73407 |  |  |
| Abrus pulchellus | 109.2189 | 18.76064 |  |  |
| Abrus pulchellus | 109.6667 | 18.29648 |  |  |
| Abrus pulchellus | 109.4116 | 18.33397 |  |  |
| Abrus pulchellus | 110.3616 | 18.73728 |  |  |
| Abrus pulchellus | 110.375 | 18.92282 |  |  |
| Abutilon indicum | 108.9122 | 18.99917 | -35232.9 | 287275.8 |
| Abutilon indicum | 109.0333 | 18.86036 | -22982.8 | 271527.7 |
| Abutilon indicum | 109.0367 | 19.17511 | -21544.3 | 306313.7 |
| Abutilon indicum | 109.1544 | 19.01211 | -9728.08 | 287914.8 |
| Abutilon indicum | 109.1741 | 18.37558 | -9781.56 | 217466.3 |
| Abutilon indicum | 109.4401 | 18.69801 | 19324.7 | 252298.9 |
| Abutilon indicum | 109.4865 | 19.15807 | 25651.28 | 303028.8 |
| Abutilon indicum | 109.7575 | 19.47974 | 55069.1 | 337810.7 |
| Abutilon indicum | 109.9578 | 18.76128 | 74063.58 | 257827 |
| Abutilon indicum | 110.2356 | 18.69662 | 103162.5 | 249961.2 |
| Abutilon indicum | 110.3153 | 19.16929 | 112768.7 | 302034.3 |
| Abutilon indicum | 109.7636 | 18.56004 |  |  |
| Abutilon indicum | 109.8299 | 18.57731 |  |  |
| Abutilon indicum | 109.8514 | 18.59658 |  |  |
| Abutilon indicum | 109.6494 | 18.24862 |  |  |
| Abutilon indicum | 109.5956 | 18.25962 |  |  |
| Abutilon indicum | 109.5913 | 18.22575 |  |  |
| Abutilon indicum | 108.7364 | 19.04886 |  |  |
| Abutilon indicum | 108.7431 | 19.06551 |  |  |
| Abutilon indicum | 108.7845 | 18.96176 |  |  |
| Abutilon indicum | 109.4708 | 19.21848 |  |  |
| Abutilon indicum | 109.3854 | 19.2347 |  |  |
| Abutilon indicum | 109.3496 | 19.21495 |  |  |
| Abutilon indicum | 109.6726 | 19.95372 |  |  |
| Abutilon indicum | 109.7673 | 19.87094 |  |  |
| Abutilon indicum | 109.6128 | 19.89049 |  |  |
| Abutilon indicum | 109.0733 | 18.7538 |  |  |
| Abutilon indicum | 109.2942 | 18.79036 |  |  |
| Abutilon indicum | 109.0958 | 18.58663 |  |  |
| Abutilon indicum | 109.7644 | 19.8949 |  |  |
| Abutilon indicum | 109.644 | 19.90119 |  |  |
| Acacia concinna | 109.1544 | 19.01211 | -9728.08 | 287914.8 |
| Acacia concinna | 109.1544 | 19.01212 | -9727.97 | 287914.9 |
| Acacia concinna | 109.1544 | 19.01212 | -9727.86 | 287915 |
| Acacia concinna | 109.1544 | 19.01212 | -9727.75 | 287915.1 |
| Acacia concinna | 109.1544 | 19.01212 | -9727.65 | 287915.2 |
| Acacia concinna | 109.0252 | 19.27446 |  |  |
| Acacia concinna | 109.1014 | 19.35996 |  |  |
| Acacia concinna | 110.0608 | 19.81061 |  |  |
| Acacia concinna | 110.2269 | 19.59606 |  |  |
| Acacia concinna | 109.5919 | 18.7588 |  |  |
| Acacia concinna | 110.4664 | 19.82394 |  |  |
| Acacia concinna | 110.7849 | 19.54901 |  |  |
| Acacia concinna | 110.7311 | 19.46723 |  |  |
| Acacia concinna | 110.4238 | 18.79326 |  |  |
| Acacia concinna | 110.377 | 18.77741 |  |  |
| Acacia concinna | 110.4097 | 18.75297 |  |  |
| Acacia concinna | 109.6895 | 18.82828 |  |  |
| Acacia concinna | 109.8822 | 18.78711 |  |  |
| Acacia concinna | 109.8668 | 19.09533 |  |  |
| Acacia concinna | 109.5509 | 19.02951 |  |  |
| Acacia concinna | 110.2149 | 20.01202 |  |  |
| Acacia concinna | 110.4622 | 19.84982 |  |  |
| Acacia concinna | 110.4148 | 19.8474 |  |  |
| Acacia concinna | 110.775 | 19.62593 |  |  |
| Acacia concinna | 110.7453 | 19.67258 |  |  |
| Acacia pennata | 108.9122 | 18.99917 | -35232.9 | 287275.8 |
| Acacia pennata | 108.9795 | 18.89322 | -28525.9 | 275338 |
| Acacia pennata | 109.1191 | 19.05985 | -13271 | 293304.6 |
| Acacia pennata | 109.2653 | 18.97408 | 1810.336 | 283359.5 |
| Acacia pennata | 109.7647 | 19.48805 | 55849.78 | 338708.9 |
| Acacia pennata | 109.5 | 18.3 |  |  |
| Acacia pennata | 110.5 | 19.3 |  |  |
| Acacia pennata | 109.2 | 18.7 |  |  |
| Acacia pennata | 109.7 | 18.6 |  |  |
| Acacia pennata | 110 | 18.5 |  |  |
| Acacia pennata | 110.034 | 19.74974 |  |  |
| Acacia pennata | 110.0523 | 19.73156 |  |  |
| Acacia pennata | 109.9554 | 19.73181 |  |  |
| Acacia pennata | 109.5857 | 19.47997 |  |  |
| Acacia pennata | 109.6455 | 19.45045 |  |  |
| Acacia pennata | 109.6404 | 19.5066 |  |  |
| Acacia pennata | 108.9886 | 19.28832 |  |  |
| Acacia pennata | 109.143 | 19.32265 |  |  |
| Acacia pennata | 109.1176 | 19.32176 |  |  |
| Acacia pennata | 109.4694 | 19.22027 |  |  |
| Acacia pennata | 109.3938 | 19.23611 |  |  |
| Acacia pennata | 109.3552 | 19.21172 |  |  |
| Acacia pennata | 108.7319 | 19.0427 |  |  |
| Acacia pennata | 108.7441 | 19.06558 |  |  |
| Acacia pennata | 108.8058 | 18.96034 |  |  |
| Acanthus ilicifolius | 110.0822 | 19.53483 | 89270.66 | 343031.3 |
| Acanthus ilicifolius | 110 | 18.5 |  |  |
| Acanthus ilicifolius | 109.5 | 18.3 |  |  |
| Acanthus ilicifolius | 109 | 19 |  |  |
| Acanthus ilicifolius | 110.8934 | 19.59098 |  |  |
| Acanthus ilicifolius | 110.5672 | 19.35404 |  |  |
| Acanthus ilicifolius | 110.3393 | 19.25554 |  |  |
| Acanthus ilicifolius | 110.3073 | 19.97897 |  |  |
| Acanthus ilicifolius | 110.8035 | 19.73141 |  |  |
| Acanthus ilicifolius | 110.7306 | 19.6092 |  |  |
| Acanthus ilicifolius | 110.9418 | 19.70946 |  |  |
| Acanthus ilicifolius | 110.8377 | 19.58961 |  |  |
| Acanthus ilicifolius | 110.8155 | 19.56624 |  |  |
| Acanthus ilicifolius | 110.2148 | 20.01157 |  |  |
| Acanthus ilicifolius | 110.457 | 19.8574 |  |  |
| Acanthus ilicifolius | 110.4108 | 19.85894 |  |  |
| Acanthus ilicifolius | 110.2159 | 20.01176 |  |  |
| Acanthus ilicifolius | 110.4672 | 19.85467 |  |  |
| Acanthus ilicifolius | 110.4141 | 19.85354 |  |  |
| Acanthus ilicifolius | 110.3759 | 19.33102 |  |  |
| Acanthus ilicifolius | 110.5667 | 19.24272 |  |  |
| Acer fabri | 108.7989 | 18.69619 | -48238.4 | 254162.2 |
| Acer fabri | 109.1191 | 19.05985 | -13271 | 293304.6 |
| Acer fabri | 109.3011 | 18.85155 | 5180.119 | 269701.6 |
| Acer fabri | 109.4865 | 19.15807 | 25651.28 | 303028.8 |
| Acer fabri | 109.8188 | 19.14345 | 60528.46 | 300462.2 |
| Acer fabri | 110.2542 | 18.78692 | 105355.2 | 259902.1 |
| Acer fabri | 109.7 | 18.6 |  |  |
| Acer fabri | 109.4373 | 19.25145 |  |  |
| Acer fabri | 109.3075 | 19.10845 |  |  |
| Acer fabri | 109.8143 | 19.04939 |  |  |
| Acer fabri | 109.7737 | 18.96435 |  |  |
| Acer fabri | 109.2938 | 18.77202 |  |  |
| Acer fabri | 109.0624 | 18.75642 |  |  |
| Acer fabri | 109.7786 | 18.58468 |  |  |
| Acer fabri | 109.6158 | 18.69854 |  |  |
| Acer fabri | 109.1393 | 18.88316 |  |  |
| Acer laurinum | 108.7989 | 18.69619 | -48238.4 | 254162.2 |
| Acer laurinum | 109.3011 | 18.85155 | 5180.119 | 269701.6 |
| Acer laurinum | 109.6579 | 18.76335 | 42477.13 | 258885.1 |
| Acer laurinum | 109.837 | 18.73456 | 61260.82 | 255199 |
| Acer laurinum | 110.2549 | 18.71143 | 105232.9 | 251551.4 |
| Acer laurinum | 109.7 | 18.6 |  |  |
| Acer laurinum | 109.5 | 18.3 |  |  |
| Acer laurinum | 109.6 | 19.8984 |  |  |
| Acer laurinum | 109.7744 | 19.76405 |  |  |
| Acer laurinum | 110.0189 | 19.70071 |  |  |
| Acer laurinum | 109.9159 | 19.78214 |  |  |
| Acer laurinum | 110.2633 | 18.89452 |  |  |
| Acer laurinum | 110.3567 | 18.70862 |  |  |
| Acer laurinum | 108.798 | 19.03609 |  |  |
| Acer laurinum | 109.2526 | 18.74276 |  |  |
| Acer laurinum | 109.9447 | 18.4587 |  |  |
| Acronychia pedunculata | 109.0627 | 18.65861 | -20574.2 | 249125 |
| Acronychia pedunculata | 109.6579 | 18.76335 | 42477.13 | 258885.1 |
| Acronychia pedunculata | 109.6828 | 18.3841 | 43968.29 | 216870.9 |
| Acronychia pedunculata | 110.1592 | 18.68652 | 95078.4 | 249035.8 |
| Acronychia pedunculata | 110.2559 | 19.09526 | 106327.7 | 293994.1 |
| Acronychia pedunculata | 110.5 | 19.3 |  |  |
| Acronychia pedunculata | 109.7 | 18.6 |  |  |
| Acronychia pedunculata | 110 | 18.5 |  |  |
| Acronychia pedunculata | 109.5 | 18.3 |  |  |
| Acronychia pedunculata | 110.0308 | 19.7678 |  |  |
| Acronychia pedunculata | 110.2061 | 19.52861 |  |  |
| Acronychia pedunculata | 109.4687 | 19.21829 |  |  |
| Acronychia pedunculata | 109.3931 | 19.23365 |  |  |
| Acronychia pedunculata | 109.3495 | 19.21821 |  |  |
| Acronychia pedunculata | 109.6678 | 19.95205 |  |  |
| Acronychia pedunculata | 109.7344 | 19.88301 |  |  |
| Acronychia pedunculata | 109.6427 | 19.8683 |  |  |
| Acronychia pedunculata | 109.0384 | 18.74884 |  |  |
| Acronychia pedunculata | 109.2871 | 18.79924 |  |  |
| Acronychia pedunculata | 109.0414 | 18.5928 |  |  |
| Acronychia pedunculata | 109.7781 | 18.56381 |  |  |
| Acronychia pedunculata | 109.8272 | 18.57402 |  |  |
| Acronychia pedunculata | 109.858 | 18.60339 |  |  |
| Acronychia pedunculata | 109.6514 | 18.25095 |  |  |
| Acronychia pedunculata | 109.5906 | 18.25709 |  |  |
| Acronychia pedunculata | 109.5989 | 18.22525 |  |  |
| Acronychia pedunculata | 108.7424 | 19.04952 |  |  |
| Acronychia pedunculata | 108.7472 | 19.06359 |  |  |
| Acronychia pedunculata | 108.7837 | 18.95251 |  |  |
| Actinodaphne pilosa | 108.7989 | 18.69619 | -48238.4 | 254162.2 |
| Actinodaphne pilosa | 109.1215 | 19.14164 | -12747.8 | 302339.7 |
| Actinodaphne pilosa | 109.3683 | 19.19189 | 13342.08 | 307122.7 |
| Actinodaphne pilosa | 109.416 | 18.58215 | 16423.77 | 239559.1 |
| Actinodaphne pilosa | 109.4865 | 19.15807 | 25651.28 | 303028.8 |
| Actinodaphne pilosa | 109.5425 | 19.03583 | 31162.38 | 289348.8 |
| Actinodaphne pilosa | 109.6579 | 18.76335 | 42477.13 | 258885.1 |
| Actinodaphne pilosa | 109.6807 | 18.37829 | 43729.57 | 216234.2 |
| Actinodaphne pilosa | 110.0275 | 19.81293 |  |  |
| Actinodaphne pilosa | 110.2426 | 19.58447 |  |  |
| Actinodaphne pilosa | 109.4679 | 19.21724 |  |  |
| Actinodaphne pilosa | 109.3953 | 19.2315 |  |  |
| Actinodaphne pilosa | 109.3457 | 19.21029 |  |  |
| Actinodaphne pilosa | 109.6706 | 19.95773 |  |  |
| Actinodaphne pilosa | 109.7429 | 19.90575 |  |  |
| Actinodaphne pilosa | 109.6039 | 19.87653 |  |  |
| Actinodaphne pilosa | 109.0144 | 18.73655 |  |  |
| Actinodaphne pilosa | 109.294 | 18.7795 |  |  |
| Actinodaphne pilosa | 109.0942 | 18.55948 |  |  |
| Actinodaphne pilosa | 109.7716 | 18.5596 |  |  |
| Actinodaphne pilosa | 109.8202 | 18.57589 |  |  |
| Actinodaphne pilosa | 109.8514 | 18.59776 |  |  |
| Actinodaphne pilosa | 109.6508 | 18.25122 |  |  |
| Actinodaphne pilosa | 109.5971 | 18.25561 |  |  |
| Actinodaphne pilosa | 109.5935 | 18.22574 |  |  |
| Actinodaphne pilosa | 108.7328 | 19.04942 |  |  |
| Actinodaphne pilosa | 108.7467 | 19.06628 |  |  |
| Actinodaphne pilosa | 108.7793 | 18.96931 |  |  |
| Adenanthera microsperma | 108.8228 | 18.4993 | -46431 | 232309.2 |
| Adenanthera microsperma | 108.982 | 18.6052 | -29265.1 | 243483.5 |
| Adenanthera microsperma | 109.0799 | 19.00987 | -17563.9 | 287906.3 |
| Adenanthera microsperma | 109.5259 | 19.13355 | 29723.31 | 300201.6 |
| Adenanthera microsperma | 109.6036 | 18.37017 | 35565.66 | 215558.3 |
| Adenanthera microsperma | 109.9556 | 19.26192 | 75237.18 | 313189.7 |
| Adenanthera microsperma | 110.1756 | 18.76435 | 97019.43 | 257602 |
| Adenanthera microsperma | 109.2 | 18.7 |  |  |
| Adenanthera microsperma | 109.5 | 18.3 |  |  |
| Adenanthera microsperma | 108.9944 | 19.20088 |  |  |
| Adenanthera microsperma | 109.3501 | 19.2307 |  |  |
| Adenanthera microsperma | 108.7225 | 18.96467 |  |  |
| Adenanthera microsperma | 109.1166 | 18.73203 |  |  |
| Adenanthera microsperma | 109.5176 | 18.28927 |  |  |
| Adenanthera microsperma | 109.6536 | 18.29188 |  |  |
| Adenanthera microsperma | 109.2704 | 18.72553 |  |  |
| Adina pilulifera | 109.1215 | 19.14164 | -12747.8 | 302339.7 |
| Adina pilulifera | 109.2014 | 19.0858 | -4531.56 | 295912 |
| Adina pilulifera | 109.453 | 19.21626 | 22314.51 | 309561.2 |
| Adina pilulifera | 109.4914 | 19.41113 | 26959.92 | 330989.8 |
| Adina pilulifera | 109.7575 | 19.47974 | 55069.1 | 337810.7 |
| Adina pilulifera | 109.5 | 18.3 |  |  |
| Adina pilulifera | 109.7 | 18.6 |  |  |
| Adina pilulifera | 109.2 | 18.7 |  |  |
| Adina pilulifera | 110.036 | 19.69586 |  |  |
| Adina pilulifera | 109.749 | 19.84254 |  |  |
| Adina pilulifera | 109.5156 | 19.5361 |  |  |
| Adina pilulifera | 109.4256 | 19.27057 |  |  |
| Adina pilulifera | 109.0569 | 19.28677 |  |  |
| Adina pilulifera | 108.6579 | 19.06821 |  |  |
| Adina pilulifera | 109.1221 | 18.74471 |  |  |
| Adina pilulifera | 109.6433 | 18.69431 |  |  |
| Adina pilulifera | 109.9921 | 18.55116 |  |  |
| Adina pilulifera | 110.3677 | 19.26538 |  |  |
| Adina pilulifera | 110.3725 | 18.82207 |  |  |
| Adina pilulifera | 109.1592 | 18.73886 |  |  |
| Adina pilulifera | 109.1976 | 18.77982 |  |  |
| Adina pilulifera | 109.4133 | 19.21028 |  |  |
| Adina pilulifera | 109.0411 | 19.28936 |  |  |
| Adina pilulifera | 109.7566 | 18.70017 |  |  |
| Adinandra hainanensis | 108.8278 | 19.25573 | -43193.9 | 315921.6 |
| Adinandra hainanensis | 109.0333 | 18.86036 | -22982.8 | 271527.7 |
| Adinandra hainanensis | 109.0544 | 19.13823 | -19805.8 | 302178.8 |
| Adinandra hainanensis | 109.1191 | 19.05985 | -13271 | 293304.6 |
| Adinandra hainanensis | 109.3736 | 18.89125 | 12945.56 | 273868.1 |
| Adinandra hainanensis | 109.6807 | 18.37829 | 43729.57 | 216234.2 |
| Adinandra hainanensis | 109.6964 | 19.03697 | 47343.87 | 289030.5 |
| Adinandra hainanensis | 109.7575 | 19.47974 | 55069.1 | 337810.7 |
| Adinandra hainanensis | 110.0914 | 18.76565 | 88154.38 | 257960.3 |
| Adinandra hainanensis | 110.1756 | 18.76435 | 97019.43 | 257602 |
| Adinandra hainanensis | 110.2356 | 18.69662 | 103162.5 | 249961.2 |
| Adinandra hainanensis | 110.2559 | 19.09526 | 106327.7 | 293994.1 |
| Adinandra hainanensis | 109.7752 | 18.55977 |  |  |
| Adinandra hainanensis | 109.8263 | 18.5851 |  |  |
| Adinandra hainanensis | 109.8492 | 18.60649 |  |  |
| Adinandra hainanensis | 109.6492 | 18.24908 |  |  |
| Adinandra hainanensis | 109.5974 | 18.26158 |  |  |
| Adinandra hainanensis | 109.5922 | 18.22355 |  |  |
| Adinandra hainanensis | 108.7376 | 19.03988 |  |  |
| Adinandra hainanensis | 108.7466 | 19.06378 |  |  |
| Adinandra hainanensis | 108.8041 | 18.95283 |  |  |
| Adinandra hainanensis | 109.4682 | 19.22031 |  |  |
| Adinandra hainanensis | 109.3876 | 19.23561 |  |  |
| Adinandra hainanensis | 109.348 | 19.21375 |  |  |
| Adinandra hainanensis | 109.6838 | 19.97052 |  |  |
| Adinandra hainanensis | 109.7486 | 19.90121 |  |  |
| Adinandra hainanensis | 109.6352 | 19.89145 |  |  |
| Adinandra hainanensis | 109.0229 | 18.73566 |  |  |
| Adinandra hainanensis | 109.2858 | 18.78768 |  |  |
| Adinandra hainanensis | 109.1058 | 18.59105 |  |  |
| Adinandra hainanensis | 108.9877 | 19.30888 |  |  |
| Adinandra hainanensis | 109.1275 | 19.36574 |  |  |
| Aegiceras corniculatum | 110.0822 | 19.53483 | 89270.66 | 343031.3 |
| Aegiceras corniculatum | 110.2332 | 18.6893 | 102892.4 | 249157.1 |
| Aegiceras corniculatum | 110.6146 | 19.99826 | 146153.1 | 392988.4 |
| Aegiceras corniculatum | 109 | 19 |  |  |
| Aegiceras corniculatum | 110.8394 | 19.63444 |  |  |
| Aegiceras corniculatum | 110.7694 | 19.6118 |  |  |
| Aegiceras corniculatum | 110.7254 | 19.56328 |  |  |
| Aegiceras corniculatum | 110.7955 | 19.51539 |  |  |
| Aegiceras corniculatum | 110.7076 | 19.44289 |  |  |
| Aegiceras corniculatum | 110.7261 | 19.57169 |  |  |
| Aegiceras corniculatum | 110.7769 | 19.6021 |  |  |
| Aegiceras corniculatum | 109.5691 | 18.30003 |  |  |
| Aegiceras corniculatum | 109.6563 | 18.30329 |  |  |
| Aegiceras corniculatum | 109.543 | 18.33067 |  |  |
| Aegiceras corniculatum | 109.4078 | 18.33132 |  |  |
| Aegiceras corniculatum | 109.5616 | 18.36716 |  |  |
| Aegiceras corniculatum | 109.6556 | 18.35804 |  |  |
| Aegiceras corniculatum | 109.3542 | 18.34501 |  |  |
| Aegiceras corniculatum | 109.5087 | 18.38867 |  |  |
| Aegiceras corniculatum | 109.6344 | 18.40756 |  |  |
| Aeschynanthus acuminatus | 109.5153 | 18.61925 | 27012 | 243364.4 |
| Aeschynanthus acuminatus | 109.5153 | 18.61925 | 27012.11 | 243364.5 |
| Aeschynanthus acuminatus | 109.5153 | 18.61925 | 27012.22 | 243364.6 |
| Aeschynanthus acuminatus | 109.5153 | 18.61925 | 27012.32 | 243364.7 |
| Aeschynanthus acuminatus | 109.5153 | 18.61925 | 27012.43 | 243364.8 |
| Aeschynanthus acuminatus | 109.0912 | 19.27187 |  |  |
| Aeschynanthus acuminatus | 109.0253 | 19.2213 |  |  |
| Aeschynanthus acuminatus | 108.9566 | 19.17007 |  |  |
| Aeschynanthus acuminatus | 108.8873 | 19.26862 |  |  |
| Aeschynanthus acuminatus | 108.9491 | 19.31334 |  |  |
| Aeschynanthus acuminatus | 108.9704 | 19.38202 |  |  |
| Aeschynanthus acuminatus | 108.9127 | 19.37554 |  |  |
| Aeschynanthus acuminatus | 108.8234 | 19.27511 |  |  |
| Aeschynanthus acuminatus | 108.9724 | 108.9724 |  |  |
| Aeschynanthus acuminatus | 109.0425 | 19.35611 |  |  |
| Aeschynanthus acuminatus | 109.6893 | 18.57915 |  |  |
| Aeschynanthus acuminatus | 109.7538 | 18.68716 |  |  |
| Aeschynanthus acuminatus | 109.6344 | 18.69496 |  |  |
| Aeschynanthus acuminatus | 109.4682 | 18.66244 |  |  |
| Aeschynanthus acuminatus | 109.4242 | 18.55702 |  |  |
| Aeschynanthus acuminatus | 109.6302 | 18.60128 |  |  |
| Aeschynanthus acuminatus | 18.60128 | 18.64553 |  |  |
| Aeschynanthus acuminatus | 109.8349 | 18.63121 |  |  |
| Aeschynanthus acuminatus | 109.7071 | 18.47107 |  |  |
| Aeschynanthus moningeriae | 109.0333 | 18.86036 | -22982.8 | 271527.7 |
| Aeschynanthus moningeriae | 109.1191 | 19.05985 | -13271 | 293304.6 |
| Aeschynanthus moningeriae | 109.3011 | 18.85155 | 5180.119 | 269701.6 |
| Aeschynanthus moningeriae | 109.3683 | 19.19189 | 13342.08 | 307122.7 |
| Aeschynanthus moningeriae | 109.6579 | 18.76335 | 42477.13 | 258885.1 |
| Aeschynanthus moningeriae | 109.8188 | 19.14345 | 60528.46 | 300462.2 |
| Aeschynanthus moningeriae | 109.8628 | 18.65961 | 63764.9 | 246839.6 |
| Aeschynanthus moningeriae | 109.869 | 18.69141 | 64504.44 | 250339.5 |
| Aeschynanthus moningeriae | 109.8844 | 18.79929 | 66438.38 | 262227.7 |
| Aeschynanthus moningeriae | 110.2238 | 18.67552 | 101866.1 | 247657.4 |
| Aeschynanthus moningeriae | 109.9983 | 18.50363 |  |  |
| Aeschynanthus moningeriae | 110.0491 | 18.46717 |  |  |
| Aeschynanthus moningeriae | 109.9447 | 18.44502 |  |  |
| Aeschynanthus moningeriae | 109.9873 | 18.54009 |  |  |
| Aeschynanthus moningeriae | 109.6563 | 18.28601 |  |  |
| Aeschynanthus moningeriae | 109.5698 | 18.33816 |  |  |
| Aeschynanthus moningeriae | 109.4819 | 18.34207 |  |  |
| Aeschynanthus moningeriae | 109.5987 | 18.36423 |  |  |
| Aeschynanthus moningeriae | 109.7401 | 18.65073 |  |  |
| Aeschynanthus moningeriae | 109.6591 | 18.63121 |  |  |
| Aeschynanthus moningeriae | 109.7428 | 18.68846 |  |  |
| Aeschynanthus moningeriae | 109.5767 | 18.59737 |  |  |
| Aeschynanthus moningeriae | 109.8349 | 19.04323 |  |  |
| Aeschynanthus moningeriae | 109.8664 | 19.04842 |  |  |
| Aeschynanthus moningeriae | 109.7634 | 19.05102 |  |  |
| Aeschynanthus moningeriae | 109.7566 | 19.09125 |  |  |
| Aeschynanthus moningeriae | 109.7717 | 18.9848 |  |  |
| Aeschynanthus moningeriae | 109.5073 | 19.23297 |  |  |
| Aeschynanthus moningeriae | 109.4613 | 19.23103 |  |  |
| Aeschynanthus moningeriae | 108.6799 | 19.12921 |  |  |
| Aeschynanthus moningeriae | 110.3773 | 19.69522 |  |  |
| Aganosma schlechteriana | 109.1741 | 18.37558 | -9781.56 | 217466.3 |
| Aganosma schlechteriana | 109.3149 | 18.41558 | 5219.958 | 221446.7 |
| Aganosma schlechteriana | 109.4044 | 18.88006 | 16149.68 | 272536.9 |
| Aganosma schlechteriana | 109.4044 | 18.88006 | 16149.8 | 272537.1 |
| Aganosma schlechteriana | 109.6365 | 19.44697 | 42283.97 | 334529.2 |
| Aganosma schlechteriana | 109.6385 | 19.50569 |  |  |
| Aganosma schlechteriana | 109.4915 | 19.5691 |  |  |
| Aganosma schlechteriana | 109.4263 | 19.51798 |  |  |
| Aganosma schlechteriana | 109.609 | 19.57234 |  |  |
| Aganosma schlechteriana | 109.1551 | 18.74163 |  |  |
| Aganosma schlechteriana | 109.1578 | 18.75918 |  |  |
| Aganosma schlechteriana | 109.1389 | 18.75203 |  |  |
| Aganosma schlechteriana | 109.1262 | 18.72634 |  |  |
| Aganosma schlechteriana | 109.1437 | 18.70911 |  |  |
| Aganosma schlechteriana | 109.2169 | 18.73057 |  |  |
| Aganosma schlechteriana | 109.231 | 18.76991 |  |  |
| Aganosma schlechteriana | 109.1884 | 18.77803 |  |  |
| Aganosma schlechteriana | 109.1595 | 18.79396 |  |  |
| Aganosma schlechteriana | 109.1269 | 18.78194 |  |  |
| Aganosma schlechteriana | 109.0885 | 18.76666 |  |  |
| Aganosma schlechteriana | 109.1959 | 18.34598 |  |  |
| Aganosma schlechteriana | 109.2488 | 18.33947 |  |  |
| Aganosma schlechteriana | 109.1623 | 18.34012 |  |  |
| Aganosma schlechteriana | 109.2831 | 18.32187 |  |  |
| Aganosma schlechteriana | 109.2612 | 18.34077 |  |  |
| Aglaia elaeagnoidea | 109.4176 | 19.08662 | 18194.62 | 295334.7 |
| Aglaia elaeagnoidea | 109.8844 | 18.79929 | 66438.38 | 262227.7 |
| Aglaia elaeagnoidea | 110.001 | 18.75027 | 78585.68 | 256495.3 |
| Aglaia elaeagnoidea | 110.1841 | 18.62937 | 97550.3 | 242652.2 |
| Aglaia elaeagnoidea | 110.2356 | 18.69662 | 103162.5 | 249961.2 |
| Aglaia elaeagnoidea | 109.4555 | 19.20606 |  |  |
| Aglaia elaeagnoidea | 109.4527 | 19.41946 |  |  |
| Aglaia elaeagnoidea | 109.4088 | 19.25793 |  |  |
| Aglaia elaeagnoidea | 109.3738 | 19.21968 |  |  |
| Aglaia elaeagnoidea | 108.6775 | 19.13083 |  |  |
| Aglaia elaeagnoidea | 108.7798 | 19.08411 |  |  |
| Aglaia elaeagnoidea | 108.764 | 19.02505 |  |  |
| Aglaia elaeagnoidea | 109.8273 | 19.02521 |  |  |
| Aglaia elaeagnoidea | 109.8311 | 19.04631 |  |  |
| Aglaia elaeagnoidea | 109.8592 | 19.05085 |  |  |
| Aglaia elaeagnoidea | 109.8153 | 19.04793 |  |  |
| Aglaia elaeagnoidea | 109.8043 | 19.01905 |  |  |
| Aglaia elaeagnoidea | 109.8929 | 19.02619 |  |  |
| Aglaia elaeagnoidea | 110.3454 | 18.83085 |  |  |
| Aglaia elaeagnoidea | 110.3749 | 18.75999 |  |  |
| Aglaia elaeagnoidea | 110.3227 | 18.76259 |  |  |
| Aglaia elaeagnoidea | 109.7466 | 18.6247 |  |  |
| Aglaia elaeagnoidea | 110.0027 | 18.45414 |  |  |
| Aglaia elaeagnoidea | 109.5705 | 18.34215 |  |  |
| Aglaia lawii | 109.0333 | 18.86036 | -22982.8 | 271527.7 |
| Aglaia lawii | 109.0365 | 18.87698 | -22580.2 | 273355 |
| Aglaia lawii | 109.1191 | 19.05985 | -13271 | 293304.6 |
| Aglaia lawii | 109.1215 | 19.14164 | -12747.8 | 302339.7 |
| Aglaia lawii | 109.416 | 18.58215 | 16423.77 | 239559.1 |
| Aglaia lawii | 109.7345 | 18.74207 | 50478.48 | 256315 |
| Aglaia lawii | 109.8073 | 18.71975 | 58090.63 | 253642.9 |
| Aglaia lawii | 109.836 | 19.04117 | 62035.47 | 289106.2 |
| Aglaia lawii | 110.2163 | 19.94128 | 104388.5 | 387624 |
| Aglaia lawii | 109.6363 | 19.48845 |  |  |
| Aglaia lawii | 19.48845 | 19.51823 |  |  |
| Aglaia lawii | 109.5841 | 19.48133 |  |  |
| Aglaia lawii | 109.6425 | 19.54346 |  |  |
| Aglaia lawii | 109.1313 | 19.32314 |  |  |
| Aglaia lawii | 109.0884 | 19.30306 |  |  |
| Aglaia lawii | 109.0781 | 19.26708 |  |  |
| Aglaia lawii | 109.1416 | 19.28847 |  |  |
| Aglaia lawii | 109.4461 | 19.27648 |  |  |
| Aglaia lawii | 109.429 | 19.25315 |  |  |
| Aglaia lawii | 109.4094 | 19.24893 |  |  |
| Aglaia lawii | 108.8402 | 19.05418 |  |  |
| Aglaia lawii | 108.904 | 19.06262 |  |  |
| Aglaia lawii | 108.856 | 19.08598 |  |  |
| Aglaia lawii | 109.7742 | 18.63154 |  |  |
| Aglaia lawii | 109.7745 | 18.62373 |  |  |
| Aglaia lawii | 109.7476 | 18.58566 |  |  |
| Aglaia lawii | 109.9268 | 18.52748 |  |  |
| Aglaia lawii | 109.9728 | 18.49265 |  |  |
| Aglaia lawii | 109.5093 | 18.31934 |  |  |
| Aglaia odorata | 108.9795 | 18.89322 | -28525.9 | 275338 |
| Aglaia odorata | 109.0367 | 19.17511 | -21544.3 | 306313.7 |
| Aglaia odorata | 109.1191 | 19.05985 | -13271 | 293304.6 |
| Aglaia odorata | 109.4176 | 19.08662 | 18194.62 | 295334.7 |
| Aglaia odorata | 109.4865 | 19.15807 | 25651.28 | 303028.8 |
| Aglaia odorata | 109.6828 | 18.3841 | 43968.29 | 216870.9 |
| Aglaia odorata | 109.9847 | 19.22407 | 78181.06 | 308928.9 |
| Aglaia odorata | 110.0282 | 18.81462 | 81625.55 | 263539.8 |
| Aglaia odorata | 110.1756 | 18.76435 | 97019.43 | 257602 |
| Aglaia odorata | 110.2332 | 18.6893 | 102892.4 | 249157.1 |
| Aglaia odorata | 110.0908 | 19.83508 |  |  |
| Aglaia odorata | 110.1025 | 19.83152 |  |  |
| Aglaia odorata | 110.1005 | 19.80375 |  |  |
| Aglaia odorata | 109.121 | 19.17798 |  |  |
| Aglaia odorata | 109.2157 | 19.16954 |  |  |
| Aglaia odorata | 109.2212 | 19.1196 |  |  |
| Aglaia odorata | 110.3109 | 18.85209 |  |  |
| Aglaia odorata | 110.3459 | 18.81505 |  |  |
| Aglaia odorata | 110.2855 | 18.82414 |  |  |
| Aglaia odorata | 109.7692 | 18.60742 |  |  |
| Aglaia odorata | 109.7729 | 18.56739 |  |  |
| Aglaia odorata | 109.8227 | 18.57911 |  |  |
| Aglaia odorata | 109.7736 | 18.59083 |  |  |
| Aglaia odorata | 109.2892 | 18.7671 |  |  |
| Aglaia odorata | 109.3057 | 18.72744 |  |  |
| Aglaia odorata | 109.282 | 18.65069 |  |  |
| Aglaia odorata | 109.2068 | 18.67411 |  |  |
| Aglaia odorata | 110.0462 | 18.48145 |  |  |
| Aglaia odorata | 110.0332 | 18.48569 |  |  |
| Aglaia odorata | 109.524 | 18.39889 |  |  |
| Aidia canthioides | 109.0333 | 18.86036 | -22982.8 | 271527.7 |
| Aidia canthioides | 109.1215 | 19.14164 | -12747.8 | 302339.7 |
| Aidia canthioides | 109.2014 | 19.0858 | -4531.56 | 295912 |
| Aidia canthioides | 109.6365 | 19.44697 | 42283.97 | 334529.2 |
| Aidia canthioides | 109.7575 | 19.47974 | 55069.1 | 337810.7 |
| Aidia canthioides | 110.4448 | 19.64652 |  |  |
| Aidia canthioides | 110.4098 | 19.63359 |  |  |
| Aidia canthioides | 110.519 | 19.59607 |  |  |
| Aidia canthioides | 110.5039 | 19.66592 |  |  |
| Aidia canthioides | 109.8687 | 19.68144 |  |  |
| Aidia canthioides | 109.8488 | 19.63035 |  |  |
| Aidia canthioides | 109.7829 | 19.67626 |  |  |
| Aidia canthioides | 109.8014 | 19.90692 |  |  |
| Aidia canthioides | 109.6298 | 19.91208 |  |  |
| Aidia canthioides | 109.5487 | 19.91854 |  |  |
| Aidia canthioides | 109.6229 | 19.96437 |  |  |
| Aidia canthioides | 109.6359 | 19.22434 |  |  |
| Aidia canthioides | 109.6833 | 19.16209 |  |  |
| Aidia canthioides | 109.6202 | 19.15819 |  |  |
| Aidia canthioides | 109.1168 | 19.30083 |  |  |
| Aidia canthioides | 108.6623 | 19.03037 |  |  |
| Aidia canthioides | 109.1422 | 18.42755 |  |  |
| Aidia canthioides | 109.0935 | 18.41322 |  |  |
| Aidia canthioides | 109.98 | 18.55063 |  |  |
| Aidia canthioides | 109.728 | 18.61116 |  |  |
| Aidia cochinchinensis | 109.1741 | 18.37558 | -9781.56 | 217466.3 |
| Aidia cochinchinensis | 109.2343 | 18.80064 | -2022.54 | 264280.3 |
| Aidia cochinchinensis | 109.675 | 18.86962 | 44597.81 | 270587.7 |
| Aidia cochinchinensis | 110.0914 | 18.76565 | 88154.38 | 257960.3 |
| Aidia cochinchinensis | 110.2542 | 18.78692 | 105355.2 | 259902.1 |
| Aidia cochinchinensis | 109.2 | 18.7 |  |  |
| Aidia cochinchinensis | 109.7 | 18.6 |  |  |
| Aidia cochinchinensis | 109.5 | 18.3 |  |  |
| Aidia cochinchinensis | 110 | 18.5 |  |  |
| Aidia pycnantha | 108.9122 | 18.99917 | -35232.9 | 287275.8 |
| Aidia pycnantha | 109.0333 | 18.86036 | -22982.8 | 271527.7 |
| Aidia pycnantha | 109.0367 | 19.17511 | -21544.3 | 306313.7 |
| Aidia pycnantha | 109.3011 | 18.85155 | 5180.119 | 269701.6 |
| Aidia pycnantha | 109.4044 | 18.88006 | 16149.68 | 272536.9 |
| Aidia pycnantha | 109.4176 | 19.08662 | 18194.62 | 295334.7 |
| Aidia pycnantha | 109.4865 | 19.15807 | 25651.28 | 303028.8 |
| Aidia pycnantha | 109.4914 | 19.41113 | 26959.92 | 330989.8 |
| Aidia pycnantha | 109.5625 | 19.41661 | 34434.96 | 331387.3 |
| Aidia pycnantha | 109.6579 | 18.76335 | 42477.13 | 258885.1 |
| Aidia pycnantha | 109.6828 | 18.3841 | 43968.29 | 216870.9 |
| Aidia pycnantha | 109.777 | 19.17887 | 56233.56 | 304494 |
| Aidia pycnantha | 109.836 | 19.04117 | 62035.47 | 289106.2 |
| Aidia pycnantha | 109.869 | 18.69141 | 64504.44 | 250339.5 |
| Aidia pycnantha | 110.1756 | 18.76435 | 97019.43 | 257602 |
| Aidia pycnantha | 110.2253 | 18.686 | 102042.4 | 248812.9 |
| Aidia pycnantha | 110.4321 | 19.37563 | 125548.1 | 324570.5 |
| Aidia pycnantha | 109.684 | 18.60693 |  |  |
| Aidia pycnantha | 109.7444 | 18.60953 |  |  |
| Aidia pycnantha | 110.4105 | 18.81667 |  |  |
| Aidia pycnantha | 110.4242 | 18.83812 |  |  |
| Aidia pycnantha | 109.9326 | 19.01738 |  |  |
| Aidia pycnantha | 110.0699 | 19.0771 |  |  |
| Aidia pycnantha | 108.8539 | 19.06152 |  |  |
| Aidia pycnantha | 108.8463 | 19.00245 |  |  |
| Aidia pycnantha | 108.871 | 19.05503 |  |  |
| Aidia pycnantha | 109.1093 | 19.28463 |  |  |
| Aidia pycnantha | 109.2281 | 19.25675 |  |  |
| Aidia pycnantha | 109.2446 | 19.67885 |  |  |
| Aidia pycnantha | 109.3805 | 19.64523 |  |  |
| Aidia pycnantha | 109.2583 | 18.7198 |  |  |
| Aidia pycnantha | 109.1986 | 18.70419 |  |  |
| Aidia pycnantha | 109.329 | 18.74061 |  |  |
| Aidia pycnantha | 109.9099 | 18.54998 |  |  |
| Aidia pycnantha | 109.8358 | 18.5298 |  |  |
| Aidia pycnantha | 109.7486 | 18.43798 |  |  |
| Aidia pycnantha | 109.4636 | 18.4269 |  |  |
| Alangium barbatum | 109.6975 | 18.72833 | 46544.54 | 254900 |
| Alangium barbatum | 110.0282 | 18.81462 | 81625.55 | 263539.8 |
| Alangium barbatum | 110.0282 | 18.81462 | 81625.65 | 263539.9 |
| Alangium barbatum | 110.0282 | 18.81462 | 81625.76 | 263540 |
| Alangium barbatum | 110.0282 | 18.81462 | 81625.87 | 263540.1 |
| Alangium barbatum | 109.7801 | 18.61108 |  |  |
| Alangium barbatum | 109.7686 | 18.59855 |  |  |
| Alangium barbatum | 109.7995 | 18.59774 |  |  |
| Alangium barbatum | 109.7839 | 18.63727 |  |  |
| Alangium barbatum | 109.7578 | 18.62442 |  |  |
| Alangium barbatum | 109.7343 | 18.60051 |  |  |
| Alangium barbatum | 109.705 | 18.59481 |  |  |
| Alangium barbatum | 109.6732 | 18.58033 |  |  |
| Alangium barbatum | 109.654 | 18.53867 |  |  |
| Alangium barbatum | 109.6382 | 18.56992 |  |  |
| Alangium barbatum | 109.6428 | 18.59872 |  |  |
| Alangium barbatum | 109.7655 | 19.13792 |  |  |
| Alangium barbatum | 109.6502 | 19.13208 |  |  |
| Alangium barbatum | 109.7958 | 19.18981 |  |  |
| Alangium barbatum | 109.6838 | 19.11587 |  |  |
| Alangium barbatum | 109.6358 | 19.13079 |  |  |
| Alangium barbatum | 109.609 | 19.15738 |  |  |
| Alangium barbatum | 109.6186 | 19.11392 |  |  |
| Alangium barbatum | 109.7044 | 19.1256 |  |  |
| Alangium chinense | 109.0333 | 18.86036 | -22982.8 | 271527.7 |
| Alangium chinense | 109.5146 | 18.9427 | 27935.88 | 279133.3 |
| Alangium chinense | 110.1756 | 18.76435 | 97019.43 | 257602 |
| Alangium chinense | 110.2542 | 18.78692 | 105355.2 | 259902.1 |
| Alangium chinense | 109.977 | 19.73751 |  |  |
| Alangium chinense | 109.9516 | 19.73299 |  |  |
| Alangium chinense | 109.918 | 19.72523 |  |  |
| Alangium chinense | 109.4689 | 19.58588 |  |  |
| Alangium chinense | 109.5417 | 19.55483 |  |  |
| Alangium chinense | 109.4305 | 19.64668 |  |  |
| Alangium chinense | 109.3522 | 19.6441 |  |  |
| Alangium chinense | 109.4675 | 19.59106 |  |  |
| Alangium chinense | 109.5129 | 19.68289 |  |  |
| Alangium chinense | 109.1222 | 19.32545 |  |  |
| Alangium chinense | 109.1016 | 19.29175 |  |  |
| Alangium chinense | 109.0875 | 19.27523 |  |  |
| Alangium chinense | 109.0878 | 19.30958 |  |  |
| Alangium chinense | 109.1335 | 19.32513 |  |  |
| Alangium chinense | 109.1304 | 19.28074 |  |  |
| Alangium chinense | 109.1616 | 19.27361 |  |  |
| Alangium chinense | 109.5046 | 18.41436 |  |  |
| Alangium chinense | 109.4566 | 18.42218 |  |  |
| Alangium chinense | 109.4236 | 18.41241 |  |  |
| Alangium chinense | 109.5719 | 18.43456 |  |  |
| Alangium kurzii | 109.6036 | 18.37017 | 35565.66 | 215558.3 |
| Alangium kurzii | 109.6579 | 18.76335 | 42477.13 | 258885.1 |
| Alangium kurzii | 109.6807 | 18.37829 | 43729.57 | 216234.2 |
| Alangium kurzii | 109.8844 | 18.79929 | 66438.38 | 262227.7 |
| Alangium kurzii | 110.2356 | 18.69662 | 103162.5 | 249961.2 |
| Alangium kurzii | 109.4566 | 18.37331 |  |  |
| Alangium kurzii | 109.4559 | 18.39873 |  |  |
| Alangium kurzii | 109.4325 | 18.40785 |  |  |
| Alangium kurzii | 109.3721 | 18.39221 |  |  |
| Alangium kurzii | 109.3405 | 18.42544 |  |  |
| Alangium kurzii | 109.379 | 18.44172 |  |  |
| Alangium kurzii | 109.8122 | 18.64223 |  |  |
| Alangium kurzii | 109.8558 | 18.59619 |  |  |
| Alangium kurzii | 109.7841 | 18.56918 |  |  |
| Alangium kurzii | 110.2963 | 19.66236 |  |  |
| Alangium kurzii | 110.285 | 19.64102 |  |  |
| Alangium kurzii | 110.2719 | 19.66139 |  |  |
| Alangium kurzii | 110.3042 | 19.67368 |  |  |
| Alangium kurzii | 110.3173 | 19.64846 |  |  |
| Alangium kurzii | 110.2819 | 19.65234 |  |  |
| Alangium kurzii | 110.2919 | 19.60772 |  |  |
| Alangium kurzii | 110.2877 | 19.65945 |  |  |
| Alangium kurzii | 109.5105 | 18.34187 |  |  |
| Alangium kurzii | 109.5029 | 18.35621 |  |  |
| Alangium kurzii | 109.5125 | 18.37576 |  |  |
| Alangium salviifolium | 108.7804 | 18.80689 | -49793.4 | 266465.5 |
| Alangium salviifolium | 109.2773 | 19.16085 | 3684.27 | 303971.4 |
| Alangium salviifolium | 109.5153 | 18.61925 | 27012 | 243364.4 |
| Alangium salviifolium | 109.6401 | 19.49205 | 42797.58 | 339502 |
| Alangium salviifolium | 110.3426 | 20.03262 | 117817.4 | 397413.5 |
| Alangium salviifolium | 109.2 | 18.7 |  |  |
| Alangium salviifolium | 109.5 | 18.3 |  |  |
| Alangium salviifolium | 110.5342 | 19.89126 |  |  |
| Alangium salviifolium | 110.515 | 19.90482 |  |  |
| Alangium salviifolium | 110.4821 | 19.9003 |  |  |
| Alangium salviifolium | 110.4876 | 19.96937 |  |  |
| Alangium salviifolium | 109.5867 | 19.5816 |  |  |
| Alangium salviifolium | 109.5738 | 19.57044 |  |  |
| Alangium salviifolium | 109.5709 | 19.58645 |  |  |
| Alangium salviifolium | 109.5959 | 19.59826 |  |  |
| Alangium salviifolium | 109.6116 | 19.58144 |  |  |
| Alangium salviifolium | 108.9802 | 19.36432 |  |  |
| Alangium salviifolium | 108.9349 | 19.41225 |  |  |
| Alangium salviifolium | 109.3338 | 19.20845 |  |  |
| Alangium salviifolium | 109.364 | 19.21494 |  |  |
| Alangium salviifolium | 109.2961 | 19.16825 |  |  |
| Alangium salviifolium | 109.0743 | 18.74614 |  |  |
| Alangium salviifolium | 109.0468 | 18.71557 |  |  |
| Alangium salviifolium | 109.7589 | 18.57081 |  |  |
| Alangium salviifolium | 109.8303 | 18.62938 |  |  |
| Albizia corniculata | 109.0365 | 18.87698 | -22580.2 | 273355 |
| Albizia corniculata | 109.0799 | 19.00987 | -17563.9 | 287906.3 |
| Albizia corniculata | 109.1191 | 19.05985 | -13271 | 293304.6 |
| Albizia corniculata | 109.4718 | 19.16737 | 24145.48 | 304100.4 |
| Albizia corniculata | 109.6365 | 19.44697 | 42283.97 | 334529.2 |
| Albizia corniculata | 109.2 | 18.7 |  |  |
| Albizia corniculata | 109.7 | 18.6 |  |  |
| Albizia corniculata | 109.5 | 18.3 |  |  |
| Albizia corniculata | 110 | 18.5 |  |  |
| Albizia corniculata | 108.7956 | 19.03268 |  |  |
| Albizia corniculata | 108.7939 | 19.02684 |  |  |
| Albizia corniculata | 109.9296 | 18.98594 |  |  |
| Albizia corniculata | 109.954 | 19.02067 |  |  |
| Albizia corniculata | 109.3545 | 18.70456 |  |  |
| Albizia corniculata | 109.3614 | 18.6987 |  |  |
| Albizia corniculata | 109.313 | 18.66423 |  |  |
| Albizia corniculata | 109.6244 | 18.67919 |  |  |
| Albizia corniculata | 109.6217 | 18.71529 |  |  |
| Albizia corniculata | 109.9447 | 18.57931 |  |  |
| Albizia corniculata | 109.9372 | 18.59168 |  |  |
| Albizia corniculata | 109.9543 | 18.5689 |  |  |
| Albizia corniculata | 109.486 | 18.47075 |  |  |
| Albizia corniculata | 109.4572 | 18.4727 |  |  |
| Albizia corniculata | 109.5602 | 18.47921 |  |  |
| Albizia procera | 108.9648 | 19.13696 | -29229.1 | 302333.4 |
| Albizia procera | 109.0333 | 18.86036 | -22982.8 | 271527.7 |
| Albizia procera | 109.0367 | 19.17511 | -21544.3 | 306313.7 |
| Albizia procera | 109.1215 | 19.14164 | -12747.8 | 302339.7 |
| Albizia procera | 109.5409 | 18.34239 | 28849.41 | 212669.7 |
| Albizia procera | 109.6401 | 19.49205 | 42797.58 | 339502 |
| Albizia procera | 109.6555 | 18.5381 | 41546.81 | 233982.1 |
| Albizia procera | 109.6579 | 18.76335 | 42477.13 | 258885.1 |
| Albizia procera | 109.6807 | 18.37829 | 43729.57 | 216234.2 |
| Albizia procera | 109.6975 | 18.72833 | 46544.54 | 254900 |
| Albizia procera | 109.7327 | 19.49162 | 52506.25 | 339192.8 |
| Albizia procera | 109.8516 | 19.08029 | 63795.23 | 293388.2 |
| Albizia procera | 109.9249 | 18.56104 | 70034.61 | 235770.5 |
| Albizia procera | 109.7 | 18.6 |  |  |
| Albizia procera | 109.5 | 18.3 |  |  |
| Albizia procera | 110 | 18.5 |  |  |
| Alchornea trewioides | 109.0799 | 19.00987 | -17563.9 | 287906.3 |
| Alchornea trewioides | 109.4865 | 19.15807 | 25651.28 | 303028.8 |
| Alchornea trewioides | 109.654 | 19.43482 | 44083.3 | 333136.1 |
| Alchornea trewioides | 109.939 | 19.84766 | 75135.47 | 377987.1 |
| Alchornea trewioides | 109.9553 | 19.3256 | 75380.26 | 320231.5 |
| Alchornea trewioides | 110.1756 | 18.76435 | 97019.43 | 257602 |
| Alchornea trewioides | 110.2238 | 18.67552 | 101866.1 | 247657.4 |
| Alchornea trewioides | 109.7 | 18.6 |  |  |
| Alchornea trewioides | 109.5 | 18.2 |  |  |
| Aleurites moluccana | 109.0578 | 19.12083 | -19509.9 | 300245 |
| Aleurites moluccana | 109.2292 | 18.32242 | -4135.79 | 211411.6 |
| Aleurites moluccana | 109.4914 | 19.41113 | 26959.92 | 330989.8 |
| Aleurites moluccana | 109.5132 | 19.56355 | 29719.98 | 347774.6 |
| Aleurites moluccana | 109.5259 | 19.13355 | 29723.31 | 300201.6 |
| Alniphyllum fortunei | 109.0333 | 18.86036 | -22982.8 | 271527.7 |
| Alniphyllum fortunei | 109.5146 | 18.9427 | 27935.88 | 279133.3 |
| Alniphyllum fortunei | 109.5377 | 19.09747 | 30846.03 | 296177.5 |
| Alniphyllum fortunei | 110.1756 | 18.76435 | 97019.43 | 257602 |
| Alniphyllum fortunei | 109.7 | 18.6 |  |  |
| Alniphyllum fortunei | 110.5 | 19.3 |  |  |
| Alniphyllum fortunei | 109.5 | 18.3 |  |  |
| Alniphyllum fortunei | 110 | 18.5 |  |  |
| Alniphyllum fortunei | 109.8671 | 19.68293 |  |  |
| Alniphyllum fortunei | 109.8884 | 19.68875 |  |  |
| Alniphyllum fortunei | 109.8678 | 19.69457 |  |  |
| Alniphyllum fortunei | 109.9427 | 18.57883 |  |  |
| Alniphyllum fortunei | 109.9475 | 18.56711 |  |  |
| Alniphyllum fortunei | 109.1577 | 18.66179 |  |  |
| Alniphyllum fortunei | 109.1642 | 18.67675 |  |  |
| Alniphyllum fortunei | 109.1841 | 18.66146 |  |  |
| Alniphyllum fortunei | 109.4656 | 19.12499 |  |  |
| Alniphyllum fortunei | 109.4896 | 19.13018 |  |  |
| Alniphyllum fortunei | 109.4842 | 19.10747 |  |  |
| Alniphyllum fortunei | 109.5226 | 18.4131 |  |  |
| Alniphyllum fortunei | 109.5549 | 18.39486 |  |  |
| Alniphyllum fortunei | 18.39486 | 18.42027 |  |  |
| Alniphyllum fortunei | 109.5782 | 18.4118 |  |  |
| Alniphyllum fortunei | 109.5727 | 18.38508 |  |  |
| Alphonsea monogyna | 108.9122 | 18.99917 | -35232.9 | 287275.8 |
| Alphonsea monogyna | 108.9515 | 18.90932 | -31419.3 | 277210.7 |
| Alphonsea monogyna | 109.0333 | 18.86036 | -22982.8 | 271527.7 |
| Alphonsea monogyna | 109.6538 | 18.29652 | 40638.73 | 207266.3 |
| Alphonsea monogyna | 109.6807 | 18.37829 | 43729.57 | 216234.2 |
| Alphonsea monogyna | 109.7 | 18.6 |  |  |
| Alphonsea monogyna | 110.5 | 19.3 |  |  |
| Alphonsea monogyna | 109.5 | 18.3 |  |  |
| Alphonsea monogyna | 109.2 | 18.7 |  |  |
| Alphonsea monogyna | 109.1 | 19.2 |  |  |
| Alphonsea monogyna | 109.4636 | 18.47368 |  |  |
| Alphonsea monogyna | 109.5549 | 18.47824 |  |  |
| Alphonsea monogyna | 109.5164 | 18.4587 |  |  |
| Alphonsea monogyna | 109.7828 | 18.60323 |  |  |
| Alphonsea monogyna | 109.8199 | 18.57915 |  |  |
| Alphonsea monogyna | 109.8021 | 18.57525 |  |  |
| Alphonsea monogyna | 109.8268 | 18.59217 |  |  |
| Alphonsea monogyna | 110.0108 | 18.61364 |  |  |
| Alphonsea monogyna | 110.0039 | 18.6208 |  |  |
| Alphonsea monogyna | 110.0163 | 18.6709 |  |  |
| Alphonsea monogyna | 110.0335 | 18.47303 |  |  |
| Alphonsea monogyna | 109.0461 | 18.73203 |  |  |
| Alphonsea monogyna | 109.1216 | 19.30135 |  |  |
| Alphonsea monogyna | 109.857 | 19.04339 |  |  |
| Alphonsea monogyna | 110.3401 | 19.25793 |  |  |
| Alseodaphne hainanensis | 109.0333 | 18.86036 | -22982.8 | 271527.7 |
| Alseodaphne hainanensis | 109.1215 | 19.14164 | -12747.8 | 302339.7 |
| Alseodaphne hainanensis | 109.3683 | 19.19189 | 13342.08 | 307122.7 |
| Alseodaphne hainanensis | 109.6006 | 18.43923 | 35449.67 | 223205.2 |
| Alseodaphne hainanensis | 109.6835 | 18.87437 | 45503.17 | 271088.8 |
| Alseodaphne hainanensis | 109.8526 | 18.71296 | 62839.29 | 252768.1 |
| Alseodaphne hainanensis | 109.8844 | 18.79929 | 66438.38 | 262227.7 |
| Alseodaphne hainanensis | 110.0914 | 18.76565 | 88154.38 | 257960.3 |
| Alseodaphne hainanensis | 109.7 | 18.6 |  |  |
| Alseodaphne hainanensis | 109.2 | 18.7 |  |  |
| Alseodaphne hainanensis | 109.5 | 18.3 |  |  |
| Alseodaphne hainanensis | 109.685 | 18.90492 |  |  |
| Alseodaphne hainanensis | 109.8999 | 18.74 |  |  |
| Alseodaphne hainanensis | 109.8803 | 18.71009 |  |  |
| Alseodaphne hainanensis | 109.9027 | 18.67496 |  |  |
| Alseodaphne hainanensis | 108.94 | 18.73236 |  |  |
| Alseodaphne hainanensis | 108.8631 | 18.70505 |  |  |
| Alseodaphne hainanensis | 109.1185 | 19.08557 |  |  |
| Alseodaphne hainanensis | 109.1182 | 19.07616 |  |  |
| Alseodaphne rugosa | 109.3116 | 18.96725 | 6666.857 | 282460.4 |
| Alseodaphne rugosa | 109.6835 | 18.87437 | 45503.17 | 271088.8 |
| Alseodaphne rugosa | 109.869 | 18.69141 | 64504.44 | 250339.5 |
| Alseodaphne rugosa | 110.2542 | 18.78692 | 105355.2 | 259902.1 |
| Alseodaphne rugosa | 110.6146 | 19.99826 | 146153.1 | 392988.4 |
| Alseodaphne rugosa | 109.7 | 18.6 |  |  |
| Alseodaphne rugosa | 109.1288 | 19.08557 |  |  |
| Alseodaphne rugosa | 109.1226 | 19.07779 |  |  |
| Alseodaphne rugosa | 109.8972 | 18.73025 |  |  |
| Alseodaphne rugosa | 109.8803 | 18.71691 |  |  |
| Alseodaphne rugosa | 109.8553 | 18.71204 |  |  |
| Alseodaphne rugosa | 109.8591 | 18.68992 |  |  |
| Alseodaphne rugosa | 108.9091 | 18.69773 |  |  |
| Alseodaphne rugosa | 108.8974 | 18.70098 |  |  |
| Alseodaphne rugosa | 108.9657 | 18.69578 |  |  |
| Alseodaphne rugosa | 108.9105 | 18.74163 |  |  |
| Alseodaphne rugosa | 108.8778 | 18.77348 |  |  |
| Alseodaphne rugosa | 109.1072 | 19.0627 |  |  |
| Alseodaphne rugosa | 109.1401 | 19.05166 |  |  |
| Alseodaphne rugosa | 109.5355 | 19.03142 |  |  |
| Alseodaphne rugosa | 109.5067 | 19.0259 |  |  |
| Alseodaphne rugosa | 109.4875 | 19.00935 |  |  |
| Alseodaphne rugosa | 109.4456 | 18.9902 |  |  |
| Altingia chinensis | 108.9086 | 18.47293 | -37468 | 229101.3 |
| Altingia chinensis | 109.0333 | 18.86036 | -22982.8 | 271527.7 |
| Altingia chinensis | 109.8844 | 18.79929 | 66438.38 | 262227.7 |
| Altingia chinensis | 109.0442 | 18.72427 |  |  |
| Altingia chinensis | 109.0319 | 18.71907 |  |  |
| Altingia chinensis | 109.0532 | 18.69826 |  |  |
| Altingia chinensis | 109.0401 | 18.682 |  |  |
| Altingia chinensis | 109.0298 | 18.65793 |  |  |
| Altingia chinensis | 108.9831 | 18.64752 |  |  |
| Altingia chinensis | 109.1122 | 18.59221 |  |  |
| Altingia chinensis | 109.115 | 18.55641 |  |  |
| Altingia chinensis | 109.1115 | 18.70281 |  |  |
| Altingia chinensis | 109.9417 | 19.01471 |  |  |
| Altingia chinensis | 109.9424 | 19.04197 |  |  |
| Altingia chinensis | 109.9739 | 19.05365 |  |  |
| Altingia chinensis | 110.0069 | 19.00042 |  |  |
| Altingia chinensis | 109.9939 | 18.95367 |  |  |
| Altingia chinensis | 110.046 | 18.93679 |  |  |
| Altingia chinensis | 110.0749 | 18.96536 |  |  |
| Altingia chinensis | 110.0378 | 19.00367 |  |  |
| Altingia chinensis | 110.0083 | 19.04716 |  |  |
| Altingia chinensis | 109.9925 | 19.03029 |  |  |
| Altingia chinensis | 110.0076 | 19.06533 |  |  |
| Altingia gracilipes | 110.2542 | 18.78692 | 105355.2 | 259902.1 |
| Altingia obovata | 109.0333 | 18.86036 | -22982.8 | 271527.7 |
| Altingia obovata | 109.429 | 18.50349 | 17547.73 | 230821.1 |
| Altingia obovata | 109.6835 | 18.87437 | 45503.17 | 271088.8 |
| Altingia obovata | 109.8795 | 18.7193 | 65691.55 | 253395.3 |
| Altingia obovata | 109.9578 | 18.76128 | 74063.58 | 257827 |
| Altingia obovata | 110.2238 | 18.67552 | 101866.1 | 247657.4 |
| Altingia obovata | 110.3309 | 19.265 | 114648.9 | 312579.5 |
| Altingia obovata | 109.827 | 19.09454 |  |  |
| Altingia obovata | 109.7879 | 19.0835 |  |  |
| Altingia obovata | 109.7995 | 19.10297 |  |  |
| Altingia obovata | 109.7851 | 19.12633 |  |  |
| Altingia obovata | 109.8497 | 19.10881 |  |  |
| Altingia obovata | 109.3601 | 19.21518 |  |  |
| Altingia obovata | 109.3464 | 19.23722 |  |  |
| Altingia obovata | 109.3518 | 19.25278 |  |  |
| Altingia obovata | 109.3415 | 19.27223 |  |  |
| Altingia obovata | 109.2962 | 19.18081 |  |  |
| Altingia obovata | 109.3258 | 19.10816 |  |  |
| Altingia obovata | 109.2873 | 19.09843 |  |  |
| Altingia obovata | 109.507 | 18.41835 |  |  |
| Altingia obovata | 109.5997 | 18.41575 |  |  |
| Altingia obovata | 109.5832 | 18.42943 |  |  |
| Altingia obovata | 109.5338 | 18.42812 |  |  |
| Altingia obovata | 109.7789 | 18.60067 |  |  |
| Altingia obovata | 109.8332 | 18.58049 |  |  |
| Alyxia sinensis | 109.5064 | 18.29956 | 25081.02 | 208034 |
| Alyxia sinensis | 109.6828 | 18.3841 | 43968.29 | 216870.9 |
| Alyxia sinensis | 110.1756 | 18.76435 | 97019.43 | 257602 |
| Alyxia sinensis | 110.1876 | 19.13499 | 99259.69 | 298557.2 |
| Alyxia sinensis | 110.2738 | 18.67292 | 107120.4 | 247246.1 |
| Alyxia sinensis | 109.2 | 18.7 |  |  |
| Alyxia sinensis | 109.7 | 18.6 |  |  |
| Alyxia sinensis | 110.3523 | 18.71874 |  |  |
| Alyxia sinensis | 110.3557 | 18.70704 |  |  |
| Alyxia sinensis | 110.3887 | 18.70834 |  |  |
| Alyxia sinensis | 110.3543 | 18.7376 |  |  |
| Alyxia sinensis | 110.3907 | 18.75321 |  |  |
| Alyxia sinensis | 110.4299 | 18.7454 |  |  |
| Alyxia sinensis | 110.445 | 18.75776 |  |  |
| Alyxia sinensis | 110.3152 | 18.84811 |  |  |
| Alyxia sinensis | 110.3372 | 18.84031 |  |  |
| Amesiodendron chinense | 108.8974 | 18.5739 | -38293.2 | 240304.7 |
| Amesiodendron chinense | 109.0333 | 18.86036 | -22982.8 | 271527.7 |
| Amesiodendron chinense | 109.0365 | 18.87698 | -22580.2 | 273355 |
| Amesiodendron chinense | 109.3011 | 18.85155 | 5180.119 | 269701.6 |
| Amesiodendron chinense | 109.869 | 18.69141 | 64504.44 | 250339.5 |
| Amesiodendron chinense | 109.2 | 18.7 |  |  |
| Amesiodendron chinense | 109.7 | 18.6 |  |  |
| Amesiodendron chinense | 110.5 | 19.3 |  |  |
| Amesiodendron chinense | 109.5 | 18.3 |  |  |
| Amesiodendron chinense | 110 | 18.5 |  |  |
| Amesiodendron chinense | 109.6368 | 18.67842 |  |  |
| Amesiodendron chinense | 109.6416 | 18.69273 |  |  |
| Amesiodendron chinense | 109.6492 | 18.65305 |  |  |
| Amesiodendron chinense | 109.7837 | 18.60945 |  |  |
| Amesiodendron chinense | 109.7927 | 18.59969 |  |  |
| Amesiodendron chinense | 109.8284 | 18.59058 |  |  |
| Amesiodendron chinense | 109.7968 | 18.62442 |  |  |
| Amesiodendron chinense | 109.8291 | 18.61271 |  |  |
| Amesiodendron chinense | 109.7295 | 18.58863 |  |  |
| Amesiodendron chinense | 109.4493 | 19.30561 |  |  |
| Amesiodendron chinense | 109.4384 | 19.29783 |  |  |
| Amesiodendron chinense | 109.4892 | 19.30107 |  |  |
| Amesiodendron chinense | 109.4885 | 19.33088 |  |  |
| Amesiodendron chinense | 109.4027 | 19.33736 |  |  |
| Ampelopsis cantoniensis | 108.982 | 18.6052 | -29265.1 | 243483.5 |
| Ampelopsis cantoniensis | 108.9868 | 19.0313 | -27277.7 | 290579.7 |
| Ampelopsis cantoniensis | 109.1191 | 19.05985 | -13271 | 293304.6 |
| Ampelopsis cantoniensis | 109.2343 | 18.80064 | -2022.54 | 264280.3 |
| Ampelopsis cantoniensis | 109.4546 | 19.30628 | 22766.04 | 319508.9 |
| Ampelopsis cantoniensis | 109.6964 | 19.03697 | 47343.87 | 289030.5 |
| Ampelopsis cantoniensis | 109.837 | 18.73456 | 61260.82 | 255199 |
| Ampelopsis cantoniensis | 110.0221 | 18.80753 | 80965.02 | 262772.1 |
| Ampelopsis cantoniensis | 110.1592 | 18.68652 | 95078.4 | 249035.8 |
| Ampelopsis cantoniensis | 110.1756 | 18.76435 | 97019.43 | 257602 |
| Ampelopsis cantoniensis | 109.1091 | 19.28892 |  |  |
| Ampelopsis cantoniensis | 109.1256 | 19.28471 |  |  |
| Ampelopsis cantoniensis | 109.1201 | 19.30156 |  |  |
| Ampelopsis cantoniensis | 109.1108 | 19.31128 |  |  |
| Ampelopsis cantoniensis | 109.0998 | 19.321 |  |  |
| Ampelopsis cantoniensis | 109.1483 | 19.30966 |  |  |
| Ampelopsis cantoniensis | 108.7936 | 19.02655 |  |  |
| Ampelopsis cantoniensis | 108.786 | 19.01292 |  |  |
| Ampelopsis cantoniensis | 108.7703 | 19.00708 |  |  |
| Ampelopsis cantoniensis | 108.8454 | 19.03629 |  |  |
| Ampelopsis cantoniensis | 108.7843 | 19.0272 |  |  |
| Ampelopsis cantoniensis | 108.7394 | 19.04181 |  |  |
| Ampelopsis cantoniensis | 108.7414 | 19.03434 |  |  |
| Ampelopsis cantoniensis | 108.7291 | 19.05219 |  |  |
| Ampelopsis cantoniensis | 109.2878 | 18.78945 |  |  |
| Ampelopsis cantoniensis | 109.3503 | 18.77352 |  |  |
| Ampelopsis cantoniensis | 109.3603 | 18.7576 |  |  |
| Ampelopsis cantoniensis | 109.3647 | 18.76702 |  |  |
| Ampelopsis cantoniensis | 109.3383 | 18.75272 |  |  |
| Ampelopsis cantoniensis | 109.3582 | 18.6994 |  |  |
| Anaxagorea luzonensis | 109.6734 | 18.36147 | 42902.58 | 214395.2 |
| Anaxagorea luzonensis | 109.68 | 18.68397 | 44567.71 | 250043.4 |
| Anaxagorea luzonensis | 109.6975 | 18.72833 | 46544.54 | 254900 |
| Anaxagorea luzonensis | 109.837 | 18.73456 | 61260.82 | 255199 |
| Anaxagorea luzonensis | 110.2332 | 18.6893 | 102892.4 | 249157.1 |
| Anaxagorea luzonensis | 109.7 | 18.6 |  |  |
| Anaxagorea luzonensis | 110 | 18.5 |  |  |
| Anaxagorea luzonensis | 110.5 | 19.3 |  |  |
| Anaxagorea luzonensis | 109.542 | 18.26173 |  |  |
| Anaxagorea luzonensis | 109.5399 | 18.24347 |  |  |
| Anaxagorea luzonensis | 109.5475 | 18.27998 |  |  |
| Anaxagorea luzonensis | 109.8826 | 18.47286 |  |  |
| Anaxagorea luzonensis | 109.8661 | 18.4644 |  |  |
| Anaxagorea luzonensis | 109.8716 | 18.5178 |  |  |
| Anaxagorea luzonensis | 109.864 | 18.52886 |  |  |
| Anaxagorea luzonensis | 109.9066 | 18.53472 |  |  |
| Anaxagorea luzonensis | 109.6189 | 18.67269 |  |  |
| Anaxagorea luzonensis | 109.6642 | 18.67008 |  |  |
| Anaxagorea luzonensis | 109.6285 | 18.70261 |  |  |
| Anaxagorea luzonensis | 109.4788 | 19.12158 |  |  |
| Anaxagorea luzonensis | 109.4768 | 19.1391 |  |  |
| Anaxagorea luzonensis | 109.4493 | 19.10731 |  |  |
| Anaxagorea luzonensis | 109.5468 | 19.15078 |  |  |
| Anaxagorea luzonensis | 110.3117 | 18.84758 |  |  |
| Anaxagorea luzonensis | 110.3076 | 18.86513 |  |  |
| Anneslea fragrans | 109.6579 | 18.76335 | 42477.13 | 258885.1 |
| Anneslea fragrans | 109.8844 | 18.79929 | 66438.38 | 262227.7 |
| Anneslea fragrans | 109.8844 | 18.79929 | 66438.49 | 262227.8 |
| Anneslea fragrans | 109.8844 | 18.79929 | 66438.6 | 262227.9 |
| Anneslea fragrans | 109.8844 | 18.79929 | 66438.71 | 262228 |
| Anneslea fragrans | 109.7 | 18.6 |  |  |
| Anneslea fragrans | 110 | 18.5 |  |  |
| Anneslea fragrans | 109.6265 | 18.67984 |  |  |
| Anneslea fragrans | 109.6306 | 18.66943 |  |  |
| Anneslea fragrans | 109.658 | 18.64406 |  |  |
| Anneslea fragrans | 109.7803 | 18.60892 |  |  |
| Anneslea fragrans | 109.8208 | 18.58354 |  |  |
| Anneslea fragrans | 109.7926 | 18.61608 |  |  |
| Anneslea fragrans | 109.8331 | 18.55035 |  |  |
| Anneslea fragrans | 109.77 | 18.52235 |  |  |
| Anneslea fragrans | 109.9334 | 18.57964 |  |  |
| Anneslea fragrans | 109.9485 | 18.54449 |  |  |
| Anneslea fragrans | 109.8675 | 18.5191 |  |  |
| Anneslea fragrans | 109.8929 | 18.52561 |  |  |
| Anneslea fragrans | 109.8716 | 18.47352 |  |  |
| Anneslea fragrans | 110.068 | 18.50477 |  |  |
| Anodendron affine | 109.5064 | 18.29956 | 25081.02 | 208034 |
| Anodendron affine | 109.6828 | 18.3841 | 43968.29 | 216870.9 |
| Anodendron affine | 110.1756 | 18.76435 | 97019.43 | 257602 |
| Anodendron affine | 110.2738 | 18.67292 | 107120.4 | 247246.1 |
| Anodendron affine | 110.7217 | 19.71514 | 156705.5 | 361456.5 |
| Anodendron affine | 109.7 | 18.6 |  |  |
| Anodendron affine | 109.5 | 18.3 |  |  |
| Anodendron affine | 109.5592 | 18.59266 |  |  |
| Anodendron affine | 109.531 | 18.58615 |  |  |
| Anodendron affine | 109.4933 | 18.5881 |  |  |
| Anodendron affine | 109.5084 | 18.65447 |  |  |
| Anodendron affine | 108.8018 | 18.95542 |  |  |
| Anodendron affine | 108.7929 | 18.93204 |  |  |
| Anodendron affine | 108.8128 | 18.9119 |  |  |
| Anodendron affine | 108.8155 | 18.89371 |  |  |
| Anodendron affine | 109.4706 | 19.30249 |  |  |
| Anodendron affine | 109.406 | 19.34072 |  |  |
| Anodendron affine | 109.4054 | 19.35886 |  |  |
| Anodendron affine | 109.5125 | 19.44888 |  |  |
| Anodendron affine | 109.4953 | 19.46053 |  |  |
| Anodendron affine | 109.4747 | 19.47348 |  |  |
| Anodendron affine | 110.057 | 19.76825 |  |  |
| Anodendron affine | 110.0546 | 19.776 |  |  |
| Anodendron affine | 110.7872 | 19.70055 |  |  |
| Antiaris toxicaria | 109.0333 | 18.86036 | -22982.8 | 271527.7 |
| Antiaris toxicaria | 109.3011 | 18.85155 | 5180.119 | 269701.6 |
| Antiaris toxicaria | 109.837 | 18.73456 | 61260.82 | 255199 |
| Antiaris toxicaria | 110.1756 | 18.76435 | 97019.43 | 257602 |
| Antiaris toxicaria | 110.416 | 19.24936 | 123547.9 | 310647 |
| Antiaris toxicaria | 110.0978 | 19.71687 |  |  |
| Antiaris toxicaria | 110.0954 | 19.70362 |  |  |
| Antiaris toxicaria | 110.0917 | 19.72721 |  |  |
| Antiaris toxicaria | 110.0697 | 19.73368 |  |  |
| Antiaris toxicaria | 111.0231 | 19.64575 |  |  |
| Antiaris toxicaria | 111.0307 | 19.64478 |  |  |
| Antiaris toxicaria | 111.0183 | 19.64414 |  |  |
| Antiaris toxicaria | 110.3728 | 18.85099 |  |  |
| Antiaris toxicaria | 110.3399 | 18.83507 |  |  |
| Antiaris toxicaria | 110.3159 | 18.85327 |  |  |
| Antiaris toxicaria | 110.3134 | 18.83247 |  |  |
| Antiaris toxicaria | 109.7782 | 18.60388 |  |  |
| Antiaris toxicaria | 109.7535 | 18.60486 |  |  |
| Antiaris toxicaria | 109.7748 | 18.56743 |  |  |
| Antiaris toxicaria | 109.8235 | 18.57915 |  |  |
| Antiaris toxicaria | 109.8404 | 18.56906 |  |  |
| Antiaris toxicaria | 109.9379 | 18.57956 |  |  |
| Antiaris toxicaria | 109.937 | 18.57419 |  |  |
| Antiaris toxicaria | 109.9569 | 18.58346 |  |  |
| Antiaris toxicaria | 109.947 | 18.5942 |  |  |
| Antidesma bunius | 109.1191 | 19.05985 | -13271 | 293304.6 |
| Antidesma bunius | 109.1544 | 19.01211 | -9728.08 | 287914.8 |
| Antidesma bunius | 109.4176 | 19.08662 | 18194.62 | 295334.7 |
| Antidesma bunius | 109.4583 | 19.56476 | 23975.71 | 348070.6 |
| Antidesma bunius | 109.6975 | 18.72833 | 46544.54 | 254900 |
| Antidesma bunius | 110.5 | 19.3 |  |  |
| Antidesma bunius | 109.2 | 18.7 |  |  |
| Antidesma bunius | 109.5 | 18.3 |  |  |
| Antidesma bunius | 109.5839 | 18.18982 |  |  |
| Antidesma bunius | 109.6007 | 18.18395 |  |  |
| Antidesma bunius | 109.6014 | 18.21135 |  |  |
| Antidesma bunius | 108.7355 | 19.0455 |  |  |
| Antidesma bunius | 108.7922 | 19.03187 |  |  |
| Antidesma bunius | 108.7551 | 19.01921 |  |  |
| Antidesma bunius | 109.5077 | 19.20574 |  |  |
| Antidesma bunius | 109.5039 | 19.22422 |  |  |
| Antidesma bunius | 109.5259 | 19.20152 |  |  |
| Antidesma bunius | 109.4751 | 19.35578 |  |  |
| Antidesma bunius | 109.4599 | 19.38687 |  |  |
| Antidesma bunius | 109.5904 | 19.45034 |  |  |
| Antidesma bunius | 109.6151 | 19.45811 |  |  |
| Antidesma bunius | 109.6481 | 19.43221 |  |  |
| Antidesma bunius | 110.423 | 19.72107 |  |  |
| Antidesma bunius | 110.448 | 19.7075 |  |  |
| Antidesma bunius | 110.4168 | 19.69522 |  |  |
| Antidesma japonicum | 109.0333 | 18.86036 | -22982.8 | 271527.7 |
| Antidesma japonicum | 109.1215 | 19.14164 | -12747.8 | 302339.7 |
| Antidesma japonicum | 109.5425 | 19.03583 | 31162.38 | 289348.8 |
| Antidesma japonicum | 109.8188 | 19.14345 | 60528.46 | 300462.2 |
| Antidesma japonicum | 110.4194 | 18.9871 | 123247.6 | 281640.2 |
| Antidesma japonicum | 109.7 | 18.6 |  |  |
| Antidesma japonicum | 109.5 | 18.3 |  |  |
| Antidesma japonicum | 109.6824 | 18.90784 |  |  |
| Antidesma japonicum | 109.6871 | 18.90224 |  |  |
| Antidesma japonicum | 109.6925 | 18.89883 |  |  |
| Antidesma japonicum | 109.6984 | 18.89639 |  |  |
| Antidesma japonicum | 109.6939 | 18.9024 |  |  |
| Antidesma japonicum | 108.9007 | 19.06416 |  |  |
| Antidesma japonicum | 108.8897 | 19.05637 |  |  |
| Antidesma japonicum | 108.797 | 18.94925 |  |  |
| Antidesma japonicum | 108.7991 | 18.95704 |  |  |
| Antidesma japonicum | 108.8004 | 18.94145 |  |  |
| Antidesma japonicum | 108.8203 | 18.90118 |  |  |
| Antidesma japonicum | 108.7661 | 18.9999 |  |  |
| Antidesma maclurei | 109.1191 | 19.05985 | -13271 | 293304.6 |
| Antidesma maclurei | 109.3233 | 18.69505 | 7006.586 | 252327.2 |
| Antidesma maclurei | 109.4273 | 19.01928 | 18993.09 | 287860.9 |
| Antidesma maclurei | 109.6538 | 18.29652 | 40638.73 | 207266.3 |
| Antidesma maclurei | 109.8188 | 19.14345 | 60528.46 | 300462.2 |
| Antidesma maclurei | 109.837 | 18.73456 | 61260.82 | 255199 |
| Antidesma maclurei | 110.0221 | 18.80753 | 80965.02 | 262772.1 |
| Antidesma maclurei | 110.1756 | 18.76435 | 97019.43 | 257602 |
| Antidesma maclurei | 110 | 18.5 |  |  |
| Antidesma maclurei | 109.5 | 18.3 |  |  |
| Antidesma maclurei | 109.8 | 19.2 |  |  |
| Antidesma maclurei | 109.7796 | 18.60535 |  |  |
| Antidesma maclurei | 109.7837 | 18.62031 |  |  |
| Antidesma maclurei | 109.8173 | 18.58257 |  |  |
| Antidesma maclurei | 109.5938 | 18.26221 |  |  |
| Antidesma maclurei | 109.5914 | 18.24493 |  |  |
| Antidesma maclurei | 109.5983 | 18.23026 |  |  |
| Antidesma maclurei | 110.0354 | 18.47172 |  |  |
| Antidesma maclurei | 110.0295 | 18.48703 |  |  |
| Antidesma maclurei | 109.9784 | 18.49061 |  |  |
| Antidesma maclurei | 109.9701 | 18.49484 |  |  |
| Antidesma maclurei | 108.8069 | 18.97636 |  |  |
| Antidesma maclurei | 108.7901 | 18.94941 |  |  |
| Antidesma maclurei | 108.7984 | 18.94454 |  |  |
| Antidesma maclurei | 108.8039 | 18.97019 |  |  |
| Antidesma maclurei | 109.8228 | 19.00347 |  |  |
| Antidesma maclurei | 109.8276 | 18.99438 |  |  |
| Antidesma maclurei | 109.7981 | 18.98464 |  |  |
| Antidesma montanum | 109.0333 | 18.86036 | -22982.8 | 271527.7 |
| Antidesma montanum | 109.6807 | 18.37829 | 43729.57 | 216234.2 |
| Antidesma montanum | 109.8516 | 19.08029 | 63795.23 | 293388.2 |
| Antidesma montanum | 110.0221 | 18.80753 | 80965.02 | 262772.1 |
| Antidesma montanum | 110.1756 | 18.76435 | 97019.43 | 257602 |
| Antidesma montanum | 110.4321 | 19.37563 | 125548.1 | 324570.5 |
| Antidesma montanum | 110.5 | 19.3 |  |  |
| Antidesma montanum | 109.7 | 18.6 |  |  |
| Antidesma montanum | 109.6512 | 18.44421 |  |  |
| Antidesma montanum | 109.6639 | 18.45447 |  |  |
| Antidesma montanum | 109.5592 | 18.48573 |  |  |
| Antidesma montanum | 109.5533 | 18.47889 |  |  |
| Antidesma montanum | 109.5327 | 18.48963 |  |  |
| Antidesma montanum | 109.5774 | 18.51568 |  |  |
| Antidesma montanum | 109.532 | 18.53326 |  |  |
| Antidesma montanum | 109.5406 | 18.558 |  |  |
| Antidesma montanum | 109.5695 | 18.59347 |  |  |
| Antidesma montanum | 109.483 | 18.57004 |  |  |
| Antidesma montanum | 109.3381 | 18.44128 |  |  |
| Antidesma montanum | 109.3587 | 18.4315 |  |  |
| Antidesma montanum | 109.4054 | 18.52854 |  |  |
| Antidesma montanum | 109.5537 | 18.25537 |  |  |
| Antidesma montanum | 109.5283 | 18.26014 |  |  |
| Antidesma montanum | 109.5332 | 18.23756 |  |  |
| Antidesma montanum | 109.5276 | 18.2343 |  |  |
| Antidesma montanum | 109.5278 | 18.2312 |  |  |
| Antirhea chinensis | 109.3163 | 18.42284 | 5389.832 | 222245.7 |
| Antirhea chinensis | 109.6579 | 18.76335 | 42477.13 | 258885.1 |
| Antirhea chinensis | 109.7365 | 18.39782 | 49680.62 | 218236.5 |
| Antirhea chinensis | 110.1756 | 18.76435 | 97019.43 | 257602 |
| Antirhea chinensis | 110.2738 | 18.67292 | 107120.4 | 247246.1 |
| Antirhea chinensis | 110.5 | 19.3 |  |  |
| Antirhea chinensis | 109.7 | 18.6 |  |  |
| Antirhea chinensis | 109.2 | 18.7 |  |  |
| Antirhea chinensis | 109.5 | 18.3 |  |  |
| Antirhea chinensis | 110 | 18.5 |  |  |
| Antirhea chinensis | 109.6028 | 18.25985 |  |  |
| Antirhea chinensis | 109.599 | 18.27387 |  |  |
| Antirhea chinensis | 109.0737 | 19.00055 |  |  |
| Antirhea chinensis | 110.0378 | 18.4758 |  |  |
| Antirhea chinensis | 109.9742 | 18.49256 |  |  |
| Antirhea chinensis | 109.8136 | 18.58094 |  |  |
| Antirhea chinensis | 109.7875 | 18.60827 |  |  |
| Antirhea chinensis | 109.1022 | 19.35951 |  |  |
| Antirhea chinensis | 109.1304 | 19.30443 |  |  |
| Antirhea chinensis | 109.5636 | 19.29082 |  |  |
| Antirhea chinensis | 109.5753 | 19.32063 |  |  |
| Antirhea chinensis | 109.6199 | 19.26295 |  |  |
| Antirhea chinensis | 110.3588 | 19.15272 |  |  |
| Antirhea chinensis | 110.4034 | 19.16569 |  |  |
| Aphanamixis polystachya | 109.2292 | 18.32242 | -4135.79 | 211411.6 |
| Aphanamixis polystachya | 109.2343 | 18.80064 | -2022.54 | 264280.3 |
| Aphanamixis polystachya | 109.6828 | 18.3841 | 43968.29 | 216870.9 |
| Aphanamixis polystachya | 109.9706 | 19.1992 | 76627.91 | 306215.7 |
| Aphanamixis polystachya | 110.2356 | 18.69662 | 103162.5 | 249961.2 |
| Aphanamixis polystachya | 109.5 | 18.3 |  |  |
| Aphanamixis polystachya | 109.7 | 18.6 |  |  |
| Aphanamixis polystachya | 109.2 | 18.7 |  |  |
| Aphanamixis polystachya | 110.5 | 19.3 |  |  |
| Aphanamixis polystachya | 110 | 18.5 |  |  |
| Aphananthe cuspidata | 109.1191 | 19.05985 | -13271 | 293304.6 |
| Aphananthe cuspidata | 109.1199 | 18.9577 | -13534.4 | 282008.9 |
| Aphananthe cuspidata | 109.1741 | 18.37558 | -9781.56 | 217466.3 |
| Aphananthe cuspidata | 109.3011 | 18.85155 | 5180.119 | 269701.6 |
| Aphananthe cuspidata | 109.6365 | 19.44697 | 42283.97 | 334529.2 |
| Aphananthe cuspidata | 109.7575 | 19.47974 | 55069.1 | 337810.7 |
| Aphananthe cuspidata | 110.0417 | 18.51136 | 82213.82 | 229965.7 |
| Aphananthe cuspidata | 110.1571 | 18.66396 | 94801.89 | 246545.8 |
| Aphananthe cuspidata | 109.5 | 18.3 |  |  |
| Aphananthe cuspidata | 109.6361 | 18.26401 |  |  |
| Aphananthe cuspidata | 109.6471 | 18.2526 |  |  |
| Aphananthe cuspidata | 109.6618 | 18.24347 |  |  |
| Aphananthe cuspidata | 109.6797 | 18.24934 |  |  |
| Aphananthe cuspidata | 109.6021 | 18.27411 |  |  |
| Aphananthe cuspidata | 109.6048 | 18.25781 |  |  |
| Aphananthe cuspidata | 109.5911 | 18.24771 |  |  |
| Aphananthe cuspidata | 109.5966 | 18.22912 |  |  |
| Aphananthe cuspidata | 109.6093 | 18.22684 |  |  |
| Aphananthe cuspidata | 109.5863 | 18.23401 |  |  |
| Aphananthe cuspidata | 109.5901 | 18.22129 |  |  |
| Aphananthe cuspidata | 109.598 | 18.21249 |  |  |
| Aphananthe cuspidata | 109.6062 | 18.20303 |  |  |
| Aphananthe cuspidata | 109.6031 | 18.19357 |  |  |
| Aphananthe cuspidata | 109.612 | 18.18281 |  |  |
| Aphananthe cuspidata | 109.6193 | 18.17694 |  |  |
| Aphananthe cuspidata | 109.599 | 18.18216 |  |  |
| Aporosa villosa | 109.0947 | 18.75221 | -16882.4 | 259370.5 |
| Aporosa villosa | 109.5146 | 18.9427 | 27935.88 | 279133.3 |
| Aporosa villosa | 109.6807 | 18.37829 | 43729.57 | 216234.2 |
| Aporosa villosa | 109.837 | 18.73456 | 61260.82 | 255199 |
| Aporosa villosa | 110.1571 | 18.66396 | 94801.89 | 246545.8 |
| Aporosa villosa | 109.5348 | 18.41135 |  |  |
| Aporosa villosa | 109.5135 | 18.4133 |  |  |
| Aporosa villosa | 109.577 | 18.41689 |  |  |
| Aporosa villosa | 109.5815 | 18.44294 |  |  |
| Aporosa villosa | 109.6505 | 18.44457 |  |  |
| Aporosa villosa | 109.669 | 18.45011 |  |  |
| Aporosa villosa | 109.6663 | 18.39474 |  |  |
| Aporosa villosa | 109.6124 | 18.36737 |  |  |
| Aporosa villosa | 109.613 | 18.33739 |  |  |
| Aporosa villosa | 109.5008 | 109.5008 |  |  |
| Aporosa villosa | 109.4579 | 18.37519 |  |  |
| Aporosa villosa | 108.9082 | 18.56357 |  |  |
| Aporosa villosa | 108.911 | 18.65793 |  |  |
| Aporosa villosa | 108.9872 | 18.67029 |  |  |
| Aporosa villosa | 109.0428 | 18.52451 |  |  |
| Aporosa villosa | 109.1424 | 18.47046 |  |  |
| Aporosa villosa | 109.1245 | 18.39816 |  |  |
| Aporosa villosa | 109.2199 | 18.37991 |  |  |
| Aporosa villosa | 109.5413 | 18.2528 |  |  |
| Aporosa villosa | 109.5935 | 18.19997 |  |  |
| Aquilaria sinensis | 108.9122 | 18.99917 | -35232.9 | 287275.8 |
| Aquilaria sinensis | 109.0333 | 18.86036 | -22982.8 | 271527.7 |
| Aquilaria sinensis | 109.2292 | 18.32242 | -4135.79 | 211411.6 |
| Aquilaria sinensis | 109.6975 | 18.72833 | 46544.54 | 254900 |
| Aquilaria sinensis | 109.8844 | 18.79929 | 66438.38 | 262227.7 |
| Aquilaria sinensis | 110.1756 | 18.76435 | 97019.43 | 257602 |
| Aquilaria sinensis | 109.2 | 18.7 |  |  |
| Aquilaria sinensis | 110 | 18.5 |  |  |
| Aquilaria sinensis | 110.7 | 19.55572 |  |  |
| Aquilaria sinensis | 110.7326 | 19.51236 |  |  |
| Aquilaria sinensis | 108.9058 | 19.06209 |  |  |
| Aquilaria sinensis | 108.8838 | 19.10881 |  |  |
| Aquilaria sinensis | 108.9161 | 19.0569 |  |  |
| Aquilaria sinensis | 108.9717 | 18.73403 |  |  |
| Aquilaria sinensis | 109.0411 | 18.73207 |  |  |
| Aquilaria sinensis | 109.0177 | 18.66573 |  |  |
| Aquilaria sinensis | 109.015 | 18.682 |  |  |
| Aquilaria sinensis | 109.7923 | 18.60002 |  |  |
| Aquilaria sinensis | 109.8273 | 18.57398 |  |  |
| Aquilaria sinensis | 109.7792 | 18.52907 |  |  |
| Aquilaria sinensis | 109.8712 | 18.5167 |  |  |
| Aquilaria sinensis | 109.7737 | 18.52386 |  |  |
| Aquilaria sinensis | 109.5897 | 18.25476 |  |  |
| Aquilaria sinensis | 109.5753 | 18.2228 |  |  |
| Aquilaria sinensis | 109.876 | 18.5193 |  |  |
| Aralia chinensis | 109.0333 | 18.86036 | -22982.8 | 271527.7 |
| Aralia chinensis | 109.4548 | 19.18904 | 22421.09 | 306546.7 |
| Aralia chinensis | 109.837 | 18.73456 | 61260.82 | 255199 |
| Aralia chinensis | 110.001 | 18.75027 | 78585.68 | 256495.3 |
| Aralia chinensis | 110.1876 | 19.13499 | 99259.69 | 298557.2 |
| Aralia chinensis | 109.7 | 19.2 |  |  |
| Aralia chinensis | 109.1509 | 19.30852 |  |  |
| Aralia chinensis | 109.1702 | 19.32602 |  |  |
| Aralia chinensis | 109.1997 | 19.26575 |  |  |
| Aralia chinensis | 109.2375 | 19.2288 |  |  |
| Aralia chinensis | 109.2155 | 19.17173 |  |  |
| Aralia chinensis | 109.2745 | 19.17368 |  |  |
| Aralia chinensis | 109.3466 | 19.21064 |  |  |
| Aralia chinensis | 109.6508 | 19.1127 |  |  |
| Aralia chinensis | 109.6893 | 19.17627 |  |  |
| Aralia chinensis | 109.6982 | 19.15941 |  |  |
| Aralia chinensis | 109.7675 | 19.17562 |  |  |
| Aralia chinensis | 109.6426 | 19.11076 |  |  |
| Aralia chinensis | 109.6439 | 19.0543 |  |  |
| Aralia chinensis | 109.6797 | 18.89327 |  |  |
| Aralia chinensis | 109.3157 | 18.72167 |  |  |
| Aralia chinensis | 109.4352 | 18.6924 |  |  |
| Aralia chinensis | 109.5904 | 18.22671 |  |  |
| Aralia chinensis | 109.6391 | 18.25736 |  |  |
| Aralia finlaysoniana | 109.3011 | 18.85155 | 5180.119 | 269701.6 |
| Aralia finlaysoniana | 109.3011 | 18.85156 | 5181.175 | 269701.6 |
| Aralia finlaysoniana | 109.3011 | 18.85156 | 5182.231 | 269701.7 |
| Aralia finlaysoniana | 109.3011 | 18.85156 | 5183.287 | 269701.8 |
| Aralia finlaysoniana | 109.416 | 18.58215 | 16423.77 | 239559.1 |
| Aralia finlaysoniana | 109.3559 | 18.75418 |  |  |
| Aralia finlaysoniana | 109.2886 | 18.78994 |  |  |
| Aralia finlaysoniana | 109.4513 | 18.78149 |  |  |
| Aralia finlaysoniana | 109.4342 | 18.69045 |  |  |
| Aralia finlaysoniana | 109.3621 | 18.63451 |  |  |
| Aralia finlaysoniana | 109.5001 | 18.60002 |  |  |
| Aralia finlaysoniana | 109.52 | 18.53167 |  |  |
| Aralia finlaysoniana | 109.7665 | 18.60197 |  |  |
| Aralia finlaysoniana | 109.829 | 18.587 |  |  |
| Aralia finlaysoniana | 109.8304 | 18.54209 |  |  |
| Aralia finlaysoniana | 109.8695 | 18.51474 |  |  |
| Aralia finlaysoniana | 109.7748 | 18.5206 |  |  |
| Aralia finlaysoniana | 109.1439 | 19.29973 |  |  |
| Aralia finlaysoniana | 109.1006 | 19.35934 |  |  |
| Aralia finlaysoniana | 108.9255 | 19.34639 |  |  |
| Aralia finlaysoniana | 108.9063 | 19.25112 |  |  |
| Aralia finlaysoniana | 108.8342 | 19.33667 |  |  |
| Aralia finlaysoniana | 108.9029 | 19.40792 |  |  |
| Aralia finlaysoniana | 110.3055 | 18.8518 |  |  |
| Aralia finlaysoniana | 110.2204 | 18.91028 |  |  |
| Archiboehmeria atrata | 110.2238 | 18.67552 | 101866.1 | 247657.4 |
| Archiboehmeria atrata | 110.2356 | 18.69662 | 103162.5 | 249961.2 |
| Archiboehmeria atrata | 110.2356 | 18.69663 | 103162.7 | 249962.3 |
| Archiboehmeria atrata | 110.2356 | 18.69664 | 103162.8 | 249963.4 |
| Archiboehmeria atrata | 110.2356 | 18.69665 | 103162.9 | 249964.5 |
| Archiboehmeria atrata | 110.0212 | 18.68732 |  |  |
| Archiboehmeria atrata | 110.0247 | 18.66325 |  |  |
| Archiboehmeria atrata | 110.0123 | 18.61446 |  |  |
| Archiboehmeria atrata | 109.9402 | 18.58192 |  |  |
| Archiboehmeria atrata | 109.8777 | 18.51552 |  |  |
| Archiboehmeria atrata | 109.8757 | 18.47124 |  |  |
| Archiboehmeria atrata | 109.8365 | 18.40023 |  |  |
| Archiboehmeria atrata | 110.0453 | 18.39241 |  |  |
| Archiboehmeria atrata | 110.037 | 18.47189 |  |  |
| Archiboehmeria atrata | 110.081 | 18.50315 |  |  |
| Archiboehmeria atrata | 109.945 | 18.57541 |  |  |
| Archiboehmeria atrata | 110.0096 | 18.60795 |  |  |
| Archiboehmeria atrata | 110.1503 | 18.6073 |  |  |
| Archiboehmeria atrata | 109.7775 | 18.44714 |  |  |
| Archiboehmeria atrata | 109.9704 | 18.48459 |  |  |
| Archiboehmeria atrata | 109.9711 | 18.54481 |  |  |
| Archiboehmeria atrata | 110.1572 | 18.60535 |  |  |
| Archiboehmeria atrata | 110.1394 | 18.64374 |  |  |
| Archiboehmeria atrata | 110.0521 | 18.66521 |  |  |
| Archiboehmeria atrata | 110.0233 | 18.70683 |  |  |
| Archidendron lucidum | 109.2343 | 18.80064 | -2022.54 | 264280.3 |
| Archidendron lucidum | 109.7631 | 18.55976 |  |  |
| Archidendron lucidum | 109.7721 | 18.56298 |  |  |
| Archidendron lucidum | 109.8716 | 18.59842 |  |  |
| Archidendron lucidum | 109.8716 | 18.60051 |  |  |
| Archidendron lucidum | 109.8656 | 18.59921 |  |  |
| Archidendron lucidum | 109.817 | 18.57917 |  |  |
| Archidendron lucidum | 109.8251 | 18.57536 |  |  |
| Archidendron lucidum | 109.4713 | 19.21785 |  |  |
| Archidendron lucidum | 109.3858 | 19.23037 |  |  |
| Archidendron lucidum | 109.3541 | 19.21114 |  |  |
| Archidendron lucidum | 109.3478 | 19.21294 |  |  |
| Archidendron lucidum | 110.106 | 18.52879 |  |  |
| Archidendron lucidum | 110.0619 | 18.53691 |  |  |
| Archidendron lucidum | 110.0765 | 18.54269 |  |  |
| Archidendron lucidum | 109.6543 | 18.25237 |  |  |
| Archidendron lucidum | 109.6588 | 18.25665 |  |  |
| Archidendron lucidum | 109.5974 | 18.22513 |  |  |
| Archidendron lucidum | 109.598 | 18.22665 |  |  |
| Archidendron lucidum | 109.5973 | 18.25569 |  |  |
| Archidendron lucidum | 109.5953 | 18.25814 |  |  |
| Archidendron utile | 109.4273 | 19.01928 | 18993.09 | 287860.9 |
| Archidendron utile | 109.5146 | 18.9427 | 27935.88 | 279133.3 |
| Archidendron utile | 109.5146 | 18.9427 | 27935.99 | 279133.4 |
| Archidendron utile | 109.5146 | 18.9427 | 27936.1 | 279133.6 |
| Archidendron utile | 110.2356 | 18.69662 | 103162.5 | 249961.2 |
| Archidendron utile | 109.7707 | 18.57151 |  |  |
| Archidendron utile | 109.7722 | 18.56072 |  |  |
| Archidendron utile | 109.768 | 18.5623 |  |  |
| Archidendron utile | 109.7765 | 18.57444 |  |  |
| Archidendron utile | 109.7742 | 18.57381 |  |  |
| Archidendron utile | 109.7791 | 18.57368 |  |  |
| Archidendron utile | 109.7628 | 18.57332 |  |  |
| Archidendron utile | 109.7689 | 18.56131 |  |  |
| Archidendron utile | 109.7815 | 18.5664 |  |  |
| Archidendron utile | 109.7774 | 18.56384 |  |  |
| Archidendron utile | 109.8555 | 18.60094 |  |  |
| Archidendron utile | 109.8568 | 18.60641 |  |  |
| Archidendron utile | 109.8718 | 18.59747 |  |  |
| Archidendron utile | 109.8701 | 18.60551 |  |  |
| Archidendron utile | 109.8614 | 18.59993 |  |  |
| Archidendron utile | 109.8147 | 18.58617 |  |  |
| Archidendron utile | 109.8163 | 18.5752 |  |  |
| Archidendron utile | 109.8246 | 18.57372 |  |  |
| Archidendron utile | 109.8161 | 18.58623 |  |  |
| Archidendron utile | 109.8183 | 18.57973 |  |  |
| Ardisia crenata | 110.0102 | 19.56902 | 81818.27 | 346997.2 |
| Ardisia crenata | 110.4194 | 18.9871 | 123247.6 | 281640.2 |
| Ardisia crenata | 110.7217 | 19.71514 | 156705.5 | 361456.5 |
| Ardisia crenata | 110.425 | 18.8038 |  |  |
| Ardisia crenata | 110.4308 | 18.79969 |  |  |
| Ardisia crenata | 110.4314 | 18.81174 |  |  |
| Ardisia crenata | 110.4294 | 18.81054 |  |  |
| Ardisia crenata | 110.4292 | 18.79564 |  |  |
| Ardisia crenata | 110.439 | 18.80392 |  |  |
| Ardisia crenata | 110.3758 | 18.77075 |  |  |
| Ardisia crenata | 110.3721 | 18.77521 |  |  |
| Ardisia crenata | 110.3768 | 18.77694 |  |  |
| Ardisia crenata | 110.3807 | 18.77088 |  |  |
| Ardisia crenata | 110.3704 | 18.76917 |  |  |
| Ardisia crenata | 110.3779 | 18.76958 |  |  |
| Ardisia crenata | 110.3749 | 18.77288 |  |  |
| Ardisia crenata | 110.3731 | 18.77334 |  |  |
| Ardisia crenata | 110.377 | 18.77561 |  |  |
| Ardisia crenata | 110.4062 | 18.77422 |  |  |
| Ardisia crenata | 110.4164 | 18.7538 |  |  |
| Ardisia crenata | 110.411 | 18.74282 |  |  |
| Ardisia crenata | 110.4215 | 18.77277 |  |  |
| Ardisia crenata | 110.4326 | 18.77823 |  |  |
| Ardisia densilepidotula | 109.1741 | 18.37558 | -9781.56 | 217466.3 |
| Ardisia densilepidotula | 109.6579 | 18.76335 | 42477.13 | 258885.1 |
| Ardisia densilepidotula | 109.869 | 18.69141 | 64504.44 | 250339.5 |
| Ardisia densilepidotula | 110.0221 | 18.80753 | 80965.02 | 262772.1 |
| Ardisia densilepidotula | 110.1756 | 18.76435 | 97019.43 | 257602 |
| Ardisia densilepidotula | 109.1223 | 19.09069 |  |  |
| Ardisia densilepidotula | 109.1205 | 19.08996 |  |  |
| Ardisia densilepidotula | 109.1194 | 19.09085 |  |  |
| Ardisia densilepidotula | 109.1226 | 19.08727 |  |  |
| Ardisia densilepidotula | 109.1219 | 19.08557 |  |  |
| Ardisia densilepidotula | 109.8871 | 18.79393 |  |  |
| Ardisia densilepidotula | 109.8847 | 18.79673 |  |  |
| Ardisia densilepidotula | 109.8678 | 18.78554 |  |  |
| Ardisia densilepidotula | 109.8683 | 18.78479 |  |  |
| Ardisia densilepidotula | 109.8861 | 18.79912 |  |  |
| Ardisia densilepidotula | 109.2633 | 19.04066 |  |  |
| Ardisia densilepidotula | 109.2587 | 19.04129 |  |  |
| Ardisia densilepidotula | 109.2603 | 19.03605 |  |  |
| Ardisia densilepidotula | 109.2715 | 19.03308 |  |  |
| Ardisia densilepidotula | 109.2609 | 19.04346 |  |  |
| Ardisia densilepidotula | 109.865 | 19.09459 |  |  |
| Ardisia densilepidotula | 109.8631 | 19.09655 |  |  |
| Ardisia densilepidotula | 109.8695 | 19.09702 |  |  |
| Ardisia densilepidotula | 109.5441 | 19.0351 |  |  |
| Ardisia densilepidotula | 109.5478 | 19.03552 |  |  |
| Ardisia fordii | 110.9107 | 19.60103 | 176251 | 348446.2 |
| Ardisia fordii | 109.7634 | 18.57075 |  |  |
| Ardisia fordii | 109.7633 | 18.56274 |  |  |
| Ardisia fordii | 109.7635 | 18.56123 |  |  |
| Ardisia fordii | 109.7732 | 18.5696 |  |  |
| Ardisia fordii | 109.7735 | 18.56057 |  |  |
| Ardisia fordii | 109.7705 | 18.56245 |  |  |
| Ardisia fordii | 109.7641 | 18.55908 |  |  |
| Ardisia fordii | 109.8675 | 18.60495 |  |  |
| Ardisia fordii | 109.8676 | 18.60464 |  |  |
| Ardisia fordii | 109.8575 | 18.59687 |  |  |
| Ardisia fordii | 109.8588 | 18.60922 |  |  |
| Ardisia fordii | 109.8607 | 18.59548 |  |  |
| Ardisia fordii | 109.8517 | 18.60739 |  |  |
| Ardisia fordii | 109.8541 | 18.6081 |  |  |
| Ardisia fordii | 109.8666 | 18.60423 |  |  |
| Ardisia fordii | 109.8131 | 18.57692 |  |  |
| Ardisia fordii | 109.8283 | 18.5756 |  |  |
| Ardisia fordii | 109.8315 | 18.57701 |  |  |
| Ardisia fordii | 109.8151 | 18.57307 |  |  |
| Ardisia fordii | 109.8322 | 18.5741 |  |  |
| Ardisia gigantifolia | 108.9795 | 18.89322 | -28525.9 | 275338 |
| Ardisia gigantifolia | 109.4273 | 19.01928 | 18993.09 | 287860.9 |
| Ardisia gigantifolia | 109.8628 | 18.65961 | 63764.9 | 246839.6 |
| Ardisia gigantifolia | 110.0221 | 18.80753 | 80965.02 | 262772.1 |
| Ardisia gigantifolia | 110.1876 | 19.13499 | 99259.69 | 298557.2 |
| Ardisia gigantifolia | 109.7737 | 18.56637 |  |  |
| Ardisia gigantifolia | 109.7787 | 18.56699 |  |  |
| Ardisia gigantifolia | 109.7823 | 18.56943 |  |  |
| Ardisia gigantifolia | 109.772 | 18.5749 |  |  |
| Ardisia gigantifolia | 109.8492 | 18.60498 |  |  |
| Ardisia gigantifolia | 109.8661 | 18.60859 |  |  |
| Ardisia gigantifolia | 109.8689 | 18.59681 |  |  |
| Ardisia gigantifolia | 109.8573 | 18.60193 |  |  |
| Ardisia gigantifolia | 109.8192 | 18.57884 |  |  |
| Ardisia gigantifolia | 109.8273 | 18.5772 |  |  |
| Ardisia gigantifolia | 109.8191 | 18.57443 |  |  |
| Ardisia gigantifolia | 109.827 | 18.57753 |  |  |
| Ardisia gigantifolia | 109.283 | 18.78071 |  |  |
| Ardisia gigantifolia | 109.2946 | 18.78679 |  |  |
| Ardisia gigantifolia | 109.2954 | 18.79461 |  |  |
| Ardisia gigantifolia | 109.2918 | 18.78339 |  |  |
| Ardisia gigantifolia | 109.2856 | 18.79839 |  |  |
| Ardisia gigantifolia | 109.1405 | 18.57097 |  |  |
| Ardisia gigantifolia | 109.0431 | 18.53568 |  |  |
| Ardisia gigantifolia | 109.127 | 18.60671 |  |  |
| Ardisia humilis | 109.0333 | 18.86036 | -22982.8 | 271527.7 |
| Ardisia humilis | 109.5146 | 18.9427 | 27935.88 | 279133.3 |
| Ardisia humilis | 109.6828 | 18.3841 | 43968.29 | 216870.9 |
| Ardisia humilis | 109.869 | 18.69141 | 64504.44 | 250339.5 |
| Ardisia humilis | 110.1756 | 18.76435 | 97019.43 | 257602 |
| Ardisia humilis | 110.1924 | 18.76787 | 98795.13 | 257948.6 |
| Ardisia humilis | 110.5702 | 19.7207 | 140860.5 | 362403.6 |
| Ardisia humilis | 110.6034 | 19.41737 | 143614.6 | 328794.4 |
| Ardisia humilis | 110.2163 | 20.01166 |  |  |
| Ardisia humilis | 110.2158 | 20.01186 |  |  |
| Ardisia humilis | 110.4193 | 19.84669 |  |  |
| Ardisia humilis | 110.4173 | 19.85422 |  |  |
| Ardisia humilis | 110.417 | 19.8583 |  |  |
| Ardisia humilis | 110.4663 | 19.8465 |  |  |
| Ardisia humilis | 110.4648 | 19.85367 |  |  |
| Ardisia humilis | 110.4366 | 18.81296 |  |  |
| Ardisia humilis | 110.3795 | 18.77766 |  |  |
| Ardisia humilis | 110.4284 | 18.73415 |  |  |
| Ardisia humilis | 110.0756 | 18.51549 |  |  |
| Ardisia humilis | 110.0651 | 18.51621 |  |  |
| Ardisia humilis | 110.0772 | 18.55833 |  |  |
| Ardisia humilis | 109.769 | 18.55898 |  |  |
| Ardisia humilis | 109.8204 | 18.57473 |  |  |
| Ardisia humilis | 109.8678 | 18.60908 |  |  |
| Ardisia humilis | 109.6543 | 18.25728 |  |  |
| Ardisia humilis | 109.5928 | 18.26019 |  |  |
| Ardisia humilis | 109.5965 | 18.22429 |  |  |
| Ardisia humilis | 109.5919 | 18.22356 |  |  |
| Ardisia lindleyana | 109.0589 | 19.27349 | -18875.4 | 317117.3 |
| Ardisia lindleyana | 109.4655 | 19.21722 |  |  |
| Ardisia lindleyana | 109.4665 | 19.21663 |  |  |
| Ardisia lindleyana | 109.3467 | 19.21215 |  |  |
| Ardisia lindleyana | 109.3465 | 19.21464 |  |  |
| Ardisia lindleyana | 109.395 | 19.2322 |  |  |
| Ardisia lindleyana | 109.3946 | 19.23355 |  |  |
| Ardisia lindleyana | 109.0381 | 19.30171 |  |  |
| Ardisia lindleyana | 109.0282 | 19.30575 |  |  |
| Ardisia lindleyana | 109.0284 | 19.28158 |  |  |
| Ardisia lindleyana | 109.0712 | 19.36166 |  |  |
| Ardisia lindleyana | 109.8041 | 19.05368 |  |  |
| Ardisia lindleyana | 109.7139 | 18.96868 |  |  |
| Ardisia lindleyana | 109.8119 | 19.03253 |  |  |
| Ardisia lindleyana | 109.7804 | 18.56332 |  |  |
| Ardisia lindleyana | 109.7804 | 18.56333 |  |  |
| Ardisia lindleyana | 109.8701 | 18.60531 |  |  |
| Ardisia lindleyana | 109.8565 | 18.60342 |  |  |
| Ardisia lindleyana | 109.8179 | 18.58092 |  |  |
| Ardisia lindleyana | 109.8128 | 18.57308 |  |  |
| Ardisia lindleyana | 109.8334 | 18.57648 |  |  |
| Ardisia maclurei | 108.9795 | 18.89322 | -28525.9 | 275338 |
| Ardisia maclurei | 109.2343 | 18.80064 | -2022.54 | 264280.3 |
| Ardisia maclurei | 109.416 | 18.58215 | 16423.77 | 239559.1 |
| Ardisia maclurei | 110.2542 | 18.78692 | 105355.2 | 259902.1 |
| Ardisia maclurei | 110.2671 | 19.67621 |  |  |
| Ardisia maclurei | 110.2382 | 19.64951 |  |  |
| Ardisia maclurei | 110.2703 | 19.56805 |  |  |
| Ardisia maclurei | 110.2298 | 19.64239 |  |  |
| Ardisia maclurei | 109.7226 | 19.07498 |  |  |
| Ardisia maclurei | 109.8762 | 19.07149 |  |  |
| Ardisia maclurei | 109.7201 | 18.9953 |  |  |
| Ardisia maclurei | 109.7415 | 19.06497 |  |  |
| Ardisia maclurei | 109.7733 | 18.56486 |  |  |
| Ardisia maclurei | 109.7696 | 18.56263 |  |  |
| Ardisia maclurei | 109.8503 | 18.60534 |  |  |
| Ardisia maclurei | 109.8548 | 18.59699 |  |  |
| Ardisia maclurei | 109.821 | 18.57293 |  |  |
| Ardisia maclurei | 109.8225 | 18.58327 |  |  |
| Ardisia maclurei | 109.0289 | 18.71827 |  |  |
| Ardisia maclurei | 109.077 | 18.68228 |  |  |
| Ardisia maclurei | 109.1364 | 18.57119 |  |  |
| Ardisia maclurei | 109.0851 | 18.56557 |  |  |
| Ardisia maclurei | 109.2739 | 18.79012 |  |  |
| Ardisia maclurei | 109.2956 | 18.78314 |  |  |
| Ardisia mamillata | 109.0333 | 18.86036 | -22982.8 | 271527.7 |
| Ardisia mamillata | 109.1191 | 19.05985 | -13271 | 293304.6 |
| Ardisia mamillata | 109.5146 | 18.9427 | 27935.88 | 279133.3 |
| Ardisia mamillata | 109.8188 | 19.14345 | 60528.46 | 300462.2 |
| Ardisia mamillata | 110.0792 | 18.68525 | 86649.62 | 249100.3 |
| Ardisia mamillata | 109.8153 | 19.10458 |  |  |
| Ardisia mamillata | 109.8887 | 19.07671 |  |  |
| Ardisia mamillata | 109.768 | 19.06752 |  |  |
| Ardisia mamillata | 108.741 | 19.04689 |  |  |
| Ardisia mamillata | 108.7349 | 19.04279 |  |  |
| Ardisia mamillata | 108.7793 | 18.9804 |  |  |
| Ardisia mamillata | 108.7935 | 18.96226 |  |  |
| Ardisia mamillata | 108.7947 | 18.95857 |  |  |
| Ardisia mamillata | 108.7851 | 18.95629 |  |  |
| Ardisia mamillata | 108.7455 | 19.0647 |  |  |
| Ardisia mamillata | 108.7432 | 19.06363 |  |  |
| Ardisia mamillata | 108.7443 | 19.06514 |  |  |
| Ardisia mamillata | 108.7447 | 19.06404 |  |  |
| Ardisia mamillata | 109.0758 | 18.70577 |  |  |
| Ardisia mamillata | 109.0362 | 18.73935 |  |  |
| Ardisia mamillata | 109.0419 | 18.56423 |  |  |
| Ardisia mamillata | 109.1037 | 18.58932 |  |  |
| Ardisia mamillata | 109.1204 | 18.53951 |  |  |
| Ardisia mamillata | 109.2871 | 18.77751 |  |  |
| Ardisia mamillata | 109.2915 | 18.79168 |  |  |
| Ardisia obtusa | 109.4044 | 18.88006 | 16149.68 | 272536.9 |
| Ardisia obtusa | 109.416 | 18.58215 | 16423.77 | 239559.1 |
| Ardisia obtusa | 109.5146 | 18.9427 | 27935.88 | 279133.3 |
| Ardisia obtusa | 109.6828 | 18.3841 | 43968.29 | 216870.9 |
| Ardisia obtusa | 109.9553 | 19.3256 | 75380.26 | 320231.5 |
| Ardisia obtusa | 109.9578 | 18.76128 | 74063.58 | 257827 |
| Ardisia obtusa | 110.0221 | 18.80753 | 80965.02 | 262772.1 |
| Ardisia obtusa | 110.0502 | 19.8863 | 86871.4 | 381966.6 |
| Ardisia obtusa | 110.2738 | 18.67292 | 107120.4 | 247246.1 |
| Ardisia obtusa | 110.3254 | 20.03167 | 116024 | 397349.5 |
| Ardisia obtusa | 110.2161 | 20.01144 |  |  |
| Ardisia obtusa | 110.2162 | 20.01163 |  |  |
| Ardisia obtusa | 110.415 | 19.85961 |  |  |
| Ardisia obtusa | 110.4192 | 19.8569 |  |  |
| Ardisia obtusa | 110.4605 | 19.85504 |  |  |
| Ardisia obtusa | 110.4568 | 19.85501 |  |  |
| Ardisia obtusa | 110.4335 | 18.80309 |  |  |
| Ardisia obtusa | 110.4231 | 18.79339 |  |  |
| Ardisia obtusa | 110.4104 | 18.78351 |  |  |
| Ardisia obtusa | 110.4087 | 18.74355 |  |  |
| Ardisia obtusa | 110.3803 | 18.76908 |  |  |
| Ardisia obtusa | 109.7196 | 19.0258 |  |  |
| Ardisia obtusa | 109.7316 | 18.97436 |  |  |
| Ardisia obtusa | 109.7661 | 18.57038 |  |  |
| Ardisia obtusa | 109.8304 | 18.57524 |  |  |
| Ardisia obtusa | 109.871 | 18.60584 |  |  |
| Ardisia obtusa | 109.8567 | 18.59827 |  |  |
| Ardisia obtusa | 109.6576 | 18.25026 |  |  |
| Ardisia obtusa | 109.5909 | 18.25923 |  |  |
| Ardisia obtusa | 109.5923 | 18.22299 |  |  |
| Ardisia obtusa | 109.5997 | 18.22229 |  |  |
| Ardisia ordinata | 108.9795 | 18.89322 | -28525.9 | 275338 |
| Ardisia ordinata | 109.1191 | 19.05985 | -13271 | 293304.6 |
| Ardisia ordinata | 109.3971 | 19.25638 | 16580.31 | 314164.5 |
| Ardisia ordinata | 109.5146 | 18.9427 | 27935.88 | 279133.3 |
| Ardisia ordinata | 109.1211 | 19.08823 |  |  |
| Ardisia ordinata | 109.1205 | 19.08735 |  |  |
| Ardisia ordinata | 109.1183 | 19.08964 |  |  |
| Ardisia ordinata | 109.1227 | 19.09021 |  |  |
| Ardisia ordinata | 109.8658 | 19.09563 |  |  |
| Ardisia ordinata | 109.8691 | 19.09612 |  |  |
| Ardisia ordinata | 109.8648 | 19.09573 |  |  |
| Ardisia ordinata | 109.8658 | 19.0968 |  |  |
| Ardisia ordinata | 109.8691 | 19.09375 |  |  |
| Ardisia ordinata | 109.5426 | 19.0344 |  |  |
| Ardisia ordinata | 109.5454 | 19.03339 |  |  |
| Ardisia ordinata | 109.5436 | 19.03774 |  |  |
| Ardisia ordinata | 109.5441 | 19.03104 |  |  |
| Ardisia ordinata | 109.5464 | 19.03717 |  |  |
| Ardisia ordinata | 109.2714 | 19.03793 |  |  |
| Ardisia ordinata | 109.2716 | 19.04449 |  |  |
| Ardisia ordinata | 109.2565 | 19.03955 |  |  |
| Ardisia ordinata | 109.2588 | 19.03639 |  |  |
| Ardisia ordinata | 109.2714 | 19.03804 |  |  |
| Ardisia ordinata | 109.2679 | 19.04131 |  |  |
| Ardisia quinquegona Blume | 109.0333 | 18.86036 | -22982.8 | 271527.7 |
| Ardisia quinquegona Blume | 109.3011 | 18.85155 | 5180.119 | 269701.6 |
| Ardisia quinquegona Blume | 109.5146 | 18.9427 | 27935.88 | 279133.3 |
| Ardisia quinquegona Blume | 109.837 | 18.73456 | 61260.82 | 255199 |
| Ardisia quinquegona Blume | 110.2253 | 18.686 | 102042.4 | 248812.9 |
| Ardisia quinquegona Blume | 110.004 | 18.77932 |  |  |
| Ardisia quinquegona Blume | 109.0556 | 18.67314 |  |  |
| Ardisia quinquegona Blume | 109.7771 | 18.83341 |  |  |
| Ardisia quinquegona Blume | 110.0382 | 18.931 |  |  |
| Ardisia quinquegona Blume | 109.5259 | 19.2391 |  |  |
| Ardisia quinquegona Blume | 109.2559 | 19.06238 |  |  |
| Ardisia quinquegona Blume | 109.357 | 18.92938 |  |  |
| Ardisia quinquegona Blume | 109.3179 | 18.77292 |  |  |
| Ardisia quinquegona Blume | 109.2741 | 18.84723 |  |  |
| Ardisia quinquegona Blume | 108.9842 | 19.12056 |  |  |
| Ardisia quinquegona Blume | 109.1245 | 18.80866 |  |  |
| Ardisia quinquegona Blume | 109.931 | 19.64304 |  |  |
| Ardisia quinquegona Blume | 110.432 | 19.37796 |  |  |
| Ardisia quinquegona Blume | 110.1377 | 19.57078 |  |  |
| Ardisia quinquegona Blume | 110.4675 | 19.40925 |  |  |
| Ardisia quinquegona Blume | 110.1478 | 19.46112 |  |  |
| Ardisia quinquegona Blume | 109.8739 | 19.46614 |  |  |
| Ardisia quinquegona Blume | 110.4004 | 19.35655 |  |  |
| Ardisia quinquegona Blume | 110.4781 | 19.3088 |  |  |
| Ardisia quinquegona Blume | 110.2545 | 19.78806 |  |  |
| Ardisia villosa | 108.9795 | 18.89322 | -28525.9 | 275338 |
| Ardisia villosa | 109.0333 | 18.86036 | -22982.8 | 271527.7 |
| Ardisia villosa | 109.1563 | 19.06338 | -9349.4 | 293575.8 |
| Ardisia villosa | 109.4273 | 19.01928 | 18993.09 | 287860.9 |
| Ardisia villosa | 110.2549 | 18.71143 | 105232.9 | 251551.4 |
| Ardisia villosa | 109.811 | 19.03408 |  |  |
| Ardisia villosa | 109.7694 | 18.95267 |  |  |
| Ardisia villosa | 109.855 | 18.9875 |  |  |
| Ardisia villosa | 109.4669 | 19.22027 |  |  |
| Ardisia villosa | 109.4708 | 19.21892 |  |  |
| Ardisia villosa | 109.3557 | 19.21968 |  |  |
| Ardisia villosa | 109.3529 | 19.21603 |  |  |
| Ardisia villosa | 109.349 | 19.21553 |  |  |
| Ardisia villosa | 109.3861 | 19.23279 |  |  |
| Ardisia villosa | 109.3899 | 19.23549 |  |  |
| Ardisia villosa | 109.072 | 18.74273 |  |  |
| Ardisia villosa | 109.0289 | 18.75465 |  |  |
| Ardisia villosa | 109.1506 | 18.56101 |  |  |
| Ardisia villosa | 109.0683 | 18.57703 |  |  |
| Ardisia villosa | 109.2778 | 18.79594 |  |  |
| Ardisia villosa | 109.2966 | 18.78591 |  |  |
| Ardisia villosa | 109.7823 | 18.5665 |  |  |
| Ardisia villosa | 109.8229 | 18.5813 |  |  |
| Ardisia villosa | 109.8571 | 18.60685 |  |  |
| Ardisia villosa | 109.8635 | 18.60574 |  |  |
| Arenga caudata | 109.7034 | 18.6413 | 46906.29 | 245259 |
| Arenga caudata | 110.1756 | 18.76435 | 97019.43 | 257602 |
| Arenga caudata | 110.1756 | 18.76436 | 97019.55 | 257602.5 |
| Arenga caudata | 110.4194 | 18.9871 | 123247.6 | 281640.2 |
| Arenga caudata | 110.4321 | 19.37563 | 125548.1 | 324570.5 |
| Arenga caudata | 109.7694 | 18.5635 |  |  |
| Arenga caudata | 109.7824 | 18.56051 |  |  |
| Arenga caudata | 109.86 | 18.59946 |  |  |
| Arenga caudata | 109.8529 | 18.60723 |  |  |
| Arenga caudata | 109.8627 | 18.60387 |  |  |
| Arenga caudata | 109.8492 | 18.60566 |  |  |
| Arenga caudata | 109.8343 | 18.58243 |  |  |
| Arenga caudata | 109.8295 | 18.57785 |  |  |
| Arenga caudata | 109.8171 | 18.58684 |  |  |
| Arenga caudata | 109.8332 | 18.58378 |  |  |
| Arenga caudata | 110.4256 | 18.79566 |  |  |
| Arenga caudata | 110.4277 | 18.79957 |  |  |
| Arenga caudata | 110.4394 | 18.81127 |  |  |
| Arenga caudata | 110.437 | 18.80626 |  |  |
| Arenga caudata | 110.422 | 18.74027 |  |  |
| Arenga caudata | 110.3992 | 18.75754 |  |  |
| Arenga caudata | 110.4077 | 18.73205 |  |  |
| Arenga caudata | 110.3707 | 18.76931 |  |  |
| Arenga caudata | 110.3774 | 18.77228 |  |  |
| Arenga caudata | 110.3779 | 18.77161 |  |  |
| Arenga pinnata | 109.3163 | 18.42284 | 5389.832 | 222245.7 |
| Arenga pinnata | 109.777 | 19.17887 | 56233.56 | 304494 |
| Arenga pinnata | 109.837 | 18.73456 | 61260.82 | 255199 |
| Arenga pinnata | 110.0914 | 18.76565 | 88154.38 | 257960.3 |
| Arenga pinnata | 110.2238 | 18.67552 | 101866.1 | 247657.4 |
| Arenga pinnata | 108.7404 | 19.04234 |  |  |
| Arenga pinnata | 108.7464 | 19.0642 |  |  |
| Arenga pinnata | 108.7436 | 19.06684 |  |  |
| Arenga pinnata | 108.7952 | 18.96897 |  |  |
| Arenga pinnata | 109.6551 | 18.25653 |  |  |
| Arenga pinnata | 109.5923 | 18.259 |  |  |
| Arenga pinnata | 109.5946 | 18.22177 |  |  |
| Arenga pinnata | 109.5913 | 18.22355 |  |  |
| Arenga pinnata | 109.0085 | 19.31102 |  |  |
| Arenga pinnata | 109.1641 | 19.32037 |  |  |
| Arenga pinnata | 109.1075 | 19.36257 |  |  |
| Arenga pinnata | 109.1308 | 19.30383 |  |  |
| Arenga pinnata | 109.778 | 18.57251 |  |  |
| Arenga pinnata | 109.8142 | 18.57393 |  |  |
| Arenga pinnata | 109.8652 | 18.60156 |  |  |
| Arenga pinnata | 109.8714 | 18.59504 |  |  |
| Arenga pinnata | 109.8509 | 18.60267 |  |  |
| Arenga pinnata | 110.3643 | 19.27849 |  |  |
| Arenga pinnata | 110.3788 | 19.32749 |  |  |
| Arenga pinnata | 110.5811 | 19.2499 |  |  |
| Artabotrys hexapetalus | 108.9122 | 18.99917 | -35232.9 | 287275.8 |
| Artabotrys hexapetalus | 108.9515 | 18.90932 | -31419.3 | 277210.7 |
| Artabotrys hexapetalus | 109.0799 | 19.00987 | -17563.9 | 287906.3 |
| Artabotrys hexapetalus | 109.1191 | 19.05985 | -13271 | 293304.6 |
| Artabotrys hexapetalus | 109.6807 | 18.37829 | 43729.57 | 216234.2 |
| Artabotrys hexapetalus | 109.6518 | 18.24889 |  |  |
| Artabotrys hexapetalus | 109.6534 | 18.25531 |  |  |
| Artabotrys hexapetalus | 109.6513 | 18.25389 |  |  |
| Artabotrys hexapetalus | 109.6489 | 18.25554 |  |  |
| Artabotrys hexapetalus | 109.596 | 18.22138 |  |  |
| Artabotrys hexapetalus | 109.5964 | 18.22302 |  |  |
| Artabotrys hexapetalus | 109.5988 | 18.22605 |  |  |
| Artabotrys hexapetalus | 109.5951 | 18.26236 |  |  |
| Artabotrys hexapetalus | 109.6002 | 18.25858 |  |  |
| Artabotrys hexapetalus | 109.5963 | 18.25764 |  |  |
| Artabotrys hexapetalus | 109.5941 | 18.25914 |  |  |
| Artabotrys hexapetalus | 109.7707 | 18.57308 |  |  |
| Artabotrys hexapetalus | 109.7729 | 18.57415 |  |  |
| Artabotrys hexapetalus | 109.7841 | 18.56608 |  |  |
| Artabotrys hexapetalus | 109.8653 | 18.59502 |  |  |
| Artabotrys hexapetalus | 109.8559 | 18.60009 |  |  |
| Artabotrys hexapetalus | 109.8632 | 18.59567 |  |  |
| Artabotrys hexapetalus | 109.8322 | 18.58235 |  |  |
| Artabotrys hexapetalus | 109.8189 | 18.57856 |  |  |
| Artabotrys hexapetalus | 109.8191 | 18.58621 |  |  |
| Artabotrys hongkongensis | 108.9795 | 18.89322 | -28525.9 | 275338 |
| Artabotrys hongkongensis | 109.5425 | 19.03583 | 31162.38 | 289348.8 |
| Artabotrys hongkongensis | 109.6807 | 18.37829 | 43729.57 | 216234.2 |
| Artabotrys hongkongensis | 110.2332 | 18.6893 | 102892.4 | 249157.1 |
| Artabotrys hongkongensis | 110.4194 | 18.9871 | 123247.6 | 281640.2 |
| Artabotrys hongkongensis | 109.6517 | 18.25048 |  |  |
| Artabotrys hongkongensis | 109.6582 | 18.25678 |  |  |
| Artabotrys hongkongensis | 109.6549 | 18.25135 |  |  |
| Artabotrys hongkongensis | 109.6588 | 18.25545 |  |  |
| Artabotrys hongkongensis | 109.5957 | 18.2246 |  |  |
| Artabotrys hongkongensis | 109.6 | 18.22295 |  |  |
| Artabotrys hongkongensis | 109.5968 | 18.22685 |  |  |
| Artabotrys hongkongensis | 109.5977 | 18.22432 |  |  |
| Artabotrys hongkongensis | 109.5994 | 18.26071 |  |  |
| Artabotrys hongkongensis | 109.5968 | 18.25874 |  |  |
| Artabotrys hongkongensis | 109.5967 | 18.26015 |  |  |
| Artabotrys hongkongensis | 109.5942 | 18.26173 |  |  |
| Artabotrys hongkongensis | 109.075 | 18.73646 |  |  |
| Artabotrys hongkongensis | 109.0146 | 18.70337 |  |  |
| Artabotrys hongkongensis | 109.1395 | 18.60378 |  |  |
| Artabotrys hongkongensis | 109.1356 | 18.61184 |  |  |
| Artabotrys hongkongensis | 109.0863 | 18.57803 |  |  |
| Artabotrys hongkongensis | 109.2957 | 18.79808 |  |  |
| Artabotrys hongkongensis | 109.278 | 18.8035 |  |  |
| Artabotrys hongkongensis | 109.28 | 18.77965 |  |  |
| Artocarpus nitidus | 108.9315 | 18.7598 | -34046.8 | 260746.5 |
| Artocarpus nitidus | 109.0333 | 18.86036 | -22982.8 | 271527.7 |
| Artocarpus nitidus | 109.1041 | 19.50575 | -13336.8 | 342643.7 |
| Artocarpus nitidus | 109.3011 | 18.85155 | 5180.119 | 269701.6 |
| Artocarpus nitidus | 109.777 | 19.17887 | 56233.56 | 304494 |
| Artocarpus nitidus | 110.2238 | 18.67552 | 101866.1 | 247657.4 |
| Artocarpus nitidus | 110.2356 | 18.69662 | 103162.5 | 249961.2 |
| Artocarpus nitidus | 108.9716 | 18.96729 |  |  |
| Artocarpus nitidus | 109.1571 | 18.87081 |  |  |
| Artocarpus nitidus | 110.0483 | 18.98539 |  |  |
| Artocarpus nitidus | 109.548 | 19.06907 |  |  |
| Artocarpus nitidus | 109.2839 | 18.90087 |  |  |
| Artocarpus nitidus | 109.0394 | 18.68181 |  |  |
| Artocarpus nitidus | 109.8217 | 18.67649 |  |  |
| Artocarpus nitidus | 109.2247 | 18.69151 |  |  |
| Artocarpus nitidus | 109.1003 | 19.01656 |  |  |
| Artocarpus nitidus | 109.7344 | 19.23954 |  |  |
| Artocarpus nitidus | 109.7923 | 19.4991 |  |  |
| Artocarpus nitidus | 110.2069 | 19.51602 |  |  |
| Artocarpus nitidus | 109.8451 | 19.21028 |  |  |
| Artocarpus nitidus | 110.1317 | 19.74526 |  |  |
| Artocarpus nitidus | 110.1648 | 19.57473 |  |  |
| Artocarpus nitidus | 109.9299 | 19.7538 |  |  |
| Artocarpus nitidus | 110.2371 | 19.54512 |  |  |
| Artocarpus nitidus | 110.5047 | 19.50142 |  |  |
| Artocarpus nitidus | 110.1342 | 19.58212 |  |  |
| Artocarpus nitidus | 110.3884 | 19.19008 |  |  |
| Artocarpus styracifolius | 108.6526 | 18.84592 | -63101 | 271225.5 |
| Artocarpus styracifolius | 109.0333 | 18.86036 | -22982.8 | 271527.7 |
| Artocarpus styracifolius | 109.1215 | 19.14164 | -12747.8 | 302339.7 |
| Artocarpus styracifolius | 109.1563 | 19.06338 | -9349.4 | 293575.8 |
| Artocarpus styracifolius | 109.3011 | 18.85155 | 5180.119 | 269701.6 |
| Artocarpus styracifolius | 109.6579 | 18.76335 | 42477.13 | 258885.1 |
| Artocarpus styracifolius | 109.6835 | 18.87437 | 45503.17 | 271088.8 |
| Artocarpus styracifolius | 109.837 | 18.73456 | 61260.82 | 255199 |
| Artocarpus styracifolius | 109.8844 | 18.79929 | 66438.38 | 262227.7 |
| Artocarpus styracifolius | 110.0914 | 18.76565 | 88154.38 | 257960.3 |
| Artocarpus styracifolius | 108.7457 | 19.04891 |  |  |
| Artocarpus styracifolius | 108.7463 | 19.05014 |  |  |
| Artocarpus styracifolius | 108.7807 | 18.97456 |  |  |
| Artocarpus styracifolius | 108.8006 | 18.9845 |  |  |
| Artocarpus styracifolius | 108.8005 | 18.95059 |  |  |
| Artocarpus styracifolius | 108.7431 | 19.06433 |  |  |
| Artocarpus styracifolius | 108.7451 | 19.06591 |  |  |
| Artocarpus styracifolius | 109.0334 | 18.7207 |  |  |
| Artocarpus styracifolius | 109.284 | 18.77734 |  |  |
| Artocarpus styracifolius | 109.0532 | 18.52963 |  |  |
| Artocarpus styracifolius | 109.0959 | 18.60936 |  |  |
| Artocarpus styracifolius | 109.776 | 18.56428 |  |  |
| Artocarpus styracifolius | 109.7688 | 18.57473 |  |  |
| Artocarpus styracifolius | 109.86 | 18.60397 |  |  |
| Artocarpus styracifolius | 109.8717 | 18.60065 |  |  |
| Artocarpus styracifolius | 109.826 | 18.57323 |  |  |
| Artocarpus styracifolius | 109.8149 | 18.58536 |  |  |
| Artocarpus styracifolius | 109.6585 | 18.2558 |  |  |
| Artocarpus styracifolius | 109.5947 | 18.25962 |  |  |
| Artocarpus styracifolius | 109.5988 | 18.22507 |  |  |
| Artocarpus tonkinensis | 108.6552 | 18.85361 | -62804.5 | 272066 |
| Artocarpus tonkinensis | 109.3011 | 18.85155 | 5180.119 | 269701.6 |
| Artocarpus tonkinensis | 109.7575 | 19.47974 | 55069.1 | 337810.7 |
| Artocarpus tonkinensis | 110.0102 | 19.56902 | 81818.27 | 346997.2 |
| Artocarpus tonkinensis | 110.0284 | 18.90832 | 81905.25 | 273901 |
| Artocarpus tonkinensis | 109.9217 | 19.0002 |  |  |
| Artocarpus tonkinensis | 109.8867 | 19.0229 |  |  |
| Artocarpus tonkinensis | 109.9011 | 18.94926 |  |  |
| Artocarpus tonkinensis | 109.764 | 18.94252 |  |  |
| Artocarpus tonkinensis | 110.0954 | 18.5293 |  |  |
| Artocarpus tonkinensis | 110.0994 | 18.54596 |  |  |
| Artocarpus tonkinensis | 110.0631 | 18.55286 |  |  |
| Artocarpus tonkinensis | 109.765 | 18.56222 |  |  |
| Artocarpus tonkinensis | 109.834 | 18.58056 |  |  |
| Artocarpus tonkinensis | 109.8527 | 18.60394 |  |  |
| Artocarpus tonkinensis | 109.6575 | 18.25249 |  |  |
| Artocarpus tonkinensis | 109.5916 | 18.2584 |  |  |
| Artocarpus tonkinensis | 109.5906 | 18.22646 |  |  |
| Artocarpus tonkinensis | 109.5923 | 18.22239 |  |  |
| Artocarpus tonkinensis | 109.5934 | 18.22663 |  |  |
| Artocarpus tonkinensis | 109.7191 | 19.46915 |  |  |
| Artocarpus tonkinensis | 109.6642 | 19.52889 |  |  |
| Artocarpus tonkinensis | 109.7364 | 19.91052 |  |  |
| Artocarpus tonkinensis | 109.6346 | 19.89226 |  |  |
| Artocarpus tonkinensis | 110.0141 | 19.75932 |  |  |
| Arytera littoralis | 109.0212 | 19.35345 | -22551.4 | 326078.8 |
| Arytera littoralis | 109.0333 | 18.86036 | -22982.8 | 271527.7 |
| Arytera littoralis | 109.2343 | 18.80064 | -2022.54 | 264280.3 |
| Arytera littoralis | 109.3011 | 18.85155 | 5180.119 | 269701.6 |
| Arytera littoralis | 109.3116 | 18.96725 | 6666.857 | 282460.4 |
| Arytera littoralis | 109.3449 | 19.70818 | 12549.17 | 364265 |
| Arytera littoralis | 109.416 | 18.58215 | 16423.77 | 239559.1 |
| Arytera littoralis | 109.429 | 18.50349 | 17547.73 | 230821.1 |
| Arytera littoralis | 109.4865 | 19.15807 | 25651.28 | 303028.8 |
| Arytera littoralis | 109.6828 | 18.3841 | 43968.29 | 216870.9 |
| Arytera littoralis | 109.6964 | 19.03697 | 47343.87 | 289030.5 |
| Arytera littoralis | 109.7097 | 19.53075 | 50211.1 | 343582.5 |
| Arytera littoralis | 109.7426 | 19.4888 | 53537.07 | 338853.4 |
| Arytera littoralis | 109.9249 | 18.56104 | 70034.61 | 235770.5 |
| Arytera littoralis | 110.1167 | 19.3938 | 92504.95 | 327351.7 |
| Arytera littoralis | 110.1756 | 18.76435 | 97019.43 | 257602 |
| Arytera littoralis | 110.2549 | 18.71143 | 105232.9 | 251551.4 |
| Arytera littoralis | 110.4194 | 18.9871 | 123247.6 | 281640.2 |
| Arytera littoralis | 110.4321 | 19.37563 | 125548.1 | 324570.5 |
| Arytera littoralis | 110.6819 | 19.92734 | 153022.5 | 385001.2 |
| Arytera littoralis | 109.9452 | 19.77624 |  |  |
| Arytera littoralis | 110.0184 | 19.73504 |  |  |
| Arytera littoralis | 109.665 | 19.95004 |  |  |
| Arytera littoralis | 109.6825 | 19.96131 |  |  |
| Arytera littoralis | 109.6271 | 19.86695 |  |  |
| Arytera littoralis | 109.6329 | 19.86664 |  |  |
| Arytera littoralis | 109.7654 | 19.90707 |  |  |
| Arytera littoralis | 109.7499 | 19.89662 |  |  |
| Arytera littoralis | 109.7007 | 19.55826 |  |  |
| Arytera littoralis | 109.7071 | 19.44843 |  |  |
| Arytera littoralis | 109.6949 | 19.46926 |  |  |
| Arytera littoralis | 109.6472 | 19.45344 |  |  |
| Arytera littoralis | 109.5872 | 19.57169 |  |  |
| Arytera littoralis | 109.7085 | 19.48706 |  |  |
| Arytera littoralis | 108.9874 | 19.30813 |  |  |
| Arytera littoralis | 109.151 | 19.34791 |  |  |
| Arytera littoralis | 108.7322 | 19.04774 |  |  |
| Arytera littoralis | 108.7442 | 19.06421 |  |  |
| Arytera littoralis | 108.8042 | 18.97091 |  |  |
| Arytera littoralis | 109.654 | 18.25574 |  |  |
| Atalantia buxifolia | 109.1741 | 18.37558 | -9781.56 | 217466.3 |
| Atalantia buxifolia | 109.4044 | 18.88006 | 16149.68 | 272536.9 |
| Atalantia buxifolia | 109.6964 | 19.03697 | 47343.87 | 289030.5 |
| Atalantia buxifolia | 110.0914 | 18.76565 | 88154.38 | 257960.3 |
| Atalantia buxifolia | 110.2738 | 18.67292 | 107120.4 | 247246.1 |
| Atalantia buxifolia | 110.4211 | 19.32994 |  |  |
| Atalantia buxifolia | 110.3974 | 19.32826 |  |  |
| Atalantia buxifolia | 110.5961 | 19.249 |  |  |
| Atalantia buxifolia | 110.574 | 19.25307 |  |  |
| Atalantia buxifolia | 110.4365 | 18.80764 |  |  |
| Atalantia buxifolia | 110.3728 | 18.77707 |  |  |
| Atalantia buxifolia | 110.4039 | 18.76691 |  |  |
| Atalantia buxifolia | 110.0759 | 18.53203 |  |  |
| Atalantia buxifolia | 110.0985 | 18.51505 |  |  |
| Atalantia buxifolia | 110.076 | 18.56201 |  |  |
| Atalantia buxifolia | 109.7642 | 18.56995 |  |  |
| Atalantia buxifolia | 109.8251 | 18.58075 |  |  |
| Atalantia buxifolia | 109.8672 | 18.60152 |  |  |
| Atalantia buxifolia | 109.6557 | 18.2528 |  |  |
| Atalantia buxifolia | 109.5931 | 18.25855 |  |  |
| Atalantia buxifolia | 109.596 | 18.22666 |  |  |
| Atalantia buxifolia | 109.0451 | 18.69255 |  |  |
| Atalantia buxifolia | 109.298 | 18.80325 |  |  |
| Atalantia buxifolia | 109.0927 | 18.56813 |  |  |
| Atalantia buxifolia | 109.4707 | 19.21961 |  |  |
| Atropa belladonna | 109.0333 | 18.86036 | -22982.8 | 271527.7 |
| Atropa belladonna | 109.0628 | 19.12587 | -18969 | 300785.7 |
| Atropa belladonna | 109.6579 | 18.76335 | 42477.13 | 258885.1 |
| Atropa belladonna | 109.6807 | 18.37829 | 43729.57 | 216234.2 |
| Atropa belladonna | 109.8795 | 18.7193 | 65691.55 | 253395.3 |
| Atropa belladonna | 110.1756 | 18.76435 | 97019.43 | 257602 |
| Atropa belladonna | 110.6577 | 19.59041 | 149719.7 | 347805 |
| Atropa belladonna | 109.7759 | 18.56272 |  |  |
| Atropa belladonna | 109.8238 | 18.57764 |  |  |
| Atropa belladonna | 109.8694 | 18.59683 |  |  |
| Atropa belladonna | 109.8658 | 18.60215 |  |  |
| Atropa belladonna | 109.853 | 18.60807 |  |  |
| Atropa belladonna | 109.8705 | 18.60615 |  |  |
| Atropa belladonna | 109.8709 | 18.59962 |  |  |
| Atropa belladonna | 109.8567 | 18.60507 |  |  |
| Atropa belladonna | 109.8641 | 18.60793 |  |  |
| Atropa belladonna | 109.8662 | 18.603 |  |  |
| Atropa belladonna | 109.7725 | 18.56984 |  |  |
| Atropa belladonna | 109.7776 | 18.565 |  |  |
| Atropa belladonna | 109.7843 | 18.55801 |  |  |
| Atropa belladonna | 109.7823 | 18.56489 |  |  |
| Atropa belladonna | 109.8344 | 18.57764 |  |  |
| Atropa belladonna | 109.827 | 18.58027 |  |  |
| Atropa belladonna | 109.8274 | 18.58236 |  |  |
| Atropa belladonna | 109.8256 | 18.58645 |  |  |
| Atropa belladonna | 109.8345 | 18.57817 |  |  |
| Atropa belladonna | 109.8272 | 18.57561 |  |  |
| Aucuba chinensis | 109.416 | 18.58215 | 16423.77 | 239559.1 |
| Aucuba chinensis | 109.5377 | 19.09747 | 30846.03 | 296177.5 |
| Aucuba chinensis | 109.6975 | 18.72833 | 46544.54 | 254900 |
| Aucuba chinensis | 110.0558 | 18.55317 |  |  |
| Aucuba chinensis | 110.0608 | 18.55043 |  |  |
| Aucuba chinensis | 110.0754 | 18.55517 |  |  |
| Aucuba chinensis | 110.0777 | 18.53566 |  |  |
| Aucuba chinensis | 110.06 | 18.51356 |  |  |
| Aucuba chinensis | 109.4708 | 19.2174 |  |  |
| Aucuba chinensis | 109.466 | 19.21824 |  |  |
| Aucuba chinensis | 109.3471 | 19.2165 |  |  |
| Aucuba chinensis | 109.353 | 19.21515 |  |  |
| Aucuba chinensis | 109.3854 | 19.23217 |  |  |
| Aucuba chinensis | 109.3873 | 19.23562 |  |  |
| Aucuba chinensis | 109.3923 | 19.23177 |  |  |
| Aucuba chinensis | 109.7652 | 18.5701 |  |  |
| Aucuba chinensis | 109.7651 | 18.5642 |  |  |
| Aucuba chinensis | 109.8597 | 18.59901 |  |  |
| Aucuba chinensis | 109.8611 | 18.60828 |  |  |
| Aucuba chinensis | 109.8558 | 18.59692 |  |  |
| Aucuba chinensis | 109.8136 | 18.58543 |  |  |
| Aucuba chinensis | 109.8285 | 18.5865 |  |  |
| Aucuba chinensis | 109.8199 | 18.58417 |  |  |
| Baccaurea ramiflora | 108.9122 | 18.99917 | -35232.9 | 287275.8 |
| Baccaurea ramiflora | 109.3011 | 18.85155 | 5180.119 | 269701.6 |
| Baccaurea ramiflora | 109.6807 | 18.37829 | 43729.57 | 216234.2 |
| Baccaurea ramiflora | 109.837 | 18.73456 | 61260.82 | 255199 |
| Baccaurea ramiflora | 110.2356 | 18.69662 | 103162.5 | 249961.2 |
| Baccaurea ramiflora | 108.7371 | 19.04093 |  |  |
| Baccaurea ramiflora | 108.7461 | 19.06552 |  |  |
| Baccaurea ramiflora | 108.8009 | 18.95834 |  |  |
| Baccaurea ramiflora | 108.9999 | 18.67104 |  |  |
| Baccaurea ramiflora | 109.2748 | 18.78838 |  |  |
| Baccaurea ramiflora | 109.0899 | 18.59372 |  |  |
| Baccaurea ramiflora | 109.6536 | 18.25264 |  |  |
| Baccaurea ramiflora | 109.5943 | 18.25906 |  |  |
| Baccaurea ramiflora | 109.5961 | 18.22576 |  |  |
| Baccaurea ramiflora | 109.7707 | 18.56143 |  |  |
| Baccaurea ramiflora | 109.8312 | 18.5773 |  |  |
| Baccaurea ramiflora | 109.8494 | 18.60046 |  |  |
| Baccaurea ramiflora | 109.6198 | 19.43776 |  |  |
| Baccaurea ramiflora | 109.7276 | 19.55392 |  |  |
| Baccaurea ramiflora | 110.0564 | 18.53801 |  |  |
| Baccaurea ramiflora | 110.087 | 18.53715 |  |  |
| Baccaurea ramiflora | 109.7752 | 19.07483 |  |  |
| Baccaurea ramiflora | 109.8757 | 19.07668 |  |  |
| Baccaurea ramiflora | 109.7893 | 18.99943 |  |  |
| Baccaurea ramiflora | 109.9217 | 19.05474 |  |  |
| Baeckea frutescens | 108.7404 | 18.5875 | -54799.4 | 242345.9 |
| Baeckea frutescens | 108.9795 | 18.89322 | -28525.9 | 275338 |
| Baeckea frutescens | 109.3442 | 19.68663 | 12404.3 | 361885.9 |
| Baeckea frutescens | 109.7575 | 19.47974 | 55069.1 | 337810.7 |
| Baeckea frutescens | 110.1841 | 18.62937 | 97550.3 | 242652.2 |
| Baeckea frutescens | 109.4294 | 18.77949 |  |  |
| Baeckea frutescens | 109.0088 | 19.14408 |  |  |
| Baeckea frutescens | 109.9155 | 18.86435 |  |  |
| Baeckea frutescens | 109.5824 | 19.05609 |  |  |
| Baeckea frutescens | 109.8258 | 19.02076 |  |  |
| Baeckea frutescens | 109.5596 | 18.7477 |  |  |
| Baeckea frutescens | 109.6591 | 18.7658 |  |  |
| Baeckea frutescens | 109.9135 | 18.67296 |  |  |
| Baeckea frutescens | 108.9637 | 18.81618 |  |  |
| Baeckea frutescens | 110.0017 | 19.00143 |  |  |
| Baeckea frutescens | 110.2822 | 19.62682 |  |  |
| Baeckea frutescens | 109.7603 | 19.35403 |  |  |
| Baeckea frutescens | 109.8009 | 19.84647 |  |  |
| Baeckea frutescens | 110.292 | 19.60707 |  |  |
| Baeckea frutescens | 110.212 | 19.7537 |  |  |
| Baeckea frutescens | 110.3738 | 19.63423 |  |  |
| Baeckea frutescens | 110.2814 | 19.8212 |  |  |
| Baeckea frutescens | 110.4537 | 19.84219 |  |  |
| Baeckea frutescens | 109.8361 | 19.82058 |  |  |
| Baeckea frutescens | 109.718 | 19.65544 |  |  |
| Bambusa bambos | 110.1756 | 18.76435 | 97019.43 | 257602 |
| Bambusa bambos | 109.7667 | 18.56006 |  |  |
| Bambusa bambos | 109.8137 | 18.58565 |  |  |
| Bambusa bambos | 109.8638 | 18.60559 |  |  |
| Bambusa bambos | 109.6487 | 18.25356 |  |  |
| Bambusa bambos | 109.5943 | 18.26162 |  |  |
| Bambusa bambos | 109.5987 | 18.22669 |  |  |
| Bambusa bambos | 108.7441 | 19.04442 |  |  |
| Bambusa bambos | 108.7468 | 19.06689 |  |  |
| Bambusa bambos | 108.783 | 18.98663 |  |  |
| Bambusa bambos | 110.0762 | 18.54348 |  |  |
| Bambusa bambos | 110.0547 | 18.54385 |  |  |
| Bambusa bambos | 110.0881 | 18.52194 |  |  |
| Bambusa bambos | 109.6876 | 19.53652 |  |  |
| Bambusa bambos | 109.5778 | 19.55236 |  |  |
| Bambusa bambos | 109.6349 | 19.43029 |  |  |
| Bambusa bambos | 110.4296 | 19.30092 |  |  |
| Bambusa bambos | 110.383 | 19.32854 |  |  |
| Bambusa bambos | 110.5801 | 19.25748 |  |  |
| Bambusa bambos | 110.5757 | 19.27128 |  |  |
| Bambusa bambos | 110.8981 | 19.60877 |  |  |
| Bambusa beecheyana | 109.3736 | 18.89125 | 12945.04 | 273868.1 |
| Bambusa beecheyana | 109.6735 | 18.88732 | 44494.38 | 272548.8 |
| Bambusa beecheyana | 109.675 | 18.86962 | 44597.81 | 270587.7 |
| Bambusa beecheyana | 109.6975 | 18.72833 | 46544.54 | 254900 |
| Bambusa beecheyana | 110.9143 | 19.67361 | 176777.4 | 356463.4 |
| Bambusa beecheyana | 109.7815 | 18.56676 |  |  |
| Bambusa beecheyana | 109.8228 | 18.57764 |  |  |
| Bambusa beecheyana | 109.8624 | 18.60709 |  |  |
| Bambusa beecheyana | 109.4713 | 19.21987 |  |  |
| Bambusa beecheyana | 109.3847 | 19.23331 |  |  |
| Bambusa beecheyana | 109.3523 | 19.21016 |  |  |
| Bambusa beecheyana | 109.6504 | 18.2516 |  |  |
| Bambusa beecheyana | 109.592 | 18.26053 |  |  |
| Bambusa beecheyana | 109.5926 | 18.22417 |  |  |
| Bambusa beecheyana | 109.5956 | 18.22201 |  |  |
| Bambusa beecheyana | 110.4024 | 19.30756 |  |  |
| Bambusa beecheyana | 110.4136 | 19.30359 |  |  |
| Bambusa beecheyana | 110.567 | 19.25766 |  |  |
| Bambusa beecheyana | 110.5693 | 19.23452 |  |  |
| Bambusa beecheyana | 110.6056 | 19.23755 |  |  |
| Bambusa beecheyana | 109.814 | 18.97675 |  |  |
| Bambusa beecheyana | 109.7805 | 19.1141 |  |  |
| Bambusa beecheyana | 109.8706 | 18.99527 |  |  |
| Bambusa beecheyana | 109.8374 | 19.02464 |  |  |
| Bambusa beecheyana | 109.9077 | 19.01818 |  |  |
| Bambusa boniopsis | 109.4282 | 18.35624 | 16993.03 | 214537.5 |
| Bambusa boniopsis | 109.5153 | 18.61925 | 27012 | 243364.4 |
| Bambusa boniopsis | 109.6535 | 18.2575 | 40487.08 | 202951.5 |
| Bambusa boniopsis | 109.9578 | 18.76128 | 74063.58 | 257827 |
| Bambusa boniopsis | 110.4793 | 19.87687 | 131725.4 | 379875.5 |
| Bambusa boniopsis | 109.8802 | 18.80257 |  |  |
| Bambusa boniopsis | 109.8726 | 18.78684 |  |  |
| Bambusa boniopsis | 109.881 | 18.79451 |  |  |
| Bambusa boniopsis | 109.8691 | 18.79362 |  |  |
| Bambusa boniopsis | 109.8719 | 18.79042 |  |  |
| Bambusa boniopsis | 109.8829 | 18.79388 |  |  |
| Bambusa boniopsis | 109.8691 | 18.80222 |  |  |
| Bambusa boniopsis | 109.8667 | 18.78994 |  |  |
| Bambusa boniopsis | 109.871 | 18.7848 |  |  |
| Bambusa boniopsis | 109.8762 | 18.78491 |  |  |
| Bambusa boniopsis | 109.2694 | 19.03425 |  |  |
| Bambusa boniopsis | 109.2734 | 19.03847 |  |  |
| Bambusa boniopsis | 109.2574 | 19.0347 |  |  |
| Bambusa boniopsis | 109.2566 | 19.0406 |  |  |
| Bambusa boniopsis | 109.2568 | 19.03624 |  |  |
| Bambusa boniopsis | 109.2706 | 19.03775 |  |  |
| Bambusa boniopsis | 109.2673 | 19.04503 |  |  |
| Bambusa boniopsis | 109.2708 | 19.04083 |  |  |
| Bambusa boniopsis | 109.2613 | 19.04044 |  |  |
| Bambusa boniopsis | 109.2735 | 19.03551 |  |  |
| Bambusa chungii | 109.6401 | 19.49205 | 42797.58 | 339502 |
| Bambusa chungii | 109.7326 | 19.59786 | 52815.33 | 350936.9 |
| Bambusa chungii | 109.891 | 18.96479 | 67604.6 | 280511.3 |
| Bambusa chungii | 110.4793 | 19.87687 | 131725.4 | 379875.5 |
| Bambusa chungii | 110.2151 | 20.01169 |  |  |
| Bambusa chungii | 110.4616 | 19.85266 |  |  |
| Bambusa chungii | 110.4158 | 19.84731 |  |  |
| Bambusa chungii | 110.4122 | 19.85376 |  |  |
| Bambusa chungii | 110.4136 | 19.85475 |  |  |
| Bambusa chungii | 110.4146 | 19.84947 |  |  |
| Bambusa chungii | 110.3792 | 19.29514 |  |  |
| Bambusa chungii | 110.4103 | 19.30647 |  |  |
| Bambusa chungii | 110.5631 | 19.24557 |  |  |
| Bambusa chungii | 110.5518 | 19.23846 |  |  |
| Bambusa chungii | 110.8081 | 19.69245 |  |  |
| Bambusa chungii | 110.8171 | 19.55521 |  |  |
| Bambusa chungii | 109.7152 | 19.55102 |  |  |
| Bambusa chungii | 109.7263 | 19.55376 |  |  |
| Bambusa chungii | 109.583 | 19.54844 |  |  |
| Bambusa chungii | 109.9036 | 18.96544 |  |  |
| Bambusa chungii | 109.7751 | 19.00208 |  |  |
| Bambusa chungii | 109.6722 | 19.95493 |  |  |
| Bambusa chungii | 109.7391 | 19.91016 |  |  |
| Bambusa chungii | 109.6413 | 19.87361 |  |  |
| Bambusa flexuosa | 110.1756 | 18.76435 | 97019.43 | 257602 |
| Bambusa flexuosa | 110.0565 | 18.81705 |  |  |
| Bambusa flexuosa | 108.9597 | 19.24683 |  |  |
| Bambusa flexuosa | 109.0762 | 18.86632 |  |  |
| Bambusa flexuosa | 109.7746 | 18.71178 |  |  |
| Bambusa flexuosa | 109.4672 | 18.98206 |  |  |
| Bambusa flexuosa | 110.0585 | 19.24035 |  |  |
| Bambusa flexuosa | 109.3345 | 19.10347 |  |  |
| Bambusa flexuosa | 109.7888 | 19.18735 |  |  |
| Bambusa flexuosa | 109.6865 | 19.23892 |  |  |
| Bambusa flexuosa | 109.6285 | 19.80959 |  |  |
| Bambusa flexuosa | 109.6198 | 19.23914 |  |  |
| Bambusa flexuosa | 110.4038 | 19.54581 |  |  |
| Bambusa flexuosa | 110.3638 | 19.68056 |  |  |
| Bambusa flexuosa | 110.3739 | 19.16522 |  |  |
| Bambusa flexuosa | 110.0205 | 19.56849 |  |  |
| Bambusa flexuosa | 109.8092 | 19.59406 |  |  |
| Bambusa flexuosa | 110.0145 | 19.61036 |  |  |
| Bambusa flexuosa | 110.3021 | 19.49124 |  |  |
| Bambusa flexuosa | 110.3712 | 19.48601 |  |  |
| Bambusa flexuosa | 109.8453 | 19.85376 |  |  |
| Bambusa textilis | 110.1756 | 18.76435 | 97019.43 | 257602 |
| Bambusa textilis | 110.1756 | 18.76435 | 97019.54 | 257602.3 |
| Bambusa textilis | 109.6466 | 19.0897 |  |  |
| Bambusa textilis | 109.6329 | 19.1681 |  |  |
| Bambusa textilis | 109.9112 | 19.2157 |  |  |
| Bambusa textilis | 109.0932 | 19.09207 |  |  |
| Bambusa textilis | 108.9748 | 18.79589 |  |  |
| Bambusa textilis | 109.2796 | 18.8741 |  |  |
| Bambusa textilis | 109.9388 | 19.14758 |  |  |
| Bambusa textilis | 109.5105 | 19.11773 |  |  |
| Bambusa textilis | 109.1976 | 18.8367 |  |  |
| Bambusa textilis | 110.3562 | 19.8533 |  |  |
| Bambusa textilis | 109.6378 | 19.84856 |  |  |
| Bambusa textilis | 109.6619 | 19.74722 |  |  |
| Bambusa textilis | 110.1849 | 19.50889 |  |  |
| Bambusa textilis | 109.709 | 19.77957 |  |  |
| Bambusa textilis | 109.945 | 19.29104 |  |  |
| Bambusa textilis | 109.8426 | 19.3919 |  |  |
| Bambusa textilis | 109.8382 | 19.17925 |  |  |
| Bambusa textilis | 110.0898 | 19.40665 |  |  |
| Bambusa textilis | 109.7456 | 19.63162 |  |  |
| Bambusa textilis | 109.7177 | 19.69082 |  |  |
| Barleria cristata | 109.0333 | 18.86036 | -22982.8 | 271527.7 |
| Barleria cristata | 109.0799 | 19.00987 | -17563.9 | 287906.3 |
| Barleria cristata | 109.4916 | 18.78671 | 25037.28 | 261953.3 |
| Barleria cristata | 109.6807 | 18.37829 | 43729.57 | 216234.2 |
| Barleria cristata | 110.4935 | 18.66623 | 130266.4 | 245981.2 |
| Barleria cristata | 109.5091 | 18.85316 |  |  |
| Barleria cristata | 109.8722 | 19.00454 |  |  |
| Barleria cristata | 110.0223 | 18.87619 |  |  |
| Barleria cristata | 109.3129 | 18.77682 |  |  |
| Barleria cristata | 109.1895 | 18.89662 |  |  |
| Barleria cristata | 109.0296 | 18.81794 |  |  |
| Barleria cristata | 109.4228 | 18.84059 |  |  |
| Barleria cristata | 109.9532 | 18.83463 |  |  |
| Barleria cristata | 109.1495 | 19.16646 |  |  |
| Barleria cristata | 110.0054 | 19.23627 |  |  |
| Barleria cristata | 110.3378 | 19.66479 |  |  |
| Barleria cristata | 110.1587 | 19.65224 |  |  |
| Barleria cristata | 109.8615 | 19.51803 |  |  |
| Barleria cristata | 110.3688 | 19.85063 |  |  |
| Barleria cristata | 109.9391 | 19.1911 |  |  |
| Barleria cristata | 109.8423 | 19.74729 |  |  |
| Barleria cristata | 109.7236 | 19.33201 |  |  |
| Barleria cristata | 110.1631 | 19.82758 |  |  |
| Barleria cristata | 110.0944 | 19.68477 |  |  |
| Barleria cristata | 109.9275 | 19.4531 |  |  |
| Barringtonia racemosa | 110.2695 | 18.67114 | 106662.8 | 247059.7 |
| Barringtonia racemosa | 110.457 | 18.89014 | 126969.4 | 270829.8 |
| Barringtonia racemosa | 110.8034 | 19.73243 |  |  |
| Barringtonia racemosa | 110.7319 | 19.58193 |  |  |
| Barringtonia racemosa | 110.7223 | 19.60209 |  |  |
| Barringtonia racemosa | 110.4236 | 18.79539 |  |  |
| Barringtonia racemosa | 110.3785 | 18.77691 |  |  |
| Barringtonia racemosa | 110.418 | 18.77218 |  |  |
| Barringtonia racemosa | 110.0562 | 18.52914 |  |  |
| Barringtonia racemosa | 110.0728 | 18.53408 |  |  |
| Barringtonia racemosa | 110.0725 | 18.55393 |  |  |
| Barringtonia racemosa | 110.0788 | 18.53272 |  |  |
| Barringtonia racemosa | 110.087 | 18.54671 |  |  |
| Barringtonia racemosa | 110.0921 | 18.53608 |  |  |
| Barringtonia racemosa | 109.7772 | 18.5613 |  |  |
| Barringtonia racemosa | 109.8171 | 18.57591 |  |  |
| Barringtonia racemosa | 109.8561 | 18.59753 |  |  |
| Barringtonia racemosa | 109.8588 | 18.60367 |  |  |
| Barringtonia racemosa | 109.8605 | 18.60393 |  |  |
| Barringtonia racemosa | 109.8592 | 18.60558 |  |  |
| Barringtonia racemosa | 109.8534 | 18.5972 |  |  |
| Barringtonia racemosa | 109.8593 | 18.60747 |  |  |
| Bauhinia championii | 109.1544 | 19.01211 | -9728.08 | 287914.8 |
| Bauhinia championii | 109.3011 | 18.85155 | 5180.119 | 269701.6 |
| Bauhinia championii | 109.5106 | 18.24137 | 25337.66 | 201585.1 |
| Bauhinia championii | 109.777 | 19.17887 | 56233.56 | 304494 |
| Bauhinia championii | 110.1756 | 18.76435 | 97019.43 | 257602 |
| Bauhinia championii | 109.6825 | 19.44816 |  |  |
| Bauhinia championii | 109.6576 | 19.5717 |  |  |
| Bauhinia championii | 109.6216 | 19.52841 |  |  |
| Bauhinia championii | 109.6218 | 19.50928 |  |  |
| Bauhinia championii | 109.642 | 19.50682 |  |  |
| Bauhinia championii | 109.678 | 19.43483 |  |  |
| Bauhinia championii | 110.3952 | 19.28858 |  |  |
| Bauhinia championii | 110.4145 | 19.3277 |  |  |
| Bauhinia championii | 110.3809 | 19.2924 |  |  |
| Bauhinia championii | 110.4174 | 19.31524 |  |  |
| Bauhinia championii | 110.3827 | 19.3097 |  |  |
| Bauhinia championii | 110.4111 | 19.31363 |  |  |
| Bauhinia championii | 110.4271 | 18.80357 |  |  |
| Bauhinia championii | 110.3743 | 18.77454 |  |  |
| Bauhinia championii | 110.4112 | 18.76085 |  |  |
| Bauhinia championii | 110.4367 | 18.81107 |  |  |
| Bauhinia championii | 110.3737 | 18.77346 |  |  |
| Bauhinia championii | 110.427 | 18.7443 |  |  |
| Bauhinia championii | 110.4365 | 18.7828 |  |  |
| Bauhinia championii | 110.4304 | 18.73189 |  |  |
| Bauhinia corymbosa | 109.6555 | 18.5381 | 41546.81 | 233982.1 |
| Bauhinia corymbosa | 109.837 | 18.73456 | 61260.82 | 255199 |
| Bauhinia corymbosa | 110.1756 | 18.76435 | 97019.43 | 257602 |
| Bauhinia corymbosa | 110.3309 | 19.265 | 114648.9 | 312579.5 |
| Bauhinia corymbosa | 110.4612 | 19.34424 | 128523.6 | 321032.5 |
| Bauhinia corymbosa | 110.2531 | 19.53624 |  |  |
| Bauhinia corymbosa | 110.2235 | 19.62526 |  |  |
| Bauhinia corymbosa | 110.3063 | 19.64782 |  |  |
| Bauhinia corymbosa | 110.4058 | 19.30972 |  |  |
| Bauhinia corymbosa | 110.3708 | 19.30801 |  |  |
| Bauhinia corymbosa | 110.5565 | 19.26242 |  |  |
| Bauhinia corymbosa | 110.5799 | 19.23164 |  |  |
| Bauhinia corymbosa | 110.4333 | 18.79457 |  |  |
| Bauhinia corymbosa | 110.3753 | 18.77254 |  |  |
| Bauhinia corymbosa | 110.4222 | 18.77477 |  |  |
| Bauhinia corymbosa | 109.7713 | 18.57151 |  |  |
| Bauhinia corymbosa | 109.8135 | 18.58236 |  |  |
| Bauhinia corymbosa | 109.8593 | 18.60391 |  |  |
| Bauhinia corymbosa | 110.0935 | 18.53086 |  |  |
| Bauhinia corymbosa | 110.0865 | 18.52846 |  |  |
| Bauhinia corymbosa | 109.6495 | 18.25416 |  |  |
| Bauhinia corymbosa | 109.6584 | 18.25305 |  |  |
| Bauhinia corymbosa | 109.5949 | 18.22534 |  |  |
| Bauhinia corymbosa | 109.6009 | 18.2585 |  |  |
| Bauhinia corymbosa | 109.5995 | 18.26204 |  |  |
| Bauhinia hainanensis | 108.7989 | 18.69619 | -48238.4 | 254162.2 |
| Bauhinia hainanensis | 109.0333 | 18.86036 | -22982.8 | 271527.7 |
| Bauhinia hainanensis | 109.1191 | 19.05985 | -13271 | 293304.6 |
| Bauhinia hainanensis | 109.1741 | 18.37558 | -9781.56 | 217466.3 |
| Bauhinia hainanensis | 109.6495 | 18.24349 | 40023.82 | 201413.4 |
| Bauhinia hainanensis | 109.6513 | 18.2497 |  |  |
| Bauhinia hainanensis | 109.6564 | 18.25424 |  |  |
| Bauhinia hainanensis | 109.6568 | 18.24944 |  |  |
| Bauhinia hainanensis | 109.6519 | 18.25651 |  |  |
| Bauhinia hainanensis | 109.5973 | 18.22687 |  |  |
| Bauhinia hainanensis | 109.5999 | 18.22628 |  |  |
| Bauhinia hainanensis | 109.5959 | 18.2221 |  |  |
| Bauhinia hainanensis | 109.5959 | 18.22211 |  |  |
| Bauhinia hainanensis | 109.5958 | 18.25743 |  |  |
| Bauhinia hainanensis | 109.5965 | 18.2624 |  |  |
| Bauhinia hainanensis | 109.5933 | 18.2618 |  |  |
| Bauhinia hainanensis | 109.2888 | 18.80273 |  |  |
| Bauhinia hainanensis | 109.2782 | 18.79609 |  |  |
| Bauhinia hainanensis | 109.2868 | 18.77806 |  |  |
| Bauhinia hainanensis | 109.2747 | 18.79702 |  |  |
| Bauhinia hainanensis | 109.2942 | 18.78361 |  |  |
| Bauhinia hainanensis | 109.1422 | 18.58129 |  |  |
| Bauhinia hainanensis | 109.1249 | 18.52028 |  |  |
| Bauhinia hainanensis | 109.1335 | 18.59345 |  |  |
| Bauhinia hainanensis | 109.1321 | 18.53524 |  |  |
| Bauhinia khasiana | 108.6526 | 18.84592 | -63101 | 271225.5 |
| Bauhinia khasiana | 109.0333 | 18.86036 | -22982.8 | 271527.7 |
| Bauhinia khasiana | 109.6807 | 18.37829 | 43729.57 | 216234.2 |
| Bauhinia khasiana | 109.6975 | 18.72833 | 46544.54 | 254900 |
| Bauhinia khasiana | 110.2738 | 18.67292 | 107120.4 | 247246.1 |
| Bauhinia khasiana | 109.6487 | 18.24913 |  |  |
| Bauhinia khasiana | 109.6579 | 18.25224 |  |  |
| Bauhinia khasiana | 109.6548 | 18.25399 |  |  |
| Bauhinia khasiana | 109.6514 | 18.25415 |  |  |
| Bauhinia khasiana | 109.5915 | 18.25929 |  |  |
| Bauhinia khasiana | 109.6003 | 18.26283 |  |  |
| Bauhinia khasiana | 109.5997 | 18.26183 |  |  |
| Bauhinia khasiana | 109.5923 | 18.22289 |  |  |
| Bauhinia khasiana | 109.5989 | 18.2236 |  |  |
| Bauhinia khasiana | 109.5979 | 18.22512 |  |  |
| Bauhinia khasiana | 109.5979 | 18.222 |  |  |
| Bauhinia khasiana | 110.0817 | 18.55184 |  |  |
| Bauhinia khasiana | 110.0657 | 18.5442 |  |  |
| Bauhinia khasiana | 110.0952 | 18.51761 |  |  |
| Bauhinia khasiana | 109.9808 | 18.55149 |  |  |
| Bauhinia khasiana | 110.0244 | 18.47433 |  |  |
| Bauhinia khasiana | 110.0525 | 18.49452 |  |  |
| Bauhinia khasiana | 110.0134 | 18.60844 |  |  |
| Bauhinia khasiana | 110.082 | 18.6234 |  |  |
| Bauhinia khasiana | 110.1459 | 18.60258 |  |  |
| Bauhinia scandens | 109.0333 | 18.86036 | -22982.8 | 271527.7 |
| Bauhinia scandens | 109.0799 | 19.00987 | -17563.9 | 287906.3 |
| Bauhinia scandens | 109.0973 | 19.21705 | -15036.4 | 310753.6 |
| Bauhinia scandens | 109.1191 | 19.05985 | -13271 | 293304.6 |
| Bauhinia scandens | 109.1741 | 18.37558 | -9781.56 | 217466.3 |
| Bauhinia scandens | 109.6575 | 18.25663 |  |  |
| Bauhinia scandens | 109.6573 | 18.25134 |  |  |
| Bauhinia scandens | 109.5976 | 18.22186 |  |  |
| Bauhinia scandens | 109.5911 | 18.22274 |  |  |
| Bauhinia scandens | 109.6001 | 18.25708 |  |  |
| Bauhinia scandens | 109.5997 | 18.25997 |  |  |
| Bauhinia scandens | 109.0381 | 19.28199 |  |  |
| Bauhinia scandens | 108.9889 | 19.31164 |  |  |
| Bauhinia scandens | 109.0355 | 19.28028 |  |  |
| Bauhinia scandens | 109.1693 | 19.32875 |  |  |
| Bauhinia scandens | 109.1275 | 19.36859 |  |  |
| Bauhinia scandens | 109.0937 | 19.34129 |  |  |
| Bauhinia scandens | 108.7336 | 19.04832 |  |  |
| Bauhinia scandens | 108.7336 | 19.04812 |  |  |
| Bauhinia scandens | 108.783 | 18.95424 |  |  |
| Bauhinia scandens | 108.7844 | 18.99059 |  |  |
| Bauhinia scandens | 108.8042 | 18.97771 |  |  |
| Bauhinia scandens | 108.7444 | 19.06645 |  |  |
| Bauhinia scandens | 108.7468 | 19.06548 |  |  |
| Bauhinia scandens | 108.7463 | 19.06353 |  |  |
| Beilschmiedia appendiculata | 108.7989 | 18.69619 | -48238.4 | 254162.2 |
| Beilschmiedia appendiculata | 109.1741 | 18.37558 | -9781.56 | 217466.3 |
| Beilschmiedia appendiculata | 109.5425 | 19.03583 | 31162.38 | 289348.8 |
| Beilschmiedia appendiculata | 109.6964 | 19.03697 | 47343.87 | 289030.5 |
| Beilschmiedia appendiculata | 109.7426 | 19.4888 | 53537.07 | 338853.4 |
| Beilschmiedia appendiculata | 110.1167 | 19.3938 | 92504.95 | 327351.7 |
| Beilschmiedia appendiculata | 109.122 | 19.08941 |  |  |
| Beilschmiedia appendiculata | 109.1193 | 19.08761 |  |  |
| Beilschmiedia appendiculata | 109.1241 | 19.089 |  |  |
| Beilschmiedia appendiculata | 109.1221 | 19.08678 |  |  |
| Beilschmiedia appendiculata | 109.8857 | 18.79797 |  |  |
| Beilschmiedia appendiculata | 109.8756 | 18.78547 |  |  |
| Beilschmiedia appendiculata | 109.8817 | 18.7864 |  |  |
| Beilschmiedia appendiculata | 109.8715 | 18.78973 |  |  |
| Beilschmiedia appendiculata | 109.2561 | 19.04309 |  |  |
| Beilschmiedia appendiculata | 109.2664 | 19.04545 |  |  |
| Beilschmiedia appendiculata | 109.2574 | 19.04477 |  |  |
| Beilschmiedia appendiculata | 109.2709 | 19.03891 |  |  |
| Beilschmiedia appendiculata | 109.8693 | 19.09399 |  |  |
| Beilschmiedia appendiculata | 109.8675 | 19.09715 |  |  |
| Beilschmiedia appendiculata | 109.8683 | 19.09374 |  |  |
| Beilschmiedia appendiculata | 109.864 | 19.09657 |  |  |
| Beilschmiedia appendiculata | 109.8681 | 19.09501 |  |  |
| Beilschmiedia appendiculata | 109.5442 | 19.03704 |  |  |
| Beilschmiedia appendiculata | 109.5508 | 19.03136 |  |  |
| Beilschmiedia appendiculata | 109.5447 | 19.03494 |  |  |
| Beilschmiedia intermedia | 108.8228 | 18.4993 | -46431 | 232309.2 |
| Beilschmiedia intermedia | 109.0333 | 18.86036 | -22982.8 | 271527.7 |
| Beilschmiedia intermedia | 109.1191 | 19.05985 | -13271 | 293304.6 |
| Beilschmiedia intermedia | 109.5425 | 19.03583 | 31162.38 | 289348.8 |
| Beilschmiedia intermedia | 109.837 | 18.73456 | 61260.82 | 255199 |
| Beilschmiedia intermedia | 110.2238 | 18.67552 | 101866.1 | 247657.4 |
| Beilschmiedia intermedia | 110.2356 | 18.69662 | 103162.5 | 249961.2 |
| Beilschmiedia intermedia | 109.0353 | 19.02894 |  |  |
| Beilschmiedia intermedia | 109.4687 | 19.02587 |  |  |
| Beilschmiedia intermedia | 110.0326 | 19.16767 |  |  |
| Beilschmiedia intermedia | 109.7986 | 19.1082 |  |  |
| Beilschmiedia intermedia | 109.5327 | 18.87732 |  |  |
| Beilschmiedia intermedia | 108.9474 | 18.77595 |  |  |
| Beilschmiedia intermedia | 109.3724 | 18.9783 |  |  |
| Beilschmiedia intermedia | 110.0605 | 19.23159 |  |  |
| Beilschmiedia intermedia | 109.28 | 18.68291 |  |  |
| Beilschmiedia intermedia | 109.7095 | 19.76236 |  |  |
| Beilschmiedia intermedia | 109.6802 | 19.79346 |  |  |
| Beilschmiedia intermedia | 109.8309 | 19.82994 |  |  |
| Beilschmiedia intermedia | 109.634 | 19.80479 |  |  |
| Beilschmiedia intermedia | 110.4056 | 19.3635 |  |  |
| Beilschmiedia intermedia | 109.8357 | 19.49686 |  |  |
| Beilschmiedia intermedia | 109.7124 | 19.84923 |  |  |
| Beilschmiedia intermedia | 110.504 | 19.45719 |  |  |
| Beilschmiedia intermedia | 109.7738 | 19.84226 |  |  |
| Beilschmiedia intermedia | 110.3447 | 19.542 |  |  |
| Beilschmiedia intermedia | 110.0817 | 19.64804 |  |  |
| Benkara scandens | 109.1544 | 19.01211 | -9728.08 | 287914.8 |
| Benkara scandens | 110.5189 | 18.97994 | 133703.2 | 280616.8 |
| Benkara scandens | 109.4691 | 19.21942 |  |  |
| Benkara scandens | 109.4669 | 19.21913 |  |  |
| Benkara scandens | 109.3555 | 19.21771 |  |  |
| Benkara scandens | 109.3487 | 19.21875 |  |  |
| Benkara scandens | 109.3457 | 19.21089 |  |  |
| Benkara scandens | 109.3888 | 19.23463 |  |  |
| Benkara scandens | 110.4294 | 18.80368 |  |  |
| Benkara scandens | 110.4239 | 18.79469 |  |  |
| Benkara scandens | 110.4108 | 18.767 |  |  |
| Benkara scandens | 110.4199 | 18.75999 |  |  |
| Benkara scandens | 110.4025 | 18.75412 |  |  |
| Benkara scandens | 110.378 | 18.77438 |  |  |
| Benkara scandens | 110.3693 | 18.77487 |  |  |
| Benkara scandens | 110.3789 | 18.76982 |  |  |
| Benkara scandens | 110.3693 | 18.77092 |  |  |
| Benkara scandens | 110.408 | 19.32915 |  |  |
| Benkara scandens | 110.4024 | 19.28729 |  |  |
| Benkara scandens | 110.4206 | 19.33046 |  |  |
| Benkara scandens | 110.5853 | 19.25618 |  |  |
| Benkara scandens | 110.5587 | 19.25293 |  |  |
| Bennettiodendron leprosipes | 109.6835 | 18.87437 | 45503.17 | 271088.8 |
| Bennettiodendron leprosipes | 109.837 | 18.73456 | 61260.82 | 255199 |
| Bennettiodendron leprosipes | 110.1756 | 18.76435 | 97019.43 | 257602 |
| Bennettiodendron leprosipes | 110.0921 | 18.52576 |  |  |
| Bennettiodendron leprosipes | 110.1055 | 18.55735 |  |  |
| Bennettiodendron leprosipes | 110.0733 | 18.53862 |  |  |
| Bennettiodendron leprosipes | 110.059 | 18.53701 |  |  |
| Bennettiodendron leprosipes | 109.7693 | 18.57179 |  |  |
| Bennettiodendron leprosipes | 109.7685 | 18.55884 |  |  |
| Bennettiodendron leprosipes | 109.8516 | 18.60695 |  |  |
| Bennettiodendron leprosipes | 109.8683 | 18.60742 |  |  |
| Bennettiodendron leprosipes | 109.8711 | 18.59907 |  |  |
| Bennettiodendron leprosipes | 109.8334 | 18.5789 |  |  |
| Bennettiodendron leprosipes | 109.829 | 18.57894 |  |  |
| Bennettiodendron leprosipes | 109.8309 | 18.57517 |  |  |
| Bennettiodendron leprosipes | 109.6516 | 18.2537 |  |  |
| Bennettiodendron leprosipes | 109.6552 | 18.2526 |  |  |
| Bennettiodendron leprosipes | 109.5944 | 18.22534 |  |  |
| Bennettiodendron leprosipes | 109.596 | 18.22716 |  |  |
| Bennettiodendron leprosipes | 109.5995 | 18.22712 |  |  |
| Bennettiodendron leprosipes | 109.5925 | 18.25664 |  |  |
| Bennettiodendron leprosipes | 109.6006 | 18.26286 |  |  |
| Bennettiodendron leprosipes | 109.5949 | 18.25631 |  |  |
| Berchemia floribunda | 109.0333 | 18.86036 | -22982.8 | 271527.7 |
| Berchemia floribunda | 109.0609 | 18.7429 | -20480.1 | 258450.9 |
| Berchemia floribunda | 109.6835 | 18.87437 | 45503.17 | 271088.8 |
| Berchemia floribunda | 109.7575 | 19.47974 | 55069.1 | 337810.7 |
| Berchemia floribunda | 110.1756 | 18.76435 | 97019.43 | 257602 |
| Berchemia floribunda | 110.7136 | 19.45974 | 155270.9 | 333236.6 |
| Berchemia floribunda | 110.0299 | 19.79418 |  |  |
| Berchemia floribunda | 110.0433 | 19.75912 |  |  |
| Berchemia floribunda | 110.0167 | 19.73635 |  |  |
| Berchemia floribunda | 109.9768 | 19.80887 |  |  |
| Berchemia floribunda | 109.2894 | 18.78501 |  |  |
| Berchemia floribunda | 109.2926 | 18.77769 |  |  |
| Berchemia floribunda | 109.2966 | 18.78634 |  |  |
| Berchemia floribunda | 109.2887 | 18.80161 |  |  |
| Berchemia floribunda | 109.1335 | 18.60653 |  |  |
| Berchemia floribunda | 109.0487 | 18.56005 |  |  |
| Berchemia floribunda | 109.1094 | 18.61788 |  |  |
| Berchemia floribunda | 109.7683 | 18.56731 |  |  |
| Berchemia floribunda | 109.7722 | 18.57174 |  |  |
| Berchemia floribunda | 109.7701 | 18.57323 |  |  |
| Berchemia floribunda | 109.8559 | 18.59825 |  |  |
| Berchemia floribunda | 109.8488 | 18.60653 |  |  |
| Berchemia floribunda | 109.8648 | 18.59577 |  |  |
| Berchemia floribunda | 109.8187 | 18.57963 |  |  |
| Berchemia floribunda | 109.8136 | 18.5745 |  |  |
| Berchemia floribunda | 109.8187 | 18.57807 |  |  |
| Berchemia lineata | 110.2738 | 18.67292 | 107120.4 | 247246.1 |
| Berchemia lineata | 109.089 | 19.00506 |  |  |
| Berchemia lineata | 109.4073 | 19.06207 |  |  |
| Berchemia lineata | 109.3176 | 18.71985 |  |  |
| Berchemia lineata | 109.0386 | 18.93719 |  |  |
| Berchemia lineata | 108.9674 | 18.86395 |  |  |
| Berchemia lineata | 108.9352 | 18.80685 |  |  |
| Berchemia lineata | 108.9889 | 18.69144 |  |  |
| Berchemia lineata | 109.2589 | 19.20531 |  |  |
| Berchemia lineata | 109.2122 | 19.23481 |  |  |
| Berchemia lineata | 108.9806 | 19.17618 |  |  |
| Berchemia lineata | 110.103 | 19.18745 |  |  |
| Berchemia lineata | 110.2961 | 19.56897 |  |  |
| Berchemia lineata | 110.1834 | 19.80778 |  |  |
| Berchemia lineata | 110.2268 | 19.68305 |  |  |
| Berchemia lineata | 109.8726 | 19.32126 |  |  |
| Berchemia lineata | 109.7285 | 19.49436 |  |  |
| Berchemia lineata | 110.3965 | 19.4169 |  |  |
| Berchemia lineata | 109.8014 | 19.30638 |  |  |
| Berchemia lineata | 110.0291 | 19.49745 |  |  |
| Berchemia lineata | 109.8113 | 19.8122 |  |  |
| Berchemia polyphylla | 109.416 | 18.58215 | 16423.77 | 239559.1 |
| Berchemia polyphylla | 110.3564 | 18.68108 | 115854.4 | 247947.5 |
| Berchemia polyphylla | 110.4177 | 19.76786 | 125007.5 | 367967.2 |
| Berchemia polyphylla | 110.4194 | 18.9871 | 123247.6 | 281640.2 |
| Berchemia polyphylla | 110.6232 | 19.24781 | 145301.2 | 310002.2 |
| Berchemia polyphylla | 110.8845 | 19.93617 | 174229.8 | 385550.5 |
| Berchemia polyphylla | 110.0542 | 18.5159 |  |  |
| Berchemia polyphylla | 110.0613 | 18.52615 |  |  |
| Berchemia polyphylla | 110.0695 | 18.52667 |  |  |
| Berchemia polyphylla | 110.085 | 18.54815 |  |  |
| Berchemia polyphylla | 110.4273 | 18.79924 |  |  |
| Berchemia polyphylla | 110.4319 | 18.80835 |  |  |
| Berchemia polyphylla | 110.4082 | 18.77457 |  |  |
| Berchemia polyphylla | 110.4041 | 18.77068 |  |  |
| Berchemia polyphylla | 110.4121 | 18.77625 |  |  |
| Berchemia polyphylla | 110.3789 | 18.77047 |  |  |
| Berchemia polyphylla | 110.3731 | 18.77506 |  |  |
| Berchemia polyphylla | 110.3781 | 18.76897 |  |  |
| Berchemia polyphylla | 110.3803 | 18.77361 |  |  |
| Berchemia polyphylla | 109.8493 | 19.05901 |  |  |
| Berchemia polyphylla | 109.8604 | 18.96962 |  |  |
| Berchemia polyphylla | 109.9076 | 19.11371 |  |  |
| Berchemia polyphylla | 109.8196 | 18.94276 |  |  |
| Berchemia polyphylla | 109.955 | 19.01596 |  |  |
| Berchemia polyphylla | 110.0237 | 19.064 |  |  |
| Berchemia polyphylla | 110.0457 | 19.01077 |  |  |
| Bischofia javanica | 109.3011 | 18.85155 | 5180.119 | 269701.6 |
| Bischofia javanica | 109.7575 | 19.47974 | 55069.1 | 337810.7 |
| Bischofia javanica | 110.1756 | 18.76435 | 97019.43 | 257602 |
| Bischofia javanica | 110.3582 | 19.99295 | 119346.6 | 392991 |
| Bischofia javanica | 109.9881 | 18.69776 |  |  |
| Bischofia javanica | 109.8693 | 19.17413 |  |  |
| Bischofia javanica | 109.8365 | 18.75807 |  |  |
| Bischofia javanica | 109.9234 | 18.66599 |  |  |
| Bischofia javanica | 109.6091 | 18.76622 |  |  |
| Bischofia javanica | 109.7788 | 19.14029 |  |  |
| Bischofia javanica | 110.0183 | 19.17615 |  |  |
| Bischofia javanica | 109.0869 | 19.01139 |  |  |
| Bischofia javanica | 109.8913 | 18.89702 |  |  |
| Bischofia javanica | 109.1574 | 18.79668 |  |  |
| Bischofia javanica | 109.137 | 19.17466 |  |  |
| Bischofia javanica | 110.4755 | 19.5936 |  |  |
| Bischofia javanica | 110.045 | 19.54468 |  |  |
| Bischofia javanica | 109.9397 | 19.41896 |  |  |
| Bischofia javanica | 109.9062 | 19.18818 |  |  |
| Bischofia javanica | 110.0557 | 19.58413 |  |  |
| Bischofia javanica | 109.59 | 19.17149 |  |  |
| Bischofia javanica | 110.4829 | 19.56087 |  |  |
| Bischofia javanica | 109.7878 | 19.74477 |  |  |
| Bischofia javanica | 109.6484 | 19.57263 |  |  |
| Blachia siamensis | 109.5106 | 18.24137 | 25337.66 | 201585.1 |
| Blachia siamensis | 109.5186 | 18.25822 | 26241.73 | 203424.4 |
| Blachia siamensis | 109.6538 | 18.29652 | 40638.73 | 207266.3 |
| Blachia siamensis | 109.9239 | 19.14611 |  |  |
| Blachia siamensis | 108.9641 | 19.21535 |  |  |
| Blachia siamensis | 109.2022 | 19.14548 |  |  |
| Blachia siamensis | 110.0226 | 18.66423 |  |  |
| Blachia siamensis | 109.5601 | 19.12103 |  |  |
| Blachia siamensis | 109.5959 | 19.10196 |  |  |
| Blachia siamensis | 109.0279 | 18.73232 |  |  |
| Blachia siamensis | 109.1164 | 18.67684 |  |  |
| Blachia siamensis | 109.347 | 19.05055 |  |  |
| Blachia siamensis | 109.8884 | 18.68363 |  |  |
| Blachia siamensis | 109.0467 | 18.99324 |  |  |
| Blachia siamensis | 110.4659 | 19.27352 |  |  |
| Blachia siamensis | 109.6773 | 19.27519 |  |  |
| Blachia siamensis | 109.6585 | 19.81846 |  |  |
| Blachia siamensis | 110.5035 | 19.21578 |  |  |
| Blachia siamensis | 110.498 | 19.62469 |  |  |
| Blachia siamensis | 110.1118 | 19.32934 |  |  |
| Blachia siamensis | 109.9934 | 19.19338 |  |  |
| Blachia siamensis | 109.6521 | 19.64346 |  |  |
| Blachia siamensis | 109.7029 | 19.31639 |  |  |
| Blastus borneensis | 109.0333 | 18.86036 | -22982.8 | 271527.7 |
| Blastus borneensis | 109.3011 | 18.85155 | 5180.119 | 269701.6 |
| Blastus borneensis | 110.4321 | 19.37563 | 125548.1 | 324570.5 |
| Blastus borneensis | 110.4321 | 19.37563 | 125548.2 | 324570.6 |
| Blastus borneensis | 110.4321 | 19.37563 | 125548.3 | 324570.7 |
| Blastus borneensis | 108.7367 | 19.05266 |  |  |
| Blastus borneensis | 108.7337 | 19.04094 |  |  |
| Blastus borneensis | 108.7841 | 18.98109 |  |  |
| Blastus borneensis | 108.802 | 18.98659 |  |  |
| Blastus borneensis | 108.7819 | 18.96379 |  |  |
| Blastus borneensis | 108.7473 | 19.06463 |  |  |
| Blastus borneensis | 108.7469 | 19.06608 |  |  |
| Blastus borneensis | 108.7438 | 19.06365 |  |  |
| Blastus borneensis | 108.7431 | 19.06569 |  |  |
| Blastus borneensis | 108.7451 | 19.0655 |  |  |
| Blastus borneensis | 109.6489 | 18.25685 |  |  |
| Blastus borneensis | 109.6566 | 18.25067 |  |  |
| Blastus borneensis | 109.6573 | 18.25479 |  |  |
| Blastus borneensis | 109.5929 | 18.22399 |  |  |
| Blastus borneensis | 109.5964 | 18.2216 |  |  |
| Blastus borneensis | 109.5953 | 18.22551 |  |  |
| Blastus borneensis | 109.5912 | 18.22575 |  |  |
| Blastus borneensis | 109.6013 | 18.26003 |  |  |
| Blastus borneensis | 109.597 | 18.25601 |  |  |
| Blastus borneensis | 109.5956 | 18.2588 |  |  |
| Blastus cochinchinensis | 109.0333 | 18.86036 | -22982.8 | 271527.7 |
| Blastus cochinchinensis | 109.1191 | 19.05985 | -13271 | 293304.6 |
| Blastus cochinchinensis | 109.3011 | 18.85155 | 5180.119 | 269701.6 |
| Blastus cochinchinensis | 109.4914 | 19.41113 | 26959.92 | 330989.8 |
| Blastus cochinchinensis | 109.5146 | 18.9427 | 27935.88 | 279133.3 |
| Blastus cochinchinensis | 109.6131 | 18.44069 | 36776.91 | 223331 |
| Blastus cochinchinensis | 109.6964 | 19.03697 | 47343.87 | 289030.5 |
| Blastus cochinchinensis | 109.7575 | 19.47974 | 55069.1 | 337810.7 |
| Blastus cochinchinensis | 109.8188 | 19.14345 | 60528.46 | 300462.2 |
| Blastus cochinchinensis | 110.2432 | 19.60202 |  |  |
| Blastus cochinchinensis | 110.3147 | 19.66912 |  |  |
| Blastus cochinchinensis | 109.9211 | 18.94366 |  |  |
| Blastus cochinchinensis | 109.8644 | 18.94061 |  |  |
| Blastus cochinchinensis | 109.7725 | 18.56691 |  |  |
| Blastus cochinchinensis | 109.7781 | 18.56348 |  |  |
| Blastus cochinchinensis | 109.8665 | 18.60889 |  |  |
| Blastus cochinchinensis | 109.8506 | 18.60161 |  |  |
| Blastus cochinchinensis | 109.8159 | 18.57792 |  |  |
| Blastus cochinchinensis | 109.8317 | 18.57472 |  |  |
| Blastus cochinchinensis | 109.6558 | 18.24964 |  |  |
| Blastus cochinchinensis | 109.5968 | 18.25575 |  |  |
| Blastus cochinchinensis | 109.5982 | 18.22494 |  |  |
| Blastus cochinchinensis | 110.0594 | 18.53379 |  |  |
| Blastus cochinchinensis | 110.1025 | 18.54575 |  |  |
| Blastus cochinchinensis | 109.0745 | 18.76013 |  |  |
| Blastus cochinchinensis | 109.2747 | 18.7906 |  |  |
| Blastus cochinchinensis | 108.7357 | 19.05067 |  |  |
| Blastus cochinchinensis | 108.7451 | 19.06415 |  |  |
| Blastus cochinchinensis | 108.795 | 18.96261 |  |  |
| Blastus pauciflorus | 109.7575 | 19.47975 | 55069.2 | 337810.8 |
| Blastus pauciflorus | 109.7575 | 19.47975 | 55069.31 | 337810.9 |
| Blastus pauciflorus | 109.7575 | 19.47975 | 55069.42 | 337811 |
| Blastus pauciflorus | 109.7575 | 19.47975 | 55069.53 | 337811.1 |
| Blastus pauciflorus | 109.7575 | 19.47975 | 55069.64 | 337811.2 |
| Blastus pauciflorus | 109.4296 | 19.23097 |  |  |
| Blastus pauciflorus | 109.0232 | 19.21771 |  |  |
| Blastus pauciflorus | 109.3262 | 18.7153 |  |  |
| Blastus pauciflorus | 109.4094 | 18.70751 |  |  |
| Blastus pauciflorus | 108.9714 | 19.04702 |  |  |
| Blastus pauciflorus | 110.0108 | 18.76263 |  |  |
| Blastus pauciflorus | 109.3041 | 19.06629 |  |  |
| Blastus pauciflorus | 108.9196 | 18.79797 |  |  |
| Blastus pauciflorus | 109.6482 | 18.78033 |  |  |
| Blastus pauciflorus | 109.0439 | 18.94072 |  |  |
| Blastus pauciflorus | 110.1139 | 19.5921 |  |  |
| Blastus pauciflorus | 109.9356 | 19.8068 |  |  |
| Blastus pauciflorus | 109.875 | 19.7461 |  |  |
| Blastus pauciflorus | 109.9147 | 19.23524 |  |  |
| Blastus pauciflorus | 110.5136 | 19.5632 |  |  |
| Blastus pauciflorus | 109.9164 | 19.26848 |  |  |
| Blastus pauciflorus | 110.3304 | 19.72768 |  |  |
| Blastus pauciflorus | 110.0763 | 19.45444 |  |  |
| Blastus pauciflorus | 110.0532 | 19.8013 |  |  |
| Blastus pauciflorus | 110.0134 | 19.62482 |  |  |
| Boehmeria nivea | 109.0333 | 18.86036 | -22982.8 | 271527.7 |
| Boehmeria nivea | 109.0799 | 19.00987 | -17563.9 | 287906.3 |
| Boehmeria nivea | 109.1215 | 19.14164 | -12747.8 | 302339.7 |
| Boehmeria nivea | 109.416 | 18.58215 | 16423.77 | 239559.1 |
| Boehmeria nivea | 109.4718 | 19.16737 | 24145.48 | 304100.4 |
| Boehmeria nivea | 109.4865 | 19.15807 | 25651.28 | 303028.8 |
| Boehmeria nivea | 109.7426 | 19.4888 | 53537.07 | 338853.4 |
| Boehmeria nivea | 109.836 | 19.04117 | 62035.47 | 289106.2 |
| Boehmeria nivea | 110.2549 | 18.71143 | 105232.9 | 251551.4 |
| Boehmeria nivea | 110.3315 | 19.15256 | 114426.5 | 300146 |
| Boehmeria nivea | 109.8825 | 18.68961 |  |  |
| Boehmeria nivea | 109.5039 | 18.88558 |  |  |
| Boehmeria nivea | 109.6199 | 19.01802 |  |  |
| Boehmeria nivea | 109.9774 | 18.97852 |  |  |
| Boehmeria nivea | 109.2045 | 19.23241 |  |  |
| Boehmeria nivea | 110.0069 | 19.22291 |  |  |
| Boehmeria nivea | 109.4668 | 18.99534 |  |  |
| Boehmeria nivea | 109.9423 | 18.67873 |  |  |
| Boehmeria nivea | 109.2382 | 18.94601 |  |  |
| Boehmeria nivea | 109.5483 | 18.7083 |  |  |
| Boehmeria nivea | 109.4442 | 18.94316 |  |  |
| Boehmeria nivea | 110.0904 | 19.5908 |  |  |
| Boehmeria nivea | 109.86 | 19.74443 |  |  |
| Boehmeria nivea | 110.1629 | 19.69893 |  |  |
| Boehmeria nivea | 110.3188 | 19.80865 |  |  |
| Boehmeria nivea | 110.2099 | 19.82336 |  |  |
| Boehmeria nivea | 110.4839 | 19.3367 |  |  |
| Boehmeria nivea | 110.4464 | 19.81306 |  |  |
| Boehmeria nivea | 110.4113 | 19.21078 |  |  |
| Boehmeria nivea | 110.4989 | 19.67573 |  |  |
| Boehmeria pilosiuscula | 109.0544 | 19.13823 | -19805.8 | 302178.8 |
| Boehmeria pilosiuscula | 109.4273 | 19.01928 | 18993.09 | 287860.9 |
| Boehmeria pilosiuscula | 109.675 | 18.86962 | 44597.81 | 270587.7 |
| Boehmeria pilosiuscula | 109.8844 | 18.79929 | 66438.38 | 262227.7 |
| Boehmeria pilosiuscula | 110.1656 | 18.6887 | 95766.13 | 249260.8 |
| Boehmeria pilosiuscula | 110.0808 | 18.56149 |  |  |
| Boehmeria pilosiuscula | 110.0556 | 18.51427 |  |  |
| Boehmeria pilosiuscula | 110.1056 | 18.55771 |  |  |
| Boehmeria pilosiuscula | 109.7788 | 18.56984 |  |  |
| Boehmeria pilosiuscula | 109.763 | 18.56457 |  |  |
| Boehmeria pilosiuscula | 109.8567 | 18.60866 |  |  |
| Boehmeria pilosiuscula | 109.8633 | 18.59936 |  |  |
| Boehmeria pilosiuscula | 109.8266 | 18.58091 |  |  |
| Boehmeria pilosiuscula | 109.8246 | 18.57344 |  |  |
| Boehmeria pilosiuscula | 109.6817 | 19.57698 |  |  |
| Boehmeria pilosiuscula | 109.7106 | 19.47559 |  |  |
| Boehmeria pilosiuscula | 109.7586 | 19.49242 |  |  |
| Boehmeria pilosiuscula | 109.7683 | 19.42897 |  |  |
| Boehmeria pilosiuscula | 109.5623 | 19.60177 |  |  |
| Boehmeria pilosiuscula | 109.4098 | 19.64511 |  |  |
| Boehmeria pilosiuscula | 110.294 | 19.62088 |  |  |
| Boehmeria pilosiuscula | 110.3241 | 19.60735 |  |  |
| Boehmeria pilosiuscula | 110.4597 | 19.65739 |  |  |
| Boehmeria pilosiuscula | 110.4322 | 19.7214 |  |  |
| Boehmeria pilosiuscula | 110.3121 | 19.65416 |  |  |
| Bombax ceiba | 109.0333 | 18.86036 | -22982.8 | 271527.7 |
| Bombax ceiba | 109.5636 | 19.26187 |  |  |
| Bombax ceiba | 109.5024 | 18.98428 |  |  |
| Bombax ceiba | 109.3723 | 18.73229 |  |  |
| Bombax ceiba | 109.6821 | 18.87982 |  |  |
| Bombax ceiba | 109.8477 | 18.69241 |  |  |
| Bombax ceiba | 109.4853 | 19.2016 |  |  |
| Bombax ceiba | 109.6034 | 18.69704 |  |  |
| Bombax ceiba | 109.4638 | 18.7805 |  |  |
| Bombax ceiba | 109.217 | 19.08907 |  |  |
| Bombax ceiba | 109.4163 | 18.78185 |  |  |
| Bombax ceiba | 109.258 | 19.08417 |  |  |
| Bombax ceiba | 109.8966 | 19.68689 |  |  |
| Bombax ceiba | 110.4819 | 19.38954 |  |  |
| Bombax ceiba | 109.9725 | 19.62445 |  |  |
| Bombax ceiba | 110.174 | 19.3007 |  |  |
| Bombax ceiba | 110.0199 | 19.82143 |  |  |
| Bombax ceiba | 110.4682 | 19.45249 |  |  |
| Bombax ceiba | 110.0867 | 19.48584 |  |  |
| Bombax ceiba | 109.8286 | 19.56554 |  |  |
| Bombax ceiba | 109.9922 | 19.68611 |  |  |
| Bonia levigata | 109.837 | 18.73456 | 61260.82 | 255199 |
| Bonia levigata | 109.838 | 18.73456 | 61366.18 | 255196.4 |
| Bonia levigata | 109.839 | 18.73456 | 61471.55 | 255193.7 |
| Bonia levigata | 109.84 | 18.73456 | 61576.91 | 255191.1 |
| Bonia levigata | 109.841 | 18.73456 | 61682.27 | 255188.5 |
| Bonia levigata | 109.6493 | 18.25539 |  |  |
| Bonia levigata | 109.6588 | 18.25367 |  |  |
| Bonia levigata | 109.6536 | 18.25534 |  |  |
| Bonia levigata | 109.5913 | 18.25717 |  |  |
| Bonia levigata | 109.6002 | 18.25667 |  |  |
| Bonia levigata | 109.5942 | 18.2568 |  |  |
| Bonia levigata | 109.5974 | 18.2255 |  |  |
| Bonia levigata | 109.5974 | 18.22228 |  |  |
| Bonia levigata | 109.6003 | 18.22192 |  |  |
| Bonia levigata | 110.4368 | 18.80758 |  |  |
| Bonia levigata | 110.4291 | 18.81372 |  |  |
| Bonia levigata | 110.4381 | 18.80598 |  |  |
| Bonia levigata | 110.4262 | 18.81099 |  |  |
| Bonia levigata | 110.3754 | 18.77462 |  |  |
| Bonia levigata | 110.3724 | 18.7728 |  |  |
| Bonia levigata | 110.3772 | 18.76871 |  |  |
| Bonia levigata | 110.3736 | 18.76886 |  |  |
| Bonia levigata | 110.4028 | 18.75194 |  |  |
| Bonia levigata | 110.4303 | 18.73365 |  |  |
| Bonia levigata | 110.4222 | 18.76651 |  |  |
| Bowringia callicarpa | 109.0365 | 18.87698 | -22580.2 | 273355 |
| Bowringia callicarpa | 109.1215 | 19.14164 | -12747.8 | 302339.7 |
| Bowringia callicarpa | 109.2757 | 19.44978 | 4460.62 | 335915.6 |
| Bowringia callicarpa | 109.4865 | 19.15807 | 25651.28 | 303028.8 |
| Bowringia callicarpa | 110.0502 | 19.8863 | 86871.4 | 381966.6 |
| Bowringia callicarpa | 109.7369 | 19.19137 |  |  |
| Bowringia callicarpa | 109.7529 | 18.67506 |  |  |
| Bowringia callicarpa | 110.02 | 19.20096 |  |  |
| Bowringia callicarpa | 109.9324 | 18.99133 |  |  |
| Bowringia callicarpa | 109.8938 | 19.21861 |  |  |
| Bowringia callicarpa | 109.8006 | 18.80941 |  |  |
| Bowringia callicarpa | 109.5474 | 19.12681 |  |  |
| Bowringia callicarpa | 109.1042 | 18.97669 |  |  |
| Bowringia callicarpa | 108.9333 | 18.892 |  |  |
| Bowringia callicarpa | 109.3649 | 18.69796 |  |  |
| Bowringia callicarpa | 110.3174 | 19.78647 |  |  |
| Bowringia callicarpa | 110.0482 | 19.36057 |  |  |
| Bowringia callicarpa | 110.0783 | 19.44471 |  |  |
| Bowringia callicarpa | 110.2986 | 19.17328 |  |  |
| Bowringia callicarpa | 110.3595 | 19.20619 |  |  |
| Bowringia callicarpa | 109.9762 | 19.44301 |  |  |
| Bowringia callicarpa | 110.1308 | 19.21876 |  |  |
| Bowringia callicarpa | 109.9775 | 19.2983 |  |  |
| Bowringia callicarpa | 109.9538 | 19.68811 |  |  |
| Bowringia callicarpa | 110.0471 | 19.67504 |  |  |
| Brassaiopsis glomerulata | 108.7989 | 18.69619 | -48238.4 | 254162.2 |
| Brassaiopsis glomerulata | 109.0578 | 19.12083 | -19509.9 | 300245 |
| Brassaiopsis glomerulata | 109.3011 | 18.85155 | 5180.119 | 269701.6 |
| Brassaiopsis glomerulata | 109.3683 | 19.19189 | 13342.08 | 307122.7 |
| Brassaiopsis glomerulata | 109.777 | 19.17887 | 56233.56 | 304494 |
| Brassaiopsis glomerulata | 109.1811 | 19.10363 |  |  |
| Brassaiopsis glomerulata | 109.6267 | 18.70402 |  |  |
| Brassaiopsis glomerulata | 109.9009 | 18.86242 |  |  |
| Brassaiopsis glomerulata | 109.5322 | 18.84033 |  |  |
| Brassaiopsis glomerulata | 109.5645 | 19.0199 |  |  |
| Brassaiopsis glomerulata | 109.1661 | 19.24013 |  |  |
| Brassaiopsis glomerulata | 109.1097 | 19.16157 |  |  |
| Brassaiopsis glomerulata | 109.769 | 19.20353 |  |  |
| Brassaiopsis glomerulata | 109.3326 | 19.05663 |  |  |
| Brassaiopsis glomerulata | 109.5255 | 18.93483 |  |  |
| Brassaiopsis glomerulata | 109.0969 | 19.12794 |  |  |
| Brassaiopsis glomerulata | 109.7486 | 19.33633 |  |  |
| Brassaiopsis glomerulata | 109.9729 | 19.28567 |  |  |
| Brassaiopsis glomerulata | 110.2808 | 19.61577 |  |  |
| Brassaiopsis glomerulata | 110.2296 | 19.22272 |  |  |
| Brassaiopsis glomerulata | 110.2744 | 19.3372 |  |  |
| Brassaiopsis glomerulata | 110.4627 | 19.18933 |  |  |
| Brassaiopsis glomerulata | 110.3928 | 19.33554 |  |  |
| Brassaiopsis glomerulata | 109.7006 | 19.25639 |  |  |
| Brassaiopsis glomerulata | 110.1772 | 19.82194 |  |  |
| Bretschneidera sinensis | 109.4273 | 19.01928 | 18993.09 | 287860.9 |
| Bretschneidera sinensis | 109.4273 | 19.01928 | 18993.2 | 287861 |
| Bretschneidera sinensis | 109.4273 | 19.01928 | 18993.31 | 287861.2 |
| Bretschneidera sinensis | 109.4273 | 19.01928 | 18993.42 | 287861.3 |
| Bretschneidera sinensis | 109.4273 | 19.01928 | 18993.53 | 287861.4 |
| Bretschneidera sinensis | 109.7431 | 18.7905 |  |  |
| Bretschneidera sinensis | 109.8717 | 19.21329 |  |  |
| Bretschneidera sinensis | 109.304 | 18.86631 |  |  |
| Bretschneidera sinensis | 109.9198 | 19.22254 |  |  |
| Bretschneidera sinensis | 109.8413 | 19.14214 |  |  |
| Bretschneidera sinensis | 109.629 | 18.74736 |  |  |
| Bretschneidera sinensis | 109.7043 | 19.11433 |  |  |
| Bretschneidera sinensis | 109.6822 | 18.7823 |  |  |
| Bretschneidera sinensis | 109.534 | 19.24088 |  |  |
| Bretschneidera sinensis | 109.5903 | 18.96776 |  |  |
| Bretschneidera sinensis | 109.7302 | 19.06011 |  |  |
| Bretschneidera sinensis | 110.0413 | 19.81733 |  |  |
| Bretschneidera sinensis | 110.134 | 19.42339 |  |  |
| Bretschneidera sinensis | 109.6847 | 19.25059 |  |  |
| Bretschneidera sinensis | 110.011 | 19.55881 |  |  |
| Bretschneidera sinensis | 110.4176 | 19.43034 |  |  |
| Bretschneidera sinensis | 109.5882 | 19.48195 |  |  |
| Bretschneidera sinensis | 109.8261 | 19.48847 |  |  |
| Bretschneidera sinensis | 110.3482 | 19.67538 |  |  |
| Bretschneidera sinensis | 109.8441 | 19.61181 |  |  |
| Breynia fruticosa | 108.9122 | 18.99917 | -35232.9 | 287275.8 |
| Breynia fruticosa | 108.9515 | 18.90932 | -31419.3 | 277210.7 |
| Breynia fruticosa | 109.0333 | 18.86036 | -22982.8 | 271527.7 |
| Breynia fruticosa | 109.1191 | 19.05985 | -13271 | 293304.6 |
| Breynia fruticosa | 109.4865 | 19.15807 | 25651.28 | 303028.8 |
| Breynia fruticosa | 109.5132 | 19.56355 | 29719.98 | 347774.6 |
| Breynia fruticosa | 109.5146 | 18.9427 | 27935.88 | 279133.3 |
| Breynia fruticosa | 109.6807 | 18.37829 | 43729.57 | 216234.2 |
| Breynia fruticosa | 109.7575 | 19.47974 | 55069.1 | 337810.7 |
| Breynia fruticosa | 109.8844 | 18.79929 | 66438.38 | 262227.7 |
| Breynia fruticosa | 110.1756 | 18.76435 | 97019.43 | 257602 |
| Breynia fruticosa | 110.1876 | 19.13499 | 99259.69 | 298557.2 |
| Breynia fruticosa | 110.0852 | 18.54988 |  |  |
| Breynia fruticosa | 110.0638 | 18.51329 |  |  |
| Breynia fruticosa | 110.0982 | 18.51843 |  |  |
| Breynia fruticosa | 109.055 | 18.7571 |  |  |
| Breynia fruticosa | 108.9957 | 18.72828 |  |  |
| Breynia fruticosa | 109.0794 | 18.56605 |  |  |
| Breynia fruticosa | 109.128 | 18.53907 |  |  |
| Breynia fruticosa | 109.1339 | 18.54285 |  |  |
| Breynia fruticosa | 109.2937 | 18.78645 |  |  |
| Breynia fruticosa | 109.2805 | 18.79292 |  |  |
| Breynia fruticosa | 109.2985 | 18.80213 |  |  |
| Breynia fruticosa | 109.6504 | 18.25142 |  |  |
| Breynia fruticosa | 109.6554 | 18.25622 |  |  |
| Breynia fruticosa | 109.5988 | 18.22358 |  |  |
| Breynia fruticosa | 109.5931 | 18.2233 |  |  |
| Breynia fruticosa | 109.5968 | 18.25613 |  |  |
| Breynia fruticosa | 109.5968 | 18.2605 |  |  |
| Breynia fruticosa | 109.6738 | 18.84846 |  |  |
| Breynia fruticosa | 109.6958 | 18.85875 |  |  |
| Breynia fruticosa | 109.6812 | 18.83556 |  |  |
| Bridelia balansae | 109.0799 | 19.00987 | -17563.9 | 287906.3 |
| Bridelia balansae | 109.3011 | 18.85155 | 5180.119 | 269701.6 |
| Bridelia balansae | 110.288 | 18.85363 | 109080.9 | 267196.6 |
| Bridelia balansae | 109.4207 | 18.684 |  |  |
| Bridelia balansae | 109.4526 | 18.84387 |  |  |
| Bridelia balansae | 109.8359 | 19.16876 |  |  |
| Bridelia balansae | 109.7822 | 18.70257 |  |  |
| Bridelia balansae | 109.4568 | 18.86508 |  |  |
| Bridelia balansae | 109.6388 | 18.94097 |  |  |
| Bridelia balansae | 109.9965 | 18.66171 |  |  |
| Bridelia balansae | 109.5296 | 18.67718 |  |  |
| Bridelia balansae | 109.3937 | 19.2199 |  |  |
| Bridelia balansae | 109.2431 | 19.10173 |  |  |
| Bridelia balansae | 109.2523 | 19.1407 |  |  |
| Bridelia balansae | 109.0917 | 18.98995 |  |  |
| Bridelia balansae | 109.7397 | 19.18476 |  |  |
| Bridelia balansae | 109.9038 | 19.28357 |  |  |
| Bridelia balansae | 110.4379 | 19.54499 |  |  |
| Bridelia balansae | 110.264 | 19.76581 |  |  |
| Bridelia balansae | 110.0827 | 19.66646 |  |  |
| Bridelia balansae | 109.8628 | 19.53714 |  |  |
| Bridelia balansae | 110.4818 | 19.75688 |  |  |
| Bridelia balansae | 110.0218 | 19.75899 |  |  |
| Bridelia stipularis | 108.9122 | 18.99917 | -35232.9 | 287275.8 |
| Bridelia stipularis | 109.0799 | 19.00987 | -17563.9 | 287906.3 |
| Bridelia stipularis | 109.1041 | 19.11352 | -14673.8 | 299287.2 |
| Bridelia stipularis | 109.3011 | 18.85155 | 5180.119 | 269701.6 |
| Bridelia stipularis | 109.6975 | 18.72833 | 46544.54 | 254900 |
| Bridelia stipularis | 109.7022 | 19.5415 |  |  |
| Bridelia stipularis | 109.6238 | 19.5372 |  |  |
| Bridelia stipularis | 109.7012 | 19.50203 |  |  |
| Bridelia stipularis | 109.5046 | 19.45422 |  |  |
| Bridelia stipularis | 109.4215 | 19.58237 |  |  |
| Bridelia stipularis | 109.4057 | 19.63993 |  |  |
| Bridelia stipularis | 109.3453 | 19.62441 |  |  |
| Bridelia stipularis | 108.737 | 19.04023 |  |  |
| Bridelia stipularis | 108.7437 | 19.0478 |  |  |
| Bridelia stipularis | 108.8068 | 18.95186 |  |  |
| Bridelia stipularis | 108.7886 | 18.98678 |  |  |
| Bridelia stipularis | 108.7441 | 19.06563 |  |  |
| Bridelia stipularis | 108.7442 | 19.06681 |  |  |
| Bridelia stipularis | 108.7439 | 19.0649 |  |  |
| Bridelia stipularis | 109.0799 | 18.67459 |  |  |
| Bridelia stipularis | 109.0108 | 18.68841 |  |  |
| Bridelia stipularis | 109.0609 | 18.56493 |  |  |
| Bridelia stipularis | 109.0639 | 18.60634 |  |  |
| Bridelia stipularis | 109.2968 | 18.78636 |  |  |
| Bridelia stipularis | 109.2886 | 18.8038 |  |  |
| Bridelia tomentosa | 108.6488 | 19.11618 | -62506.4 | 301115.3 |
| Bridelia tomentosa | 109.4548 | 19.18904 | 22421.09 | 306546.7 |
| Bridelia tomentosa | 109.5153 | 18.61925 | 27012 | 243364.4 |
| Bridelia tomentosa | 109.7575 | 19.47974 | 55069.1 | 337810.7 |
| Bridelia tomentosa | 109.9046 | 19.17419 |  |  |
| Bridelia tomentosa | 109.1657 | 18.67259 |  |  |
| Bridelia tomentosa | 109.7876 | 18.81612 |  |  |
| Bridelia tomentosa | 109.2696 | 19.01207 |  |  |
| Bridelia tomentosa | 109.7284 | 19.2255 |  |  |
| Bridelia tomentosa | 109.0891 | 18.90761 |  |  |
| Bridelia tomentosa | 109.4667 | 19.19693 |  |  |
| Bridelia tomentosa | 109.0789 | 19.14117 |  |  |
| Bridelia tomentosa | 108.9602 | 19.17338 |  |  |
| Bridelia tomentosa | 109.8965 | 18.79027 |  |  |
| Bridelia tomentosa | 109.8996 | 18.80907 |  |  |
| Bridelia tomentosa | 109.901 | 19.28575 |  |  |
| Bridelia tomentosa | 109.5904 | 19.77716 |  |  |
| Bridelia tomentosa | 110.2348 | 19.39532 |  |  |
| Bridelia tomentosa | 110.4603 | 19.19437 |  |  |
| Bridelia tomentosa | 110.1523 | 19.35759 |  |  |
| Bridelia tomentosa | 110.2047 | 19.20178 |  |  |
| Bridelia tomentosa | 110.2145 | 19.80184 |  |  |
| Bridelia tomentosa | 110.4569 | 19.36138 |  |  |
| Bridelia tomentosa | 109.5762 | 19.55958 |  |  |
| Broussonetia kaempferi | 109.2343 | 18.80064 | -2022.54 | 264280.3 |
| Broussonetia kaempferi | 109.3011 | 18.85155 | 5180.119 | 269701.6 |
| Broussonetia kaempferi | 109.3683 | 19.19189 | 13342.08 | 307122.7 |
| Broussonetia kaempferi | 109.7575 | 19.47974 | 55069.1 | 337810.7 |
| Broussonetia kaempferi | 109.8855 | 18.96521 | 67022.55 | 280572.5 |
| Broussonetia kaempferi | 110.0165 | 19.72488 |  |  |
| Broussonetia kaempferi | 109.9872 | 19.71207 |  |  |
| Broussonetia kaempferi | 110.0483 | 19.74528 |  |  |
| Broussonetia kaempferi | 110.0668 | 19.77299 |  |  |
| Broussonetia kaempferi | 110.0508 | 19.81111 |  |  |
| Broussonetia kaempferi | 110.2108 | 19.63905 |  |  |
| Broussonetia kaempferi | 110.2888 | 19.60193 |  |  |
| Broussonetia kaempferi | 110.2766 | 19.67586 |  |  |
| Broussonetia kaempferi | 110.293 | 19.61916 |  |  |
| Broussonetia kaempferi | 110.269 | 19.5283 |  |  |
| Broussonetia kaempferi | 109.4702 | 19.21852 |  |  |
| Broussonetia kaempferi | 109.466 | 19.21816 |  |  |
| Broussonetia kaempferi | 109.3534 | 19.21079 |  |  |
| Broussonetia kaempferi | 109.3506 | 19.21896 |  |  |
| Broussonetia kaempferi | 109.3913 | 19.2308 |  |  |
| Broussonetia kaempferi | 109.3943 | 19.23113 |  |  |
| Broussonetia kaempferi | 109.3926 | 19.23034 |  |  |
| Broussonetia kaempferi | 110.1014 | 18.52098 |  |  |
| Broussonetia kaempferi | 110.0901 | 18.5168 |  |  |
| Broussonetia kaempferi | 110.0841 | 18.55552 |  |  |
| Broussonetia papyrifera | 108.7989 | 18.69619 | -48238.4 | 254162.2 |
| Broussonetia papyrifera | 109.0333 | 18.86036 | -22982.8 | 271527.7 |
| Broussonetia papyrifera | 109.1191 | 19.05985 | -13271 | 293304.6 |
| Broussonetia papyrifera | 109.1741 | 18.37558 | -9781.56 | 217466.3 |
| Broussonetia papyrifera | 109.3011 | 18.85155 | 5180.119 | 269701.6 |
| Broussonetia papyrifera | 109.4865 | 19.15807 | 25651.28 | 303028.8 |
| Broussonetia papyrifera | 109.654 | 19.43482 | 44083.3 | 333136.1 |
| Broussonetia papyrifera | 109.7345 | 18.74207 | 50478.48 | 256315 |
| Broussonetia papyrifera | 109.8544 | 19.05325 | 64007.07 | 290391.5 |
| Broussonetia papyrifera | 109.6759 | 19.4985 |  |  |
| Broussonetia papyrifera | 109.716 | 19.46083 |  |  |
| Broussonetia papyrifera | 109.6253 | 19.54786 |  |  |
| Broussonetia papyrifera | 109.0343 | 19.30904 |  |  |
| Broussonetia papyrifera | 109.0375 | 19.30142 |  |  |
| Broussonetia papyrifera | 109.0167 | 19.27721 |  |  |
| Broussonetia papyrifera | 109.078 | 19.35405 |  |  |
| Broussonetia papyrifera | 109.1654 | 19.30253 |  |  |
| Broussonetia papyrifera | 109.0908 | 19.34054 |  |  |
| Broussonetia papyrifera | 109.4691 | 19.22008 |  |  |
| Broussonetia papyrifera | 109.3872 | 19.23559 |  |  |
| Broussonetia papyrifera | 109.3494 | 19.21837 |  |  |
| Broussonetia papyrifera | 108.7456 | 19.04317 |  |  |
| Broussonetia papyrifera | 108.7439 | 19.06451 |  |  |
| Broussonetia papyrifera | 108.7946 | 18.97499 |  |  |
| Broussonetia papyrifera | 109.6512 | 18.25506 |  |  |
| Broussonetia papyrifera | 109.6587 | 18.2555 |  |  |
| Broussonetia papyrifera | 109.5908 | 18.22667 |  |  |
| Broussonetia papyrifera | 109.5987 | 18.25771 |  |  |
| Broussonetia papyrifera | 109.5985 | 18.26 |  |  |
| Brucea javanica | 109.0367 | 19.17511 | -21544.3 | 306313.7 |
| Brucea javanica | 109.1215 | 19.14164 | -12747.8 | 302339.7 |
| Brucea javanica | 109.4865 | 19.15807 | 25651.28 | 303028.8 |
| Brucea javanica | 109.6365 | 19.44697 | 42283.97 | 334529.2 |
| Brucea javanica | 109.7575 | 19.47974 | 55069.1 | 337810.7 |
| Brucea javanica | 109.9218 | 19.14374 |  |  |
| Brucea javanica | 109.7087 | 19.09316 |  |  |
| Brucea javanica | 109.19 | 18.90645 |  |  |
| Brucea javanica | 109.9467 | 18.82884 |  |  |
| Brucea javanica | 109.0864 | 18.70674 |  |  |
| Brucea javanica | 108.9708 | 19.07931 |  |  |
| Brucea javanica | 109.8902 | 18.66919 |  |  |
| Brucea javanica | 109.0799 | 18.78534 |  |  |
| Brucea javanica | 108.9651 | 18.75218 |  |  |
| Brucea javanica | 109.1131 | 19.14393 |  |  |
| Brucea javanica | 109.9287 | 19.78462 |  |  |
| Brucea javanica | 109.7934 | 19.16448 |  |  |
| Brucea javanica | 110.052 | 19.36127 |  |  |
| Brucea javanica | 109.6881 | 19.56402 |  |  |
| Brucea javanica | 110.0522 | 19.56053 |  |  |
| Brucea javanica | 110.2006 | 19.19661 |  |  |
| Brucea javanica | 110.3834 | 19.30135 |  |  |
| Brucea javanica | 110.3891 | 19.53358 |  |  |
| Brucea javanica | 109.8151 | 19.62566 |  |  |
| Brucea javanica | 109.6994 | 19.46733 |  |  |
| Bruguiera gymnorhiza | 110.0822 | 19.53483 | 89270.66 | 343031.3 |
| Bruguiera sexangula | 109.7111 | 18.26491 | 46605.9 | 203606.1 |
| Bruguiera sexangula | 110.2332 | 18.6893 | 102892.4 | 249157.1 |
| Bruguiera sexangula | 110.2738 | 18.67292 | 107120.4 | 247246.1 |
| Bruguiera sexangula | 110.577 | 19.95677 | 142122.3 | 388485.7 |
| Bruguiera sexangula | 110.6146 | 19.99826 | 146153.1 | 392988.4 |
| Bruguiera sexangula | 110.2151 | 20.01213 |  |  |
| Bruguiera sexangula | 110.2158 | 20.01172 |  |  |
| Bruguiera sexangula | 110.414 | 19.85615 |  |  |
| Bruguiera sexangula | 110.4168 | 19.85848 |  |  |
| Bruguiera sexangula | 110.4643 | 19.85045 |  |  |
| Bruguiera sexangula | 110.465 | 19.84881 |  |  |
| Bruguiera sexangula | 110.8699 | 19.62917 |  |  |
| Bruguiera sexangula | 110.9251 | 19.67809 |  |  |
| Bruguiera sexangula | 110.742 | 19.62086 |  |  |
| Bruguiera sexangula | 110.7001 | 19.52853 |  |  |
| Bruguiera sexangula | 110.0751 | 18.54494 |  |  |
| Bruguiera sexangula | 110.0827 | 18.55752 |  |  |
| Bruguiera sexangula | 110.0914 | 18.55866 |  |  |
| Bruguiera sexangula | 109.6487 | 18.2528 |  |  |
| Bruguiera sexangula | 109.6504 | 18.2552 |  |  |
| Bruguiera sexangula | 109.5912 | 18.22221 |  |  |
| Bruguiera sexangula | 109.5959 | 18.22435 |  |  |
| Bruguiera sexangula | 109.6013 | 18.2586 |  |  |
| Bruguiera sexangula | 109.5937 | 18.25995 |  |  |
| Bruguiera sexangula | 109.5921 | 18.2566 |  |  |
| Buchanania arborescens | 109.1191 | 19.05985 | -13271 | 293304.6 |
| Buchanania arborescens | 109.1741 | 18.37558 | -9781.56 | 217466.3 |
| Buchanania arborescens | 109.3011 | 18.85155 | 5180.119 | 269701.6 |
| Buchanania arborescens | 109.6271 | 19.59807 | 41761.54 | 351259.2 |
| Buchanania arborescens | 109.869 | 18.69141 | 64504.44 | 250339.5 |
| Buchanania arborescens | 109.6611 | 19.4393 |  |  |
| Buchanania arborescens | 109.5793 | 19.47264 |  |  |
| Buchanania arborescens | 109.6795 | 19.52269 |  |  |
| Buchanania arborescens | 109.0143 | 19.28692 |  |  |
| Buchanania arborescens | 109.0276 | 19.30324 |  |  |
| Buchanania arborescens | 109.0346 | 19.27818 |  |  |
| Buchanania arborescens | 109.1435 | 19.3255 |  |  |
| Buchanania arborescens | 109.1484 | 19.32385 |  |  |
| Buchanania arborescens | 109.4679 | 19.21984 |  |  |
| Buchanania arborescens | 109.3917 | 19.22975 |  |  |
| Buchanania arborescens | 109.3504 | 19.20986 |  |  |
| Buchanania arborescens | 108.7462 | 19.04393 |  |  |
| Buchanania arborescens | 108.7471 | 19.06601 |  |  |
| Buchanania arborescens | 108.7905 | 18.98123 |  |  |
| Buchanania arborescens | 109.0134 | 18.72788 |  |  |
| Buchanania arborescens | 109.2767 | 18.78393 |  |  |
| Buchanania arborescens | 109.0403 | 18.52479 |  |  |
| Buchanania arborescens | 109.7704 | 18.57206 |  |  |
| Buchanania arborescens | 109.8275 | 18.57995 |  |  |
| Buchanania arborescens | 109.87 | 18.6035 |  |  |
| Buddleja asiatica | 109.0333 | 18.86036 | -22982.8 | 271527.7 |
| Buddleja asiatica | 109.0367 | 19.17511 | -21544.3 | 306313.7 |
| Buddleja asiatica | 109.1191 | 19.05985 | -13271 | 293304.6 |
| Buddleja asiatica | 109.2343 | 18.80064 | -2022.54 | 264280.3 |
| Buddleja asiatica | 109.3011 | 18.85155 | 5180.119 | 269701.6 |
| Buddleja asiatica | 109.4546 | 19.30628 | 22766.04 | 319508.9 |
| Buddleja asiatica | 109.675 | 18.86962 | 44597.81 | 270587.7 |
| Buddleja asiatica | 109.6964 | 19.03697 | 47343.87 | 289030.5 |
| Buddleja asiatica | 109.7097 | 19.53075 | 50211.1 | 343582.5 |
| Buddleja asiatica | 109.8073 | 18.71975 | 58090.63 | 253642.9 |
| Buddleja asiatica | 109.836 | 19.04117 | 62035.47 | 289106.2 |
| Buddleja asiatica | 110.0914 | 18.76565 | 88154.38 | 257960.3 |
| Buddleja asiatica | 110.2238 | 18.67552 | 101866.1 | 247657.4 |
| Buddleja asiatica | 109.6842 | 19.95396 |  |  |
| Buddleja asiatica | 109.7622 | 19.88441 |  |  |
| Buddleja asiatica | 109.6287 | 19.8766 |  |  |
| Buddleja asiatica | 110.2241 | 19.53828 |  |  |
| Buddleja asiatica | 110.2572 | 19.67728 |  |  |
| Buddleja asiatica | 110.2482 | 19.6406 |  |  |
| Buddleja asiatica | 109.8301 | 19.00054 |  |  |
| Buddleja asiatica | 109.7452 | 19.05498 |  |  |
| Buddleja asiatica | 109.7932 | 19.01876 |  |  |
| Buddleja asiatica | 110.0951 | 18.54812 |  |  |
| Buddleja asiatica | 110.066 | 18.53081 |  |  |
| Buddleja asiatica | 110.1012 | 18.52114 |  |  |
| Buddleja asiatica | 109.7709 | 18.56548 |  |  |
| Buddleja asiatica | 109.7732 | 18.57331 |  |  |
| Buddleja asiatica | 109.8636 | 18.60078 |  |  |
| Buddleja asiatica | 109.858 | 18.59788 |  |  |
| Buddleja asiatica | 109.8302 | 18.58376 |  |  |
| Buddleja asiatica | 109.8328 | 18.58697 |  |  |
| Buddleja asiatica | 109.9365 | 18.57915 |  |  |
| Buddleja asiatica | 110.01 | 18.68911 |  |  |
| Buddleja lindleyana | 109.1544 | 19.01211 | -9728.08 | 287914.8 |
| Buddleja lindleyana | 109.1544 | 19.01212 | -9727.97 | 287914.9 |
| Buddleja lindleyana | 109.1544 | 19.01212 | -9727.86 | 287915 |
| Buddleja lindleyana | 109.416 | 18.58215 | 16423.77 | 239559.1 |
| Buddleja lindleyana | 109.5153 | 18.61925 | 27012 | 243364.4 |
| Buddleja lindleyana | 109.7827 | 18.56812 |  |  |
| Buddleja lindleyana | 109.7655 | 18.57333 |  |  |
| Buddleja lindleyana | 109.8592 | 18.59629 |  |  |
| Buddleja lindleyana | 109.8628 | 18.60517 |  |  |
| Buddleja lindleyana | 109.8194 | 18.5854 |  |  |
| Buddleja lindleyana | 109.8256 | 18.57947 |  |  |
| Buddleja lindleyana | 108.9887 | 19.2766 |  |  |
| Buddleja lindleyana | 109.0232 | 19.27567 |  |  |
| Buddleja lindleyana | 109.1177 | 19.31908 |  |  |
| Buddleja lindleyana | 109.0676 | 19.35321 |  |  |
| Buddleja lindleyana | 109.714 | 18.70277 |  |  |
| Buddleja lindleyana | 109.7792 | 18.69757 |  |  |
| Buddleja lindleyana | 109.8802 | 18.69496 |  |  |
| Buddleja lindleyana | 109.9221 | 18.66374 |  |  |
| Buddleja lindleyana | 109.784 | 18.61039 |  |  |
| Buddleja lindleyana | 109.7765 | 18.52838 |  |  |
| Buddleja lindleyana | 109.8747 | 18.51405 |  |  |
| Buddleja lindleyana | 109.151 | 19.30492 |  |  |
| Buddleja lindleyana | 109.2395 | 19.23038 |  |  |
| Buddleja lindleyana | 108.8859 | 19.12467 |  |  |
| Buxus sinica | 109.0333 | 18.86036 | -22982.8 | 271527.7 |
| Buxus sinica | 109.0799 | 19.00987 | -17563.9 | 287906.3 |
| Buxus sinica | 109.8628 | 18.65961 | 63764.9 | 246839.6 |
| Buxus sinica | 109.7757 | 18.56278 |  |  |
| Buxus sinica | 109.784 | 18.56243 |  |  |
| Buxus sinica | 109.7784 | 18.57044 |  |  |
| Buxus sinica | 109.7751 | 18.57361 |  |  |
| Buxus sinica | 109.8197 | 18.57944 |  |  |
| Buxus sinica | 109.8291 | 18.58232 |  |  |
| Buxus sinica | 109.8326 | 18.58385 |  |  |
| Buxus sinica | 109.8268 | 18.5817 |  |  |
| Buxus sinica | 109.8573 | 18.60817 |  |  |
| Buxus sinica | 109.8502 | 18.60242 |  |  |
| Buxus sinica | 109.8717 | 18.60356 |  |  |
| Buxus sinica | 109.8494 | 18.6072 |  |  |
| Buxus sinica | 109.7157 | 18.70537 |  |  |
| Buxus sinica | 109.6203 | 18.68033 |  |  |
| Buxus sinica | 109.6196 | 18.71545 |  |  |
| Buxus sinica | 109.6611 | 18.72456 |  |  |
| Buxus sinica | 109.7044 | 18.74797 |  |  |
| Buxus sinica | 109.7724 | 18.56646 |  |  |
| Buxus sinica | 109.7803 | 18.60941 |  |  |
| Buxus sinica | 109.8283 | 18.58273 |  |  |
| Caesalpinia bonduc | 108.6526 | 18.84592 | -63101 | 271225.5 |
| Caesalpinia bonduc | 109.0333 | 18.86036 | -22982.8 | 271527.7 |
| Caesalpinia bonduc | 109.1741 | 18.37558 | -9781.56 | 217466.3 |
| Caesalpinia bonduc | 110.2332 | 18.6893 | 102892.4 | 249157.1 |
| Caesalpinia bonduc | 110.6819 | 19.92734 | 153022.5 | 385001.2 |
| Caesalpinia bonduc | 109.0136 | 18.70609 |  |  |
| Caesalpinia bonduc | 109.2824 | 18.78537 |  |  |
| Caesalpinia bonduc | 109.0822 | 18.57799 |  |  |
| Caesalpinia bonduc | 109.6489 | 18.25167 |  |  |
| Caesalpinia bonduc | 109.5929 | 18.25913 |  |  |
| Caesalpinia bonduc | 109.5982 | 18.22397 |  |  |
| Caesalpinia bonduc | 109.0199 | 19.28834 |  |  |
| Caesalpinia bonduc | 109.0872 | 19.31219 |  |  |
| Caesalpinia bonduc | 109.1677 | 19.30283 |  |  |
| Caesalpinia bonduc | 109.109 | 19.30885 |  |  |
| Caesalpinia bonduc | 109.1174 | 19.3318 |  |  |
| Caesalpinia bonduc | 110.0907 | 18.56073 |  |  |
| Caesalpinia bonduc | 110.0729 | 18.52678 |  |  |
| Caesalpinia bonduc | 110.0885 | 18.51692 |  |  |
| Caesalpinia bonduc | 110.0687 | 18.55135 |  |  |
| Caesalpinia bonduc | 110.9259 | 19.71792 |  |  |
| Caesalpinia bonduc | 110.7702 | 19.55134 |  |  |
| Caesalpinia bonduc | 110.6495 | 19.62906 |  |  |
| Caesalpinia bonduc | 110.7231 | 19.51519 |  |  |
| Caesalpinia bonduc | 110.7751 | 19.44398 |  |  |
| Caesalpinia crista | 109.0333 | 18.86036 | -22982.8 | 271527.7 |
| Caesalpinia crista | 109.6807 | 18.37829 | 43729.57 | 216234.2 |
| Caesalpinia crista | 109.8543 | 19.93082 | 66517.9 | 387406.2 |
| Caesalpinia crista | 110.2163 | 19.94128 | 104388.5 | 387624 |
| Caesalpinia crista | 110.3897 | 18.85302 | 119795.1 | 266882.9 |
| Caesalpinia crista | 110.4708 | 19.26302 | 129336.6 | 312030.1 |
| Caesalpinia crista | 110.4308 | 18.79528 |  |  |
| Caesalpinia crista | 110.382 | 18.77627 |  |  |
| Caesalpinia crista | 110.416 | 18.74389 |  |  |
| Caesalpinia crista | 110.2153 | 20.01223 |  |  |
| Caesalpinia crista | 110.2155 | 20.0116 |  |  |
| Caesalpinia crista | 110.2153 | 20.01174 |  |  |
| Caesalpinia crista | 110.2155 | 20.01174 |  |  |
| Caesalpinia crista | 110.2151 | 20.01206 |  |  |
| Caesalpinia crista | 108.7331 | 19.04826 |  |  |
| Caesalpinia crista | 108.745 | 19.06604 |  |  |
| Caesalpinia crista | 108.7893 | 18.98355 |  |  |
| Caesalpinia crista | 109.0266 | 18.68858 |  |  |
| Caesalpinia crista | 109.2788 | 18.79852 |  |  |
| Caesalpinia crista | 109.1375 | 18.53876 |  |  |
| Caesalpinia crista | 109.0917 | 18.59622 |  |  |
| Caesalpinia crista | 109.6536 | 18.25785 |  |  |
| Caesalpinia crista | 109.5932 | 18.26287 |  |  |
| Caesalpinia crista | 109.5922 | 18.2227 |  |  |
| Caesalpinia crista | 109.6001 | 18.2221 |  |  |
| Caesalpinia crista | 109.5934 | 18.2257 |  |  |
| Caesalpinia decapetala | 109.3163 | 18.42284 | 5389.832 | 222245.7 |
| Caesalpinia decapetala | 109.3163 | 18.42284 | 5389.94 | 222245.8 |
| Caesalpinia decapetala | 109.3163 | 18.42284 | 5390.049 | 222245.9 |
| Caesalpinia decapetala | 109.3163 | 18.42284 | 5390.158 | 222246 |
| Caesalpinia decapetala | 109.6883 | 19.91302 | 49106.79 | 385899 |
| Caesalpinia decapetala | 109.6508 | 18.25292 |  |  |
| Caesalpinia decapetala | 109.6492 | 18.2533 |  |  |
| Caesalpinia decapetala | 109.6498 | 18.25446 |  |  |
| Caesalpinia decapetala | 109.6508 | 18.25057 |  |  |
| Caesalpinia decapetala | 109.6591 | 18.25163 |  |  |
| Caesalpinia decapetala | 109.6548 | 18.25305 |  |  |
| Caesalpinia decapetala | 109.6493 | 18.2516 |  |  |
| Caesalpinia decapetala | 109.599 | 18.2577 |  |  |
| Caesalpinia decapetala | 109.5999 | 18.25822 |  |  |
| Caesalpinia decapetala | 109.5957 | 18.26124 |  |  |
| Caesalpinia decapetala | 109.5978 | 18.26255 |  |  |
| Caesalpinia decapetala | 109.599 | 18.26017 |  |  |
| Caesalpinia decapetala | 109.5976 | 18.22198 |  |  |
| Caesalpinia decapetala | 109.5953 | 18.22611 |  |  |
| Caesalpinia decapetala | 109.5971 | 18.22305 |  |  |
| Caesalpinia decapetala | 109.5947 | 18.22335 |  |  |
| Caesalpinia decapetala | 109.5937 | 18.22663 |  |  |
| Caesalpinia decapetala | 109.5936 | 18.22505 |  |  |
| Caesalpinia decapetala | 109.5932 | 18.22331 |  |  |
| Caesalpinia decapetala | 109.5912 | 18.22425 |  |  |
| Caesalpinia minax | 110.875 | 19.86071 | 173068.7 | 377227.8 |
| Caesalpinia minax | 109.5976 | 18.96264 |  |  |
| Caesalpinia minax | 109.5872 | 19.18929 |  |  |
| Caesalpinia minax | 109.4529 | 19.15924 |  |  |
| Caesalpinia minax | 109.4125 | 18.88276 |  |  |
| Caesalpinia minax | 109.3373 | 18.67572 |  |  |
| Caesalpinia minax | 109.9418 | 19.18291 |  |  |
| Caesalpinia minax | 109.8194 | 18.84031 |  |  |
| Caesalpinia minax | 109.8482 | 18.92685 |  |  |
| Caesalpinia minax | 109.3021 | 19.13406 |  |  |
| Caesalpinia minax | 110.2908 | 19.16686 |  |  |
| Caesalpinia minax | 109.9109 | 19.59006 |  |  |
| Caesalpinia minax | 109.9649 | 19.50267 |  |  |
| Caesalpinia minax | 110.4448 | 19.30755 |  |  |
| Caesalpinia minax | 109.9271 | 19.5009 |  |  |
| Caesalpinia minax | 109.8568 | 19.70769 |  |  |
| Caesalpinia minax | 110.0653 | 19.4662 |  |  |
| Caesalpinia minax | 110.063 | 19.5184 |  |  |
| Caesalpinia minax | 109.6625 | 19.52327 |  |  |
| Caesalpinia minax | 109.6039 | 19.76535 |  |  |
| Caesalpinia minax | 110.3204 | 19.84408 |  |  |
| Caesalpinia sappan | 109.0799 | 19.00987 | -17563.9 | 287906.3 |
| Caesalpinia sappan | 109.4273 | 19.01928 | 18993.09 | 287860.9 |
| Caesalpinia sappan | 109.445 | 19.2126 | 21468.7 | 309180.4 |
| Caesalpinia sappan | 109.6807 | 18.37829 | 43729.57 | 216234.2 |
| Caesalpinia sappan | 110.2125 | 18.73421 | 100828.3 | 254175.6 |
| Caesalpinia sappan | 109.4707 | 19.21729 |  |  |
| Caesalpinia sappan | 109.3869 | 19.23053 |  |  |
| Caesalpinia sappan | 109.3539 | 19.21161 |  |  |
| Caesalpinia sappan | 110.0777 | 18.51951 |  |  |
| Caesalpinia sappan | 110.0931 | 18.54196 |  |  |
| Caesalpinia sappan | 110.0968 | 18.53997 |  |  |
| Caesalpinia sappan | 110.1001 | 18.52954 |  |  |
| Caesalpinia sappan | 110.0741 | 18.55485 |  |  |
| Caesalpinia sappan | 109.6621 | 19.50699 |  |  |
| Caesalpinia sappan | 109.5822 | 19.44023 |  |  |
| Caesalpinia sappan | 109.6873 | 19.55259 |  |  |
| Caesalpinia sappan | 109.6613 | 19.5404 |  |  |
| Caesalpinia sappan | 108.9934 | 18.67312 |  |  |
| Caesalpinia sappan | 109.2828 | 18.80208 |  |  |
| Caesalpinia sappan | 109.1414 | 18.58822 |  |  |
| Caesalpinia sappan | 109.6555 | 18.25741 |  |  |
| Caesalpinia sappan | 109.5903 | 18.25753 |  |  |
| Caesalpinia sappan | 109.5972 | 18.22212 |  |  |
| Caesalpinia sappan | 109.5917 | 18.2269 |  |  |
| Caesalpinia sappan | 109.5938 | 18.22709 |  |  |
| Callerya dielsiana | 109.0799 | 19.00987 | -17563.9 | 287906.3 |
| Callerya dielsiana | 109.3233 | 18.69505 | 7006.586 | 252327.2 |
| Callerya dielsiana | 109.3233 | 18.69505 | 7006.695 | 252327.3 |
| Callerya dielsiana | 109.3233 | 18.69505 | 7006.804 | 252327.4 |
| Callerya dielsiana | 110.2256 | 19.86031 | 105147.4 | 378650.1 |
| Callerya dielsiana | 110.4147 | 19.31939 |  |  |
| Callerya dielsiana | 110.3989 | 19.31836 |  |  |
| Callerya dielsiana | 110.3703 | 19.3023 |  |  |
| Callerya dielsiana | 110.3731 | 19.3058 |  |  |
| Callerya dielsiana | 110.3985 | 19.28685 |  |  |
| Callerya dielsiana | 110.5728 | 19.24464 |  |  |
| Callerya dielsiana | 110.5642 | 19.22269 |  |  |
| Callerya dielsiana | 110.5386 | 19.24096 |  |  |
| Callerya dielsiana | 110.5812 | 19.25047 |  |  |
| Callerya dielsiana | 110.5503 | 19.22977 |  |  |
| Callerya dielsiana | 109.702 | 19.56315 |  |  |
| Callerya dielsiana | 109.5675 | 19.46759 |  |  |
| Callerya dielsiana | 109.6705 | 19.46343 |  |  |
| Callerya dielsiana | 109.7145 | 19.54713 |  |  |
| Callerya dielsiana | 109.6579 | 19.58224 |  |  |
| Callerya dielsiana | 109.568 | 19.51393 |  |  |
| Callerya dielsiana | 109.6257 | 19.52624 |  |  |
| Callerya dielsiana | 109.5932 | 19.53313 |  |  |
| Callerya dielsiana | 109.6042 | 19.45892 |  |  |
| Callerya dielsiana | 109.6414 | 19.50747 |  |  |
| Callerya nitida | 108.9648 | 19.13696 | -29229.1 | 302333.4 |
| Callerya nitida | 109.0365 | 18.87698 | -22580.2 | 273355 |
| Callerya nitida | 109.1191 | 19.05985 | -13271 | 293304.6 |
| Callerya nitida | 109.3011 | 18.85155 | 5180.119 | 269701.6 |
| Callerya nitida | 109.6807 | 18.37829 | 43729.57 | 216234.2 |
| Callerya nitida | 108.7393 | 19.04875 |  |  |
| Callerya nitida | 108.7441 | 19.06525 |  |  |
| Callerya nitida | 108.7804 | 18.95161 |  |  |
| Callerya nitida | 108.7941 | 18.9881 |  |  |
| Callerya nitida | 108.7966 | 18.97736 |  |  |
| Callerya nitida | 109.0175 | 18.68714 |  |  |
| Callerya nitida | 109.2984 | 18.78729 |  |  |
| Callerya nitida | 109.1478 | 18.59338 |  |  |
| Callerya nitida | 109.1078 | 18.61587 |  |  |
| Callerya nitida | 109.1225 | 18.55639 |  |  |
| Callerya nitida | 109.1248 | 18.53549 |  |  |
| Callerya nitida | 109.652 | 18.254 |  |  |
| Callerya nitida | 109.595 | 18.25624 |  |  |
| Callerya nitida | 109.5973 | 18.22287 |  |  |
| Callerya nitida | 109.6489 | 18.25672 |  |  |
| Callerya nitida | 109.6007 | 18.25707 |  |  |
| Callerya nitida | 109.594 | 18.22344 |  |  |
| Callerya nitida | 109.6565 | 18.25131 |  |  |
| Callerya nitida | 109.5914 | 18.2608 |  |  |
| Callerya nitida | 109.6004 | 18.22394 |  |  |
| Callerya oosperma | 108.9648 | 19.13696 | -29229.1 | 302333.4 |
| Callerya oosperma | 109.0578 | 19.12083 | -19509.9 | 300245 |
| Callerya oosperma | 109.3011 | 18.85155 | 5180.119 | 269701.6 |
| Callerya oosperma | 109.4865 | 19.15807 | 25651.28 | 303028.8 |
| Callerya oosperma | 109.7575 | 19.47974 | 55069.1 | 337810.7 |
| Callerya oosperma | 109.7764 | 18.55929 |  |  |
| Callerya oosperma | 109.8306 | 18.57649 |  |  |
| Callerya oosperma | 109.8597 | 18.59989 |  |  |
| Callerya oosperma | 109.4657 | 19.21689 |  |  |
| Callerya oosperma | 109.3871 | 19.2311 |  |  |
| Callerya oosperma | 109.3538 | 19.21026 |  |  |
| Callerya oosperma | 108.734 | 19.04695 |  |  |
| Callerya oosperma | 108.7446 | 19.06393 |  |  |
| Callerya oosperma | 108.7971 | 18.95084 |  |  |
| Callerya oosperma | 109.0078 | 18.67269 |  |  |
| Callerya oosperma | 109.2945 | 18.79689 |  |  |
| Callerya oosperma | 109.098 | 18.57658 |  |  |
| Callerya oosperma | 109.6515 | 18.2559 |  |  |
| Callerya oosperma | 109.5969 | 18.26169 |  |  |
| Callerya oosperma | 109.5963 | 18.22203 |  |  |
| Callerya oosperma | 109.7284 | 19.51068 |  |  |
| Callerya oosperma | 109.6247 | 19.57762 |  |  |
| Callerya oosperma | 110.0024 | 19.73324 |  |  |
| Callerya oosperma | 109.9816 | 19.71285 |  |  |
| Callerya oosperma | 109.0917 | 19.32458 |  |  |
| Callerya reticulata | 109.1191 | 19.05985 | -13271 | 293304.6 |
| Callerya reticulata | 109.4273 | 19.01928 | 18993.09 | 287860.9 |
| Callerya reticulata | 109.4583 | 19.56476 | 23975.71 | 348070.6 |
| Callerya reticulata | 109.4865 | 19.15807 | 25651.28 | 303028.8 |
| Callerya reticulata | 109.6365 | 19.44697 | 42283.97 | 334529.2 |
| Callerya reticulata | 109.7685 | 18.56337 |  |  |
| Callerya reticulata | 109.8143 | 18.57294 |  |  |
| Callerya reticulata | 109.8537 | 18.59826 |  |  |
| Callerya reticulata | 109.4692 | 19.21763 |  |  |
| Callerya reticulata | 109.3908 | 19.23384 |  |  |
| Callerya reticulata | 109.3491 | 19.21395 |  |  |
| Callerya reticulata | 108.7421 | 19.04043 |  |  |
| Callerya reticulata | 108.7469 | 19.06637 |  |  |
| Callerya reticulata | 108.788 | 18.99027 |  |  |
| Callerya reticulata | 109.0834 | 18.74558 |  |  |
| Callerya reticulata | 109.2996 | 18.79914 |  |  |
| Callerya reticulata | 109.0933 | 18.55391 |  |  |
| Callerya reticulata | 109.6497 | 18.24977 |  |  |
| Callerya reticulata | 109.5908 | 18.25795 |  |  |
| Callerya reticulata | 109.5952 | 18.22578 |  |  |
| Callerya reticulata | 109.7154 | 19.42662 |  |  |
| Callerya reticulata | 109.6878 | 19.4401 |  |  |
| Callerya reticulata | 109.0353 | 19.28577 |  |  |
| Callerya reticulata | 109.1286 | 19.3608 |  |  |
| Callerya reticulata | 109.0967 | 19.33991 |  |  |
| Callerya speciosa | 109.6579 | 18.76335 | 42477.13 | 258885.1 |
| Callerya speciosa | 109.6807 | 18.37829 | 43729.57 | 216234.2 |
| Callerya speciosa | 109.7326 | 19.59786 | 52815.33 | 350936.9 |
| Callerya speciosa | 109.837 | 18.73456 | 61260.82 | 255199 |
| Callerya speciosa | 110.1756 | 18.76435 | 97019.43 | 257602 |
| Callerya speciosa | 109.0069 | 18.74689 |  |  |
| Callerya speciosa | 109.6934 | 18.67711 |  |  |
| Callerya speciosa | 109.5069 | 19.16109 |  |  |
| Callerya speciosa | 109.9056 | 18.97878 |  |  |
| Callerya speciosa | 109.3761 | 19.20457 |  |  |
| Callerya speciosa | 109.987 | 19.26108 |  |  |
| Callerya speciosa | 109.9419 | 19.25676 |  |  |
| Callerya speciosa | 109.3508 | 18.73889 |  |  |
| Callerya speciosa | 109.7383 | 19.25558 |  |  |
| Callerya speciosa | 110.1289 | 19.24275 |  |  |
| Callerya speciosa | 109.7463 | 19.65152 |  |  |
| Callerya speciosa | 109.6894 | 19.82818 |  |  |
| Callerya speciosa | 109.9607 | 19.50588 |  |  |
| Callerya speciosa | 109.7336 | 19.37784 |  |  |
| Callerya speciosa | 109.5898 | 19.62949 |  |  |
| Callerya speciosa | 110.3674 | 19.50394 |  |  |
| Callerya speciosa | 110.1541 | 19.43215 |  |  |
| Callerya speciosa | 109.6335 | 19.34099 |  |  |
| Callerya speciosa | 110.3527 | 19.79612 |  |  |
| Callerya speciosa | 110.4239 | 19.6895 |  |  |
| Callicarpa candicans | 108.9444 | 18.88828 | -32234.3 | 274907.9 |
| Callicarpa candicans | 109.0333 | 18.86036 | -22982.8 | 271527.7 |
| Callicarpa candicans | 109.1041 | 19.50575 | -13336.8 | 342643.7 |
| Callicarpa candicans | 109.1215 | 19.14164 | -12747.8 | 302339.7 |
| Callicarpa candicans | 109.2292 | 18.32242 | -4135.79 | 211411.6 |
| Callicarpa candicans | 109.6579 | 18.76335 | 42477.13 | 258885.1 |
| Callicarpa candicans | 109.7024 | 18.36108 | 45971.25 | 214268.6 |
| Callicarpa candicans | 109.7426 | 19.4888 | 53537.07 | 338853.4 |
| Callicarpa candicans | 109.836 | 19.04117 | 62035.47 | 289106.2 |
| Callicarpa candicans | 109.939 | 19.84766 | 75135.47 | 377987.1 |
| Callicarpa candicans | 110.0221 | 18.80753 | 80965.02 | 262772.1 |
| Callicarpa candicans | 110.0473 | 18.44082 | 82618.44 | 222148.5 |
| Callicarpa candicans | 110.2559 | 19.09526 | 106327.7 | 293994.1 |
| Callicarpa candicans | 110.3722 | 19.99502 | 120817.2 | 393186.7 |
| Callicarpa candicans | 109.84 | 19.13219 |  |  |
| Callicarpa candicans | 109.2767 | 18.99107 |  |  |
| Callicarpa candicans | 109.6794 | 19.16685 |  |  |
| Callicarpa candicans | 109.935 | 19.18377 |  |  |
| Callicarpa candicans | 109.4208 | 18.81492 |  |  |
| Callicarpa candicans | 109.1996 | 18.67993 |  |  |
| Callicarpa candicans | 109.8398 | 18.83649 |  |  |
| Callicarpa candicans | 109.2929 | 19.22742 |  |  |
| Callicarpa candicans | 109.5744 | 18.84511 |  |  |
| Callicarpa candicans | 109.7057 | 19.2729 |  |  |
| Callicarpa candicans | 109.7799 | 19.36084 |  |  |
| Callicarpa candicans | 109.7541 | 19.6853 |  |  |
| Callicarpa candicans | 109.6476 | 19.28287 |  |  |
| Callicarpa candicans | 110.0639 | 19.53181 |  |  |
| Callicarpa candicans | 109.8674 | 19.5675 |  |  |
| Callicarpa candicans | 110.4955 | 19.76939 |  |  |
| Callicarpa candicans | 109.674 | 19.25958 |  |  |
| Callicarpa candicans | 109.8381 | 19.30461 |  |  |
| Callicarpa candicans | 110.0409 | 19.34161 |  |  |
| Callicarpa candicans | 110.0623 | 19.37986 |  |  |
| Callicarpa formosana | 108.6526 | 18.84592 | -63101 | 271225.5 |
| Callicarpa formosana | 109.0333 | 18.86036 | -22982.8 | 271527.7 |
| Callicarpa formosana | 109.1741 | 18.37558 | -9781.56 | 217466.3 |
| Callicarpa formosana | 109.3011 | 18.85155 | 5180.119 | 269701.6 |
| Callicarpa formosana | 109.3163 | 18.42284 | 5389.832 | 222245.7 |
| Callicarpa formosana | 109.3736 | 18.89125 | 12945.56 | 273868.1 |
| Callicarpa formosana | 109.445 | 19.2126 | 21468.7 | 309180.4 |
| Callicarpa formosana | 110.2559 | 19.09526 | 106327.7 | 293994.1 |
| Callicarpa formosana | 110.9392 | 19.65746 | 179350.1 | 354627.5 |
| Callicarpa formosana | 110.4039 | 19.28126 |  |  |
| Callicarpa formosana | 110.3904 | 19.32635 |  |  |
| Callicarpa formosana | 110.5429 | 19.22529 |  |  |
| Callicarpa formosana | 110.5918 | 19.27142 |  |  |
| Callicarpa formosana | 110.5825 | 19.23968 |  |  |
| Callicarpa formosana | 109.6583 | 18.25081 |  |  |
| Callicarpa formosana | 109.5909 | 18.25578 |  |  |
| Callicarpa formosana | 109.5991 | 18.22575 |  |  |
| Callicarpa formosana | 109.0007 | 18.68821 |  |  |
| Callicarpa formosana | 109.2779 | 18.78143 |  |  |
| Callicarpa formosana | 109.049 | 18.57346 |  |  |
| Callicarpa formosana | 109.4658 | 19.21977 |  |  |
| Callicarpa formosana | 109.3892 | 19.22984 |  |  |
| Callicarpa formosana | 109.3494 | 19.21449 |  |  |
| Callicarpa formosana | 109.3529 | 19.21269 |  |  |
| Callicarpa formosana | 109.3517 | 19.21234 |  |  |
| Callicarpa formosana | 109.6239 | 19.52802 |  |  |
| Callicarpa formosana | 109.593 | 19.48081 |  |  |
| Callicarpa formosana | 109.6958 | 19.50157 |  |  |
| Callicarpa formosana | 109.5979 | 19.53375 |  |  |
| Callicarpa kochiana | 110.1756 | 18.76435 | 97019.43 | 257602 |
| Callicarpa kochiana | 109.0368 | 18.947 |  |  |
| Callicarpa kochiana | 110.0438 | 19.22286 |  |  |
| Callicarpa kochiana | 109.6975 | 18.78459 |  |  |
| Callicarpa kochiana | 109.2014 | 19.26647 |  |  |
| Callicarpa kochiana | 109.6383 | 18.80063 |  |  |
| Callicarpa kochiana | 109.7012 | 19.0679 |  |  |
| Callicarpa kochiana | 109.7666 | 19.12804 |  |  |
| Callicarpa kochiana | 108.9643 | 19.2635 |  |  |
| Callicarpa kochiana | 109.8655 | 19.04226 |  |  |
| Callicarpa kochiana | 109.1458 | 18.88879 |  |  |
| Callicarpa kochiana | 109.8867 | 19.69514 |  |  |
| Callicarpa kochiana | 109.9548 | 19.79745 |  |  |
| Callicarpa kochiana | 110.0832 | 19.61672 |  |  |
| Callicarpa kochiana | 110.4056 | 19.21291 |  |  |
| Callicarpa kochiana | 110.2857 | 19.40805 |  |  |
| Callicarpa kochiana | 109.863 | 19.38358 |  |  |
| Callicarpa kochiana | 109.7849 | 19.78681 |  |  |
| Callicarpa kochiana | 109.8954 | 19.38901 |  |  |
| Callicarpa kochiana | 110.1081 | 19.48056 |  |  |
| Callicarpa kochiana | 109.8663 | 19.82859 |  |  |
| Callicarpa longifolia | 109.0333 | 18.86036 | -22982.8 | 271527.7 |
| Callicarpa longifolia | 109.1191 | 19.05985 | -13271 | 293304.6 |
| Callicarpa longifolia | 109.445 | 19.2126 | 21468.7 | 309180.4 |
| Callicarpa longifolia | 109.5104 | 19.51803 | 29281.71 | 342750.8 |
| Callicarpa longifolia | 109.7097 | 19.53075 | 50211.1 | 343582.5 |
| Callicarpa longifolia | 109.768 | 18.56149 |  |  |
| Callicarpa longifolia | 109.8251 | 18.57976 |  |  |
| Callicarpa longifolia | 109.8691 | 18.60441 |  |  |
| Callicarpa longifolia | 109.471 | 19.21951 |  |  |
| Callicarpa longifolia | 109.3882 | 19.23403 |  |  |
| Callicarpa longifolia | 109.3465 | 19.21891 |  |  |
| Callicarpa longifolia | 110.0911 | 18.51907 |  |  |
| Callicarpa longifolia | 110.0592 | 18.53777 |  |  |
| Callicarpa longifolia | 110.0573 | 18.55894 |  |  |
| Callicarpa longifolia | 110.063 | 18.55931 |  |  |
| Callicarpa longifolia | 110.0619 | 18.52234 |  |  |
| Callicarpa longifolia | 109.0112 | 18.73798 |  |  |
| Callicarpa longifolia | 109.2781 | 18.77867 |  |  |
| Callicarpa longifolia | 109.0637 | 18.56351 |  |  |
| Callicarpa longifolia | 109.0919 | 18.56907 |  |  |
| Callicarpa longifolia | 109.1505 | 18.55747 |  |  |
| Callicarpa longifolia | 109.6722 | 19.43461 |  |  |
| Callicarpa longifolia | 109.6736 | 19.53096 |  |  |
| Callicarpa longifolia | 109.5973 | 19.48079 |  |  |
| Callicarpa longifolia | 109.7031 | 19.49788 |  |  |
| Callicarpa rubella | 109.2288 | 19.12078 | -1538.7 | 299692.3 |
| Callicarpa rubella | 109.2343 | 18.80064 | -2022.54 | 264280.3 |
| Callicarpa rubella | 109.2773 | 19.16085 | 3684.27 | 303971.4 |
| Callicarpa rubella | 109.3011 | 18.85155 | 5180.119 | 269701.6 |
| Callicarpa rubella | 109.5425 | 19.03583 | 31162.38 | 289348.8 |
| Callicarpa rubella | 109.675 | 18.86962 | 44597.81 | 270587.7 |
| Callicarpa rubella | 109.4714 | 19.21782 |  |  |
| Callicarpa rubella | 109.4699 | 19.21657 |  |  |
| Callicarpa rubella | 109.4687 | 19.21756 |  |  |
| Callicarpa rubella | 109.4705 | 19.2194 |  |  |
| Callicarpa rubella | 109.4706 | 19.22008 |  |  |
| Callicarpa rubella | 109.471 | 19.21804 |  |  |
| Callicarpa rubella | 109.3512 | 19.21577 |  |  |
| Callicarpa rubella | 109.3536 | 19.21627 |  |  |
| Callicarpa rubella | 109.3552 | 19.21244 |  |  |
| Callicarpa rubella | 109.3541 | 19.21345 |  |  |
| Callicarpa rubella | 109.3541 | 19.21656 |  |  |
| Callicarpa rubella | 109.352 | 19.21004 |  |  |
| Callicarpa rubella | 109.3494 | 19.21015 |  |  |
| Callicarpa rubella | 109.3922 | 19.23452 |  |  |
| Callicarpa rubella | 109.3899 | 19.23603 |  |  |
| Callicarpa rubella | 109.3875 | 19.23389 |  |  |
| Callicarpa rubella | 109.3878 | 19.23445 |  |  |
| Callicarpa rubella | 109.3874 | 19.23613 |  |  |
| Callicarpa rubella | 109.3856 | 19.23627 |  |  |
| Callicarpa rubella | 109.3875 | 19.23052 |  |  |
| Calocedrus macrolepis | 109.1191 | 19.05985 | -13271 | 293304.6 |
| Calocedrus macrolepis | 109.1204 | 19.08606 |  |  |
| Calocedrus macrolepis | 109.1168 | 19.09054 |  |  |
| Calocedrus macrolepis | 109.1232 | 19.08666 |  |  |
| Calocedrus macrolepis | 109.1222 | 19.08777 |  |  |
| Calocedrus macrolepis | 109.124 | 19.08842 |  |  |
| Calocedrus macrolepis | 109.1195 | 19.08744 |  |  |
| Calocedrus macrolepis | 109.1195 | 19.08938 |  |  |
| Calocedrus macrolepis | 109.1182 | 19.08691 |  |  |
| Calocedrus macrolepis | 109.1226 | 19.08877 |  |  |
| Calocedrus macrolepis | 109.117 | 19.08861 |  |  |
| Calocedrus macrolepis | 109.123 | 19.08963 |  |  |
| Calocedrus macrolepis | 109.1182 | 19.08931 |  |  |
| Calocedrus macrolepis | 109.1175 | 19.08593 |  |  |
| Calocedrus macrolepis | 109.1214 | 19.08636 |  |  |
| Calocedrus macrolepis | 109.1191 | 19.08858 |  |  |
| Calocedrus macrolepis | 109.1205 | 19.08977 |  |  |
| Calocedrus macrolepis | 109.1209 | 19.08657 |  |  |
| Calocedrus macrolepis | 109.1231 | 19.08928 |  |  |
| Calocedrus macrolepis | 109.1174 | 19.08565 |  |  |
| Calocedrus macrolepis | 109.1191 | 19.08912 |  |  |
| Calophyllum membranaceum | 109.1041 | 19.50575 | -13336.8 | 342643.7 |
| Calophyllum membranaceum | 109.3011 | 18.85155 | 5180.119 | 269701.6 |
| Calophyllum membranaceum | 109.4865 | 19.15807 | 25651.28 | 303028.8 |
| Calophyllum membranaceum | 109.6579 | 18.76335 | 42477.13 | 258885.1 |
| Calophyllum membranaceum | 109.6807 | 18.37829 | 43729.57 | 216234.2 |
| Calophyllum membranaceum | 109.869 | 18.69141 | 64504.44 | 250339.5 |
| Calophyllum membranaceum | 110.4321 | 19.37563 | 125548.1 | 324570.5 |
| Calophyllum membranaceum | 109.2369 | 18.9055 |  |  |
| Calophyllum membranaceum | 109.3315 | 19.14152 |  |  |
| Calophyllum membranaceum | 109.4933 | 19.11031 |  |  |
| Calophyllum membranaceum | 109.0284 | 19.02489 |  |  |
| Calophyllum membranaceum | 109.7089 | 19.20878 |  |  |
| Calophyllum membranaceum | 109.1059 | 18.74849 |  |  |
| Calophyllum membranaceum | 108.9924 | 19.03567 |  |  |
| Calophyllum membranaceum | 109.6584 | 18.98097 |  |  |
| Calophyllum membranaceum | 109.6745 | 18.65788 |  |  |
| Calophyllum membranaceum | 110.2143 | 19.39158 |  |  |
| Calophyllum membranaceum | 110.1504 | 19.32825 |  |  |
| Calophyllum membranaceum | 110.5006 | 19.22673 |  |  |
| Calophyllum membranaceum | 110.1866 | 19.38467 |  |  |
| Calophyllum membranaceum | 109.8245 | 19.42129 |  |  |
| Calophyllum membranaceum | 109.9323 | 19.23672 |  |  |
| Calophyllum membranaceum | 110.3762 | 19.38428 |  |  |
| Calophyllum membranaceum | 110.1864 | 19.76973 |  |  |
| Calophyllum membranaceum | 110.2247 | 19.20085 |  |  |
| Calophyllum membranaceum | 110.1055 | 19.54342 |  |  |
| Calophyllum membranaceum | 110.1207 | 19.39067 |  |  |
| Calotropis gigantea | 108.6526 | 18.84592 | -63101 | 271225.5 |
| Calotropis gigantea | 108.7804 | 18.80689 | -49793.4 | 266465.5 |
| Calotropis gigantea | 108.7989 | 18.69619 | -48238.4 | 254162.2 |
| Calotropis gigantea | 108.9603 | 18.4598 | -32055.2 | 227476.9 |
| Calotropis gigantea | 109.0333 | 18.86036 | -22982.8 | 271527.7 |
| Calotropis gigantea | 109.655 | 18.25733 |  |  |
| Calotropis gigantea | 109.5907 | 18.25861 |  |  |
| Calotropis gigantea | 109.5938 | 18.22494 |  |  |
| Calotropis gigantea | 109.5934 | 18.22369 |  |  |
| Calotropis gigantea | 109.5952 | 18.2261 |  |  |
| Calotropis gigantea | 109.5914 | 18.2232 |  |  |
| Calotropis gigantea | 109.5936 | 18.22699 |  |  |
| Calotropis gigantea | 108.7362 | 19.04855 |  |  |
| Calotropis gigantea | 108.744 | 19.06412 |  |  |
| Calotropis gigantea | 108.8045 | 18.97935 |  |  |
| Calotropis gigantea | 108.7922 | 18.95395 |  |  |
| Calotropis gigantea | 108.7829 | 18.95196 |  |  |
| Calotropis gigantea | 108.797 | 18.97958 |  |  |
| Calotropis gigantea | 108.7842 | 18.95826 |  |  |
| Calotropis gigantea | 109.0236 | 19.2873 |  |  |
| Calotropis gigantea | 109.0858 | 19.36823 |  |  |
| Calotropis gigantea | 109.1537 | 19.36859 |  |  |
| Calotropis gigantea | 109.1428 | 19.3376 |  |  |
| Calotropis gigantea | 109.0855 | 19.35103 |  |  |
| Calotropis gigantea | 109.1327 | 19.36348 |  |  |
| Camellia caudata | 108.9795 | 18.89322 | -28525.9 | 275338 |
| Camellia caudata | 109.0333 | 18.86036 | -22982.8 | 271527.7 |
| Camellia caudata | 109.2343 | 18.80064 | -2022.54 | 264280.3 |
| Camellia caudata | 109.3011 | 18.85155 | 5180.119 | 269701.6 |
| Camellia caudata | 109.5425 | 19.03583 | 31162.38 | 289348.8 |
| Camellia caudata | 109.6807 | 18.37829 | 43729.57 | 216234.2 |
| Camellia caudata | 109.777 | 19.17887 | 56233.56 | 304494 |
| Camellia caudata | 110.1206 | 19.04626 | 91979.26 | 288915.8 |
| Camellia caudata | 110.1756 | 18.76435 | 97019.43 | 257602 |
| Camellia caudata | 110.2238 | 18.67552 | 101866.1 | 247657.4 |
| Camellia caudata | 109.0196 | 19.29173 |  |  |
| Camellia caudata | 108.9997 | 19.29144 |  |  |
| Camellia caudata | 109.0229 | 19.28987 |  |  |
| Camellia caudata | 109.0137 | 19.30773 |  |  |
| Camellia caudata | 109.035 | 19.30658 |  |  |
| Camellia caudata | 109.1274 | 19.30966 |  |  |
| Camellia caudata | 109.0769 | 19.31789 |  |  |
| Camellia caudata | 109.1576 | 19.30711 |  |  |
| Camellia caudata | 109.1219 | 19.35534 |  |  |
| Camellia caudata | 109.7611 | 19.08181 |  |  |
| Camellia caudata | 109.7772 | 19.02123 |  |  |
| Camellia caudata | 109.8507 | 19.0916 |  |  |
| Camellia caudata | 109.7217 | 19.07979 |  |  |
| Camellia caudata | 109.8071 | 18.98236 |  |  |
| Camellia caudata | 109.8778 | 19.09603 |  |  |
| Camellia caudata | 109.7425 | 19.06799 |  |  |
| Camellia caudata | 109.8458 | 19.03482 |  |  |
| Camellia caudata | 109.7183 | 19.04953 |  |  |
| Camellia caudata | 109.7332 | 19.05617 |  |  |
| Camellia caudata | 109.8321 | 18.96222 |  |  |
| Camellia furfuracea | 109.3011 | 18.85155 | 5180.119 | 269701.6 |
| Camellia furfuracea | 109.429 | 18.50349 | 17547.73 | 230821.1 |
| Camellia furfuracea | 110.457 | 18.89014 | 126969.4 | 270829.8 |
| Camellia furfuracea | 109.821 | 19.10764 |  |  |
| Camellia furfuracea | 109.8296 | 18.70505 |  |  |
| Camellia furfuracea | 109.3713 | 19.06877 |  |  |
| Camellia furfuracea | 109.5749 | 18.79358 |  |  |
| Camellia furfuracea | 109.9548 | 18.77425 |  |  |
| Camellia furfuracea | 108.9935 | 18.85729 |  |  |
| Camellia furfuracea | 109.7389 | 18.82563 |  |  |
| Camellia furfuracea | 109.9268 | 18.66323 |  |  |
| Camellia furfuracea | 109.064 | 18.94239 |  |  |
| Camellia furfuracea | 109.9533 | 19.38235 |  |  |
| Camellia furfuracea | 109.7077 | 19.4959 |  |  |
| Camellia furfuracea | 109.6519 | 19.33775 |  |  |
| Camellia furfuracea | 109.7745 | 19.60937 |  |  |
| Camellia furfuracea | 109.6881 | 19.45634 |  |  |
| Camellia furfuracea | 109.9722 | 19.21201 |  |  |
| Camellia furfuracea | 110.3053 | 19.41512 |  |  |
| Camellia furfuracea | 109.7243 | 19.69753 |  |  |
| Camellia furfuracea | 109.9721 | 19.43601 |  |  |
| Camellia furfuracea | 110.5101 | 19.63922 |  |  |
| Camellia furfuracea | 109.9203 | 19.3487 |  |  |
| Camellia kissi | 109.3233 | 18.69505 | 7006.586 | 252327.2 |
| Camellia kissi | 110.1206 | 19.04626 | 91979.26 | 288915.8 |
| Camellia kissi | 110.1206 | 19.04625 | 91979.43 | 288914.3 |
| Camellia kissi | 110.1206 | 19.04626 | 91979.57 | 288915.7 |
| Camellia kissi | 110.3779 | 19.30761 |  |  |
| Camellia kissi | 110.4302 | 19.30979 |  |  |
| Camellia kissi | 110.3853 | 19.31095 |  |  |
| Camellia kissi | 110.3671 | 19.30867 |  |  |
| Camellia kissi | 110.434 | 19.32197 |  |  |
| Camellia kissi | 110.4274 | 19.3226 |  |  |
| Camellia kissi | 110.3895 | 19.29109 |  |  |
| Camellia kissi | 110.3928 | 19.27636 |  |  |
| Camellia kissi | 110.3656 | 19.31077 |  |  |
| Camellia kissi | 110.5645 | 19.25029 |  |  |
| Camellia kissi | 110.5902 | 19.22453 |  |  |
| Camellia kissi | 110.5826 | 19.24916 |  |  |
| Camellia kissi | 110.5561 | 19.24255 |  |  |
| Camellia kissi | 110.538 | 19.25992 |  |  |
| Camellia kissi | 110.5431 | 19.27359 |  |  |
| Camellia kissi | 110.5762 | 19.25368 |  |  |
| Camellia kissi | 110.5536 | 19.22031 |  |  |
| Camellia kissi | 110.5973 | 19.22785 |  |  |
| Camellia kissi | 110.5384 | 19.259 |  |  |
| Camellia kissi | 110.547 | 19.25409 |  |  |
| Camellia kissii | 109.5153 | 18.61925 | 27012 | 243364.4 |
| Camellia kissii | 109.5153 | 18.61925 | 27012.11 | 243364.5 |
| Camellia kissii | 109.5153 | 18.61925 | 27012.22 | 243364.6 |
| Camellia kissii | 109.5153 | 18.61925 | 27012.32 | 243364.7 |
| Camellia kissii | 109.5153 | 18.61925 | 27012.43 | 243364.8 |
| Camellia kissii | 108.7328 | 19.03983 |  |  |
| Camellia kissii | 108.734 | 19.03993 |  |  |
| Camellia kissii | 108.7314 | 19.0482 |  |  |
| Camellia kissii | 108.7838 | 18.97278 |  |  |
| Camellia kissii | 108.7985 | 18.95008 |  |  |
| Camellia kissii | 108.8087 | 18.9672 |  |  |
| Camellia kissii | 108.7445 | 19.06355 |  |  |
| Camellia kissii | 108.7457 | 19.06663 |  |  |
| Camellia kissii | 108.7439 | 19.06375 |  |  |
| Camellia kissii | 109.7799 | 18.57301 |  |  |
| Camellia kissii | 109.7674 | 18.56901 |  |  |
| Camellia kissii | 109.8601 | 18.60686 |  |  |
| Camellia kissii | 109.8567 | 18.60895 |  |  |
| Camellia kissii | 109.8523 | 18.60519 |  |  |
| Camellia kissii | 109.858 | 18.60271 |  |  |
| Camellia kissii | 109.8254 | 18.5772 |  |  |
| Camellia kissii | 109.8142 | 18.58148 |  |  |
| Camellia kissii | 109.8178 | 18.57962 |  |  |
| Camellia kissii | 109.8183 | 18.57782 |  |  |
| Camellia kissii | 109.8271 | 18.57584 |  |  |
| Campylotropis macrocarpa | 109.5153 | 18.61925 | 27012 | 243364.4 |
| Campylotropis macrocarpa | 109.5153 | 18.61925 | 27012.11 | 243364.5 |
| Campylotropis macrocarpa | 109.5153 | 18.61925 | 27012.22 | 243364.6 |
| Campylotropis macrocarpa | 109.5153 | 18.61925 | 27012.32 | 243364.7 |
| Campylotropis macrocarpa | 109.5153 | 18.61925 | 27012.43 | 243364.8 |
| Campylotropis macrocarpa | 109.0237 | 19.30377 |  |  |
| Campylotropis macrocarpa | 108.996 | 19.27618 |  |  |
| Campylotropis macrocarpa | 109.0325 | 19.31302 |  |  |
| Campylotropis macrocarpa | 109.0094 | 19.30925 |  |  |
| Campylotropis macrocarpa | 109.0222 | 19.31251 |  |  |
| Campylotropis macrocarpa | 108.9929 | 19.28437 |  |  |
| Campylotropis macrocarpa | 109.0346 | 19.3101 |  |  |
| Campylotropis macrocarpa | 109.0163 | 19.29567 |  |  |
| Campylotropis macrocarpa | 109.0367 | 19.29825 |  |  |
| Campylotropis macrocarpa | 109.0861 | 19.32988 |  |  |
| Campylotropis macrocarpa | 109.1485 | 19.3384 |  |  |
| Campylotropis macrocarpa | 109.1103 | 19.36131 |  |  |
| Campylotropis macrocarpa | 109.0729 | 19.32123 |  |  |
| Campylotropis macrocarpa | 109.1217 | 19.35953 |  |  |
| Campylotropis macrocarpa | 109.0804 | 19.3537 |  |  |
| Campylotropis macrocarpa | 109.1417 | 19.30972 |  |  |
| Campylotropis macrocarpa | 109.0843 | 19.35484 |  |  |
| Campylotropis macrocarpa | 109.156 | 19.34466 |  |  |
| Campylotropis macrocarpa | 109.1479 | 19.30855 |  |  |
| Campylotropis macrocarpa | 109.1205 | 19.35644 |  |  |
| Canarium album | 108.7989 | 18.69619 | -48238.4 | 254162.2 |
| Canarium album | 109.0323 | 19.15088 | -22080.8 | 303649.4 |
| Canarium album | 109.0333 | 18.86036 | -22982.8 | 271527.7 |
| Canarium album | 109.1215 | 19.14164 | -12747.8 | 302339.7 |
| Canarium album | 109.2248 | 18.51402 | -3968.98 | 232615.4 |
| Canarium album | 109.2333 | 18.38237 | -3508.87 | 218029 |
| Canarium album | 109.3011 | 18.85155 | 5180.119 | 269701.6 |
| Canarium album | 109.414 | 19.57115 | 19347.16 | 348910 |
| Canarium album | 109.6365 | 19.44697 | 42283.97 | 334529.2 |
| Canarium album | 109.6579 | 18.76335 | 42477.13 | 258885.1 |
| Canarium album | 109.7575 | 19.47974 | 55069.1 | 337810.7 |
| Canarium album | 109.837 | 18.73456 | 61260.82 | 255199 |
| Canarium album | 110.2356 | 18.69662 | 103162.5 | 249961.2 |
| Canarium album | 110.6819 | 19.92734 | 153022.5 | 385001.2 |
| Canarium album | 108.9201 | 18.78525 |  |  |
| Canarium album | 109.9315 | 19.11946 |  |  |
| Canarium album | 109.5465 | 18.98264 |  |  |
| Canarium album | 108.9782 | 18.78961 |  |  |
| Canarium album | 109.0185 | 19.23876 |  |  |
| Canarium album | 109.8889 | 18.74709 |  |  |
| Canarium album | 109.9542 | 19.22758 |  |  |
| Canarium album | 109.9872 | 19.07841 |  |  |
| Canarium album | 109.6677 | 19.1576 |  |  |
| Canarium album | 110.2177 | 19.57425 |  |  |
| Canarium album | 109.8087 | 19.75503 |  |  |
| Canarium album | 110.4842 | 19.31149 |  |  |
| Canarium album | 110.2692 | 19.36793 |  |  |
| Canarium album | 109.7796 | 19.66466 |  |  |
| Canarium album | 109.9718 | 19.61164 |  |  |
| Canarium album | 109.9815 | 19.71659 |  |  |
| Canarium album | 109.9933 | 19.20523 |  |  |
| Canarium album | 110.3285 | 19.1983 |  |  |
| Canarium album | 110.0626 | 19.85669 |  |  |
| Canarium album | 109.6696 | 19.49982 |  |  |
| Canarium pimela | 109.5079 | 19.14711 | 27874.67 | 301753.8 |
| Canarium pimela | 109.837 | 18.73456 | 61260.82 | 255199 |
| Canarium pimela | 108.9222 | 19.23237 |  |  |
| Canarium pimela | 109.2633 | 18.98642 |  |  |
| Canarium pimela | 109.9364 | 19.12397 |  |  |
| Canarium pimela | 109.2676 | 19.12585 |  |  |
| Canarium pimela | 108.9261 | 19.14158 |  |  |
| Canarium pimela | 109.6567 | 19.00548 |  |  |
| Canarium pimela | 109.3128 | 18.87029 |  |  |
| Canarium pimela | 109.3236 | 18.909 |  |  |
| Canarium pimela | 109.3493 | 19.08659 |  |  |
| Canarium pimela | 109.626 | 19.29171 |  |  |
| Canarium pimela | 110.0038 | 19.38476 |  |  |
| Canarium pimela | 109.641 | 19.72634 |  |  |
| Canarium pimela | 110.4751 | 19.30531 |  |  |
| Canarium pimela | 109.8136 | 19.37332 |  |  |
| Canarium pimela | 110.3969 | 19.56057 |  |  |
| Canarium pimela | 110.279 | 19.41175 |  |  |
| Canarium pimela | 110.0921 | 19.3846 |  |  |
| Canarium pimela | 109.8125 | 19.34947 |  |  |
| Canarium pimela | 109.8807 | 19.30496 |  |  |
| Canarium pimela | 110.0476 | 19.40248 |  |  |
| Canavalia cathartica | 109.0365 | 18.87698 | -22580.2 | 273355 |
| Canavalia cathartica | 109.1741 | 18.37558 | -9781.56 | 217466.3 |
| Canavalia cathartica | 109.7326 | 19.59786 | 52815.33 | 350936.9 |
| Canavalia cathartica | 109.9845 | 18.39526 | 75860.07 | 217274.4 |
| Canavalia cathartica | 110.3254 | 20.03167 | 116024 | 397349.5 |
| Canavalia cathartica | 109.4659 | 19.21828 |  |  |
| Canavalia cathartica | 109.3889 | 19.23188 |  |  |
| Canavalia cathartica | 109.3503 | 19.21816 |  |  |
| Canavalia cathartica | 109.354 | 19.21652 |  |  |
| Canavalia cathartica | 109.3543 | 19.21991 |  |  |
| Canavalia cathartica | 109.35 | 19.21008 |  |  |
| Canavalia cathartica | 109.6568 | 18.25505 |  |  |
| Canavalia cathartica | 109.5918 | 18.26044 |  |  |
| Canavalia cathartica | 109.5909 | 18.22401 |  |  |
| Canavalia cathartica | 109.5933 | 18.22474 |  |  |
| Canavalia cathartica | 109.5967 | 18.22446 |  |  |
| Canavalia cathartica | 109.5927 | 18.22193 |  |  |
| Canavalia cathartica | 109.6765 | 19.96901 |  |  |
| Canavalia cathartica | 109.7421 | 19.8936 |  |  |
| Canavalia cathartica | 110.431 | 18.79398 |  |  |
| Canavalia cathartica | 110.3806 | 18.77738 |  |  |
| Canavalia cathartica | 110.4318 | 18.7714 |  |  |
| Canavalia cathartica | 110.2151 | 20.01218 |  |  |
| Canavalia cathartica | 110.457 | 19.84716 |  |  |
| Canavalia cathartica | 110.4168 | 19.85288 |  |  |
| Canavalia rosea | 108.6526 | 18.84592 | -63101 | 271225.5 |
| Canavalia rosea | 109.5301 | 18.22869 | 27361.37 | 200123.7 |
| Canavalia rosea | 109.9845 | 18.39526 | 75860.07 | 217274.4 |
| Canavalia rosea | 110.2332 | 18.6893 | 102892.4 | 249157.1 |
| Canavalia rosea | 110.2332 | 19.6893 | 105499.4 | 359726.6 |
| Canavalia rosea | 109.0166 | 19.31036 |  |  |
| Canavalia rosea | 109.1366 | 19.34727 |  |  |
| Canavalia rosea | 109.0906 | 19.30161 |  |  |
| Canavalia rosea | 109.1023 | 19.36142 |  |  |
| Canavalia rosea | 109.1558 | 19.34138 |  |  |
| Canavalia rosea | 110.4318 | 18.80517 |  |  |
| Canavalia rosea | 110.3808 | 18.77316 |  |  |
| Canavalia rosea | 110.4046 | 18.74994 |  |  |
| Canavalia rosea | 110.4186 | 18.74477 |  |  |
| Canavalia rosea | 110.4121 | 18.77594 |  |  |
| Canavalia rosea | 108.745 | 19.04435 |  |  |
| Canavalia rosea | 108.7444 | 19.06508 |  |  |
| Canavalia rosea | 108.7896 | 18.98893 |  |  |
| Canavalia rosea | 109.0826 | 18.75314 |  |  |
| Canavalia rosea | 109.279 | 18.79921 |  |  |
| Canavalia rosea | 109.1446 | 18.62283 |  |  |
| Canavalia rosea | 109.6528 | 18.25017 |  |  |
| Canavalia rosea | 109.5906 | 18.26106 |  |  |
| Canavalia rosea | 109.5928 | 18.22687 |  |  |
| Canavalia rosea | 110.0634 | 18.55382 |  |  |
| Canthium horridum | 109.1215 | 19.14164 | -12747.8 | 302339.7 |
| Canthium horridum | 109.4865 | 19.15807 | 25651.28 | 303028.8 |
| Canthium horridum | 109.5079 | 19.14711 | 27874.67 | 301753.8 |
| Canthium horridum | 109.6975 | 18.72833 | 46544.54 | 254900 |
| Canthium horridum | 109.7575 | 19.47974 | 55069.1 | 337810.7 |
| Canthium horridum | 109.7776 | 18.56384 |  |  |
| Canthium horridum | 109.8269 | 18.57491 |  |  |
| Canthium horridum | 109.8505 | 18.60796 |  |  |
| Canthium horridum | 109.8711 | 18.60391 |  |  |
| Canthium horridum | 109.8549 | 18.60702 |  |  |
| Canthium horridum | 109.8689 | 18.6011 |  |  |
| Canthium horridum | 110.4156 | 19.31498 |  |  |
| Canthium horridum | 110.5861 | 19.2394 |  |  |
| Canthium horridum | 109.9108 | 18.96823 |  |  |
| Canthium horridum | 109.7928 | 19.06506 |  |  |
| Canthium horridum | 109.8823 | 18.96929 |  |  |
| Canthium horridum | 110.0912 | 18.55874 |  |  |
| Canthium horridum | 110.105 | 18.53512 |  |  |
| Canthium horridum | 109.5759 | 19.55555 |  |  |
| Canthium horridum | 108.9842 | 19.31012 |  |  |
| Canthium horridum | 109.0986 | 19.31201 |  |  |
| Canthium horridum | 108.7457 | 19.06593 |  |  |
| Canthium horridum | 108.7899 | 18.96829 |  |  |
| Canthium horridum | 109.0651 | 18.68713 |  |  |
| Canthium horridum | 110.0719 | 19.81255 |  |  |
| Canthium simile | 109.429 | 18.50349 | 17547.73 | 230821.1 |
| Canthium simile | 109.6579 | 18.76335 | 42477.13 | 258885.1 |
| Canthium simile | 109.6975 | 18.72833 | 46544.54 | 254900 |
| Canthium simile | 109.869 | 18.69141 | 64504.44 | 250339.5 |
| Canthium simile | 110.1924 | 18.76787 | 98795.13 | 257948.6 |
| Canthium simile | 109.7742 | 18.56533 |  |  |
| Canthium simile | 109.8235 | 18.57517 |  |  |
| Canthium simile | 109.8662 | 18.60399 |  |  |
| Canthium simile | 109.8705 | 18.60152 |  |  |
| Canthium simile | 109.8493 | 18.60337 |  |  |
| Canthium simile | 109.6507 | 18.2561 |  |  |
| Canthium simile | 109.5946 | 18.2622 |  |  |
| Canthium simile | 109.6003 | 18.2265 |  |  |
| Canthium simile | 109.5933 | 18.22451 |  |  |
| Canthium simile | 109.5979 | 18.22696 |  |  |
| Canthium simile | 110.0555 | 18.52115 |  |  |
| Canthium simile | 110.1021 | 18.55849 |  |  |
| Canthium simile | 110.0711 | 18.55299 |  |  |
| Canthium simile | 110.0825 | 18.55254 |  |  |
| Canthium simile | 109.7399 | 19.11381 |  |  |
| Canthium simile | 109.899 | 19.00104 |  |  |
| Canthium simile | 109.8688 | 18.98795 |  |  |
| Canthium simile | 109.7892 | 19.00734 |  |  |
| Canthium simile | 109.7678 | 18.98627 |  |  |
| Canthium simile | 109.8569 | 19.06378 |  |  |
| Capparis cantoniensis | 109.1215 | 19.14164 | -12747.8 | 302339.7 |
| Capparis cantoniensis | 109.1592 | 19.12667 | -8839.36 | 300563.6 |
| Capparis cantoniensis | 109.3011 | 18.85155 | 5180.119 | 269701.6 |
| Capparis cantoniensis | 109.4865 | 19.15807 | 25651.28 | 303028.8 |
| Capparis cantoniensis | 110.1756 | 18.76435 | 97019.43 | 257602 |
| Capparis cantoniensis | 109.1705 | 18.78428 |  |  |
| Capparis cantoniensis | 108.9673 | 19.01096 |  |  |
| Capparis cantoniensis | 109.802 | 18.7076 |  |  |
| Capparis cantoniensis | 109.8604 | 18.97805 |  |  |
| Capparis cantoniensis | 109.3298 | 18.726 |  |  |
| Capparis cantoniensis | 109.6341 | 18.89252 |  |  |
| Capparis cantoniensis | 110.0104 | 19.23059 |  |  |
| Capparis cantoniensis | 108.9971 | 19.24787 |  |  |
| Capparis cantoniensis | 110.0322 | 18.69279 |  |  |
| Capparis cantoniensis | 109.6043 | 18.92551 |  |  |
| Capparis cantoniensis | 109.5864 | 19.72657 |  |  |
| Capparis cantoniensis | 109.8461 | 19.2445 |  |  |
| Capparis cantoniensis | 110.2205 | 19.5391 |  |  |
| Capparis cantoniensis | 110.0461 | 19.84616 |  |  |
| Capparis cantoniensis | 109.9555 | 19.63847 |  |  |
| Capparis cantoniensis | 110.091 | 19.41056 |  |  |
| Capparis cantoniensis | 110.3491 | 19.84932 |  |  |
| Capparis cantoniensis | 109.7374 | 19.68946 |  |  |
| Capparis cantoniensis | 110.4709 | 19.23303 |  |  |
| Capparis cantoniensis | 110.2377 | 19.775 |  |  |
| Capparis micracantha | 108.9122 | 18.99917 | -35232.9 | 287275.8 |
| Capparis micracantha | 109.0365 | 18.87698 | -22580.2 | 273355 |
| Capparis micracantha | 109.1041 | 19.50575 | -13336.8 | 342643.7 |
| Capparis micracantha | 109.5795 | 19.8585 | 37567.77 | 380183.2 |
| Capparis micracantha | 109.776 | 18.56618 |  |  |
| Capparis micracantha | 109.7781 | 18.57398 |  |  |
| Capparis micracantha | 109.7822 | 18.56188 |  |  |
| Capparis micracantha | 109.766 | 18.57056 |  |  |
| Capparis micracantha | 109.7832 | 18.55948 |  |  |
| Capparis micracantha | 109.7649 | 18.57132 |  |  |
| Capparis micracantha | 109.8587 | 18.60045 |  |  |
| Capparis micracantha | 109.8503 | 18.60077 |  |  |
| Capparis micracantha | 109.8655 | 18.60164 |  |  |
| Capparis micracantha | 109.8521 | 18.59637 |  |  |
| Capparis micracantha | 109.8514 | 18.60083 |  |  |
| Capparis micracantha | 109.8645 | 18.59859 |  |  |
| Capparis micracantha | 109.8534 | 18.59683 |  |  |
| Capparis micracantha | 109.8152 | 18.5838 |  |  |
| Capparis micracantha | 109.8315 | 18.58215 |  |  |
| Capparis micracantha | 109.8159 | 18.58191 |  |  |
| Capparis micracantha | 109.8284 | 18.5796 |  |  |
| Capparis micracantha | 109.8134 | 18.57509 |  |  |
| Capparis micracantha | 109.826 | 18.58132 |  |  |
| Capparis micracantha | 109.8125 | 18.57852 |  |  |
| Capparis micracantha | 109.6828 | 18.3841 | 43968.29 | 216870.9 |
| Capparis sepiaria | 109.1544 | 19.01211 | -9728.08 | 287914.8 |
| Capparis sepiaria | 109.1544 | 19.01212 | -9727.97 | 287914.9 |
| Capparis sepiaria | 109.1544 | 19.01212 | -9727.86 | 287915 |
| Capparis sepiaria | 109.1544 | 19.01212 | -9727.75 | 287915.1 |
| Capparis sepiaria | 109.1544 | 19.01212 | -9727.65 | 287915.2 |
| Capparis sepiaria | 109.0533 | 18.7584 |  |  |
| Capparis sepiaria | 109.2867 | 18.79584 |  |  |
| Capparis sepiaria | 109.0373 | 18.57453 |  |  |
| Capparis sepiaria | 109.0991 | 18.53277 |  |  |
| Capparis sepiaria | 109.0655 | 18.52711 |  |  |
| Capparis sepiaria | 109.0347 | 18.52102 |  |  |
| Capparis sepiaria | 109.6553 | 18.25194 |  |  |
| Capparis sepiaria | 109.5913 | 18.25933 |  |  |
| Capparis sepiaria | 109.5974 | 18.2247 |  |  |
| Capparis sepiaria | 109.5914 | 18.22189 |  |  |
| Capparis sepiaria | 109.5941 | 18.22189 |  |  |
| Capparis sepiaria | 109.5979 | 18.2228 |  |  |
| Capparis sepiaria | 109.5948 | 18.22145 |  |  |
| Capparis sepiaria | 110.0573 | 18.53572 |  |  |
| Capparis sepiaria | 110.0807 | 18.5449 |  |  |
| Capparis sepiaria | 110.0737 | 18.54757 |  |  |
| Capparis sepiaria | 110.055 | 18.55023 |  |  |
| Capparis sepiaria | 110.0546 | 18.51973 |  |  |
| Capparis sepiaria | 110.0698 | 18.51913 |  |  |
| Capparis sepiaria | 110.0917 | 18.52279 |  |  |
| Capparis spinosa | 109.2292 | 18.32242 | -4135.79 | 211411.6 |
| Capparis spinosa | 109.429 | 18.50349 | 17547.73 | 230821.1 |
| Capparis spinosa | 109.4401 | 18.69801 | 19324.7 | 252298.9 |
| Capparis spinosa | 110.2332 | 18.6893 | 102892.4 | 249157.1 |
| Capparis spinosa | 109.2659 | 18.7974 |  |  |
| Capparis spinosa | 109.6277 | 18.76687 |  |  |
| Capparis spinosa | 109.765 | 18.85937 |  |  |
| Capparis spinosa | 109.6842 | 19.03801 |  |  |
| Capparis spinosa | 109.4658 | 18.77817 |  |  |
| Capparis spinosa | 109.7369 | 19.15659 |  |  |
| Capparis spinosa | 109.8135 | 18.945 |  |  |
| Capparis spinosa | 109.7387 | 18.91212 |  |  |
| Capparis spinosa | 109.7911 | 18.87215 |  |  |
| Capparis spinosa | 109.8148 | 19.52999 |  |  |
| Capparis spinosa | 109.8832 | 19.65346 |  |  |
| Capparis spinosa | 110.337 | 19.62551 |  |  |
| Capparis spinosa | 110.1237 | 19.78744 |  |  |
| Capparis spinosa | 110.1575 | 19.72171 |  |  |
| Capparis spinosa | 109.6138 | 19.22935 |  |  |
| Capparis spinosa | 110.0022 | 19.33308 |  |  |
| Capparis spinosa | 110.2403 | 19.63525 |  |  |
| Capparis spinosa | 109.9324 | 19.75075 |  |  |
| Capparis spinosa | 110.3913 | 19.34408 |  |  |
| Capparis spinosa | 110.1908 | 19.37416 |  |  |
| Capparis zeylanica | 109.1199 | 18.9577 | -13534.4 | 282008.9 |
| Capparis zeylanica | 109.1199 | 18.9577 | -13534.1 | 282008.8 |
| Capparis zeylanica | 109.1199 | 18.95769 | -13534 | 282008.2 |
| Capparis zeylanica | 109.5864 | 19.42585 |  |  |
| Capparis zeylanica | 109.6821 | 19.46008 |  |  |
| Capparis zeylanica | 109.7315 | 19.51746 |  |  |
| Capparis zeylanica | 109.4712 | 19.21703 |  |  |
| Capparis zeylanica | 109.39 | 19.23612 |  |  |
| Capparis zeylanica | 109.3545 | 19.21714 |  |  |
| Capparis zeylanica | 108.7372 | 19.05205 |  |  |
| Capparis zeylanica | 108.7475 | 19.06662 |  |  |
| Capparis zeylanica | 108.8039 | 18.96193 |  |  |
| Capparis zeylanica | 109.0206 | 18.69613 |  |  |
| Capparis zeylanica | 109.2816 | 18.79981 |  |  |
| Capparis zeylanica | 109.037 | 18.60802 |  |  |
| Capparis zeylanica | 109.6556 | 18.24894 |  |  |
| Capparis zeylanica | 109.5917 | 18.26227 |  |  |
| Capparis zeylanica | 109.5912 | 18.226 |  |  |
| Capparis zeylanica | 110.2147 | 20.01165 |  |  |
| Capparis zeylanica | 110.4565 | 19.85456 |  |  |
| Capparis zeylanica | 110.4668 | 19.84971 |  |  |
| Capparis zeylanica | 110.4114 | 19.84674 |  |  |
| Capparis zeylanica | 110.4197 | 19.85527 |  |  |
| Carallia brachiata | 109.0333 | 18.86036 | -22982.8 | 271527.7 |
| Carallia brachiata | 109.0365 | 18.87698 | -22580.2 | 273355 |
| Carallia brachiata | 109.0544 | 19.13823 | -19805.8 | 302178.8 |
| Carallia brachiata | 109.4914 | 19.41113 | 26959.92 | 330989.8 |
| Carallia brachiata | 109.4916 | 18.78671 | 25037.28 | 261953.3 |
| Carallia brachiata | 109.8526 | 18.71296 | 62839.29 | 252768.1 |
| Carallia brachiata | 110.1756 | 18.76435 | 97019.43 | 257602 |
| Carallia brachiata | 110.475 | 19.15751 | 129516.1 | 300354 |
| Carallia brachiata | 109.2054 | 19.18936 |  |  |
| Carallia brachiata | 109.4959 | 18.86751 |  |  |
| Carallia brachiata | 109.1396 | 19.22929 |  |  |
| Carallia brachiata | 109.7897 | 18.6724 |  |  |
| Carallia brachiata | 109.1937 | 19.03281 |  |  |
| Carallia brachiata | 109.1305 | 18.92974 |  |  |
| Carallia brachiata | 109.0136 | 18.84554 |  |  |
| Carallia brachiata | 109.7361 | 18.97727 |  |  |
| Carallia brachiata | 109.2223 | 19.10644 |  |  |
| Carallia brachiata | 109.3729 | 18.71887 |  |  |
| Carallia brachiata | 110.1663 | 19.34472 |  |  |
| Carallia brachiata | 109.6364 | 19.24842 |  |  |
| Carallia brachiata | 109.8232 | 19.52801 |  |  |
| Carallia brachiata | 110.4173 | 19.59828 |  |  |
| Carallia brachiata | 110.3507 | 19.63926 |  |  |
| Carallia brachiata | 110.2101 | 19.4051 |  |  |
| Carallia brachiata | 110.4656 | 19.66107 |  |  |
| Carallia brachiata | 109.9525 | 19.17963 |  |  |
| Carallia brachiata | 110.4959 | 19.41515 |  |  |
| Carallia brachiata | 109.6187 | 19.21652 |  |  |
| Carpinus londoniana | 109.0333 | 18.86036 | -22982.8 | 271527.7 |
| Carpinus londoniana | 109.1191 | 19.05985 | -13271 | 293304.6 |
| Carpinus londoniana | 109.3163 | 18.42284 | 5389.832 | 222245.7 |
| Carpinus londoniana | 109.4865 | 19.15807 | 25651.28 | 303028.8 |
| Carpinus londoniana | 109.6579 | 18.76335 | 42477.13 | 258885.1 |
| Carpinus londoniana | 109.7454 | 18.76502 | 51701.67 | 258821.5 |
| Carpinus londoniana | 110.2857 | 19.56986 |  |  |
| Carpinus londoniana | 110.2753 | 19.54444 |  |  |
| Carpinus londoniana | 110.2235 | 19.62704 |  |  |
| Carpinus londoniana | 110.2554 | 19.65396 |  |  |
| Carpinus londoniana | 110.2362 | 19.56074 |  |  |
| Carpinus londoniana | 110.265 | 19.53761 |  |  |
| Carpinus londoniana | 110.2551 | 19.62571 |  |  |
| Carpinus londoniana | 109.7554 | 19.06706 |  |  |
| Carpinus londoniana | 109.907 | 19.07846 |  |  |
| Carpinus londoniana | 109.7765 | 19.04773 |  |  |
| Carpinus londoniana | 109.7117 | 19.09508 |  |  |
| Carpinus londoniana | 109.8263 | 19.01568 |  |  |
| Carpinus londoniana | 109.8529 | 19.0651 |  |  |
| Carpinus londoniana | 109.7415 | 19.05492 |  |  |
| Carpinus londoniana | 109.9158 | 19.09737 |  |  |
| Carpinus londoniana | 109.8714 | 18.98147 |  |  |
| Carpinus londoniana | 109.7665 | 19.04435 |  |  |
| Carpinus londoniana | 109.72 | 19.09625 |  |  |
| Carpinus londoniana | 109.7965 | 19.07974 |  |  |
| Carpinus londoniana | 109.85 | 19.08494 |  |  |
| Caryota maxima | 109.2248 | 18.51402 | -3968.98 | 232615.4 |
| Caryota maxima | 109.979 | 18.6218 | 75908.64 | 242345.2 |
| Caryota maxima | 110.0221 | 18.80753 | 80965.02 | 262772.1 |
| Caryota maxima | 109.7787 | 18.57038 |  |  |
| Caryota maxima | 109.8177 | 18.58232 |  |  |
| Caryota maxima | 109.8615 | 18.60324 |  |  |
| Caryota maxima | 109.8704 | 18.60624 |  |  |
| Caryota maxima | 109.8583 | 18.60541 |  |  |
| Caryota maxima | 109.8644 | 18.60008 |  |  |
| Caryota maxima | 109.8666 | 18.60114 |  |  |
| Caryota maxima | 109.8587 | 18.59561 |  |  |
| Caryota maxima | 109.8582 | 18.60011 |  |  |
| Caryota maxima | 109.658 | 18.25732 |  |  |
| Caryota maxima | 109.5931 | 18.25826 |  |  |
| Caryota maxima | 109.5973 | 18.22144 |  |  |
| Caryota maxima | 109.5912 | 18.22184 |  |  |
| Caryota maxima | 109.5963 | 18.22493 |  |  |
| Caryota maxima | 109.5978 | 18.2229 |  |  |
| Caryota maxima | 109.5951 | 18.22276 |  |  |
| Caryota maxima | 109.5944 | 18.22553 |  |  |
| Caryota maxima | 109.591 | 18.22182 |  |  |
| Caryota maxima | 109.5945 | 18.22528 |  |  |
| Caryota maxima | 109.5955 | 18.2265 |  |  |
| Caryota mitis | 109.1191 | 19.05985 | -13271 | 293304.6 |
| Caryota mitis | 109.3163 | 18.42284 | 5389.832 | 222245.7 |
| Caryota mitis | 109.5259 | 19.13355 | 29723.31 | 300201.6 |
| Caryota mitis | 109.6006 | 18.43923 | 35449.67 | 223205.2 |
| Caryota mitis | 109.6975 | 18.72833 | 46544.54 | 254900 |
| Caryota mitis | 110.1756 | 18.76435 | 97019.43 | 257602 |
| Caryota mitis | 109.6501 | 18.25393 |  |  |
| Caryota mitis | 109.5904 | 18.26249 |  |  |
| Caryota mitis | 109.5909 | 18.22421 |  |  |
| Caryota mitis | 109.5999 | 18.22163 |  |  |
| Caryota mitis | 109.5966 | 18.2251 |  |  |
| Caryota mitis | 109.5918 | 18.22421 |  |  |
| Caryota mitis | 109.5965 | 18.22445 |  |  |
| Caryota mitis | 109.4655 | 19.21822 |  |  |
| Caryota mitis | 109.3894 | 19.23133 |  |  |
| Caryota mitis | 109.3506 | 19.21842 |  |  |
| Caryota mitis | 110.0663 | 18.54535 |  |  |
| Caryota mitis | 110.0742 | 18.56183 |  |  |
| Caryota mitis | 110.0574 | 18.52998 |  |  |
| Caryota mitis | 110.0741 | 18.51804 |  |  |
| Caryota mitis | 110.0919 | 18.56229 |  |  |
| Caryota mitis | 110.0952 | 18.52243 |  |  |
| Caryota mitis | 110.0703 | 18.52991 |  |  |
| Caryota mitis | 110.0666 | 18.53199 |  |  |
| Caryota mitis | 110.1025 | 18.5397 |  |  |
| Caryota mitis | 110.0541 | 18.53067 |  |  |
| Casearia glomerata | 109.5425 | 19.03583 | 31162.38 | 289348.8 |
| Casearia glomerata | 109.6807 | 18.37829 | 43729.57 | 216234.2 |
| Casearia glomerata | 109.7575 | 19.47974 | 55069.1 | 337810.7 |
| Casearia glomerata | 109.9147 | 19.26366 | 70941.33 | 313492.5 |
| Casearia glomerata | 109.4676 | 18.95829 |  |  |
| Casearia glomerata | 108.9741 | 19.22863 |  |  |
| Casearia glomerata | 109.6399 | 19.10279 |  |  |
| Casearia glomerata | 109.7534 | 18.80816 |  |  |
| Casearia glomerata | 109.8925 | 18.81647 |  |  |
| Casearia glomerata | 109.1938 | 18.72159 |  |  |
| Casearia glomerata | 109.8884 | 18.84353 |  |  |
| Casearia glomerata | 109.5139 | 19.20337 |  |  |
| Casearia glomerata | 108.9616 | 18.70075 |  |  |
| Casearia glomerata | 109.2949 | 18.81686 |  |  |
| Casearia glomerata | 110.4819 | 19.44942 |  |  |
| Casearia glomerata | 109.7378 | 19.59214 |  |  |
| Casearia glomerata | 109.7245 | 19.32019 |  |  |
| Casearia glomerata | 109.9068 | 19.5385 |  |  |
| Casearia glomerata | 110.3332 | 19.64261 |  |  |
| Casearia glomerata | 110.3974 | 19.44319 |  |  |
| Casearia glomerata | 109.6362 | 19.31997 |  |  |
| Casearia glomerata | 110.3349 | 19.47201 |  |  |
| Casearia glomerata | 110.0025 | 19.30596 |  |  |
| Casearia glomerata | 110.4132 | 19.45527 |  |  |
| Casearia membranacea | 109.0333 | 18.86036 | -22982.8 | 271527.7 |
| Casearia membranacea | 109.0609 | 18.7429 | -20480.1 | 258450.9 |
| Casearia membranacea | 109.3163 | 18.42284 | 5389.832 | 222245.7 |
| Casearia membranacea | 109.5425 | 19.03583 | 31162.38 | 289348.8 |
| Casearia membranacea | 109.6365 | 19.44697 | 42283.97 | 334529.2 |
| Casearia membranacea | 109.6579 | 18.76335 | 42477.13 | 258885.1 |
| Casearia membranacea | 109.869 | 18.69141 | 64504.44 | 250339.5 |
| Casearia membranacea | 110.0221 | 18.80753 | 80965.02 | 262772.1 |
| Casearia membranacea | 110.3309 | 19.265 | 114648.9 | 312579.5 |
| Casearia membranacea | 110.4793 | 19.87687 | 131725.4 | 379875.5 |
| Casearia membranacea | 108.7324 | 19.03973 |  |  |
| Casearia membranacea | 108.7447 | 19.06497 |  |  |
| Casearia membranacea | 108.8029 | 18.98743 |  |  |
| Casearia membranacea | 109.7655 | 18.56817 |  |  |
| Casearia membranacea | 109.825 | 18.5784 |  |  |
| Casearia membranacea | 109.862 | 18.59925 |  |  |
| Casearia membranacea | 110.0774 | 18.54055 |  |  |
| Casearia membranacea | 110.0977 | 18.52897 |  |  |
| Casearia membranacea | 110.0841 | 18.5619 |  |  |
| Casearia membranacea | 110.0572 | 18.54688 |  |  |
| Casearia membranacea | 109.8082 | 19.0053 |  |  |
| Casearia membranacea | 109.8971 | 19.02973 |  |  |
| Casearia membranacea | 109.7735 | 19.08131 |  |  |
| Casearia membranacea | 110.421 | 19.2861 |  |  |
| Casearia membranacea | 110.4288 | 19.29555 |  |  |
| Casearia membranacea | 110.3837 | 19.28958 |  |  |
| Casearia membranacea | 110.3899 | 19.32374 |  |  |
| Casearia membranacea | 110.4029 | 19.32258 |  |  |
| Casearia membranacea | 110.5747 | 19.269 |  |  |
| Casearia membranacea | 110.6023 | 19.23914 |  |  |
| Castanopsis carlesii | 110.1756 | 18.76435 | 97019.43 | 257602 |
| Castanopsis carlesii | 109.9248 | 18.85202 |  |  |
| Castanopsis carlesii | 109.1034 | 18.68063 |  |  |
| Castanopsis carlesii | 109.3072 | 19.14286 |  |  |
| Castanopsis carlesii | 110.0207 | 19.02652 |  |  |
| Castanopsis carlesii | 109.7871 | 19.25067 |  |  |
| Castanopsis carlesii | 108.9349 | 18.6673 |  |  |
| Castanopsis carlesii | 109.9827 | 18.78673 |  |  |
| Castanopsis carlesii | 109.0987 | 18.9274 |  |  |
| Castanopsis carlesii | 109.8953 | 19.09575 |  |  |
| Castanopsis carlesii | 109.5631 | 18.98038 |  |  |
| Castanopsis carlesii | 110.2188 | 19.31759 |  |  |
| Castanopsis carlesii | 109.9395 | 19.83704 |  |  |
| Castanopsis carlesii | 110.0515 | 19.62732 |  |  |
| Castanopsis carlesii | 109.8766 | 19.52529 |  |  |
| Castanopsis carlesii | 110.0101 | 19.29642 |  |  |
| Castanopsis carlesii | 109.9652 | 19.28547 |  |  |
| Castanopsis carlesii | 110.0572 | 19.68122 |  |  |
| Castanopsis carlesii | 110.2651 | 19.50206 |  |  |
| Castanopsis carlesii | 110.4109 | 19.796 |  |  |
| Castanopsis carlesii | 109.9107 | 19.17517 |  |  |
| Castanopsis faberi | 109.1215 | 19.14164 | -12747.8 | 302339.7 |
| Castanopsis faberi | 109.4865 | 19.15807 | 25651.28 | 303028.8 |
| Castanopsis faberi | 109.6975 | 18.72833 | 46544.54 | 254900 |
| Castanopsis faberi | 109.8844 | 18.79929 | 66438.38 | 262227.7 |
| Castanopsis faberi | 110.0558 | 18.52812 | 83749.62 | 231782.9 |
| Castanopsis faberi | 110.4381 | 19.37049 |  |  |
| Castanopsis faberi | 110.4019 | 19.57395 |  |  |
| Castanopsis faberi | 110.1094 | 19.43813 |  |  |
| Castanopsis faberi | 110.1483 | 19.78398 |  |  |
| Castanopsis faberi | 110.1955 | 19.70919 |  |  |
| Castanopsis faberi | 109.7236 | 19.38572 |  |  |
| Castanopsis faberi | 110.2254 | 19.25773 |  |  |
| Castanopsis faberi | 109.7276 | 19.36968 |  |  |
| Castanopsis faberi | 109.7431 | 19.52529 |  |  |
| Castanopsis faberi | 109.5978 | 19.31535 |  |  |
| Castanopsis faberi | 109.6705 | 19.42641 |  |  |
| Castanopsis faberi | 109.9536 | 19.39308 |  |  |
| Castanopsis faberi | 110.3987 | 19.76075 |  |  |
| Castanopsis faberi | 110.4961 | 19.62871 |  |  |
| Castanopsis faberi | 109.736 | 19.6249 |  |  |
| Castanopsis faberi | 109.6588 | 19.70831 |  |  |
| Castanopsis faberi | 110.3621 | 19.18384 |  |  |
| Castanopsis faberi | 110.2885 | 19.77816 |  |  |
| Castanopsis faberi | 110.0547 | 19.43372 |  |  |
| Castanopsis faberi | 110.0737 | 19.46842 |  |  |
| Castanopsis fissa | 109.0333 | 18.86036 | -22982.8 | 271527.7 |
| Castanopsis fissa | 109.5377 | 19.09747 | 30846.03 | 296177.5 |
| Castanopsis fissa | 110.0914 | 18.76565 | 88154.38 | 257960.3 |
| Castanopsis fissa | 110.1289 | 18.96031 | 92621.93 | 279390.9 |
| Castanopsis fissa | 110.1756 | 18.76435 | 97019.43 | 257602 |
| Castanopsis fissa | 108.7359 | 19.04224 |  |  |
| Castanopsis fissa | 108.7445 | 19.06541 |  |  |
| Castanopsis fissa | 108.8076 | 18.97731 |  |  |
| Castanopsis fissa | 108.7836 | 18.98003 |  |  |
| Castanopsis fissa | 108.7932 | 18.95682 |  |  |
| Castanopsis fissa | 109.6488 | 18.25596 |  |  |
| Castanopsis fissa | 109.5968 | 18.25657 |  |  |
| Castanopsis fissa | 109.5926 | 18.22345 |  |  |
| Castanopsis fissa | 109.5914 | 18.22389 |  |  |
| Castanopsis fissa | 109.5914 | 18.22632 |  |  |
| Castanopsis fissa | 109.7634 | 18.56491 |  |  |
| Castanopsis fissa | 109.8345 | 18.57527 |  |  |
| Castanopsis fissa | 109.8716 | 18.60815 |  |  |
| Castanopsis fissa | 109.8613 | 18.6038 |  |  |
| Castanopsis fissa | 109.8623 | 18.5991 |  |  |
| Castanopsis fissa | 110.0979 | 18.5565 |  |  |
| Castanopsis fissa | 110.1035 | 18.5384 |  |  |
| Castanopsis fissa | 110.5917 | 19.25567 |  |  |
| Castanopsis fissa | 110.5737 | 19.26056 |  |  |
| Castanopsis fissa | 110.5685 | 19.23983 |  |  |
| Castanopsis hystrix | 109.0333 | 18.86036 | -22982.8 | 271527.7 |
| Castanopsis hystrix | 109.1191 | 19.05985 | -13271 | 293304.6 |
| Castanopsis hystrix | 109.4273 | 19.01928 | 18993.09 | 287860.9 |
| Castanopsis hystrix | 109.4865 | 19.15807 | 25651.28 | 303028.8 |
| Castanopsis hystrix | 109.777 | 19.17887 | 56233.56 | 304494 |
| Castanopsis hystrix | 109.7699 | 19.51828 |  |  |
| Castanopsis hystrix | 110.2594 | 19.77844 |  |  |
| Castanopsis hystrix | 110.2451 | 19.19357 |  |  |
| Castanopsis hystrix | 109.8465 | 19.77095 |  |  |
| Castanopsis hystrix | 109.7796 | 19.53463 |  |  |
| Castanopsis hystrix | 110.4889 | 19.81751 |  |  |
| Castanopsis hystrix | 110.3469 | 19.2567 |  |  |
| Castanopsis hystrix | 109.96 | 19.38218 |  |  |
| Castanopsis hystrix | 110.4851 | 19.21394 |  |  |
| Castanopsis hystrix | 110.2132 | 19.37239 |  |  |
| Castanopsis hystrix | 110.0381 | 19.17301 |  |  |
| Castanopsis hystrix | 110.2682 | 19.60194 |  |  |
| Castanopsis hystrix | 109.7701 | 19.7459 |  |  |
| Castanopsis hystrix | 110.0308 | 19.21497 |  |  |
| Castanopsis hystrix | 110.4914 | 19.54434 |  |  |
| Castanopsis hystrix | 109.5773 | 19.7923 |  |  |
| Castanopsis hystrix | 110.1906 | 19.70627 |  |  |
| Castanopsis hystrix | 110.086 | 19.21546 |  |  |
| Castanopsis hystrix | 110.3854 | 19.46035 |  |  |
| Castanopsis hystrix | 110.4351 | 19.66382 |  |  |
| Castanopsis indica | 108.7989 | 18.69619 | -48238.4 | 254162.2 |
| Castanopsis indica | 109.3011 | 18.85155 | 5180.119 | 269701.6 |
| Castanopsis indica | 109.5377 | 19.09747 | 30846.03 | 296177.5 |
| Castanopsis indica | 109.6975 | 18.72833 | 46544.54 | 254900 |
| Castanopsis indica | 110.1756 | 18.76435 | 97019.43 | 257602 |
| Castanopsis indica | 110.1982 | 19.45681 |  |  |
| Castanopsis indica | 109.6589 | 19.67321 |  |  |
| Castanopsis indica | 110.3761 | 19.39148 |  |  |
| Castanopsis indica | 109.9058 | 19.16486 |  |  |
| Castanopsis indica | 110.4496 | 19.32294 |  |  |
| Castanopsis indica | 110.0583 | 19.57029 |  |  |
| Castanopsis indica | 109.9652 | 19.40033 |  |  |
| Castanopsis indica | 109.7745 | 19.77272 |  |  |
| Castanopsis indica | 109.5819 | 19.76662 |  |  |
| Castanopsis indica | 109.5881 | 19.72376 |  |  |
| Castanopsis indica | 110.0472 | 19.48487 |  |  |
| Castanopsis indica | 110.4079 | 19.47891 |  |  |
| Castanopsis indica | 110.1448 | 19.47226 |  |  |
| Castanopsis indica | 109.7002 | 19.72471 |  |  |
| Castanopsis indica | 109.5801 | 19.38133 |  |  |
| Castanopsis indica | 110.2984 | 19.38214 |  |  |
| Castanopsis indica | 109.8495 | 19.43087 |  |  |
| Castanopsis indica | 110.0452 | 19.38292 |  |  |
| Castanopsis indica | 110.3541 | 19.69795 |  |  |
| Castanopsis indica | 110.5007 | 19.73482 |  |  |
| Catunaregam spinosa | 109.1741 | 18.37558 | -9781.56 | 217466.3 |
| Catunaregam spinosa | 109.5146 | 18.9427 | 27935.88 | 279133.3 |
| Catunaregam spinosa | 109.6828 | 18.3841 | 43968.29 | 216870.9 |
| Catunaregam spinosa | 110.1756 | 18.76435 | 97019.43 | 257602 |
| Catunaregam spinosa | 110.2253 | 18.686 | 102042.4 | 248812.9 |
| Catunaregam spinosa | 110.0001 | 19.21976 |  |  |
| Catunaregam spinosa | 109.9669 | 18.74472 |  |  |
| Catunaregam spinosa | 109.9332 | 18.91903 |  |  |
| Catunaregam spinosa | 109.3855 | 19.19048 |  |  |
| Catunaregam spinosa | 109.574 | 18.77733 |  |  |
| Catunaregam spinosa | 109.8457 | 18.87282 |  |  |
| Catunaregam spinosa | 109.276 | 18.8237 |  |  |
| Catunaregam spinosa | 109.8536 | 19.05042 |  |  |
| Catunaregam spinosa | 109.5597 | 18.99789 |  |  |
| Catunaregam spinosa | 109.391 | 18.77243 |  |  |
| Catunaregam spinosa | 109.7391 | 19.80292 |  |  |
| Catunaregam spinosa | 109.9101 | 19.56959 |  |  |
| Catunaregam spinosa | 109.7999 | 19.23407 |  |  |
| Catunaregam spinosa | 110.3507 | 19.28036 |  |  |
| Catunaregam spinosa | 110.1279 | 19.43653 |  |  |
| Catunaregam spinosa | 110.0252 | 19.58478 |  |  |
| Catunaregam spinosa | 109.5901 | 19.40145 |  |  |
| Catunaregam spinosa | 110.0675 | 19.34049 |  |  |
| Catunaregam spinosa | 110.0669 | 19.42702 |  |  |
| Catunaregam spinosa | 109.8922 | 19.43109 |  |  |
| Cayratia ciliifera | 108.9122 | 18.99917 | -35232.9 | 287275.8 |
| Cayratia ciliifera | 108.9122 | 18.99918 | -35232.7 | 287275.9 |
| Cayratia ciliifera | 108.9122 | 18.99918 | -35232.6 | 287276 |
| Cayratia ciliifera | 109.0333 | 18.86036 | -22982.8 | 271527.7 |
| Cayratia ciliifera | 109.1544 | 19.01211 | -9728.08 | 287914.8 |
| Cayratia ciliifera | 109.0223 | 19.2862 |  |  |
| Cayratia ciliifera | 108.9862 | 19.27646 |  |  |
| Cayratia ciliifera | 108.998 | 19.28205 |  |  |
| Cayratia ciliifera | 109.0358 | 19.31102 |  |  |
| Cayratia ciliifera | 109.0964 | 19.32512 |  |  |
| Cayratia ciliifera | 109.1421 | 19.3613 |  |  |
| Cayratia ciliifera | 109.074 | 19.32925 |  |  |
| Cayratia ciliifera | 109.1573 | 19.366 |  |  |
| Cayratia ciliifera | 109.09 | 19.30824 |  |  |
| Cayratia ciliifera | 108.7361 | 19.05042 |  |  |
| Cayratia ciliifera | 108.7467 | 19.06687 |  |  |
| Cayratia ciliifera | 108.7992 | 18.98222 |  |  |
| Cayratia ciliifera | 109.0217 | 18.67195 |  |  |
| Cayratia ciliifera | 109.2788 | 18.79864 |  |  |
| Cayratia ciliifera | 109.0822 | 18.58906 |  |  |
| Cayratia ciliifera | 109.6539 | 18.25419 |  |  |
| Cayratia ciliifera | 109.594 | 18.25748 |  |  |
| Cayratia ciliifera | 109.5913 | 18.22168 |  |  |
| Cayratia ciliifera | 109.5908 | 18.22651 |  |  |
| Cayratia ciliifera | 109.5917 | 18.22278 |  |  |
| Cayratia corniculata | 108.7989 | 18.69619 | -48238.4 | 254162.2 |
| Cayratia corniculata | 109.1191 | 19.05985 | -13271 | 293304.6 |
| Cayratia corniculata | 109.416 | 18.58215 | 16423.77 | 239559.1 |
| Cayratia corniculata | 109.5146 | 18.9427 | 27935.88 | 279133.3 |
| Cayratia corniculata | 110.2356 | 18.69662 | 103162.5 | 249961.2 |
| Cayratia corniculata | 108.7447 | 19.04748 |  |  |
| Cayratia corniculata | 108.7436 | 19.06437 |  |  |
| Cayratia corniculata | 108.8099 | 18.97249 |  |  |
| Cayratia corniculata | 108.7923 | 18.97903 |  |  |
| Cayratia corniculata | 108.806 | 18.98949 |  |  |
| Cayratia corniculata | 108.8064 | 18.9664 |  |  |
| Cayratia corniculata | 109.4656 | 19.2164 |  |  |
| Cayratia corniculata | 109.39 | 19.23168 |  |  |
| Cayratia corniculata | 109.3467 | 19.22055 |  |  |
| Cayratia corniculata | 109.3475 | 19.21087 |  |  |
| Cayratia corniculata | 109.3481 | 19.21825 |  |  |
| Cayratia corniculata | 109.7807 | 19.01002 |  |  |
| Cayratia corniculata | 109.8244 | 18.96358 |  |  |
| Cayratia corniculata | 109.7988 | 19.04635 |  |  |
| Cayratia corniculata | 109.9039 | 19.07408 |  |  |
| Cayratia corniculata | 109.8101 | 18.98588 |  |  |
| Cayratia corniculata | 109.7363 | 18.96863 |  |  |
| Cayratia corniculata | 109.7301 | 19.03631 |  |  |
| Cayratia corniculata | 109.7266 | 19.03969 |  |  |
| Cayratia corniculata | 109.7699 | 18.96504 |  |  |
| Cayratia geniculata | 108.982 | 18.6052 | -29265.1 | 243483.5 |
| Cayratia geniculata | 109.1811 | 19.79136 | -4312.79 | 373967.5 |
| Cayratia geniculata | 109.6261 | 18.68372 | 38881.47 | 250170.7 |
| Cayratia geniculata | 110.0221 | 18.80753 | 80965.02 | 262772.1 |
| Cayratia geniculata | 110.3315 | 19.15256 | 114426.5 | 300146 |
| Cayratia geniculata | 109.815 | 19.014 |  |  |
| Cayratia geniculata | 109.8192 | 18.97133 |  |  |
| Cayratia geniculata | 109.8468 | 19.05798 |  |  |
| Cayratia geniculata | 110.062 | 18.5424 |  |  |
| Cayratia geniculata | 110.0977 | 18.53406 |  |  |
| Cayratia geniculata | 110.0947 | 18.53315 |  |  |
| Cayratia geniculata | 109.0234 | 18.66967 |  |  |
| Cayratia geniculata | 109.2762 | 18.78314 |  |  |
| Cayratia geniculata | 109.1218 | 18.5426 |  |  |
| Cayratia geniculata | 109.6592 | 18.25714 |  |  |
| Cayratia geniculata | 109.5944 | 18.26129 |  |  |
| Cayratia geniculata | 109.5978 | 18.22715 |  |  |
| Cayratia geniculata | 109.5971 | 18.22645 |  |  |
| Cayratia geniculata | 109.5978 | 18.22502 |  |  |
| Cayratia geniculata | 109.5953 | 18.2248 |  |  |
| Cayratia geniculata | 109.7689 | 18.56772 |  |  |
| Cayratia geniculata | 109.8306 | 18.57389 |  |  |
| Cayratia geniculata | 109.8596 | 18.59896 |  |  |
| Cayratia geniculata | 109.8553 | 18.60107 |  |  |
| Cayratia geniculata | 109.8592 | 18.60739 |  |  |
| Cayratia japonica | 108.794 | 18.69511 | -48760 | 254058.8 |
| Cayratia japonica | 108.9795 | 18.89322 | -28525.9 | 275338 |
| Cayratia japonica | 109.414 | 19.57115 | 19347.16 | 348910 |
| Cayratia japonica | 109.416 | 18.58215 | 16423.77 | 239559.1 |
| Cayratia japonica | 109.4865 | 19.15807 | 25651.28 | 303028.8 |
| Cayratia japonica | 109.4701 | 19.21758 |  |  |
| Cayratia japonica | 109.3904 | 19.23078 |  |  |
| Cayratia japonica | 109.3465 | 19.21471 |  |  |
| Cayratia japonica | 109.3463 | 19.21261 |  |  |
| Cayratia japonica | 109.3522 | 19.21749 |  |  |
| Cayratia japonica | 109.3513 | 19.21286 |  |  |
| Cayratia japonica | 109.6186 | 19.57764 |  |  |
| Cayratia japonica | 109.585 | 19.425 |  |  |
| Cayratia japonica | 109.6591 | 19.51583 |  |  |
| Cayratia japonica | 109.8364 | 19.05382 |  |  |
| Cayratia japonica | 109.8943 | 19.01798 |  |  |
| Cayratia japonica | 109.9051 | 18.96032 |  |  |
| Cayratia japonica | 109.8905 | 19.03165 |  |  |
| Cayratia japonica | 109.862 | 19.1107 |  |  |
| Cayratia japonica | 110.0301 | 19.72991 |  |  |
| Cayratia japonica | 110.0169 | 19.77854 |  |  |
| Cayratia japonica | 110.0189 | 19.8155 |  |  |
| Cayratia japonica | 110.0505 | 19.75329 |  |  |
| Cayratia japonica | 110.0624 | 19.78004 |  |  |
| Cayratia japonica | 110.0024 | 19.78106 |  |  |
| Celastrus hindsii | 109.1191 | 19.05985 | -13271 | 293304.6 |
| Celastrus hindsii | 109.6964 | 19.03697 | 47343.87 | 289030.5 |
| Celastrus hindsii | 109.6964 | 19.03697 | 47344.93 | 289030.6 |
| Celastrus hindsii | 109.6964 | 19.03697 | 47345.98 | 289030.7 |
| Celastrus hindsii | 109.6964 | 19.03697 | 47347.04 | 289030.8 |
| Celastrus hindsii | 109.9576 | 19.74752 |  |  |
| Celastrus hindsii | 110.0416 | 19.78711 |  |  |
| Celastrus hindsii | 110.0067 | 19.73742 |  |  |
| Celastrus hindsii | 109.9768 | 19.80731 |  |  |
| Celastrus hindsii | 110.0537 | 19.77715 |  |  |
| Celastrus hindsii | 109.9997 | 19.7606 |  |  |
| Celastrus hindsii | 109.6535 | 18.24963 |  |  |
| Celastrus hindsii | 109.5956 | 18.26087 |  |  |
| Celastrus hindsii | 109.5991 | 18.2263 |  |  |
| Celastrus hindsii | 109.5981 | 18.22691 |  |  |
| Celastrus hindsii | 109.7786 | 18.55969 |  |  |
| Celastrus hindsii | 109.8313 | 18.57917 |  |  |
| Celastrus hindsii | 109.8544 | 18.59883 |  |  |
| Celastrus hindsii | 109.4709 | 19.21799 |  |  |
| Celastrus hindsii | 109.3937 | 19.23023 |  |  |
| Celastrus hindsii | 109.3451 | 19.2199 |  |  |
| Celastrus hindsii | 109.3471 | 19.21519 |  |  |
| Celastrus hindsii | 109.3454 | 19.2116 |  |  |
| Celastrus hindsii | 109.8333 | 18.57766 |  |  |
| Celastrus hindsii | 109.8215 | 18.57563 |  |  |
| Celastrus kusanoi | 109.6628 | 18.86803 | 43304.48 | 270446.3 |
| Celastrus monospermus | 109.4401 | 18.69801 | 19324.7 | 252298.9 |
| Celastrus monospermus | 109.5146 | 18.9427 | 27935.56 | 279133.3 |
| Celastrus monospermus | 109.7806 | 18.56093 |  |  |
| Celastrus monospermus | 109.8313 | 18.57671 |  |  |
| Celastrus monospermus | 109.8509 | 18.60196 |  |  |
| Celastrus monospermus | 109.4673 | 19.21811 |  |  |
| Celastrus monospermus | 109.3934 | 19.2343 |  |  |
| Celastrus monospermus | 109.3493 | 19.21857 |  |  |
| Celastrus monospermus | 109.785 | 18.57451 |  |  |
| Celastrus monospermus | 109.8154 | 18.58711 |  |  |
| Celastrus monospermus | 109.8543 | 18.60256 |  |  |
| Celastrus monospermus | 109.4675 | 19.21795 |  |  |
| Celastrus monospermus | 109.3886 | 19.23225 |  |  |
| Celastrus monospermus | 109.3481 | 19.21389 |  |  |
| Celastrus monospermus | 109.7836 | 18.56241 |  |  |
| Celastrus monospermus | 109.827 | 18.57389 |  |  |
| Celastrus monospermus | 109.8492 | 18.59646 |  |  |
| Celastrus monospermus | 109.4695 | 19.22032 |  |  |
| Celastrus monospermus | 109.3896 | 19.2332 |  |  |
| Celastrus monospermus | 109.3553 | 19.21609 |  |  |
| Celastrus monospermus | 109.8345 | 18.57324 |  |  |
| Celastrus monospermus | 109.8584 | 18.60645 |  |  |
| Celtis bungeana | 109.0333 | 18.86036 | -22982.8 | 271527.7 |
| Celtis bungeana | 109.1191 | 19.05985 | -13271 | 293304.6 |
| Celtis bungeana | 109.1544 | 19.01211 | -9728.08 | 287914.8 |
| Celtis bungeana | 109.3011 | 18.85155 | 5180.119 | 269701.6 |
| Celtis bungeana | 109.3011 | 18.85156 | 5181.175 | 269701.6 |
| Celtis bungeana | 109.073 | 18.69016 |  |  |
| Celtis bungeana | 109.2958 | 18.7873 |  |  |
| Celtis bungeana | 109.13 | 18.52982 |  |  |
| Celtis bungeana | 109.0799 | 18.74565 |  |  |
| Celtis bungeana | 109.2739 | 18.78142 |  |  |
| Celtis bungeana | 109.1438 | 18.56492 |  |  |
| Celtis bungeana | 109.06 | 18.71519 |  |  |
| Celtis bungeana | 109.2856 | 18.79063 |  |  |
| Celtis bungeana | 109.1267 | 18.57088 |  |  |
| Celtis bungeana | 109.7751 | 18.56411 |  |  |
| Celtis bungeana | 109.8218 | 18.5849 |  |  |
| Celtis bungeana | 109.8687 | 18.60049 |  |  |
| Celtis bungeana | 109.7719 | 18.57376 |  |  |
| Celtis bungeana | 109.8196 | 18.57391 |  |  |
| Celtis bungeana | 109.849 | 18.59717 |  |  |
| Celtis bungeana | 109.7653 | 18.55865 |  |  |
| Celtis bungeana | 109.8296 | 18.57702 |  |  |
| Celtis bungeana | 109.8586 | 18.59656 |  |  |
| Celtis bungeana | 109.8606 | 18.60426 |  |  |
| Celtis bungeana | 109.8635 | 18.59664 |  |  |
| Celtis philippensis | 109.0333 | 18.86036 | -22982.8 | 271527.7 |
| Celtis philippensis | 109.0609 | 18.7429 | -20480.1 | 258450.9 |
| Celtis philippensis | 109.3569 | 19.7494 | 13938.79 | 368784.9 |
| Celtis philippensis | 109.6535 | 18.2575 | 40487.08 | 202951.5 |
| Celtis philippensis | 109.9845 | 18.39526 | 75860.07 | 217274.4 |
| Celtis philippensis | 109.6531 | 18.25207 |  |  |
| Celtis philippensis | 109.6557 | 18.25193 |  |  |
| Celtis philippensis | 109.6493 | 18.25342 |  |  |
| Celtis philippensis | 109.6535 | 18.25541 |  |  |
| Celtis philippensis | 109.6578 | 18.25666 |  |  |
| Celtis philippensis | 109.5986 | 18.26082 |  |  |
| Celtis philippensis | 109.6014 | 18.2586 |  |  |
| Celtis philippensis | 109.5985 | 18.26023 |  |  |
| Celtis philippensis | 109.5995 | 18.22194 |  |  |
| Celtis philippensis | 109.591 | 18.22225 |  |  |
| Celtis philippensis | 109.5953 | 18.22721 |  |  |
| Celtis philippensis | 109.6999 | 19.45794 |  |  |
| Celtis philippensis | 109.7072 | 19.45448 |  |  |
| Celtis philippensis | 109.7178 | 19.50787 |  |  |
| Celtis philippensis | 109.633 | 19.52372 |  |  |
| Celtis philippensis | 109.6262 | 19.51847 |  |  |
| Celtis philippensis | 109.5808 | 19.56121 |  |  |
| Celtis philippensis | 109.6636 | 19.48164 |  |  |
| Celtis philippensis | 109.6851 | 19.48087 |  |  |
| Celtis philippensis | 109.5757 | 19.50883 |  |  |
| Celtis sinensis | 108.9868 | 19.0313 | -27277.7 | 290579.7 |
| Celtis sinensis | 109.0799 | 19.00987 | -17563.9 | 287906.3 |
| Celtis sinensis | 109.1544 | 19.01211 | -9728.08 | 287914.8 |
| Celtis sinensis | 109.777 | 19.17887 | 56233.56 | 304494 |
| Celtis sinensis | 110.3426 | 20.03262 | 117817.4 | 397413.5 |
| Celtis sinensis | 109.7193 | 19.50944 |  |  |
| Celtis sinensis | 109.6997 | 19.45785 |  |  |
| Celtis sinensis | 109.6851 | 19.47234 |  |  |
| Celtis sinensis | 109.7162 | 19.50293 |  |  |
| Celtis sinensis | 109.6745 | 19.57319 |  |  |
| Celtis sinensis | 108.735 | 19.04411 |  |  |
| Celtis sinensis | 108.745 | 19.06591 |  |  |
| Celtis sinensis | 108.7934 | 18.97961 |  |  |
| Celtis sinensis | 108.7447 | 19.04841 |  |  |
| Celtis sinensis | 108.7469 | 19.06524 |  |  |
| Celtis sinensis | 108.8083 | 18.97152 |  |  |
| Celtis sinensis | 110.1045 | 18.52197 |  |  |
| Celtis sinensis | 110.0824 | 18.53256 |  |  |
| Celtis sinensis | 110.0914 | 18.52538 |  |  |
| Celtis sinensis | 110.0969 | 18.52542 |  |  |
| Celtis sinensis | 110.0605 | 18.53991 |  |  |
| Celtis sinensis | 110.1022 | 18.52495 |  |  |
| Celtis sinensis | 110.0935 | 18.51631 |  |  |
| Celtis sinensis | 110.0674 | 18.55054 |  |  |
| Celtis sinensis | 110.1022 | 18.55973 |  |  |
| Celtis timorensis | 109.0333 | 18.86036 | -22982.8 | 271527.7 |
| Celtis timorensis | 109.3163 | 18.42284 | 5389.832 | 222245.7 |
| Celtis timorensis | 109.6535 | 18.2575 | 40487.08 | 202951.5 |
| Celtis timorensis | 109.6964 | 19.03697 | 47343.87 | 289030.5 |
| Celtis timorensis | 110.1756 | 18.76435 | 97019.43 | 257602 |
| Celtis timorensis | 108.7455 | 19.04452 |  |  |
| Celtis timorensis | 108.7455 | 19.06585 |  |  |
| Celtis timorensis | 108.7995 | 18.97076 |  |  |
| Celtis timorensis | 108.7846 | 18.9611 |  |  |
| Celtis timorensis | 108.78 | 18.96548 |  |  |
| Celtis timorensis | 108.796 | 18.97603 |  |  |
| Celtis timorensis | 110.0072 | 19.72373 |  |  |
| Celtis timorensis | 110.0012 | 19.77879 |  |  |
| Celtis timorensis | 110.0116 | 19.80914 |  |  |
| Celtis timorensis | 109.9747 | 19.76028 |  |  |
| Celtis timorensis | 110.3114 | 19.657 |  |  |
| Celtis timorensis | 110.287 | 19.61398 |  |  |
| Celtis timorensis | 110.2311 | 19.61853 |  |  |
| Celtis timorensis | 110.2817 | 19.5269 |  |  |
| Celtis timorensis | 109.4681 | 19.21956 |  |  |
| Celtis timorensis | 109.388 | 19.23001 |  |  |
| Celtis timorensis | 109.3524 | 19.2201 |  |  |
| Celtis timorensis | 109.4665 | 19.21693 |  |  |
| Celtis timorensis | 109.3958 | 19.23399 |  |  |
| Celtis timorensis | 109.3477 | 19.22 |  |  |
| Celtis vandervoetiana | 109.416 | 18.58215 | 16423.77 | 239559.1 |
| Celtis vandervoetiana | 109.7672 | 18.56349 |  |  |
| Celtis vandervoetiana | 109.779 | 18.57361 |  |  |
| Celtis vandervoetiana | 109.7655 | 18.55912 |  |  |
| Celtis vandervoetiana | 109.7748 | 18.55926 |  |  |
| Celtis vandervoetiana | 109.7846 | 18.56187 |  |  |
| Celtis vandervoetiana | 109.7666 | 18.55998 |  |  |
| Celtis vandervoetiana | 109.7836 | 18.55839 |  |  |
| Celtis vandervoetiana | 109.8328 | 18.57324 |  |  |
| Celtis vandervoetiana | 109.8262 | 18.57706 |  |  |
| Celtis vandervoetiana | 109.8187 | 18.58379 |  |  |
| Celtis vandervoetiana | 109.8223 | 18.57561 |  |  |
| Celtis vandervoetiana | 109.8145 | 18.58624 |  |  |
| Celtis vandervoetiana | 109.8235 | 18.57547 |  |  |
| Celtis vandervoetiana | 109.8563 | 18.59823 |  |  |
| Celtis vandervoetiana | 109.8716 | 18.59882 |  |  |
| Celtis vandervoetiana | 109.8516 | 18.60787 |  |  |
| Celtis vandervoetiana | 109.8655 | 18.60732 |  |  |
| Celtis vandervoetiana | 109.8536 | 18.60607 |  |  |
| Celtis vandervoetiana | 109.8704 | 18.60196 |  |  |
| Celtis vandervoetiana | 109.8513 | 18.60783 |  |  |
| Cephalotaxus fortunei | 109.0333 | 18.86036 | -22982.8 | 271527.7 |
| Cephalotaxus fortunei | 109.8158 | 19.00488 |  |  |
| Cephalotaxus fortunei | 109.8902 | 18.99391 |  |  |
| Cephalotaxus fortunei | 109.8731 | 19.05099 |  |  |
| Cephalotaxus fortunei | 109.8557 | 19.00323 |  |  |
| Cephalotaxus fortunei | 109.9105 | 18.98373 |  |  |
| Cephalotaxus fortunei | 109.7927 | 19.03322 |  |  |
| Cephalotaxus fortunei | 109.8083 | 18.97726 |  |  |
| Cephalotaxus fortunei | 109.7865 | 19.11792 |  |  |
| Cephalotaxus fortunei | 109.7799 | 18.98303 |  |  |
| Cephalotaxus fortunei | 109.9079 | 19.02654 |  |  |
| Cephalotaxus fortunei | 109.7559 | 19.0013 |  |  |
| Cephalotaxus fortunei | 109.9187 | 19.07716 |  |  |
| Cephalotaxus fortunei | 109.8488 | 19.03048 |  |  |
| Cephalotaxus fortunei | 109.8625 | 19.00217 |  |  |
| Cephalotaxus fortunei | 109.8181 | 18.95983 |  |  |
| Cephalotaxus fortunei | 109.7731 | 19.07124 |  |  |
| Cephalotaxus fortunei | 109.9042 | 19.07881 |  |  |
| Cephalotaxus fortunei | 109.8821 | 18.97936 |  |  |
| Cephalotaxus fortunei | 109.8097 | 19.05629 |  |  |
| Cephalotaxus fortunei | 109.7649 | 19.00958 |  |  |
| Cephalotaxus mannii | 109.0333 | 18.86036 | -22982.8 | 271527.7 |
| Cephalotaxus mannii | 109.1191 | 19.05985 | -13271 | 293304.6 |
| Cephalotaxus mannii | 109.1215 | 19.14164 | -12747.8 | 302339.7 |
| Cephalotaxus mannii | 109.1544 | 19.01211 | -9728.08 | 287914.8 |
| Cephalotaxus mannii | 109.5425 | 19.03583 | 31162.38 | 289348.8 |
| Cephalotaxus mannii | 109.1221 | 19.0886 |  |  |
| Cephalotaxus mannii | 109.1169 | 19.08619 |  |  |
| Cephalotaxus mannii | 109.1233 | 19.08975 |  |  |
| Cephalotaxus mannii | 109.119 | 19.08609 |  |  |
| Cephalotaxus mannii | 109.1189 | 19.08664 |  |  |
| Cephalotaxus mannii | 109.1191 | 19.08837 |  |  |
| Cephalotaxus mannii | 109.6671 | 18.84095 |  |  |
| Cephalotaxus mannii | 109.6596 | 18.84281 |  |  |
| Cephalotaxus mannii | 109.6789 | 18.85063 |  |  |
| Cephalotaxus mannii | 109.263 | 19.03917 |  |  |
| Cephalotaxus mannii | 109.2579 | 19.03687 |  |  |
| Cephalotaxus mannii | 109.2575 | 19.04542 |  |  |
| Cephalotaxus mannii | 109.2626 | 19.03684 |  |  |
| Cephalotaxus mannii | 109.2727 | 19.03441 |  |  |
| Cephalotaxus mannii | 109.7569 | 19.20619 |  |  |
| Cephalotaxus mannii | 109.7575 | 19.16089 |  |  |
| Cephalotaxus mannii | 109.7751 | 19.18952 |  |  |
| Cephalotaxus mannii | 109.7612 | 19.1604 |  |  |
| Cephalotaxus mannii | 109.7583 | 19.19743 |  |  |
| Cephalotaxus mannii | 109.7672 | 19.16059 |  |  |
| Cephalotaxus sinensis | 109.0333 | 18.86036 | -22982.8 | 271527.7 |
| Cephalotaxus sinensis | 109.8188 | 19.14345 | 60528.46 | 300462.2 |
| Cephalotaxus sinensis | 109.8188 | 19.14345 | 60528.57 | 300462.3 |
| Cephalotaxus sinensis | 109.8188 | 19.14345 | 60528.68 | 300462.4 |
| Cephalotaxus sinensis | 109.8188 | 19.14346 | 60528.78 | 300462.5 |
| Cephalotaxus sinensis | 108.7407 | 19.04652 |  |  |
| Cephalotaxus sinensis | 108.745 | 19.0666 |  |  |
| Cephalotaxus sinensis | 108.8011 | 18.9845 |  |  |
| Cephalotaxus sinensis | 108.7886 | 18.97892 |  |  |
| Cephalotaxus sinensis | 108.7942 | 18.97633 |  |  |
| Cephalotaxus sinensis | 108.7842 | 18.98188 |  |  |
| Cephalotaxus sinensis | 108.7826 | 18.96456 |  |  |
| Cephalotaxus sinensis | 108.785 | 18.95578 |  |  |
| Cephalotaxus sinensis | 108.7991 | 18.98876 |  |  |
| Cephalotaxus sinensis | 108.7948 | 18.98356 |  |  |
| Cephalotaxus sinensis | 109.7789 | 18.57153 |  |  |
| Cephalotaxus sinensis | 109.8245 | 18.58226 |  |  |
| Cephalotaxus sinensis | 109.8584 | 18.59559 |  |  |
| Cephalotaxus sinensis | 109.8639 | 18.60082 |  |  |
| Cephalotaxus sinensis | 109.8677 | 18.60631 |  |  |
| Cephalotaxus sinensis | 109.8587 | 18.59955 |  |  |
| Cephalotaxus sinensis | 109.8516 | 18.59706 |  |  |
| Cephalotaxus sinensis | 109.8688 | 18.5985 |  |  |
| Cephalotaxus sinensis | 109.8611 | 18.60321 |  |  |
| Cephalotaxus sinensis | 109.8584 | 18.60542 |  |  |
| Cerbera manghas | 109.1741 | 18.37558 | -9781.56 | 217466.3 |
| Cerbera manghas | 109.6401 | 19.49205 | 42797.58 | 339502 |
| Cerbera manghas | 110.2332 | 18.6893 | 102892.4 | 249157.1 |
| Cerbera manghas | 110.4635 | 19.25438 | 128544.6 | 311091.5 |
| Cerbera manghas | 109.5348 | 18.67316 |  |  |
| Cerbera manghas | 109.3667 | 19.1367 |  |  |
| Cerbera manghas | 109.3938 | 19.24768 |  |  |
| Cerbera manghas | 109.9983 | 19.23855 |  |  |
| Cerbera manghas | 109.5284 | 18.85158 |  |  |
| Cerbera manghas | 109.9573 | 19.04228 |  |  |
| Cerbera manghas | 109.2105 | 19.20416 |  |  |
| Cerbera manghas | 109.5399 | 19.06818 |  |  |
| Cerbera manghas | 108.9706 | 19.03429 |  |  |
| Cerbera manghas | 109.5598 | 18.98845 |  |  |
| Cerbera manghas | 110.1685 | 19.81121 |  |  |
| Cerbera manghas | 110.0289 | 19.62767 |  |  |
| Cerbera manghas | 110.3762 | 19.47813 |  |  |
| Cerbera manghas | 110.0291 | 19.78424 |  |  |
| Cerbera manghas | 109.9669 | 19.47773 |  |  |
| Cerbera manghas | 110.4811 | 19.67277 |  |  |
| Cerbera manghas | 109.6863 | 19.4059 |  |  |
| Cerbera manghas | 110.294 | 19.37028 |  |  |
| Cerbera manghas | 109.9147 | 19.4219 |  |  |
| Cerbera manghas | 110.3056 | 19.50239 |  |  |
| Ceriops tagal | 109.7111 | 18.26491 | 46605.9 | 203606.1 |
| Ceriops tagal | 110.6146 | 19.99826 | 146153.1 | 392988.4 |
| Ceriops tagal | 109.9669 | 19.76945 |  |  |
| Ceriops tagal | 109.9679 | 19.75939 |  |  |
| Ceriops tagal | 109.9746 | 19.75894 |  |  |
| Ceriops tagal | 110.0139 | 19.78588 |  |  |
| Ceriops tagal | 110.0618 | 19.76383 |  |  |
| Ceriops tagal | 109.615 | 19.50156 |  |  |
| Ceriops tagal | 109.5917 | 19.53305 |  |  |
| Ceriops tagal | 109.7258 | 19.43045 |  |  |
| Ceriops tagal | 109.7057 | 19.44804 |  |  |
| Ceriops tagal | 109.5915 | 19.47133 |  |  |
| Ceriops tagal | 110.9282 | 19.57542 |  |  |
| Ceriops tagal | 110.7731 | 19.67182 |  |  |
| Ceriops tagal | 110.781 | 19.68408 |  |  |
| Ceriops tagal | 110.7854 | 19.66529 |  |  |
| Ceriops tagal | 109.6487 | 18.25222 |  |  |
| Ceriops tagal | 109.5905 | 18.25893 |  |  |
| Ceriops tagal | 109.5922 | 18.22667 |  |  |
| Ceriops tagal | 109.6523 | 18.24868 |  |  |
| Ceriops tagal | 109.5964 | 18.25888 |  |  |
| Ceriops tagal | 109.5968 | 18.2255 |  |  |
| Chassalia curviflora | 108.982 | 18.6052 | -29265.1 | 243483.5 |
| Chassalia curviflora | 109.2625 | 19.48091 | 3180.794 | 339398.2 |
| Chassalia curviflora | 109.3011 | 18.85155 | 5180.119 | 269701.6 |
| Chassalia curviflora | 109.4273 | 19.01928 | 18993.09 | 287860.9 |
| Chassalia curviflora | 109.4865 | 19.15807 | 25651.28 | 303028.8 |
| Chassalia curviflora | 109.7345 | 18.74207 | 50478.48 | 256315 |
| Chassalia curviflora | 109.7575 | 19.47974 | 55069.1 | 337810.7 |
| Chassalia curviflora | 109.7647 | 19.48805 | 55849.78 | 338708.9 |
| Chassalia curviflora | 110.0221 | 18.80753 | 80965.02 | 262772.1 |
| Chassalia curviflora | 110.1296 | 18.59148 | 91703.1 | 238599.5 |
| Chassalia curviflora | 109.7711 | 18.56717 |  |  |
| Chassalia curviflora | 109.8336 | 18.58206 |  |  |
| Chassalia curviflora | 109.8572 | 18.59682 |  |  |
| Chassalia curviflora | 109.4664 | 19.22006 |  |  |
| Chassalia curviflora | 109.3853 | 19.23076 |  |  |
| Chassalia curviflora | 109.3516 | 19.21363 |  |  |
| Chassalia curviflora | 110.0996 | 18.5607 |  |  |
| Chassalia curviflora | 110.0724 | 18.54608 |  |  |
| Chassalia curviflora | 110.0686 | 18.53989 |  |  |
| Chassalia curviflora | 110.064 | 18.53267 |  |  |
| Chassalia curviflora | 110.101 | 18.56192 |  |  |
| Chassalia curviflora | 109.0187 | 19.29829 |  |  |
| Chassalia curviflora | 109.0165 | 19.29521 |  |  |
| Chassalia curviflora | 108.9951 | 19.3102 |  |  |
| Chassalia curviflora | 109.0172 | 19.28809 |  |  |
| Chassalia curviflora | 109.0879 | 19.31559 |  |  |
| Chassalia curviflora | 109.1568 | 19.31491 |  |  |
| Chassalia curviflora | 109.1102 | 19.35067 |  |  |
| Chassalia curviflora | 109.1521 | 19.35449 |  |  |
| Chassalia curviflora | 109.0896 | 19.33705 |  |  |
| Chieniodendron hainanense | 109.1741 | 18.37558 | -9781.56 | 217466.3 |
| Chieniodendron hainanense | 109.416 | 18.58215 | 16423.77 | 239559.1 |
| Chieniodendron hainanense | 109.4865 | 19.15807 | 25651.28 | 303028.8 |
| Chieniodendron hainanense | 109.5425 | 19.03583 | 31162.38 | 289348.8 |
| Chieniodendron hainanense | 110.2542 | 18.78692 | 105355.2 | 259902.1 |
| Chieniodendron hainanense | 110.0559 | 18.54212 |  |  |
| Chieniodendron hainanense | 110.09 | 18.52254 |  |  |
| Chieniodendron hainanense | 110.0931 | 18.5589 |  |  |
| Chieniodendron hainanense | 110.0639 | 18.5485 |  |  |
| Chieniodendron hainanense | 110.0622 | 18.51351 |  |  |
| Chieniodendron hainanense | 110.089 | 18.53817 |  |  |
| Chieniodendron hainanense | 109.0058 | 19.29276 |  |  |
| Chieniodendron hainanense | 109.0241 | 19.28647 |  |  |
| Chieniodendron hainanense | 108.994 | 19.2758 |  |  |
| Chieniodendron hainanense | 108.9872 | 19.28085 |  |  |
| Chieniodendron hainanense | 109.096 | 19.33308 |  |  |
| Chieniodendron hainanense | 109.084 | 19.3495 |  |  |
| Chieniodendron hainanense | 109.1502 | 19.35444 |  |  |
| Chieniodendron hainanense | 109.0908 | 19.3232 |  |  |
| Chieniodendron hainanense | 108.7398 | 19.04908 |  |  |
| Chieniodendron hainanense | 108.7452 | 19.06532 |  |  |
| Chieniodendron hainanense | 108.7914 | 18.96466 |  |  |
| Chieniodendron hainanense | 109.5981 | 18.26074 |  |  |
| Chieniodendron hainanense | 109.594 | 18.22616 |  |  |
| Chieniodendron hainanense | 109.4663 | 19.21728 |  |  |
| Chionanthus ramiflorus | 109.0367 | 19.17511 | -21544.3 | 306313.7 |
| Chionanthus ramiflorus | 109.0628 | 19.12587 | -18969 | 300785.7 |
| Chionanthus ramiflorus | 109.2014 | 19.0858 | -4531.56 | 295912 |
| Chionanthus ramiflorus | 109.3683 | 19.19189 | 13342.08 | 307122.7 |
| Chionanthus ramiflorus | 109.7575 | 19.47974 | 55069.1 | 337810.7 |
| Chionanthus ramiflorus | 109.767 | 18.56463 |  |  |
| Chionanthus ramiflorus | 109.819 | 18.57538 |  |  |
| Chionanthus ramiflorus | 109.8625 | 18.60442 |  |  |
| Chionanthus ramiflorus | 109.467 | 19.21975 |  |  |
| Chionanthus ramiflorus | 109.3916 | 19.23518 |  |  |
| Chionanthus ramiflorus | 109.3528 | 19.21233 |  |  |
| Chionanthus ramiflorus | 110.0546 | 18.56204 |  |  |
| Chionanthus ramiflorus | 110.0824 | 18.5361 |  |  |
| Chionanthus ramiflorus | 110.0669 | 18.55187 |  |  |
| Chionanthus ramiflorus | 110.066 | 18.52508 |  |  |
| Chionanthus ramiflorus | 109.6591 | 18.2534 |  |  |
| Chionanthus ramiflorus | 109.5977 | 18.25604 |  |  |
| Chionanthus ramiflorus | 109.5931 | 18.22708 |  |  |
| Chionanthus ramiflorus | 109.7003 | 19.55476 |  |  |
| Chionanthus ramiflorus | 109.6044 | 19.58256 |  |  |
| Chionanthus ramiflorus | 109.9824 | 19.72925 |  |  |
| Chionanthus ramiflorus | 109.9914 | 19.72076 |  |  |
| Chionanthus ramiflorus | 109.2951 | 18.79412 |  |  |
| Chionanthus ramiflorus | 109.0641 | 18.53596 |  |  |
| Chionanthus ramiflorus | 109.8771 | 19.11643 |  |  |
| Chloranthus spicatus | 109.5425 | 19.03583 | 31162.38 | 289348.8 |
| Chloranthus spicatus | 110.2238 | 18.67552 | 101866.1 | 247657.4 |
| Chloranthus spicatus | 110.4256 | 18.80085 |  |  |
| Chloranthus spicatus | 110.4277 | 18.80597 |  |  |
| Chloranthus spicatus | 110.4311 | 18.79344 |  |  |
| Chloranthus spicatus | 110.4377 | 18.79833 |  |  |
| Chloranthus spicatus | 110.4368 | 18.80996 |  |  |
| Chloranthus spicatus | 110.4273 | 18.80964 |  |  |
| Chloranthus spicatus | 110.4393 | 18.80155 |  |  |
| Chloranthus spicatus | 110.4344 | 18.80658 |  |  |
| Chloranthus spicatus | 110.427 | 18.7962 |  |  |
| Chloranthus spicatus | 110.4325 | 18.80573 |  |  |
| Chloranthus spicatus | 110.3766 | 18.77604 |  |  |
| Chloranthus spicatus | 110.3695 | 18.77075 |  |  |
| Chloranthus spicatus | 110.3807 | 18.76829 |  |  |
| Chloranthus spicatus | 110.3826 | 18.77307 |  |  |
| Chloranthus spicatus | 110.4325 | 18.75732 |  |  |
| Chloranthus spicatus | 110.4228 | 18.78257 |  |  |
| Chloranthus spicatus | 110.4087 | 18.76263 |  |  |
| Chloranthus spicatus | 110.4042 | 18.75731 |  |  |
| Chloranthus spicatus | 110.4119 | 18.75557 |  |  |
| Chloranthus spicatus | 110.4363 | 18.76823 |  |  |
| Choerospondias axillaris | 109.0333 | 18.86036 | -22982.8 | 271527.7 |
| Choerospondias axillaris | 109.4914 | 19.41113 | 26959.92 | 330989.8 |
| Choerospondias axillaris | 109.5409 | 18.34239 | 28849.41 | 212669.7 |
| Choerospondias axillaris | 109.6579 | 18.76335 | 42477.13 | 258885.1 |
| Choerospondias axillaris | 110.0282 | 18.81462 | 81625.55 | 263539.8 |
| Choerospondias axillaris | 108.9744 | 19.17691 |  |  |
| Choerospondias axillaris | 109.5308 | 18.99951 |  |  |
| Choerospondias axillaris | 109.8009 | 19.0342 |  |  |
| Choerospondias axillaris | 109.5914 | 18.85321 |  |  |
| Choerospondias axillaris | 109.5747 | 18.90068 |  |  |
| Choerospondias axillaris | 110.035 | 18.71003 |  |  |
| Choerospondias axillaris | 109.225 | 19.25859 |  |  |
| Choerospondias axillaris | 109.8363 | 18.87768 |  |  |
| Choerospondias axillaris | 109.2762 | 18.95345 |  |  |
| Choerospondias axillaris | 109.1882 | 19.00425 |  |  |
| Choerospondias axillaris | 110.3732 | 19.24566 |  |  |
| Choerospondias axillaris | 109.868 | 19.18246 |  |  |
| Choerospondias axillaris | 110.096 | 19.40812 |  |  |
| Choerospondias axillaris | 109.7077 | 19.80819 |  |  |
| Choerospondias axillaris | 109.9916 | 19.71343 |  |  |
| Choerospondias axillaris | 110.2411 | 19.62698 |  |  |
| Choerospondias axillaris | 110.39 | 19.24912 |  |  |
| Choerospondias axillaris | 110.0953 | 19.72777 |  |  |
| Choerospondias axillaris | 110.1021 | 19.69788 |  |  |
| Choerospondias axillaris | 110.2136 | 19.723 |  |  |
| Chukrasia tabularis | 109.0323 | 19.15088 | -22080.8 | 303649.4 |
| Chukrasia tabularis | 109.0365 | 18.87698 | -22580.2 | 273355 |
| Chukrasia tabularis | 109.3011 | 18.85155 | 5180.119 | 269701.6 |
| Chukrasia tabularis | 109.4865 | 19.15807 | 25651.28 | 303028.8 |
| Chukrasia tabularis | 109.7707 | 18.56491 |  |  |
| Chukrasia tabularis | 109.8249 | 18.57896 |  |  |
| Chukrasia tabularis | 109.8563 | 18.60793 |  |  |
| Chukrasia tabularis | 109.467 | 19.21769 |  |  |
| Chukrasia tabularis | 109.3949 | 19.23494 |  |  |
| Chukrasia tabularis | 109.3453 | 19.21749 |  |  |
| Chukrasia tabularis | 110.0884 | 18.54685 |  |  |
| Chukrasia tabularis | 110.0859 | 18.51652 |  |  |
| Chukrasia tabularis | 110.0942 | 18.5273 |  |  |
| Chukrasia tabularis | 109.6588 | 18.25323 |  |  |
| Chukrasia tabularis | 109.5976 | 18.25653 |  |  |
| Chukrasia tabularis | 109.5922 | 18.22206 |  |  |
| Chukrasia tabularis | 109.6 | 18.22541 |  |  |
| Chukrasia tabularis | 109.593 | 18.22698 |  |  |
| Chukrasia tabularis | 109.5955 | 18.22578 |  |  |
| Chukrasia tabularis | 108.9855 | 19.28712 |  |  |
| Chukrasia tabularis | 109.0051 | 19.28118 |  |  |
| Chukrasia tabularis | 109.0232 | 19.28426 |  |  |
| Chukrasia tabularis | 108.9925 | 19.28372 |  |  |
| Chukrasia tabularis | 109.1374 | 19.31806 |  |  |
| Chuniophoenix hainanensis | 109.837 | 18.73456 | 61260.82 | 255199 |
| Chuniophoenix hainanensis | 109.838 | 18.73456 | 61366.18 | 255196.4 |
| Chuniophoenix hainanensis | 109.839 | 18.73456 | 61471.55 | 255193.7 |
| Chuniophoenix hainanensis | 109.84 | 18.73456 | 61576.91 | 255191.1 |
| Chuniophoenix hainanensis | 109.841 | 18.73456 | 61682.27 | 255188.5 |
| Chuniophoenix hainanensis | 109.877 | 18.79002 |  |  |
| Chuniophoenix hainanensis | 109.8651 | 18.79887 |  |  |
| Chuniophoenix hainanensis | 109.873 | 18.7861 |  |  |
| Chuniophoenix hainanensis | 109.8652 | 18.78768 |  |  |
| Chuniophoenix hainanensis | 109.2712 | 19.04572 |  |  |
| Chuniophoenix hainanensis | 109.256 | 19.03361 |  |  |
| Chuniophoenix hainanensis | 109.26 | 19.03752 |  |  |
| Chuniophoenix hainanensis | 109.2649 | 19.03493 |  |  |
| Chuniophoenix hainanensis | 109.2696 | 19.03863 |  |  |
| Chuniophoenix hainanensis | 109.2671 | 19.03973 |  |  |
| Chuniophoenix hainanensis | 109.8676 | 19.09526 |  |  |
| Chuniophoenix hainanensis | 109.8688 | 19.09574 |  |  |
| Chuniophoenix hainanensis | 109.869 | 19.09626 |  |  |
| Chuniophoenix hainanensis | 109.5474 | 19.03656 |  |  |
| Chuniophoenix hainanensis | 109.4718 | 19.39983 |  |  |
| Chuniophoenix hainanensis | 109.4557 | 19.39011 |  |  |
| Chuniophoenix hainanensis | 109.5425 | 19.02948 |  |  |
| Chuniophoenix hainanensis | 109.549 | 19.03213 |  |  |
| Chuniophoenix hainanensis | 109.5489 | 19.03779 |  |  |
| Chuniophoenix hainanensis | 109.5435 | 19.03688 |  |  |
| Cinnamomum bejolghota | 109.0333 | 18.86036 | -22982.8 | 271527.7 |
| Cinnamomum bejolghota | 109.1215 | 19.14164 | -12747.8 | 302339.7 |
| Cinnamomum bejolghota | 109.1592 | 19.12667 | -8839.36 | 300563.6 |
| Cinnamomum bejolghota | 109.3683 | 19.19189 | 13342.08 | 307122.7 |
| Cinnamomum bejolghota | 109.416 | 18.58215 | 16423.77 | 239559.1 |
| Cinnamomum bejolghota | 109.6835 | 18.87437 | 45503.17 | 271088.8 |
| Cinnamomum bejolghota | 109.6975 | 18.72833 | 46544.54 | 254900 |
| Cinnamomum bejolghota | 109.8844 | 18.79929 | 66438.38 | 262227.7 |
| Cinnamomum bejolghota | 110.0221 | 18.80753 | 80965.02 | 262772.1 |
| Cinnamomum bejolghota | 109.1839 | 18.95871 |  |  |
| Cinnamomum bejolghota | 109.4966 | 19.05542 |  |  |
| Cinnamomum bejolghota | 109.727 | 18.78555 |  |  |
| Cinnamomum bejolghota | 109.4435 | 18.96254 |  |  |
| Cinnamomum bejolghota | 109.1279 | 19.02106 |  |  |
| Cinnamomum bejolghota | 109.0504 | 18.87312 |  |  |
| Cinnamomum bejolghota | 109.7581 | 18.98092 |  |  |
| Cinnamomum bejolghota | 109.4096 | 19.24313 |  |  |
| Cinnamomum bejolghota | 109.7442 | 19.086 |  |  |
| Cinnamomum bejolghota | 109.8983 | 18.67566 |  |  |
| Cinnamomum bejolghota | 110.3931 | 19.80151 |  |  |
| Cinnamomum bejolghota | 110.1287 | 19.56639 |  |  |
| Cinnamomum bejolghota | 110.4735 | 19.29003 |  |  |
| Cinnamomum bejolghota | 109.8714 | 19.23483 |  |  |
| Cinnamomum bejolghota | 110.2257 | 19.43315 |  |  |
| Cinnamomum bejolghota | 109.9587 | 19.29337 |  |  |
| Cinnamomum bejolghota | 109.9148 | 19.78794 |  |  |
| Cinnamomum bejolghota | 110.0367 | 19.58593 |  |  |
| Cinnamomum bejolghota | 109.8575 | 19.35912 |  |  |
| Cinnamomum bejolghota | 110.0572 | 19.79454 |  |  |
| Cinnamomum burmannii | 108.7989 | 18.69619 | -48238.4 | 254162.2 |
| Cinnamomum burmannii | 108.9122 | 18.99917 | -35232.9 | 287275.8 |
| Cinnamomum burmannii | 109.1191 | 19.05985 | -13271 | 293304.6 |
| Cinnamomum burmannii | 109.1544 | 19.01211 | -9728.08 | 287914.8 |
| Cinnamomum burmannii | 109.414 | 19.57115 | 19347.16 | 348910 |
| Cinnamomum burmannii | 109.5064 | 18.29956 | 25081.02 | 208034 |
| Cinnamomum burmannii | 109.6579 | 18.76335 | 42477.13 | 258885.1 |
| Cinnamomum burmannii | 109.6964 | 19.03697 | 47343.87 | 289030.5 |
| Cinnamomum burmannii | 109.7426 | 19.4888 | 53537.07 | 338853.4 |
| Cinnamomum burmannii | 109.7575 | 19.47974 | 55069.1 | 337810.7 |
| Cinnamomum burmannii | 109.869 | 18.69141 | 64504.44 | 250339.5 |
| Cinnamomum burmannii | 108.9689 | 19.23785 |  |  |
| Cinnamomum burmannii | 109.4969 | 18.84163 |  |  |
| Cinnamomum burmannii | 109.8802 | 18.73754 |  |  |
| Cinnamomum burmannii | 108.9612 | 18.66035 |  |  |
| Cinnamomum burmannii | 108.9972 | 18.67725 |  |  |
| Cinnamomum burmannii | 109.0737 | 19.03644 |  |  |
| Cinnamomum burmannii | 109.6651 | 18.98425 |  |  |
| Cinnamomum burmannii | 109.0328 | 18.95807 |  |  |
| Cinnamomum burmannii | 109.0907 | 18.86759 |  |  |
| Cinnamomum burmannii | 109.3741 | 19.14998 |  |  |
| Cinnamomum burmannii | 110.2542 | 19.69559 |  |  |
| Cinnamomum burmannii | 110.3166 | 19.83713 |  |  |
| Cinnamomum burmannii | 109.8723 | 19.35718 |  |  |
| Cinnamomum burmannii | 110.3379 | 19.21033 |  |  |
| Cinnamomum burmannii | 110.1643 | 19.72757 |  |  |
| Cinnamomum burmannii | 109.7698 | 19.48486 |  |  |
| Cinnamomum burmannii | 110.3404 | 19.22506 |  |  |
| Cinnamomum burmannii | 110.0958 | 19.54681 |  |  |
| Cinnamomum burmannii | 109.695 | 19.60984 |  |  |
| Cinnamomum burmannii | 110.1729 | 19.61176 |  |  |
| Cinnamomum liangii | 109.0333 | 18.86036 | -22982.8 | 271527.7 |
| Cinnamomum liangii | 109.1191 | 19.05985 | -13271 | 293304.6 |
| Cinnamomum liangii | 109.5425 | 19.03583 | 31162.38 | 289348.8 |
| Cinnamomum liangii | 109.6975 | 18.72833 | 46544.54 | 254900 |
| Cinnamomum liangii | 109.6997 | 18.47449 | 46019.61 | 226820.3 |
| Cinnamomum liangii | 110.0605 | 18.52153 |  |  |
| Cinnamomum liangii | 110.0767 | 18.54392 |  |  |
| Cinnamomum liangii | 110.0997 | 18.51442 |  |  |
| Cinnamomum liangii | 110.0922 | 18.51283 |  |  |
| Cinnamomum liangii | 110.0815 | 18.55836 |  |  |
| Cinnamomum liangii | 110.0551 | 18.54566 |  |  |
| Cinnamomum liangii | 110.0787 | 18.52946 |  |  |
| Cinnamomum liangii | 110.0971 | 18.54012 |  |  |
| Cinnamomum liangii | 110.0757 | 18.52638 |  |  |
| Cinnamomum liangii | 110.0994 | 18.54824 |  |  |
| Cinnamomum liangii | 110.0791 | 18.55063 |  |  |
| Cinnamomum liangii | 110.1027 | 18.53956 |  |  |
| Cinnamomum liangii | 110.0951 | 18.52819 |  |  |
| Cinnamomum liangii | 110.0983 | 18.53727 |  |  |
| Cinnamomum liangii | 110.0773 | 18.52908 |  |  |
| Cinnamomum liangii | 110.0673 | 18.56187 |  |  |
| Cinnamomum liangii | 110.075 | 18.52111 |  |  |
| Cinnamomum liangii | 110.059 | 18.52563 |  |  |
| Cinnamomum liangii | 110.0933 | 18.54229 |  |  |
| Cinnamomum liangii | 110.0691 | 18.5559 |  |  |
| Cinnamomum micranthum | 109.6997 | 18.47449 | 46019.61 | 226820.3 |
| Cinnamomum micranthum | 109.3109 | 18.9205 |  |  |
| Cinnamomum micranthum | 109.5247 | 18.96926 |  |  |
| Cinnamomum micranthum | 109.4896 | 18.66291 |  |  |
| Cinnamomum micranthum | 109.6221 | 18.68111 |  |  |
| Cinnamomum micranthum | 109.7004 | 18.88003 |  |  |
| Cinnamomum micranthum | 109.5839 | 19.05409 |  |  |
| Cinnamomum micranthum | 109.081 | 18.72464 |  |  |
| Cinnamomum micranthum | 109.8054 | 19.18954 |  |  |
| Cinnamomum micranthum | 109.0597 | 19.07252 |  |  |
| Cinnamomum micranthum | 109.1343 | 19.24767 |  |  |
| Cinnamomum micranthum | 110.2315 | 19.52172 |  |  |
| Cinnamomum micranthum | 109.6009 | 19.54124 |  |  |
| Cinnamomum micranthum | 109.6866 | 19.25519 |  |  |
| Cinnamomum micranthum | 110.0939 | 19.72484 |  |  |
| Cinnamomum micranthum | 109.9445 | 19.70085 |  |  |
| Cinnamomum micranthum | 110.2762 | 19.63752 |  |  |
| Cinnamomum micranthum | 109.8672 | 19.67824 |  |  |
| Cinnamomum micranthum | 109.6042 | 19.70879 |  |  |
| Cinnamomum micranthum | 110.0626 | 19.84035 |  |  |
| Cinnamomum micranthum | 110.0852 | 19.5576 |  |  |
| Cissus assamica | 110.2256 | 19.86031 | 105147.4 | 378650.1 |
| Cissus assamica | 108.7411 | 19.0525 |  |  |
| Cissus assamica | 108.7457 | 19.06436 |  |  |
| Cissus assamica | 108.7876 | 18.97349 |  |  |
| Cissus assamica | 109.0471 | 18.6643 |  |  |
| Cissus assamica | 109.2967 | 18.79941 |  |  |
| Cissus assamica | 109.0928 | 18.54157 |  |  |
| Cissus assamica | 108.7433 | 19.05121 |  |  |
| Cissus assamica | 108.7444 | 19.0662 |  |  |
| Cissus assamica | 108.7859 | 18.97755 |  |  |
| Cissus assamica | 108.9969 | 18.72502 |  |  |
| Cissus assamica | 109.2834 | 18.77911 |  |  |
| Cissus assamica | 109.0376 | 18.55419 |  |  |
| Cissus assamica | 109.6505 | 18.25343 |  |  |
| Cissus assamica | 109.5983 | 18.26107 |  |  |
| Cissus assamica | 109.5994 | 18.22464 |  |  |
| Cissus assamica | 109.7721 | 18.55927 |  |  |
| Cissus assamica | 109.8344 | 18.5748 |  |  |
| Cissus assamica | 109.856 | 18.60344 |  |  |
| Cissus assamica | 110.0732 | 18.51508 |  |  |
| Cissus assamica | 110.1026 | 18.55831 |  |  |
| Cissus hexangularis | 108.9122 | 18.99917 | -35232.9 | 287275.8 |
| Cissus hexangularis | 108.9515 | 18.90932 | -31419.3 | 277210.7 |
| Cissus hexangularis | 109.1191 | 19.05985 | -13271 | 293304.6 |
| Cissus hexangularis | 109.1544 | 19.01211 | -9728.08 | 287914.8 |
| Cissus hexangularis | 109.3011 | 18.85155 | 5180.119 | 269701.6 |
| Cissus hexangularis | 109.3683 | 19.19189 | 13342.08 | 307122.7 |
| Cissus hexangularis | 109.4546 | 19.30628 | 22766.04 | 319508.9 |
| Cissus hexangularis | 109.5079 | 19.14711 | 27874.67 | 301753.8 |
| Cissus hexangularis | 109.5409 | 18.34239 | 28849.41 | 212669.7 |
| Cissus hexangularis | 109.6535 | 18.2575 | 40487.08 | 202951.5 |
| Cissus hexangularis | 109.7575 | 19.47974 | 55069.1 | 337810.7 |
| Cissus hexangularis | 109.9556 | 19.26192 | 75237.18 | 313189.7 |
| Cissus hexangularis | 110.1338 | 18.56051 | 92062.33 | 235164.3 |
| Cissus hexangularis | 109.0046 | 19.28997 |  |  |
| Cissus hexangularis | 109.0167 | 19.3016 |  |  |
| Cissus hexangularis | 108.9946 | 19.28018 |  |  |
| Cissus hexangularis | 109.0384 | 19.28443 |  |  |
| Cissus hexangularis | 109.0699 | 19.30912 |  |  |
| Cissus hexangularis | 109.1509 | 19.35395 |  |  |
| Cissus hexangularis | 109.1302 | 19.3378 |  |  |
| Cissus hexangularis | 108.7463 | 19.04986 |  |  |
| Cissus hexangularis | 108.7471 | 19.0648 |  |  |
| Cissus hexangularis | 108.7999 | 18.9578 |  |  |
| Cissus hexangularis | 109.0645 | 18.71171 |  |  |
| Cissus hexangularis | 109.2998 | 18.78418 |  |  |
| Cissus hexangularis | 109.1369 | 18.52064 |  |  |
| Cissus hexangularis | 109.4682 | 19.21791 |  |  |
| Cissus hexangularis | 109.3868 | 19.22964 |  |  |
| Cissus hexangularis | 109.3534 | 19.21376 |  |  |
| Cissus hexangularis | 109.813 | 18.94634 |  |  |
| Cissus hexangularis | 109.8146 | 19.02449 |  |  |
| Cissus hexangularis | 109.7762 | 18.97056 |  |  |
| Cissus hexangularis | 109.7769 | 19.08213 |  |  |
| Cissus pteroclada | 108.8974 | 18.5739 | -38293.2 | 240304.7 |
| Cissus pteroclada | 109.8844 | 18.79929 | 66438.38 | 262227.7 |
| Cissus pteroclada | 109.7574 | 19.05731 |  |  |
| Cissus pteroclada | 109.8871 | 19.04803 |  |  |
| Cissus pteroclada | 109.8465 | 19.01289 |  |  |
| Cissus pteroclada | 109.8827 | 18.95856 |  |  |
| Cissus pteroclada | 109.7704 | 19.01558 |  |  |
| Cissus pteroclada | 109.7215 | 19.01257 |  |  |
| Cissus pteroclada | 109.7518 | 19.10109 |  |  |
| Cissus pteroclada | 109.7925 | 18.96608 |  |  |
| Cissus pteroclada | 109.0646 | 18.70567 |  |  |
| Cissus pteroclada | 109.0361 | 18.66431 |  |  |
| Cissus pteroclada | 109.0336 | 18.69794 |  |  |
| Cissus pteroclada | 109.0646 | 18.67344 |  |  |
| Cissus pteroclada | 108.9969 | 18.69165 |  |  |
| Cissus pteroclada | 109.2936 | 18.80281 |  |  |
| Cissus pteroclada | 109.2902 | 18.79397 |  |  |
| Cissus pteroclada | 109.2803 | 18.78204 |  |  |
| Cissus pteroclada | 109.1024 | 18.59685 |  |  |
| Cissus pteroclada | 109.0544 | 18.55853 |  |  |
| Cissus pteroclada | 109.13 | 18.58813 |  |  |
| Cissus pteroclada | 109.1339 | 18.62222 |  |  |
| Cissus repens | 108.7989 | 18.69619 | -48238.4 | 254162.2 |
| Cissus repens | 109.4044 | 18.88006 | 16149.68 | 272536.9 |
| Cissus repens | 109.4176 | 19.08662 | 18194.62 | 295334.7 |
| Cissus repens | 109.6997 | 18.47449 | 46019.61 | 226820.3 |
| Cissus repens | 109.837 | 18.73456 | 61260.82 | 255199 |
| Cissus repens | 108.7374 | 19.04522 |  |  |
| Cissus repens | 108.7476 | 19.0638 |  |  |
| Cissus repens | 108.7886 | 18.96465 |  |  |
| Cissus repens | 109.0826 | 18.68843 |  |  |
| Cissus repens | 109.2771 | 18.79326 |  |  |
| Cissus repens | 109.1415 | 18.61133 |  |  |
| Cissus repens | 109.1468 | 18.53029 |  |  |
| Cissus repens | 109.1464 | 18.52004 |  |  |
| Cissus repens | 109.7787 | 18.56075 |  |  |
| Cissus repens | 109.8135 | 18.58462 |  |  |
| Cissus repens | 109.8515 | 18.60442 |  |  |
| Cissus repens | 109.0182 | 19.28821 |  |  |
| Cissus repens | 109.0078 | 19.28483 |  |  |
| Cissus repens | 108.9874 | 19.27842 |  |  |
| Cissus repens | 109.0145 | 19.29848 |  |  |
| Cissus repens | 109.1287 | 19.33545 |  |  |
| Cissus repens | 109.1123 | 19.3229 |  |  |
| Cissus repens | 109.0841 | 19.36007 |  |  |
| Cissus repens | 110.3749 | 18.77314 |  |  |
| Cissus repens | 110.413 | 18.75805 |  |  |
| Citrus japonica | 109.0333 | 18.86036 | -22982.8 | 271527.7 |
| Citrus japonica | 109.1191 | 19.05985 | -13271 | 293304.6 |
| Citrus japonica | 109.3736 | 18.89125 | 12945.56 | 273868.1 |
| Citrus japonica | 109.416 | 18.58215 | 16423.77 | 239559.1 |
| Citrus japonica | 109.6628 | 18.86803 | 43304.48 | 270446.3 |
| Citrus japonica | 110.001 | 18.75027 | 78585.68 | 256495.3 |
| Citrus japonica | 110.2559 | 19.09526 | 106327.7 | 293994.1 |
| Citrus japonica | 109.4042 | 19.07658 |  |  |
| Citrus japonica | 108.9963 | 18.84392 |  |  |
| Citrus japonica | 109.1932 | 18.78705 |  |  |
| Citrus japonica | 109.1536 | 18.78137 |  |  |
| Citrus japonica | 109.9082 | 19.0114 |  |  |
| Citrus japonica | 109.9415 | 18.70774 |  |  |
| Citrus japonica | 109.2451 | 18.9047 |  |  |
| Citrus japonica | 109.0794 | 19.08521 |  |  |
| Citrus japonica | 109.7924 | 18.96891 |  |  |
| Citrus japonica | 109.1606 | 19.07152 |  |  |
| Citrus japonica | 110.0766 | 19.43442 |  |  |
| Citrus japonica | 109.6069 | 19.30658 |  |  |
| Citrus japonica | 110.4343 | 19.79884 |  |  |
| Citrus japonica | 110.2155 | 19.24759 |  |  |
| Citrus japonica | 109.7116 | 19.22033 |  |  |
| Citrus japonica | 109.9315 | 19.52656 |  |  |
| Citrus japonica | 110.0177 | 19.5568 |  |  |
| Citrus japonica | 110.1722 | 19.46563 |  |  |
| Citrus japonica | 110.2736 | 19.68573 |  |  |
| Citrus japonica | 110.5085 | 19.207 |  |  |
| Claoxylon indicum | 108.9122 | 18.99917 | -35232.9 | 287275.8 |
| Claoxylon indicum | 109.3011 | 18.85155 | 5180.119 | 269701.6 |
| Claoxylon indicum | 109.5132 | 19.56355 | 29719.98 | 347774.6 |
| Claoxylon indicum | 109.6807 | 18.37829 | 43729.57 | 216234.2 |
| Claoxylon indicum | 110.1756 | 18.76435 | 97019.43 | 257602 |
| Claoxylon indicum | 110.2177 | 18.75379 | 101417.9 | 256328.6 |
| Claoxylon indicum | 109.7544 | 19.07458 |  |  |
| Claoxylon indicum | 108.935 | 18.70287 |  |  |
| Claoxylon indicum | 109.8538 | 18.83271 |  |  |
| Claoxylon indicum | 109.7519 | 18.84876 |  |  |
| Claoxylon indicum | 109.4099 | 19.00886 |  |  |
| Claoxylon indicum | 109.9565 | 18.70744 |  |  |
| Claoxylon indicum | 109.2208 | 19.06591 |  |  |
| Claoxylon indicum | 109.7625 | 19.25522 |  |  |
| Claoxylon indicum | 109.1916 | 19.19947 |  |  |
| Claoxylon indicum | 108.9869 | 18.88472 |  |  |
| Claoxylon indicum | 110.3507 | 19.28494 |  |  |
| Claoxylon indicum | 109.9789 | 19.53729 |  |  |
| Claoxylon indicum | 109.9765 | 19.37103 |  |  |
| Claoxylon indicum | 110.0401 | 19.73745 |  |  |
| Claoxylon indicum | 109.8003 | 19.69022 |  |  |
| Claoxylon indicum | 109.7288 | 19.47815 |  |  |
| Claoxylon indicum | 110.0025 | 19.36402 |  |  |
| Claoxylon indicum | 109.5885 | 19.71424 |  |  |
| Claoxylon indicum | 109.7157 | 19.60246 |  |  |
| Claoxylon indicum | 110.3767 | 19.17598 |  |  |
| Clausena excavata | 108.9444 | 18.88828 | -32234.3 | 274907.9 |
| Clausena excavata | 109.1544 | 19.01211 | -9728.08 | 287914.8 |
| Clausena excavata | 109.4044 | 18.88006 | 16149.68 | 272536.9 |
| Clausena excavata | 109.5146 | 18.9427 | 27935.88 | 279133.3 |
| Clausena excavata | 109.7575 | 19.47974 | 55069.1 | 337810.7 |
| Clausena excavata | 109.7752 | 18.56342 |  |  |
| Clausena excavata | 109.8125 | 18.58714 |  |  |
| Clausena excavata | 109.8659 | 18.60882 |  |  |
| Clausena excavata | 109.4667 | 19.21805 |  |  |
| Clausena excavata | 109.393 | 19.22949 |  |  |
| Clausena excavata | 109.3479 | 19.21741 |  |  |
| Clausena excavata | 110.0811 | 18.52049 |  |  |
| Clausena excavata | 110.0808 | 18.54813 |  |  |
| Clausena excavata | 110.082 | 18.54745 |  |  |
| Clausena excavata | 109.0058 | 18.75375 |  |  |
| Clausena excavata | 109.2794 | 18.78255 |  |  |
| Clausena excavata | 109.1164 | 18.55025 |  |  |
| Clausena excavata | 109.9976 | 19.72836 |  |  |
| Clausena excavata | 110.0294 | 19.72894 |  |  |
| Clausena excavata | 110.0086 | 19.78187 |  |  |
| Clausena excavata | 110.38 | 18.77067 |  |  |
| Clausena excavata | 110.4084 | 18.77376 |  |  |
| Clausena excavata | 110.4396 | 18.79461 |  |  |
| Clausena excavata | 110.4246 | 18.8135 |  |  |
| Clausena excavata | 110.4297 | 18.80167 |  |  |
| Clausena lenis | 108.8974 | 18.5739 | -38293.2 | 240304.7 |
| Clausena lenis | 109.5186 | 18.25822 | 26241.94 | 203424.4 |
| Clausena lenis | 109.6997 | 18.47449 | 46019.61 | 226820.3 |
| Clausena lenis | 109.7805 | 18.56903 |  |  |
| Clausena lenis | 109.8299 | 18.57865 |  |  |
| Clausena lenis | 109.8532 | 18.6017 |  |  |
| Clausena lenis | 109.7683 | 18.56753 |  |  |
| Clausena lenis | 109.8144 | 18.57329 |  |  |
| Clausena lenis | 109.8693 | 18.59615 |  |  |
| Clausena lenis | 109.6509 | 18.25327 |  |  |
| Clausena lenis | 109.5923 | 18.26063 |  |  |
| Clausena lenis | 109.5969 | 18.22685 |  |  |
| Clausena lenis | 109.6566 | 18.25203 |  |  |
| Clausena lenis | 109.5929 | 18.25813 |  |  |
| Clausena lenis | 109.5908 | 18.22417 |  |  |
| Clausena lenis | 110.0705 | 18.55752 |  |  |
| Clausena lenis | 110.1008 | 18.54093 |  |  |
| Clausena lenis | 110.0864 | 18.51598 |  |  |
| Clausena lenis | 110.0879 | 18.52734 |  |  |
| Clausena lenis | 110.0812 | 18.52843 |  |  |
| Clausena lenis | 110.0672 | 18.55366 |  |  |
| Clausena lenis | 110.0807 | 18.54104 |  |  |
| Clausena lenis | 110.0601 | 18.56038 |  |  |
| Cleidion brevipetiolatum | 108.9515 | 18.90932 | -31419.3 | 277210.7 |
| Cleidion brevipetiolatum | 109.0333 | 18.86036 | -22982.8 | 271527.7 |
| Cleidion brevipetiolatum | 109.1544 | 19.01211 | -9728.08 | 287914.8 |
| Cleidion brevipetiolatum | 109.4044 | 18.88006 | 16149.68 | 272536.9 |
| Cleidion brevipetiolatum | 109.6997 | 18.47449 | 46019.61 | 226820.3 |
| Cleidion brevipetiolatum | 110.0732 | 18.51897 |  |  |
| Cleidion brevipetiolatum | 110.1006 | 18.53813 |  |  |
| Cleidion brevipetiolatum | 110.0753 | 18.52942 |  |  |
| Cleidion brevipetiolatum | 110.0763 | 18.54923 |  |  |
| Cleidion brevipetiolatum | 110.0756 | 18.53211 |  |  |
| Cleidion brevipetiolatum | 110.0912 | 18.54943 |  |  |
| Cleidion brevipetiolatum | 110.0708 | 18.52129 |  |  |
| Cleidion brevipetiolatum | 110.0663 | 18.53584 |  |  |
| Cleidion brevipetiolatum | 108.7446 | 19.04136 |  |  |
| Cleidion brevipetiolatum | 108.7449 | 19.06569 |  |  |
| Cleidion brevipetiolatum | 108.8078 | 18.97085 |  |  |
| Cleidion brevipetiolatum | 109.0147 | 18.69326 |  |  |
| Cleidion brevipetiolatum | 109.2964 | 18.79217 |  |  |
| Cleidion brevipetiolatum | 109.1465 | 18.60445 |  |  |
| Cleidion brevipetiolatum | 109.0886 | 18.60245 |  |  |
| Cleidion brevipetiolatum | 109.0611 | 18.59812 |  |  |
| Cleidion brevipetiolatum | 109.0685 | 18.52677 |  |  |
| Cleidion brevipetiolatum | 108.8087 | 18.98459 |  |  |
| Cleidion brevipetiolatum | 108.7851 | 18.99058 |  |  |
| Cleidion brevipetiolatum | 108.7908 | 18.95674 |  |  |
| Cleistanthus concinnus | 109.0333 | 18.86036 | -22982.8 | 271527.7 |
| Cleistanthus concinnus | 109.0365 | 18.87698 | -22580.2 | 273355 |
| Cleistanthus concinnus | 109.1191 | 19.05985 | -13271 | 293304.6 |
| Cleistanthus concinnus | 108.74 | 19.04939 |  |  |
| Cleistanthus concinnus | 108.7446 | 19.06357 |  |  |
| Cleistanthus concinnus | 108.7921 | 18.96339 |  |  |
| Cleistanthus concinnus | 109.0567 | 18.72102 |  |  |
| Cleistanthus concinnus | 109.2952 | 18.79689 |  |  |
| Cleistanthus concinnus | 109.1232 | 18.53363 |  |  |
| Cleistanthus concinnus | 109.0364 | 18.56696 |  |  |
| Cleistanthus concinnus | 109.1019 | 18.57592 |  |  |
| Cleistanthus concinnus | 109.124 | 18.53744 |  |  |
| Cleistanthus concinnus | 109.1075 | 18.61874 |  |  |
| Cleistanthus concinnus | 109.002 | 19.29842 |  |  |
| Cleistanthus concinnus | 109.0094 | 19.28524 |  |  |
| Cleistanthus concinnus | 109.0255 | 19.30286 |  |  |
| Cleistanthus concinnus | 108.9885 | 19.29352 |  |  |
| Cleistanthus concinnus | 109.156 | 19.35388 |  |  |
| Cleistanthus concinnus | 109.1391 | 19.36952 |  |  |
| Cleistanthus concinnus | 109.1006 | 19.32637 |  |  |
| Cleistanthus concinnus | 109.0992 | 19.30813 |  |  |
| Cleistanthus concinnus | 109.1012 | 19.31971 |  |  |
| Cleistanthus concinnus | 109.1099 | 19.35931 |  |  |
| Cleistanthus sumatranus | 108.9122 | 18.99917 | -35232.9 | 287275.8 |
| Cleistanthus sumatranus | 109.3011 | 18.85155 | 5180.119 | 269701.6 |
| Cleistanthus sumatranus | 109.4044 | 18.88006 | 16149.68 | 272536.9 |
| Cleistanthus sumatranus | 109.6975 | 18.72833 | 46544.54 | 254900 |
| Cleistanthus sumatranus | 109.837 | 18.73456 | 61260.82 | 255199 |
| Cleistanthus sumatranus | 110.2356 | 18.69662 | 103162.5 | 249961.2 |
| Cleistanthus sumatranus | 110.4321 | 19.37563 | 125548.1 | 324570.5 |
| Cleistanthus sumatranus | 108.7455 | 19.051 |  |  |
| Cleistanthus sumatranus | 108.7455 | 19.06475 |  |  |
| Cleistanthus sumatranus | 108.8108 | 18.98465 |  |  |
| Cleistanthus sumatranus | 109.7638 | 18.56179 |  |  |
| Cleistanthus sumatranus | 109.8175 | 18.57661 |  |  |
| Cleistanthus sumatranus | 109.8607 | 18.60616 |  |  |
| Cleistanthus sumatranus | 109.651 | 18.25221 |  |  |
| Cleistanthus sumatranus | 109.6009 | 18.25575 |  |  |
| Cleistanthus sumatranus | 109.5993 | 18.22341 |  |  |
| Cleistanthus sumatranus | 110.3654 | 19.32149 |  |  |
| Cleistanthus sumatranus | 110.3773 | 19.31423 |  |  |
| Cleistanthus sumatranus | 110.4089 | 19.33172 |  |  |
| Cleistanthus sumatranus | 110.4 | 19.33269 |  |  |
| Cleistanthus sumatranus | 110.4065 | 19.31121 |  |  |
| Cleistanthus sumatranus | 110.5522 | 19.22663 |  |  |
| Cleistanthus sumatranus | 110.5631 | 19.23918 |  |  |
| Cleistanthus sumatranus | 110.3822 | 18.77604 |  |  |
| Cleistanthus sumatranus | 110.4309 | 18.7582 |  |  |
| Cleistanthus sumatranus | 110.4345 | 18.80775 |  |  |
| Cleistanthus sumatranus | 110.426 | 18.79462 |  |  |
| Clerodendrum bungei | 109.1215 | 19.14164 | -12747.8 | 302339.7 |
| Clerodendrum bungei | 109.1215 | 19.14164 | -12747.7 | 302339.8 |
| Clerodendrum bungei | 109.1215 | 19.14164 | -12747.5 | 302339.9 |
| Clerodendrum bungei | 109.1215 | 19.14165 | -12747.4 | 302340 |
| Clerodendrum bungei | 109.1215 | 19.14165 | -12747.3 | 302340.1 |
| Clerodendrum bungei | 109.0103 | 18.85963 |  |  |
| Clerodendrum bungei | 109.767 | 19.03681 |  |  |
| Clerodendrum bungei | 109.9811 | 18.68314 |  |  |
| Clerodendrum bungei | 109.2266 | 19.18148 |  |  |
| Clerodendrum bungei | 109.3854 | 18.85642 |  |  |
| Clerodendrum bungei | 109.9477 | 18.94507 |  |  |
| Clerodendrum bungei | 109.8192 | 18.80193 |  |  |
| Clerodendrum bungei | 109.3521 | 19.0689 |  |  |
| Clerodendrum bungei | 109.0896 | 18.72757 |  |  |
| Clerodendrum bungei | 109.9718 | 18.7838 |  |  |
| Clerodendrum bungei | 110.129 | 19.521 |  |  |
| Clerodendrum bungei | 110.2531 | 19.17992 |  |  |
| Clerodendrum bungei | 109.6129 | 19.26316 |  |  |
| Clerodendrum bungei | 110.2462 | 19.23539 |  |  |
| Clerodendrum bungei | 109.6367 | 19.30022 |  |  |
| Clerodendrum bungei | 110.2441 | 19.25699 |  |  |
| Clerodendrum bungei | 110.4988 | 19.64986 |  |  |
| Clerodendrum bungei | 110.4368 | 19.37973 |  |  |
| Clerodendrum bungei | 110.2609 | 19.47269 |  |  |
| Clerodendrum bungei | 109.9081 | 19.79062 |  |  |
| Clerodendrum canescens | 109.5146 | 18.9427 | 27935.88 | 279133.3 |
| Clerodendrum canescens | 109.6579 | 18.76335 | 42477.13 | 258885.1 |
| Clerodendrum canescens | 110.1756 | 18.76435 | 97019.43 | 257602 |
| Clerodendrum canescens | 110.2356 | 18.69662 | 103162.5 | 249961.2 |
| Clerodendrum canescens | 110.7217 | 19.71514 | 156705.5 | 361456.5 |
| Clerodendrum canescens | 108.9337 | 18.77519 |  |  |
| Clerodendrum canescens | 109.4232 | 19.01138 |  |  |
| Clerodendrum canescens | 109.9145 | 18.66315 |  |  |
| Clerodendrum canescens | 109.0122 | 19.13044 |  |  |
| Clerodendrum canescens | 109.1686 | 19.03534 |  |  |
| Clerodendrum canescens | 109.2729 | 19.07413 |  |  |
| Clerodendrum canescens | 109.9004 | 18.68191 |  |  |
| Clerodendrum canescens | 109.1683 | 18.70347 |  |  |
| Clerodendrum canescens | 109.6242 | 18.94268 |  |  |
| Clerodendrum canescens | 109.0959 | 18.94508 |  |  |
| Clerodendrum canescens | 109.8261 | 19.57334 |  |  |
| Clerodendrum canescens | 110.1075 | 19.63563 |  |  |
| Clerodendrum canescens | 109.7215 | 19.51562 |  |  |
| Clerodendrum canescens | 109.8536 | 19.74318 |  |  |
| Clerodendrum canescens | 109.9903 | 19.34368 |  |  |
| Clerodendrum canescens | 109.5865 | 19.23619 |  |  |
| Clerodendrum canescens | 109.5922 | 19.50436 |  |  |
| Clerodendrum canescens | 109.5989 | 19.49297 |  |  |
| Clerodendrum canescens | 109.6809 | 19.45006 |  |  |
| Clerodendrum canescens | 110.1112 | 19.40059 |  |  |
| Clerodendrum chinense | 109.6579 | 18.76335 | 42477.13 | 258885.1 |
| Clerodendrum chinense | 109.9968 | 19.7158 |  |  |
| Clerodendrum chinense | 109.9655 | 19.77126 |  |  |
| Clerodendrum chinense | 109.9942 | 19.74437 |  |  |
| Clerodendrum chinense | 110.0125 | 19.76108 |  |  |
| Clerodendrum chinense | 109.9457 | 19.76404 |  |  |
| Clerodendrum chinense | 109.9929 | 19.71061 |  |  |
| Clerodendrum chinense | 110.0368 | 19.79745 |  |  |
| Clerodendrum chinense | 109.9637 | 19.75057 |  |  |
| Clerodendrum chinense | 109.7828 | 18.56791 |  |  |
| Clerodendrum chinense | 109.8227 | 18.58053 |  |  |
| Clerodendrum chinense | 109.8548 | 18.60491 |  |  |
| Clerodendrum chinense | 109.7696 | 18.56406 |  |  |
| Clerodendrum chinense | 109.8237 | 18.57487 |  |  |
| Clerodendrum chinense | 109.8687 | 18.59882 |  |  |
| Clerodendrum chinense | 109.7663 | 18.56108 |  |  |
| Clerodendrum chinense | 109.8144 | 18.58393 |  |  |
| Clerodendrum chinense | 109.8618 | 18.60843 |  |  |
| Clerodendrum chinense | 109.7768 | 18.56768 |  |  |
| Clerodendrum chinense | 109.8277 | 18.57483 |  |  |
| Clerodendrum chinense | 109.8646 | 18.59798 |  |  |
| Clerodendrum cyrtophyllum | 109.1199 | 18.9577 | -13534.4 | 282008.9 |
| Clerodendrum cyrtophyllum | 109.3011 | 18.85155 | 5180.119 | 269701.6 |
| Clerodendrum cyrtophyllum | 109.3442 | 19.68663 | 12404.3 | 361885.9 |
| Clerodendrum cyrtophyllum | 109.4273 | 19.01928 | 18993.09 | 287860.9 |
| Clerodendrum cyrtophyllum | 109.4916 | 18.78671 | 25037.28 | 261953.3 |
| Clerodendrum cyrtophyllum | 109.5146 | 18.9427 | 27935.88 | 279133.3 |
| Clerodendrum cyrtophyllum | 109.6555 | 18.5381 | 41546.81 | 233982.1 |
| Clerodendrum cyrtophyllum | 109.6807 | 18.37829 | 43729.57 | 216234.2 |
| Clerodendrum cyrtophyllum | 109.837 | 18.73456 | 61260.82 | 255199 |
| Clerodendrum cyrtophyllum | 110.0473 | 18.44082 | 82618.44 | 222148.5 |
| Clerodendrum cyrtophyllum | 110.2738 | 18.67292 | 107120.4 | 247246.1 |
| Clerodendrum cyrtophyllum | 110.475 | 19.15751 | 129516.1 | 300354 |
| Clerodendrum cyrtophyllum | 110.7217 | 19.71514 | 156705.5 | 361456.5 |
| Clerodendrum cyrtophyllum | 109.9908 | 19.75178 |  |  |
| Clerodendrum cyrtophyllum | 110.0187 | 19.81537 |  |  |
| Clerodendrum cyrtophyllum | 110.0669 | 19.79068 |  |  |
| Clerodendrum cyrtophyllum | 110.0782 | 19.77671 |  |  |
| Clerodendrum cyrtophyllum | 109.9922 | 19.76965 |  |  |
| Clerodendrum cyrtophyllum | 109.9817 | 19.72639 |  |  |
| Clerodendrum cyrtophyllum | 109.9793 | 19.78277 |  |  |
| Clerodendrum cyrtophyllum | 109.9566 | 19.71642 |  |  |
| Clerodendrum cyrtophyllum | 109.7768 | 18.5729 |  |  |
| Clerodendrum cyrtophyllum | 109.8222 | 18.58537 |  |  |
| Clerodendrum cyrtophyllum | 109.8589 | 18.60802 |  |  |
| Clerodendrum cyrtophyllum | 109.7833 | 18.56698 |  |  |
| Clerodendrum cyrtophyllum | 109.8228 | 18.57615 |  |  |
| Clerodendrum cyrtophyllum | 109.8713 | 18.6006 |  |  |
| Clerodendrum cyrtophyllum | 109.7786 | 18.56189 |  |  |
| Clerodendrum cyrtophyllum | 109.8224 | 18.58047 |  |  |
| Clerodendrum cyrtophyllum | 109.8561 | 18.60835 |  |  |
| Clerodendrum cyrtophyllum | 109.7777 | 18.56863 |  |  |
| Clerodendrum cyrtophyllum | 109.8157 | 18.5805 |  |  |
| Clerodendrum cyrtophyllum | 109.8527 | 18.59792 |  |  |
| Clerodendrum fortunatum | 110.1876 | 19.13499 | 99259.69 | 298557.2 |
| Clerodendrum fortunatum | 110.3487 | 19.26196 | 116503.7 | 312200.6 |
| Clerodendrum fortunatum | 110.4758 | 19.2591 | 129844.1 | 311584.8 |
| Clerodendrum fortunatum | 109.7834 | 18.57089 |  |  |
| Clerodendrum fortunatum | 109.8319 | 18.58288 |  |  |
| Clerodendrum fortunatum | 109.8608 | 18.59622 |  |  |
| Clerodendrum fortunatum | 109.7635 | 18.56737 |  |  |
| Clerodendrum fortunatum | 109.8344 | 18.58678 |  |  |
| Clerodendrum fortunatum | 109.86 | 18.60505 |  |  |
| Clerodendrum fortunatum | 109.7729 | 18.56299 |  |  |
| Clerodendrum fortunatum | 109.8202 | 18.57523 |  |  |
| Clerodendrum fortunatum | 109.8509 | 18.60687 |  |  |
| Clerodendrum fortunatum | 110.4028 | 19.29375 |  |  |
| Clerodendrum fortunatum | 110.4161 | 19.28884 |  |  |
| Clerodendrum fortunatum | 110.4236 | 19.30667 |  |  |
| Clerodendrum fortunatum | 110.4119 | 19.30814 |  |  |
| Clerodendrum fortunatum | 110.4166 | 19.30153 |  |  |
| Clerodendrum fortunatum | 110.6063 | 19.25766 |  |  |
| Clerodendrum fortunatum | 110.5978 | 19.23397 |  |  |
| Clerodendrum fortunatum | 110.5555 | 19.27638 |  |  |
| Clerodendrum fortunatum | 110.5665 | 19.25785 |  |  |
| Clerodendrum fortunatum | 110.5821 | 19.23564 |  |  |
| Clerodendrum fortunatum | 110.5683 | 19.25701 |  |  |
| Clerodendrum japonicum | 109.1215 | 19.14164 | -12747.8 | 302339.7 |
| Clerodendrum japonicum | 109.5153 | 18.61925 | 27012 | 243364.4 |
| Clerodendrum japonicum | 109.6271 | 19.59807 | 41761.54 | 351259.2 |
| Clerodendrum japonicum | 109.7426 | 19.4888 | 53537.07 | 338853.4 |
| Clerodendrum japonicum | 109.7575 | 19.47974 | 55069.1 | 337810.7 |
| Clerodendrum japonicum | 109.7768 | 18.56389 |  |  |
| Clerodendrum japonicum | 109.8228 | 18.57539 |  |  |
| Clerodendrum japonicum | 109.8502 | 18.5992 |  |  |
| Clerodendrum japonicum | 109.4672 | 19.21692 |  |  |
| Clerodendrum japonicum | 109.3878 | 19.23088 |  |  |
| Clerodendrum japonicum | 109.3492 | 19.21261 |  |  |
| Clerodendrum japonicum | 110.0928 | 18.5221 |  |  |
| Clerodendrum japonicum | 110.0945 | 18.52984 |  |  |
| Clerodendrum japonicum | 110.0747 | 18.55324 |  |  |
| Clerodendrum japonicum | 110.1052 | 18.56217 |  |  |
| Clerodendrum japonicum | 109.6571 | 18.25187 |  |  |
| Clerodendrum japonicum | 109.5929 | 18.26224 |  |  |
| Clerodendrum japonicum | 109.5952 | 18.22407 |  |  |
| Clerodendrum japonicum | 110.0133 | 19.76686 |  |  |
| Clerodendrum japonicum | 110.0567 | 19.73127 |  |  |
| Clerodendrum japonicum | 109.7296 | 19.55239 |  |  |
| Clerodendrum japonicum | 109.6199 | 19.57448 |  |  |
| Clerodendrum japonicum | 110.8157 | 19.55418 |  |  |
| Clerodendrum japonicum | 110.432 | 18.80241 |  |  |
| Clerodendrum japonicum | 110.933 | 19.66783 |  |  |
| Clerodendrum lindleyi | 109.6516 | 18.61501 | 41369.75 | 242499.1 |
| Clerodendrum lindleyi | 109.7575 | 19.47974 | 55069.1 | 337810.7 |
| Clerodendrum lindleyi | 110.0102 | 19.56902 | 81818.27 | 346997.2 |
| Clerodendrum lindleyi | 110.0221 | 18.80753 | 80965.02 | 262772.1 |
| Clerodendrum lindleyi | 110.2256 | 19.86031 | 105147.4 | 378650.1 |
| Clerodendrum lindleyi | 109.0914 | 18.77047 |  |  |
| Clerodendrum lindleyi | 108.9879 | 18.8486 |  |  |
| Clerodendrum lindleyi | 109.8353 | 19.08226 |  |  |
| Clerodendrum lindleyi | 109.6786 | 18.83656 |  |  |
| Clerodendrum lindleyi | 108.9583 | 19.24406 |  |  |
| Clerodendrum lindleyi | 109.552 | 19.13131 |  |  |
| Clerodendrum lindleyi | 110.0472 | 18.91874 |  |  |
| Clerodendrum lindleyi | 109.7761 | 19.21987 |  |  |
| Clerodendrum lindleyi | 109.8666 | 18.97978 |  |  |
| Clerodendrum lindleyi | 109.106 | 18.71675 |  |  |
| Clerodendrum lindleyi | 110.2263 | 19.64293 |  |  |
| Clerodendrum lindleyi | 109.9264 | 19.22834 |  |  |
| Clerodendrum lindleyi | 109.6968 | 19.38078 |  |  |
| Clerodendrum lindleyi | 109.6131 | 19.80622 |  |  |
| Clerodendrum lindleyi | 109.6999 | 19.5374 |  |  |
| Clerodendrum lindleyi | 109.7699 | 19.25953 |  |  |
| Clerodendrum lindleyi | 109.7673 | 19.38452 |  |  |
| Clerodendrum lindleyi | 110.5018 | 19.39297 |  |  |
| Clerodendrum lindleyi | 109.981 | 19.17775 |  |  |
| Clerodendrum lindleyi | 110.0767 | 19.34395 |  |  |
| Cocculus laurifolius | 109.0333 | 18.86036 | -22982.8 | 271527.7 |
| Cocculus laurifolius | 109.1544 | 19.01211 | -9728.08 | 287914.8 |
| Cocculus laurifolius | 110.1206 | 19.04626 | 91979.26 | 288915.8 |
| Cocculus laurifolius | 109.7666 | 18.56879 |  |  |
| Cocculus laurifolius | 109.8201 | 18.58615 |  |  |
| Cocculus laurifolius | 109.8656 | 18.60151 |  |  |
| Cocculus laurifolius | 109.4697 | 19.21799 |  |  |
| Cocculus laurifolius | 109.3863 | 19.23602 |  |  |
| Cocculus laurifolius | 109.3502 | 19.21749 |  |  |
| Cocculus laurifolius | 109.7815 | 18.56713 |  |  |
| Cocculus laurifolius | 109.8222 | 18.57861 |  |  |
| Cocculus laurifolius | 109.8576 | 18.5997 |  |  |
| Cocculus laurifolius | 109.4709 | 19.22003 |  |  |
| Cocculus laurifolius | 109.3888 | 19.23244 |  |  |
| Cocculus laurifolius | 109.3452 | 19.21641 |  |  |
| Cocculus laurifolius | 108.7437 | 19.0446 |  |  |
| Cocculus laurifolius | 108.7443 | 19.06513 |  |  |
| Cocculus laurifolius | 108.7984 | 18.98232 |  |  |
| Cocculus laurifolius | 109.0696 | 18.74602 |  |  |
| Cocculus laurifolius | 109.277 | 18.80278 |  |  |
| Cocculus laurifolius | 109.1301 | 18.58428 |  |  |
| Cocculus laurifolius | 109.04 | 18.52624 |  |  |
| Cocculus laurifolius | 109.1419 | 18.56047 |  |  |
| Cocos nucifera | 109.1215 | 19.14164 | -12747.8 | 302339.7 |
| Cocos nucifera | 109.3011 | 18.85155 | 5180.119 | 269701.6 |
| Cocos nucifera | 109.3163 | 18.42284 | 5389.832 | 222245.7 |
| Cocos nucifera | 110.0437 | 18.51229 | 82426.67 | 230063.3 |
| Cocos nucifera | 109.9854 | 18.7299 |  |  |
| Cocos nucifera | 109.5419 | 18.76536 |  |  |
| Cocos nucifera | 109.129 | 19.07088 |  |  |
| Cocos nucifera | 109.0009 | 18.8079 |  |  |
| Cocos nucifera | 109.1757 | 18.80397 |  |  |
| Cocos nucifera | 109.1094 | 19.12939 |  |  |
| Cocos nucifera | 109.2738 | 18.89907 |  |  |
| Cocos nucifera | 109.0854 | 18.93692 |  |  |
| Cocos nucifera | 109.0338 | 19.05998 |  |  |
| Cocos nucifera | 109.8907 | 18.78442 |  |  |
| Cocos nucifera | 110.4659 | 19.58594 |  |  |
| Cocos nucifera | 110.1977 | 19.487 |  |  |
| Cocos nucifera | 110.0338 | 19.26432 |  |  |
| Cocos nucifera | 109.6668 | 19.53385 |  |  |
| Cocos nucifera | 110.2651 | 19.6092 |  |  |
| Cocos nucifera | 110.2235 | 19.60166 |  |  |
| Cocos nucifera | 109.8855 | 19.20729 |  |  |
| Cocos nucifera | 110.4917 | 19.45134 |  |  |
| Cocos nucifera | 110.0874 | 19.77802 |  |  |
| Cocos nucifera | 110.2413 | 19.38791 |  |  |
| Colubrina asiatica | 109.2292 | 18.32242 | -4135.79 | 211411.6 |
| Colubrina asiatica | 109.5106 | 18.24137 | 25337.66 | 201585.1 |
| Colubrina asiatica | 110.2177 | 18.75379 | 101417.9 | 256328.6 |
| Colubrina asiatica | 110.2332 | 18.6893 | 102892.4 | 249157.1 |
| Colubrina asiatica | 110.577 | 19.95677 | 142122.3 | 388485.7 |
| Colubrina asiatica | 110.0757 | 18.54945 |  |  |
| Colubrina asiatica | 110.0789 | 18.55905 |  |  |
| Colubrina asiatica | 110.1024 | 18.55609 |  |  |
| Colubrina asiatica | 110.0825 | 18.56071 |  |  |
| Colubrina asiatica | 110.1054 | 18.51988 |  |  |
| Colubrina asiatica | 110.0759 | 18.52501 |  |  |
| Colubrina asiatica | 110.0946 | 18.52555 |  |  |
| Colubrina asiatica | 109.0643 | 18.67973 |  |  |
| Colubrina asiatica | 109.2847 | 18.77746 |  |  |
| Colubrina asiatica | 109.1483 | 18.52214 |  |  |
| Colubrina asiatica | 108.9937 | 18.73779 |  |  |
| Colubrina asiatica | 109.2963 | 18.7867 |  |  |
| Colubrina asiatica | 109.1096 | 18.61266 |  |  |
| Colubrina asiatica | 109.6518 | 18.25572 |  |  |
| Colubrina asiatica | 109.5979 | 18.2617 |  |  |
| Colubrina asiatica | 109.5977 | 18.22198 |  |  |
| Colubrina asiatica | 109.6556 | 18.25476 |  |  |
| Colubrina asiatica | 109.5987 | 18.25873 |  |  |
| Colubrina asiatica | 109.5959 | 18.22288 |  |  |
| Colubrina asiatica | 109.5981 | 18.22369 |  |  |
| Combretum alfredii | 109.1741 | 18.37558 | -9781.56 | 217466.3 |
| Combretum alfredii | 109.3233 | 18.69505 | 7006.586 | 252327.2 |
| Combretum alfredii | 109.4548 | 19.18904 | 22421.09 | 306546.7 |
| Combretum alfredii | 109.5146 | 18.9427 | 27935.88 | 279133.3 |
| Combretum alfredii | 109.8628 | 18.65961 | 63764.9 | 246839.6 |
| Combretum alfredii | 110.2356 | 18.69662 | 103162.5 | 249961.2 |
| Combretum alfredii | 109.7674 | 18.56225 |  |  |
| Combretum alfredii | 109.8176 | 18.58668 |  |  |
| Combretum alfredii | 109.8662 | 18.60221 |  |  |
| Combretum alfredii | 109.4685 | 19.21663 |  |  |
| Combretum alfredii | 109.3922 | 19.2324 |  |  |
| Combretum alfredii | 109.3496 | 19.21166 |  |  |
| Combretum alfredii | 110.1044 | 18.51349 |  |  |
| Combretum alfredii | 110.0673 | 18.52227 |  |  |
| Combretum alfredii | 110.0607 | 18.5135 |  |  |
| Combretum alfredii | 110.0892 | 18.53712 |  |  |
| Combretum alfredii | 108.7408 | 19.04035 |  |  |
| Combretum alfredii | 108.746 | 19.06585 |  |  |
| Combretum alfredii | 108.8114 | 18.97031 |  |  |
| Combretum alfredii | 109.0835 | 18.71865 |  |  |
| Combretum alfredii | 109.293 | 18.78522 |  |  |
| Combretum alfredii | 109.1255 | 18.53641 |  |  |
| Combretum alfredii | 109.6512 | 18.25599 |  |  |
| Combretum alfredii | 109.601 | 18.25728 |  |  |
| Combretum alfredii | 109.5936 | 18.22174 |  |  |
| Combretum alfredii | 109.1466 | 19.3181 |  |  |
| Combretum punctatum | 109.5259 | 19.13355 | 29723.31 | 300201.6 |
| Combretum punctatum | 109.7426 | 19.4888 | 53537.07 | 338853.4 |
| Combretum punctatum | 109.7426 | 19.4888 | 53537.18 | 338853.5 |
| Combretum punctatum | 109.7426 | 19.4888 | 53537.28 | 338853.6 |
| Combretum punctatum | 109.8844 | 18.79929 | 66438.38 | 262227.7 |
| Combretum punctatum | 109.7676 | 19.11912 |  |  |
| Combretum punctatum | 109.7498 | 19.05276 |  |  |
| Combretum punctatum | 109.7385 | 18.95344 |  |  |
| Combretum punctatum | 109.7285 | 18.95993 |  |  |
| Combretum punctatum | 109.7374 | 19.01805 |  |  |
| Combretum punctatum | 109.7937 | 19.05923 |  |  |
| Combretum punctatum | 109.7866 | 19.08139 |  |  |
| Combretum punctatum | 109.7427 | 19.06338 |  |  |
| Combretum punctatum | 109.7205 | 19.11708 |  |  |
| Combretum punctatum | 109.8933 | 18.95158 |  |  |
| Combretum punctatum | 109.7685 | 19.05831 |  |  |
| Combretum punctatum | 109.8463 | 19.07446 |  |  |
| Combretum punctatum | 109.8678 | 18.99895 |  |  |
| Combretum punctatum | 109.799 | 19.10535 |  |  |
| Combretum punctatum | 109.4713 | 19.21752 |  |  |
| Combretum punctatum | 109.3952 | 19.23239 |  |  |
| Combretum punctatum | 109.3514 | 19.21033 |  |  |
| Combretum punctatum | 109.4668 | 19.21984 |  |  |
| Combretum punctatum | 109.3933 | 19.23374 |  |  |
| Combretum punctatum | 109.3535 | 19.21319 |  |  |
| Cordia dichotoma | 108.7989 | 18.69619 | -48238.4 | 254162.2 |
| Cordia dichotoma | 109.1741 | 18.37558 | -9781.56 | 217466.3 |
| Cordia dichotoma | 109.6807 | 18.37829 | 43729.57 | 216234.2 |
| Cordia dichotoma | 109.7163 | 19.87976 | 51934.37 | 382144 |
| Cordia dichotoma | 109.777 | 19.17887 | 56233.56 | 304494 |
| Cordia dichotoma | 109.399 | 18.9812 |  |  |
| Cordia dichotoma | 109.0905 | 18.99858 |  |  |
| Cordia dichotoma | 109.5469 | 19.09198 |  |  |
| Cordia dichotoma | 109.5428 | 19.0955 |  |  |
| Cordia dichotoma | 109.7689 | 19.14619 |  |  |
| Cordia dichotoma | 109.6339 | 19.12832 |  |  |
| Cordia dichotoma | 109.5297 | 19.0159 |  |  |
| Cordia dichotoma | 109.098 | 19.13914 |  |  |
| Cordia dichotoma | 109.566 | 19.23784 |  |  |
| Cordia dichotoma | 109.5256 | 18.89515 |  |  |
| Cordia dichotoma | 109.8903 | 18.91378 |  |  |
| Cordia dichotoma | 109.1822 | 19.16237 |  |  |
| Cordia dichotoma | 109.993 | 18.67735 |  |  |
| Cordia dichotoma | 109.8761 | 19.1446 |  |  |
| Cordia dichotoma | 109.1212 | 18.7811 |  |  |
| Cordia dichotoma | 109.8906 | 18.95886 |  |  |
| Cordia dichotoma | 109.0558 | 19.19585 |  |  |
| Cordia dichotoma | 109.5241 | 19.21788 |  |  |
| Cordia dichotoma | 109.067 | 18.7339 |  |  |
| Cordia dichotoma | 108.9768 | 19.08223 |  |  |
| Cordyline fruticosa | 109.2343 | 18.80064 | -2022.54 | 264280.3 |
| Cordyline fruticosa | 109.777 | 19.17887 | 56233.56 | 304494 |
| Cordyline fruticosa | 110.0914 | 18.76565 | 88154.38 | 257960.3 |
| Cordyline fruticosa | 109.2486 | 18.72666 |  |  |
| Cordyline fruticosa | 109.8864 | 19.23205 |  |  |
| Cordyline fruticosa | 109.7158 | 19.12934 |  |  |
| Cordyline fruticosa | 109.1943 | 18.74217 |  |  |
| Cordyline fruticosa | 109.444 | 19.09844 |  |  |
| Cordyline fruticosa | 109.5874 | 19.15455 |  |  |
| Cordyline fruticosa | 109.608 | 19.12586 |  |  |
| Cordyline fruticosa | 109.5972 | 19.12846 |  |  |
| Cordyline fruticosa | 109.1325 | 19.07501 |  |  |
| Cordyline fruticosa | 109.8132 | 18.94999 |  |  |
| Cordyline fruticosa | 110.5074 | 19.26041 |  |  |
| Cordyline fruticosa | 110.1832 | 19.24159 |  |  |
| Cordyline fruticosa | 109.6678 | 19.27756 |  |  |
| Cordyline fruticosa | 110.2119 | 19.61225 |  |  |
| Cordyline fruticosa | 109.6092 | 19.69743 |  |  |
| Cordyline fruticosa | 110.1772 | 19.37606 |  |  |
| Cordyline fruticosa | 110.3153 | 19.72918 |  |  |
| Cordyline fruticosa | 109.6872 | 19.55757 |  |  |
| Cordyline fruticosa | 109.7839 | 19.39883 |  |  |
| Cordyline fruticosa | 110.3879 | 19.40232 |  |  |
| Craibiodendron scleranthum | 109.869 | 18.69141 | 64504.44 | 250339.5 |
| Craibiodendron scleranthum | 109.869 | 18.69141 | 64504.55 | 250339.6 |
| Craibiodendron scleranthum | 109.869 | 18.69141 | 64504.66 | 250339.7 |
| Craibiodendron scleranthum | 109.869 | 18.69141 | 64504.87 | 250339.8 |
| Craibiodendron scleranthum | 109.869 | 18.69141 | 64504.98 | 250340 |
| Craibiodendron scleranthum | 109.4786 | 19.17996 |  |  |
| Craibiodendron scleranthum | 109.9859 | 19.06964 |  |  |
| Craibiodendron scleranthum | 109.9504 | 19.09893 |  |  |
| Craibiodendron scleranthum | 109.991 | 19.20644 |  |  |
| Craibiodendron scleranthum | 109.8001 | 19.07881 |  |  |
| Craibiodendron scleranthum | 109.1042 | 18.88957 |  |  |
| Craibiodendron scleranthum | 109.3163 | 18.89881 |  |  |
| Craibiodendron scleranthum | 109.7934 | 18.69853 |  |  |
| Craibiodendron scleranthum | 109.4064 | 18.83785 |  |  |
| Craibiodendron scleranthum | 109.5035 | 18.87355 |  |  |
| Craibiodendron scleranthum | 109.9519 | 19.75586 |  |  |
| Craibiodendron scleranthum | 110.1371 | 19.75647 |  |  |
| Craibiodendron scleranthum | 110.077 | 19.81478 |  |  |
| Craibiodendron scleranthum | 110.3019 | 19.36829 |  |  |
| Craibiodendron scleranthum | 109.886 | 19.48506 |  |  |
| Craibiodendron scleranthum | 110.4601 | 19.26589 |  |  |
| Craibiodendron scleranthum | 110.3642 | 19.38368 |  |  |
| Craibiodendron scleranthum | 109.709 | 19.42547 |  |  |
| Craibiodendron scleranthum | 109.994 | 19.77699 |  |  |
| Craibiodendron scleranthum | 109.9979 | 19.27565 |  |  |
| Cratoxylum cochinchinense | 108.9515 | 18.90932 | -31419.3 | 277210.7 |
| Cratoxylum cochinchinense | 109.0367 | 19.17511 | -21544.3 | 306313.7 |
| Cratoxylum cochinchinense | 109.1191 | 19.05985 | -13271 | 293304.6 |
| Cratoxylum cochinchinense | 109.5146 | 18.9427 | 27935.88 | 279133.3 |
| Cratoxylum cochinchinense | 110.1756 | 18.76435 | 97019.43 | 257602 |
| Cratoxylum cochinchinense | 109.1581 | 19.20079 |  |  |
| Cratoxylum cochinchinense | 109.2348 | 19.00823 |  |  |
| Cratoxylum cochinchinense | 108.9617 | 18.93303 |  |  |
| Cratoxylum cochinchinense | 109.3902 | 18.69857 |  |  |
| Cratoxylum cochinchinense | 109.3301 | 18.96913 |  |  |
| Cratoxylum cochinchinense | 109.4572 | 18.82882 |  |  |
| Cratoxylum cochinchinense | 109.6631 | 19.16525 |  |  |
| Cratoxylum cochinchinense | 109.1247 | 19.02879 |  |  |
| Cratoxylum cochinchinense | 109.0243 | 18.82212 |  |  |
| Cratoxylum cochinchinense | 109.8884 | 19.1463 |  |  |
| Cratoxylum cochinchinense | 110.1223 | 19.25338 |  |  |
| Cratoxylum cochinchinense | 109.5769 | 19.83779 |  |  |
| Cratoxylum cochinchinense | 110.0711 | 19.46304 |  |  |
| Cratoxylum cochinchinense | 110.4569 | 19.36439 |  |  |
| Cratoxylum cochinchinense | 110.304 | 19.64812 |  |  |
| Cratoxylum cochinchinense | 110.481 | 19.60792 |  |  |
| Cratoxylum cochinchinense | 110.1438 | 19.43213 |  |  |
| Cratoxylum cochinchinense | 110.4928 | 19.47051 |  |  |
| Cratoxylum cochinchinense | 110.1174 | 19.7562 |  |  |
| Cratoxylum cochinchinense | 110.2203 | 19.2767 |  |  |
| Crescentia cujete | 109.416 | 18.58215 | 16423.77 | 239559.1 |
| Crescentia cujete | 110.3486 | 20.03905 | 118456.9 | 398109.7 |
| Crescentia cujete | 109.4289 | 18.70615 |  |  |
| Crescentia cujete | 109.3048 | 18.6721 |  |  |
| Crescentia cujete | 109.6424 | 18.74451 |  |  |
| Crescentia cujete | 109.2183 | 18.97869 |  |  |
| Crescentia cujete | 109.906 | 18.73197 |  |  |
| Crescentia cujete | 109.6515 | 18.91864 |  |  |
| Crescentia cujete | 109.6384 | 18.80348 |  |  |
| Crescentia cujete | 109.736 | 18.85606 |  |  |
| Crescentia cujete | 109.2096 | 18.93521 |  |  |
| Crescentia cujete | 109.367 | 18.77071 |  |  |
| Crescentia cujete | 109.8017 | 19.44518 |  |  |
| Crescentia cujete | 110.2891 | 19.60075 |  |  |
| Crescentia cujete | 109.9013 | 19.19598 |  |  |
| Crescentia cujete | 109.6226 | 19.39568 |  |  |
| Crescentia cujete | 110.2998 | 19.59612 |  |  |
| Crescentia cujete | 109.8506 | 19.33634 |  |  |
| Crescentia cujete | 110.4122 | 19.48014 |  |  |
| Crescentia cujete | 110.1635 | 19.17177 |  |  |
| Crescentia cujete | 110.1346 | 19.44882 |  |  |
| Crescentia cujete | 109.9193 | 19.1792 |  |  |
| Crotalaria pallida | 108.7941 | 19.11474 | -47239.5 | 300451.1 |
| Crotalaria pallida | 109.1191 | 19.05985 | -13271 | 293304.6 |
| Crotalaria pallida | 109.6401 | 19.49205 | 42797.58 | 339502 |
| Crotalaria pallida | 109.7097 | 19.53075 | 50211.1 | 343582.5 |
| Crotalaria pallida | 109.7575 | 19.47974 | 55069.1 | 337810.7 |
| Crotalaria pallida | 109.1333 | 18.76108 |  |  |
| Crotalaria pallida | 109.8912 | 18.89364 |  |  |
| Crotalaria pallida | 109.9449 | 18.80646 |  |  |
| Crotalaria pallida | 109.0109 | 18.73837 |  |  |
| Crotalaria pallida | 109.6632 | 18.97058 |  |  |
| Crotalaria pallida | 109.8013 | 18.70166 |  |  |
| Crotalaria pallida | 109.7077 | 19.11857 |  |  |
| Crotalaria pallida | 109.1884 | 19.00567 |  |  |
| Crotalaria pallida | 110.0305 | 19.16854 |  |  |
| Crotalaria pallida | 109.606 | 18.98524 |  |  |
| Crotalaria pallida | 109.6511 | 19.55432 |  |  |
| Crotalaria pallida | 109.6353 | 19.38406 |  |  |
| Crotalaria pallida | 109.6563 | 19.37129 |  |  |
| Crotalaria pallida | 109.9727 | 19.34823 |  |  |
| Crotalaria pallida | 109.8899 | 19.51042 |  |  |
| Crotalaria pallida | 109.9143 | 19.42415 |  |  |
| Crotalaria pallida | 109.9708 | 19.40083 |  |  |
| Crotalaria pallida | 110.1315 | 19.26293 |  |  |
| Crotalaria pallida | 109.9229 | 19.55796 |  |  |
| Crotalaria pallida | 109.892 | 19.40048 |  |  |
| Croton cascarilloides | 109.0609 | 18.7429 | -20480.1 | 258450.9 |
| Croton cascarilloides | 109.1191 | 19.05985 | -13271 | 293304.6 |
| Croton cascarilloides | 109.416 | 18.58215 | 16423.77 | 239559.1 |
| Croton cascarilloides | 110.2256 | 19.86031 | 105147.4 | 378650.1 |
| Croton cascarilloides | 110.3153 | 19.16929 | 112768.7 | 302034.3 |
| Croton cascarilloides | 108.7378 | 19.04482 |  |  |
| Croton cascarilloides | 108.7459 | 19.06621 |  |  |
| Croton cascarilloides | 108.7829 | 18.98865 |  |  |
| Croton cascarilloides | 109.0811 | 18.73592 |  |  |
| Croton cascarilloides | 109.2807 | 18.79624 |  |  |
| Croton cascarilloides | 109.0796 | 18.56118 |  |  |
| Croton cascarilloides | 109.7536 | 19.10682 |  |  |
| Croton cascarilloides | 109.7439 | 19.07307 |  |  |
| Croton cascarilloides | 109.7143 | 19.0032 |  |  |
| Croton cascarilloides | 109.9066 | 18.94968 |  |  |
| Croton cascarilloides | 109.4708 | 19.2202 |  |  |
| Croton cascarilloides | 109.3958 | 19.23135 |  |  |
| Croton cascarilloides | 109.3542 | 19.2187 |  |  |
| Croton cascarilloides | 109.4667 | 19.2185 |  |  |
| Croton cascarilloides | 109.3954 | 19.22964 |  |  |
| Croton cascarilloides | 109.3503 | 19.21106 |  |  |
| Croton cascarilloides | 109.1474 | 19.36008 |  |  |
| Croton cascarilloides | 109.1413 | 19.32269 |  |  |
| Croton cascarilloides | 109.0144 | 19.29969 |  |  |
| Croton cascarilloides | 109.0167 | 19.30511 |  |  |
| Croton crassifolius | 109.0575 | 18.39577 | -22026.4 | 220074.8 |
| Croton crassifolius | 109.0947 | 18.75221 | -16882.4 | 259370.5 |
| Croton crassifolius | 109.2085 | 18.98615 | -4124.48 | 284872.7 |
| Croton crassifolius | 109.939 | 19.84766 | 75135.47 | 377987.1 |
| Croton crassifolius | 110.8845 | 19.93617 | 174229.8 | 385550.5 |
| Croton crassifolius | 110.4108 | 19.30958 |  |  |
| Croton crassifolius | 110.4213 | 19.29368 |  |  |
| Croton crassifolius | 110.4267 | 19.3277 |  |  |
| Croton crassifolius | 110.3598 | 19.30977 |  |  |
| Croton crassifolius | 110.3781 | 19.30703 |  |  |
| Croton crassifolius | 110.5951 | 19.21872 |  |  |
| Croton crassifolius | 110.5538 | 19.24802 |  |  |
| Croton crassifolius | 110.5462 | 19.2227 |  |  |
| Croton crassifolius | 110.583 | 19.25851 |  |  |
| Croton crassifolius | 109.6517 | 18.25233 |  |  |
| Croton crassifolius | 109.5962 | 18.26076 |  |  |
| Croton crassifolius | 109.5949 | 18.22554 |  |  |
| Croton crassifolius | 110.2162 | 20.01163 |  |  |
| Croton crassifolius | 109.672 | 19.95703 |  |  |
| Croton crassifolius | 109.7415 | 19.90886 |  |  |
| Croton crassifolius | 109.6015 | 19.87946 |  |  |
| Croton crassifolius | 110.4636 | 19.85492 |  |  |
| Croton crassifolius | 110.4115 | 19.84899 |  |  |
| Croton crassifolius | 108.7351 | 19.05082 |  |  |
| Croton crassifolius | 108.7448 | 19.06628 |  |  |
| Croton kongensis | 109.0799 | 19.00987 | -17563.9 | 287906.3 |
| Croton kongensis | 109.1215 | 19.14164 | -12747.8 | 302339.7 |
| Croton kongensis | 109.2747 | 19.85903 | 5691.175 | 381153.9 |
| Croton kongensis | 109.6807 | 18.37829 | 43729.57 | 216234.2 |
| Croton kongensis | 109.9845 | 18.39526 | 75860.07 | 217274.4 |
| Croton kongensis | 108.7408 | 19.04332 |  |  |
| Croton kongensis | 108.7435 | 19.06585 |  |  |
| Croton kongensis | 108.7912 | 18.98429 |  |  |
| Croton kongensis | 110.1015 | 18.54128 |  |  |
| Croton kongensis | 110.0731 | 18.51669 |  |  |
| Croton kongensis | 110.0949 | 18.55668 |  |  |
| Croton kongensis | 109.6944 | 19.42778 |  |  |
| Croton kongensis | 109.614 | 19.43815 |  |  |
| Croton kongensis | 109.6416 | 19.52163 |  |  |
| Croton kongensis | 109.6618 | 19.57028 |  |  |
| Croton kongensis | 109.0225 | 19.31118 |  |  |
| Croton kongensis | 109.0186 | 19.29909 |  |  |
| Croton kongensis | 109.0123 | 19.29682 |  |  |
| Croton kongensis | 109.0051 | 19.27939 |  |  |
| Croton kongensis | 109.1459 | 19.36578 |  |  |
| Croton kongensis | 109.1474 | 19.33069 |  |  |
| Croton kongensis | 109.0893 | 19.34918 |  |  |
| Croton kongensis | 109.6514 | 18.25743 |  |  |
| Croton kongensis | 109.5982 | 18.26041 |  |  |
| Croton kongensis | 109.6002 | 18.22258 |  |  |
| Croton laevigatus | 109.0609 | 18.7429 | -20480.1 | 258450.9 |
| Croton laevigatus | 109.0799 | 19.00987 | -17563.9 | 287906.3 |
| Croton laevigatus | 109.1191 | 19.05985 | -13271 | 293304.6 |
| Croton laevigatus | 109.1215 | 19.14164 | -12747.8 | 302339.7 |
| Croton laevigatus | 109.4865 | 19.15807 | 25651.28 | 303028.8 |
| Croton laevigatus | 109.5336 | 18.35654 | 28131.06 | 214255.7 |
| Croton laevigatus | 109.6807 | 18.37829 | 43729.57 | 216234.2 |
| Croton laevigatus | 109.9845 | 18.39526 | 75860.07 | 217274.4 |
| Croton laevigatus | 110.1756 | 18.76435 | 97019.43 | 257602 |
| Croton laevigatus | 109.123 | 19.08692 |  |  |
| Croton laevigatus | 109.8667 | 19.09744 |  |  |
| Croton laevigatus | 109.8683 | 19.09567 |  |  |
| Croton laevigatus | 109.8669 | 19.09725 |  |  |
| Croton laevigatus | 109.5414 | 19.0306 |  |  |
| Croton laevigatus | 109.5493 | 19.03361 |  |  |
| Croton laevigatus | 109.551 | 19.03197 |  |  |
| Croton laevigatus | 109.549 | 19.0335 |  |  |
| Croton laevigatus | 109.122 | 19.08975 |  |  |
| Croton laevigatus | 109.121 | 19.08845 |  |  |
| Croton laevigatus | 109.1192 | 19.0886 |  |  |
| Croton laevigatus | 109.8653 | 18.79093 |  |  |
| Croton laevigatus | 109.8878 | 18.7973 |  |  |
| Croton laevigatus | 109.8655 | 18.80001 |  |  |
| Croton laevigatus | 109.8845 | 18.78492 |  |  |
| Croton laevigatus | 109.261 | 19.03821 |  |  |
| Croton laevigatus | 109.2669 | 19.0383 |  |  |
| Croton laevigatus | 109.2643 | 19.04534 |  |  |
| Croton laevigatus | 109.2688 | 19.04413 |  |  |
| Croton laevigatus | 109.2574 | 19.03398 |  |  |
| Croton tiglium | 109.0365 | 18.87698 | -22580.2 | 273355 |
| Croton tiglium | 109.1191 | 19.05985 | -13271 | 293304.6 |
| Croton tiglium | 109.1723 | 19.64304 | -5730.41 | 357602 |
| Croton tiglium | 109.2248 | 18.51402 | -3968.98 | 232615.4 |
| Croton tiglium | 109.4865 | 19.15807 | 25651.28 | 303028.8 |
| Croton tiglium | 109.68 | 18.68397 | 44567.71 | 250043.4 |
| Croton tiglium | 109.6997 | 18.47449 | 46019.61 | 226820.3 |
| Croton tiglium | 109.7575 | 19.47974 | 55069.1 | 337810.7 |
| Croton tiglium | 109.8188 | 19.14345 | 60528.46 | 300462.2 |
| Croton tiglium | 110.0305 | 18.90546 | 82120.94 | 273579.6 |
| Croton tiglium | 110.2356 | 18.69662 | 103162.5 | 249961.2 |
| Croton tiglium | 109.9064 | 18.66259 |  |  |
| Croton tiglium | 109.6529 | 18.80008 |  |  |
| Croton tiglium | 109.064 | 19.11465 |  |  |
| Croton tiglium | 109.3222 | 18.66909 |  |  |
| Croton tiglium | 109.2771 | 19.18566 |  |  |
| Croton tiglium | 109.9893 | 18.68531 |  |  |
| Croton tiglium | 109.5728 | 19.26076 |  |  |
| Croton tiglium | 109.1211 | 19.0102 |  |  |
| Croton tiglium | 108.9723 | 19.01292 |  |  |
| Croton tiglium | 109.4281 | 18.74915 |  |  |
| Croton tiglium | 109.789 | 19.24945 |  |  |
| Croton tiglium | 109.6846 | 19.64125 |  |  |
| Croton tiglium | 110.2484 | 19.26412 |  |  |
| Croton tiglium | 109.7449 | 19.44182 |  |  |
| Croton tiglium | 110.1784 | 19.23796 |  |  |
| Croton tiglium | 110.4958 | 19.4861 |  |  |
| Croton tiglium | 109.689 | 19.8396 |  |  |
| Croton tiglium | 109.8576 | 19.7112 |  |  |
| Croton tiglium | 110.2714 | 19.80293 |  |  |
| Croton tiglium | 110.3423 | 19.54749 |  |  |
| Cryptocarya chinensis | 108.7989 | 18.69619 | -48238.4 | 254162.2 |
| Cryptocarya chinensis | 108.982 | 18.6052 | -29265.1 | 243483.5 |
| Cryptocarya chinensis | 109.0333 | 18.86036 | -22982.8 | 271527.7 |
| Cryptocarya chinensis | 109.1191 | 19.05985 | -13271 | 293304.6 |
| Cryptocarya chinensis | 109.1215 | 19.14164 | -12747.8 | 302339.7 |
| Cryptocarya chinensis | 109.4401 | 18.69801 | 19324.7 | 252298.9 |
| Cryptocarya chinensis | 109.6131 | 18.44069 | 36776.91 | 223331 |
| Cryptocarya chinensis | 109.6835 | 18.87437 | 45503.17 | 271088.8 |
| Cryptocarya chinensis | 109.836 | 19.04117 | 62035.47 | 289106.2 |
| Cryptocarya chinensis | 109.8795 | 18.7193 | 65691.55 | 253395.3 |
| Cryptocarya chinensis | 109.7672 | 18.57178 |  |  |
| Cryptocarya chinensis | 109.8124 | 18.58008 |  |  |
| Cryptocarya chinensis | 109.8571 | 18.59544 |  |  |
| Cryptocarya chinensis | 109.4666 | 19.21736 |  |  |
| Cryptocarya chinensis | 109.3943 | 19.2295 |  |  |
| Cryptocarya chinensis | 109.3479 | 19.21366 |  |  |
| Cryptocarya chinensis | 110.1007 | 18.51988 |  |  |
| Cryptocarya chinensis | 110.0961 | 18.5329 |  |  |
| Cryptocarya chinensis | 108.7437 | 19.04125 |  |  |
| Cryptocarya chinensis | 108.7468 | 19.06547 |  |  |
| Cryptocarya chinensis | 108.7811 | 18.96557 |  |  |
| Cryptocarya chinensis | 109.0193 | 18.72535 |  |  |
| Cryptocarya chinensis | 109.2809 | 18.79435 |  |  |
| Cryptocarya chinensis | 109.138 | 18.53937 |  |  |
| Cryptocarya chinensis | 109.6589 | 18.2492 |  |  |
| Cryptocarya chinensis | 109.5953 | 18.25827 |  |  |
| Cryptocarya chinensis | 109.5982 | 18.2265 |  |  |
| Cryptocarya chinensis | 110.3292 | 19.64078 |  |  |
| Cryptocarya chinensis | 110.3322 | 19.60463 |  |  |
| Cryptocarya chinensis | 110.3027 | 19.56313 |  |  |
| Cryptocarya concinna | 109.0333 | 18.86036 | -22982.8 | 271527.7 |
| Cryptocarya concinna | 109.1544 | 19.01211 | -9728.08 | 287914.8 |
| Cryptocarya concinna | 110.2332 | 18.6893 | 102892.4 | 249157.1 |
| Cryptocarya concinna | 110.457 | 18.89014 | 126969.4 | 270829.8 |
| Cryptocarya concinna | 110.6819 | 19.92734 | 153022.5 | 385001.2 |
| Cryptocarya concinna | 110.8273 | 19.57536 |  |  |
| Cryptocarya concinna | 110.8398 | 19.64598 |  |  |
| Cryptocarya concinna | 110.9179 | 19.61522 |  |  |
| Cryptocarya concinna | 110.9179 | 19.70019 |  |  |
| Cryptocarya concinna | 110.7982 | 19.68184 |  |  |
| Cryptocarya concinna | 110.8912 | 19.56362 |  |  |
| Cryptocarya concinna | 110.9285 | 19.59379 |  |  |
| Cryptocarya concinna | 109.7802 | 18.56467 |  |  |
| Cryptocarya concinna | 109.8272 | 18.57638 |  |  |
| Cryptocarya concinna | 109.8529 | 18.59646 |  |  |
| Cryptocarya concinna | 110.675 | 19.64141 |  |  |
| Cryptocarya concinna | 110.6719 | 19.57245 |  |  |
| Cryptocarya concinna | 110.7257 | 19.48114 |  |  |
| Cryptocarya concinna | 110.7031 | 19.5037 |  |  |
| Cryptocarya concinna | 109.7722 | 18.5736 |  |  |
| Cryptocarya concinna | 109.8277 | 18.57607 |  |  |
| Cryptocarya concinna | 109.8537 | 18.59692 |  |  |
| Cryptocarya concinna | 109.7648 | 18.56668 |  |  |
| Cryptocarya concinna | 109.8284 | 18.5733 |  |  |
| Cryptocarya concinna | 109.8517 | 18.59606 |  |  |
| Cryptocarya densiflora | 109.5425 | 19.03583 | 31162.38 | 289348.8 |
| Cryptocarya densiflora | 109.6835 | 18.87437 | 45503.17 | 271088.8 |
| Cryptocarya densiflora | 109.837 | 18.73456 | 61260.82 | 255199 |
| Cryptocarya densiflora | 109.8844 | 18.79929 | 66438.38 | 262227.7 |
| Cryptocarya densiflora | 110.1756 | 18.76435 | 97019.43 | 257602 |
| Cryptocarya densiflora | 109.7777 | 18.57248 |  |  |
| Cryptocarya densiflora | 109.8159 | 18.58031 |  |  |
| Cryptocarya densiflora | 109.865 | 18.5991 |  |  |
| Cryptocarya densiflora | 109.7693 | 18.56034 |  |  |
| Cryptocarya densiflora | 109.8338 | 18.57564 |  |  |
| Cryptocarya densiflora | 109.8536 | 18.60007 |  |  |
| Cryptocarya densiflora | 109.7773 | 18.57298 |  |  |
| Cryptocarya densiflora | 109.8168 | 18.58056 |  |  |
| Cryptocarya densiflora | 109.8528 | 18.59727 |  |  |
| Cryptocarya densiflora | 109.7676 | 18.56376 |  |  |
| Cryptocarya densiflora | 109.8188 | 18.57957 |  |  |
| Cryptocarya densiflora | 109.8541 | 18.60416 |  |  |
| Cryptocarya densiflora | 109.7527 | 19.09737 |  |  |
| Cryptocarya densiflora | 109.874 | 19.04737 |  |  |
| Cryptocarya densiflora | 109.7728 | 19.11239 |  |  |
| Cryptocarya densiflora | 109.715 | 18.9985 |  |  |
| Cryptocarya densiflora | 109.8659 | 19.02715 |  |  |
| Cryptocarya densiflora | 109.7948 | 19.04849 |  |  |
| Cryptocarya densiflora | 109.7607 | 19.08154 |  |  |
| Cryptocarya densiflora | 109.8032 | 19.03869 |  |  |
| Cryptocarya metcalfiana | 109.0333 | 18.86036 | -22982.8 | 271527.7 |
| Cryptocarya metcalfiana | 109.1215 | 19.14164 | -12747.8 | 302339.7 |
| Cryptocarya metcalfiana | 109.1741 | 18.37558 | -9781.56 | 217466.3 |
| Cryptocarya metcalfiana | 109.5425 | 19.03583 | 31162.38 | 289348.8 |
| Cryptocarya metcalfiana | 109.6579 | 18.76335 | 42477.13 | 258885.1 |
| Cryptocarya metcalfiana | 110.0914 | 18.76565 | 88154.38 | 257960.3 |
| Cryptocarya metcalfiana | 109.1202 | 19.08849 |  |  |
| Cryptocarya metcalfiana | 109.8655 | 19.09604 |  |  |
| Cryptocarya metcalfiana | 109.8669 | 19.09724 |  |  |
| Cryptocarya metcalfiana | 109.8636 | 19.09349 |  |  |
| Cryptocarya metcalfiana | 109.5486 | 19.03688 |  |  |
| Cryptocarya metcalfiana | 109.5516 | 19.03554 |  |  |
| Cryptocarya metcalfiana | 109.5434 | 19.03097 |  |  |
| Cryptocarya metcalfiana | 109.124 | 19.0863 |  |  |
| Cryptocarya metcalfiana | 109.121 | 19.08597 |  |  |
| Cryptocarya metcalfiana | 109.8685 | 18.79691 |  |  |
| Cryptocarya metcalfiana | 109.8787 | 18.78651 |  |  |
| Cryptocarya metcalfiana | 109.8716 | 18.78533 |  |  |
| Cryptocarya metcalfiana | 109.8806 | 18.80091 |  |  |
| Cryptocarya metcalfiana | 109.2719 | 19.0372 |  |  |
| Cryptocarya metcalfiana | 109.2663 | 19.03835 |  |  |
| Cryptocarya metcalfiana | 109.2575 | 19.04467 |  |  |
| Cryptocarya metcalfiana | 109.2568 | 19.04615 |  |  |
| Cryptocarya metcalfiana | 109.2626 | 19.03812 |  |  |
| Cryptocarya metcalfiana | 109.2607 | 19.04347 |  |  |
| Cryptocarya metcalfiana | 109.2613 | 19.04613 |  |  |
| Cryptolepis sinensis | 108.8228 | 18.4993 | -46431 | 232309.2 |
| Cryptolepis sinensis | 109.3011 | 18.85155 | 5180.119 | 269701.6 |
| Cryptolepis sinensis | 109.5377 | 19.09747 | 30846.03 | 296177.5 |
| Cryptolepis sinensis | 109.7632 | 18.66259 | 53269.54 | 247444.4 |
| Cryptolepis sinensis | 109.8639 | 19.57237 | 66490.61 | 347758.1 |
| Cryptolepis sinensis | 110.1492 | 19.21509 | 95439.41 | 307509.8 |
| Cryptolepis sinensis | 110.4194 | 18.9871 | 123247.6 | 281640.2 |
| Cryptolepis sinensis | 110.6146 | 19.99826 | 146153.1 | 392988.4 |
| Cryptolepis sinensis | 109.7841 | 18.56999 |  |  |
| Cryptolepis sinensis | 109.8254 | 18.57839 |  |  |
| Cryptolepis sinensis | 109.8674 | 18.60641 |  |  |
| Cryptolepis sinensis | 109.4699 | 19.2164 |  |  |
| Cryptolepis sinensis | 109.3899 | 19.23567 |  |  |
| Cryptolepis sinensis | 109.3513 | 19.21671 |  |  |
| Cryptolepis sinensis | 108.7351 | 19.04214 |  |  |
| Cryptolepis sinensis | 108.7475 | 19.06554 |  |  |
| Cryptolepis sinensis | 108.7932 | 18.98117 |  |  |
| Cryptolepis sinensis | 109.6578 | 18.25471 |  |  |
| Cryptolepis sinensis | 109.5998 | 18.25623 |  |  |
| Cryptolepis sinensis | 109.5907 | 18.22307 |  |  |
| Cryptolepis sinensis | 109.9554 | 19.73331 |  |  |
| Cryptolepis sinensis | 110.0248 | 19.78393 |  |  |
| Cryptolepis sinensis | 110.3292 | 19.6027 |  |  |
| Cryptolepis sinensis | 110.3236 | 19.58211 |  |  |
| Cryptolepis sinensis | 110.3101 | 19.6672 |  |  |
| Cryptolepis sinensis | 110.4676 | 19.85931 |  |  |
| Cryptolepis sinensis | 110.4129 | 19.85943 |  |  |
| Cryptolepis sinensis | 110.2156 | 20.01179 |  |  |
| Cycas changjiangensis | 108.9122 | 18.99917 | -35232.9 | 287275.8 |
| Cycas changjiangensis | 109.1191 | 19.05985 | -13271 | 293304.6 |
| Cycas changjiangensis | 109.0346 | 19.31096 |  |  |
| Cycas changjiangensis | 109.0278 | 19.30221 |  |  |
| Cycas changjiangensis | 109.0304 | 19.29878 |  |  |
| Cycas changjiangensis | 108.9854 | 19.30365 |  |  |
| Cycas changjiangensis | 109.1391 | 19.36086 |  |  |
| Cycas changjiangensis | 109.1388 | 19.36927 |  |  |
| Cycas changjiangensis | 109.1104 | 19.3594 |  |  |
| Cycas changjiangensis | 109.0679 | 19.30509 |  |  |
| Cycas changjiangensis | 109.0384 | 19.3037 |  |  |
| Cycas changjiangensis | 109.0069 | 19.30817 |  |  |
| Cycas changjiangensis | 109.0059 | 19.2833 |  |  |
| Cycas changjiangensis | 108.9856 | 19.29862 |  |  |
| Cycas changjiangensis | 109.1387 | 19.33427 |  |  |
| Cycas changjiangensis | 109.113 | 19.31602 |  |  |
| Cycas changjiangensis | 108.9875 | 19.31168 |  |  |
| Cycas changjiangensis | 109.1369 | 19.3128 |  |  |
| Cycas changjiangensis | 109.0751 | 19.34166 |  |  |
| Cycas changjiangensis | 109.1063 | 19.35259 |  |  |
| Cycas changjiangensis | 109.132 | 19.36623 |  |  |
| Cycas changjiangensis | 109.1011 | 19.35266 |  |  |
| Cyclocarya paliurus | 110.1756 | 18.76435 | 97019.43 | 257602 |
| Cyclocarya paliurus | 109.7384 | 18.9736 |  |  |
| Cyclocarya paliurus | 108.9778 | 18.67893 |  |  |
| Cyclocarya paliurus | 109.0611 | 19.19258 |  |  |
| Cyclocarya paliurus | 109.0128 | 19.24454 |  |  |
| Cyclocarya paliurus | 109.1019 | 18.71244 |  |  |
| Cyclocarya paliurus | 109.8246 | 19.25416 |  |  |
| Cyclocarya paliurus | 109.2483 | 18.89459 |  |  |
| Cyclocarya paliurus | 109.0555 | 19.04103 |  |  |
| Cyclocarya paliurus | 109.6429 | 18.9847 |  |  |
| Cyclocarya paliurus | 110.0217 | 19.20722 |  |  |
| Cyclocarya paliurus | 110.4324 | 19.71421 |  |  |
| Cyclocarya paliurus | 109.9044 | 19.36097 |  |  |
| Cyclocarya paliurus | 110.3536 | 19.63277 |  |  |
| Cyclocarya paliurus | 110.0167 | 19.59553 |  |  |
| Cyclocarya paliurus | 110.1871 | 19.54088 |  |  |
| Cyclocarya paliurus | 110.1601 | 19.42732 |  |  |
| Cyclocarya paliurus | 110.3154 | 19.56271 |  |  |
| Cyclocarya paliurus | 110.4196 | 19.4435 |  |  |
| Cyclocarya paliurus | 109.8749 | 19.46011 |  |  |
| Cyclocarya paliurus | 109.7336 | 19.49082 |  |  |
| Cynanchum corymbosum | 109.1191 | 19.05985 | -13271 | 293304.6 |
| Cynanchum corymbosum | 109.1191 | 19.05985 | -13270.7 | 293304.7 |
| Cynanchum corymbosum | 109.1191 | 19.05985 | -13270.6 | 293304.8 |
| Cynanchum corymbosum | 109.1191 | 19.05985 | -13270.5 | 293304.9 |
| Cynanchum corymbosum | 109.1191 | 19.05985 | -13270.4 | 293305.1 |
| Cynanchum corymbosum | 110.4387 | 18.79603 |  |  |
| Cynanchum corymbosum | 110.4358 | 18.80309 |  |  |
| Cynanchum corymbosum | 110.4242 | 18.79681 |  |  |
| Cynanchum corymbosum | 110.4308 | 18.79755 |  |  |
| Cynanchum corymbosum | 110.4239 | 18.80226 |  |  |
| Cynanchum corymbosum | 110.4292 | 18.80327 |  |  |
| Cynanchum corymbosum | 110.3826 | 18.77193 |  |  |
| Cynanchum corymbosum | 110.3749 | 18.76876 |  |  |
| Cynanchum corymbosum | 110.3816 | 18.76895 |  |  |
| Cynanchum corymbosum | 110.371 | 18.76945 |  |  |
| Cynanchum corymbosum | 110.3775 | 18.775 |  |  |
| Cynanchum corymbosum | 110.4044 | 18.77145 |  |  |
| Cynanchum corymbosum | 109.6859 | 19.55255 |  |  |
| Cynanchum corymbosum | 109.6215 | 19.47747 |  |  |
| Cynanchum corymbosum | 109.6265 | 19.50574 |  |  |
| Cynanchum corymbosum | 109.08 | 18.6639 |  |  |
| Cynanchum corymbosum | 109.278 | 18.79511 |  |  |
| Cynanchum corymbosum | 109.0373 | 18.56436 |  |  |
| Cynanchum corymbosum | 109.0567 | 18.58083 |  |  |
| Cynanchum corymbosum | 109.1226 | 18.57466 |  |  |
| Dacrycarpus imbricatus | 108.7989 | 18.69619 | -48238.4 | 254162.2 |
| Dacrycarpus imbricatus | 109.4865 | 19.15807 | 25651.28 | 303028.8 |
| Dacrycarpus imbricatus | 109.5425 | 19.03583 | 31162.38 | 289348.8 |
| Dacrycarpus imbricatus | 109.6579 | 18.76335 | 42477.13 | 258885.1 |
| Dacrycarpus imbricatus | 109.6975 | 18.72833 | 46544.54 | 254900 |
| Dacrycarpus imbricatus | 110.2536 | 19.61862 |  |  |
| Dacrycarpus imbricatus | 110.2339 | 19.62475 |  |  |
| Dacrycarpus imbricatus | 110.2178 | 19.67372 |  |  |
| Dacrycarpus imbricatus | 110.3077 | 19.55413 |  |  |
| Dacrycarpus imbricatus | 110.3291 | 19.57424 |  |  |
| Dacrycarpus imbricatus | 109.9098 | 18.9524 |  |  |
| Dacrycarpus imbricatus | 109.7552 | 18.94233 |  |  |
| Dacrycarpus imbricatus | 109.9036 | 18.94908 |  |  |
| Dacrycarpus imbricatus | 109.7941 | 19.0637 |  |  |
| Dacrycarpus imbricatus | 109.8724 | 19.10647 |  |  |
| Dacrycarpus imbricatus | 110.0722 | 18.53774 |  |  |
| Dacrycarpus imbricatus | 110.1059 | 18.53566 |  |  |
| Dacrycarpus imbricatus | 110.0961 | 18.55147 |  |  |
| Dacrycarpus imbricatus | 110.0873 | 18.52665 |  |  |
| Dacrycarpus imbricatus | 109.4688 | 19.21876 |  |  |
| Dacrycarpus imbricatus | 109.4707 | 19.21997 |  |  |
| Dacrycarpus imbricatus | 109.4699 | 19.21745 |  |  |
| Dacrycarpus imbricatus | 109.7763 | 18.55897 |  |  |
| Dacrycarpus imbricatus | 109.7844 | 18.56108 |  |  |
| Dacrycarpus imbricatus | 108.7466 | 19.06557 |  |  |
| Dacrydium pectinatum | 108.7989 | 18.69619 | -48238.4 | 254162.2 |
| Dacrydium pectinatum | 108.982 | 18.6052 | -29265.1 | 243483.5 |
| Dacrydium pectinatum | 109.4865 | 19.15807 | 25651.28 | 303028.8 |
| Dacrydium pectinatum | 109.5153 | 18.61925 | 27012 | 243364.4 |
| Dacrydium pectinatum | 109.6579 | 18.76335 | 42477.13 | 258885.1 |
| Dacrydium pectinatum | 109.6829 | 18.85594 |  |  |
| Dacrydium pectinatum | 109.6642 | 18.83054 |  |  |
| Dacrydium pectinatum | 109.6636 | 18.8515 |  |  |
| Dacrydium pectinatum | 109.6762 | 18.84352 |  |  |
| Dacrydium pectinatum | 109.6909 | 18.84753 |  |  |
| Dacrydium pectinatum | 109.6591 | 18.85279 |  |  |
| Dacrydium pectinatum | 109.879 | 18.78969 |  |  |
| Dacrydium pectinatum | 109.8679 | 18.79803 |  |  |
| Dacrydium pectinatum | 109.8879 | 18.78712 |  |  |
| Dacrydium pectinatum | 109.8782 | 18.79794 |  |  |
| Dacrydium pectinatum | 109.8846 | 18.78763 |  |  |
| Dacrydium pectinatum | 109.8793 | 18.79027 |  |  |
| Dacrydium pectinatum | 109.2645 | 19.03959 |  |  |
| Dacrydium pectinatum | 109.2674 | 19.04288 |  |  |
| Dacrydium pectinatum | 109.2727 | 19.04198 |  |  |
| Dacrydium pectinatum | 109.2691 | 19.03771 |  |  |
| Dacrydium pectinatum | 109.257 | 19.04335 |  |  |
| Dacrydium pectinatum | 109.2637 | 19.03407 |  |  |
| Dacrydium pectinatum | 109.2584 | 19.04617 |  |  |
| Dacrydium pectinatum | 109.2738 | 19.043 |  |  |
| Dalbergia assamica | 109.0333 | 18.86036 | -22982.8 | 271527.7 |
| Dalbergia assamica | 109.1191 | 19.05985 | -13271 | 293304.6 |
| Dalbergia assamica | 109.4044 | 18.88006 | 16149.68 | 272536.9 |
| Dalbergia assamica | 109.4865 | 19.15807 | 25651.28 | 303028.8 |
| Dalbergia assamica | 109.7575 | 19.47974 | 55069.1 | 337810.7 |
| Dalbergia assamica | 109.4691 | 19.21752 |  |  |
| Dalbergia assamica | 109.4695 | 19.21821 |  |  |
| Dalbergia assamica | 109.4666 | 19.21996 |  |  |
| Dalbergia assamica | 109.4697 | 19.2188 |  |  |
| Dalbergia assamica | 109.4698 | 19.21646 |  |  |
| Dalbergia assamica | 109.4687 | 19.21881 |  |  |
| Dalbergia assamica | 109.9814 | 19.74887 |  |  |
| Dalbergia assamica | 109.9667 | 19.77701 |  |  |
| Dalbergia assamica | 110.0043 | 19.76017 |  |  |
| Dalbergia assamica | 110.0488 | 19.79161 |  |  |
| Dalbergia assamica | 109.9892 | 19.72794 |  |  |
| Dalbergia assamica | 110.0939 | 18.53107 |  |  |
| Dalbergia assamica | 110.0549 | 18.5536 |  |  |
| Dalbergia assamica | 110.1004 | 18.55952 |  |  |
| Dalbergia assamica | 110.0927 | 18.52402 |  |  |
| Dalbergia assamica | 110.0701 | 18.51641 |  |  |
| Dalbergia assamica | 109.0724 | 18.75373 |  |  |
| Dalbergia assamica | 109.0753 | 18.71733 |  |  |
| Dalbergia assamica | 109.0172 | 18.75122 |  |  |
| Dalbergia assamica | 109.0455 | 18.71885 |  |  |
| Dalbergia benthamii | 109.0333 | 18.86036 | -22982.8 | 271527.7 |
| Dalbergia benthamii | 109.2343 | 18.80064 | -2022.54 | 264280.3 |
| Dalbergia benthamii | 109.6401 | 19.49205 | 42797.58 | 339502 |
| Dalbergia benthamii | 109.6807 | 18.37829 | 43729.57 | 216234.2 |
| Dalbergia benthamii | 110.1756 | 18.76435 | 97019.43 | 257602 |
| Dalbergia benthamii | 110.2641 | 19.61412 |  |  |
| Dalbergia benthamii | 110.2775 | 19.54015 |  |  |
| Dalbergia benthamii | 109.3927 | 19.23532 |  |  |
| Dalbergia benthamii | 109.3909 | 19.23131 |  |  |
| Dalbergia benthamii | 109.3914 | 19.23192 |  |  |
| Dalbergia benthamii | 109.689 | 19.45562 |  |  |
| Dalbergia benthamii | 109.6858 | 19.49533 |  |  |
| Dalbergia benthamii | 109.7043 | 19.48569 |  |  |
| Dalbergia benthamii | 109.6624 | 19.5145 |  |  |
| Dalbergia benthamii | 110.078 | 19.81113 |  |  |
| Dalbergia benthamii | 109.9463 | 19.7329 |  |  |
| Dalbergia benthamii | 109.9855 | 19.74939 |  |  |
| Dalbergia benthamii | 110.375 | 18.7695 |  |  |
| Dalbergia benthamii | 110.375 | 18.77661 |  |  |
| Dalbergia benthamii | 109.8486 | 18.60904 |  |  |
| Dalbergia benthamii | 109.8535 | 18.60477 |  |  |
| Dalbergia benthamii | 109.6008 | 18.25848 |  |  |
| Dalbergia benthamii | 109.6004 | 18.25722 |  |  |
| Dalbergia benthamii | 110.1026 | 18.51833 |  |  |
| Dalbergia benthamii | 110.0835 | 18.52255 |  |  |
| Dalbergia hainanensis | 109.0333 | 18.86036 | -22982.8 | 271527.7 |
| Dalbergia hainanensis | 109.4865 | 19.15807 | 25651.28 | 303028.8 |
| Dalbergia hainanensis | 109.6579 | 18.76335 | 42477.13 | 258885.1 |
| Dalbergia hainanensis | 109.6807 | 18.37829 | 43729.57 | 216234.2 |
| Dalbergia hainanensis | 109.6964 | 19.03697 | 47343.87 | 289030.5 |
| Dalbergia hainanensis | 109.837 | 18.73456 | 61260.82 | 255199 |
| Dalbergia hainanensis | 110.1756 | 18.76435 | 97019.43 | 257602 |
| Dalbergia hainanensis | 109.8713 | 18.79157 |  |  |
| Dalbergia hainanensis | 109.8656 | 18.80215 |  |  |
| Dalbergia hainanensis | 109.8674 | 18.79862 |  |  |
| Dalbergia hainanensis | 109.8677 | 18.79164 |  |  |
| Dalbergia hainanensis | 109.8744 | 18.78704 |  |  |
| Dalbergia hainanensis | 109.264 | 19.04025 |  |  |
| Dalbergia hainanensis | 109.2671 | 19.04295 |  |  |
| Dalbergia hainanensis | 109.2591 | 19.03755 |  |  |
| Dalbergia hainanensis | 109.2689 | 19.03827 |  |  |
| Dalbergia hainanensis | 109.2676 | 19.03349 |  |  |
| Dalbergia hainanensis | 109.2711 | 19.03307 |  |  |
| Dalbergia hainanensis | 109.1182 | 19.08564 |  |  |
| Dalbergia hainanensis | 109.122 | 19.08883 |  |  |
| Dalbergia hainanensis | 109.1171 | 19.09066 |  |  |
| Dalbergia hainanensis | 109.1177 | 19.08639 |  |  |
| Dalbergia hainanensis | 109.8695 | 19.09758 |  |  |
| Dalbergia hainanensis | 109.8629 | 19.09312 |  |  |
| Dalbergia hainanensis | 109.5517 | 19.03787 |  |  |
| Dalbergia hainanensis | 109.5413 | 19.02887 |  |  |
| Dalbergia hainanensis | 109.8658 | 19.09401 |  |  |
| Dalbergia hancei | 109.0799 | 19.00987 | -17563.9 | 287906.3 |
| Dalbergia hancei | 109.1544 | 19.01211 | -9728.08 | 287914.8 |
| Dalbergia hancei | 109.2343 | 18.80064 | -2022.54 | 264280.3 |
| Dalbergia hancei | 109.2757 | 19.44978 | 4460.62 | 335915.6 |
| Dalbergia hancei | 110.0914 | 18.76565 | 88154.38 | 257960.3 |
| Dalbergia hancei | 110.2079 | 19.59122 |  |  |
| Dalbergia hancei | 110.3246 | 19.55479 |  |  |
| Dalbergia hancei | 110.2437 | 19.59585 |  |  |
| Dalbergia hancei | 110.2422 | 19.59841 |  |  |
| Dalbergia hancei | 110.2267 | 19.59105 |  |  |
| Dalbergia hancei | 110.2109 | 19.57173 |  |  |
| Dalbergia hancei | 110.3047 | 19.59106 |  |  |
| Dalbergia hancei | 110.2247 | 19.62491 |  |  |
| Dalbergia hancei | 110.2249 | 19.55209 |  |  |
| Dalbergia hancei | 110.095 | 18.5359 |  |  |
| Dalbergia hancei | 110.0638 | 18.5524 |  |  |
| Dalbergia hancei | 110.0538 | 18.53177 |  |  |
| Dalbergia hancei | 110.0901 | 18.55004 |  |  |
| Dalbergia hancei | 110.0926 | 18.55235 |  |  |
| Dalbergia hancei | 110.0726 | 18.53384 |  |  |
| Dalbergia hancei | 110.0904 | 18.55167 |  |  |
| Dalbergia hancei | 110.0889 | 18.5512 |  |  |
| Dalbergia hancei | 110.1001 | 18.54764 |  |  |
| Dalbergia hancei | 110.0599 | 18.54364 |  |  |
| Dalbergia hancei | 110.0946 | 18.54416 |  |  |
| Dalbergia odorifera | 109.1741 | 18.37558 | -9781.56 | 217466.3 |
| Dalbergia odorifera | 109.4044 | 18.88006 | 16149.68 | 272536.9 |
| Dalbergia odorifera | 109.4401 | 18.69801 | 19324.7 | 252298.9 |
| Dalbergia odorifera | 110.2125 | 18.73421 | 100828.3 | 254175.6 |
| Dalbergia odorifera | 110.4509 | 19.26444 | 127246.5 | 312233.1 |
| Dalbergia odorifera | 109.3535 | 19.21669 |  |  |
| Dalbergia odorifera | 109.3553 | 19.21234 |  |  |
| Dalbergia odorifera | 109.3526 | 19.21639 |  |  |
| Dalbergia odorifera | 109.3552 | 19.21889 |  |  |
| Dalbergia odorifera | 109.3454 | 19.21905 |  |  |
| Dalbergia odorifera | 109.3498 | 19.21353 |  |  |
| Dalbergia odorifera | 109.0629 | 18.59695 |  |  |
| Dalbergia odorifera | 109.0854 | 18.57524 |  |  |
| Dalbergia odorifera | 109.0459 | 18.55965 |  |  |
| Dalbergia odorifera | 109.0991 | 18.53613 |  |  |
| Dalbergia odorifera | 109.1434 | 18.51943 |  |  |
| Dalbergia odorifera | 108.7474 | 19.06384 |  |  |
| Dalbergia odorifera | 108.7467 | 19.06624 |  |  |
| Dalbergia odorifera | 108.7434 | 19.06358 |  |  |
| Dalbergia odorifera | 108.7445 | 19.06638 |  |  |
| Dalbergia odorifera | 109.6557 | 18.25358 |  |  |
| Dalbergia odorifera | 109.654 | 18.25753 |  |  |
| Dalbergia odorifera | 109.6549 | 18.2536 |  |  |
| Dalbergia odorifera | 109.6489 | 18.255 |  |  |
| Dalbergia odorifera | 109.6544 | 18.25453 |  |  |
| Damnacanthus hainanensis | 109.675 | 18.86962 | 44597.28 | 270587.7 |
| Damnacanthus hainanensis | 109.6835 | 18.87437 | 45503.17 | 271088.8 |
| Damnacanthus hainanensis | 109.8636 | 19.09462 |  |  |
| Damnacanthus hainanensis | 109.863 | 19.0939 |  |  |
| Damnacanthus hainanensis | 109.8646 | 19.09617 |  |  |
| Damnacanthus hainanensis | 109.8677 | 19.09725 |  |  |
| Damnacanthus hainanensis | 109.8635 | 19.09642 |  |  |
| Damnacanthus hainanensis | 109.8649 | 19.09751 |  |  |
| Damnacanthus hainanensis | 109.8665 | 19.09349 |  |  |
| Damnacanthus hainanensis | 109.8683 | 19.0948 |  |  |
| Damnacanthus hainanensis | 109.8654 | 19.09569 |  |  |
| Damnacanthus hainanensis | 109.5478 | 19.03754 |  |  |
| Damnacanthus hainanensis | 109.5465 | 19.03609 |  |  |
| Damnacanthus hainanensis | 109.5458 | 19.03333 |  |  |
| Damnacanthus hainanensis | 109.5512 | 19.03386 |  |  |
| Damnacanthus hainanensis | 109.5512 | 19.03329 |  |  |
| Damnacanthus hainanensis | 109.5418 | 19.03752 |  |  |
| Damnacanthus hainanensis | 109.548 | 19.03062 |  |  |
| Damnacanthus hainanensis | 109.5446 | 19.0305 |  |  |
| Damnacanthus hainanensis | 109.5461 | 19.03569 |  |  |
| Damnacanthus hainanensis | 109.5458 | 19.03752 |  |  |
| Damnacanthus hainanensis | 109.5516 | 19.03641 |  |  |
| Daphniphyllum calycinum | 109.8844 | 18.79929 | 66438.38 | 262227.7 |
| Daphniphyllum calycinum | 110.1492 | 19.21509 | 95439.41 | 307509.8 |
| Daphniphyllum calycinum | 110.1756 | 18.76435 | 97019.43 | 257602 |
| Daphniphyllum calycinum | 110.2549 | 18.71143 | 105232.9 | 251551.4 |
| Daphniphyllum calycinum | 110.6901 | 19.65936 | 153267.3 | 355357.4 |
| Daphniphyllum calycinum | 110.9044 | 19.53915 |  |  |
| Daphniphyllum calycinum | 110.7867 | 19.57512 |  |  |
| Daphniphyllum calycinum | 110.937 | 19.53917 |  |  |
| Daphniphyllum calycinum | 110.8566 | 19.59327 |  |  |
| Daphniphyllum calycinum | 110.8824 | 19.53404 |  |  |
| Daphniphyllum calycinum | 110.8792 | 19.63107 |  |  |
| Daphniphyllum calycinum | 110.0538 | 19.81051 |  |  |
| Daphniphyllum calycinum | 110.0721 | 19.77223 |  |  |
| Daphniphyllum calycinum | 109.9452 | 19.73618 |  |  |
| Daphniphyllum calycinum | 109.9774 | 19.72368 |  |  |
| Daphniphyllum calycinum | 109.9834 | 19.74523 |  |  |
| Daphniphyllum calycinum | 110.0454 | 19.73646 |  |  |
| Daphniphyllum calycinum | 110.4218 | 18.75374 |  |  |
| Daphniphyllum calycinum | 110.4044 | 18.75498 |  |  |
| Daphniphyllum calycinum | 110.4374 | 18.73181 |  |  |
| Daphniphyllum calycinum | 109.5959 | 18.22534 |  |  |
| Daphniphyllum calycinum | 109.5925 | 18.22329 |  |  |
| Daphniphyllum calycinum | 109.5909 | 18.22284 |  |  |
| Daphniphyllum calycinum | 110.3636 | 19.32393 |  |  |
| Daphniphyllum calycinum | 110.414 | 19.30722 |  |  |
| Daphniphyllum paxianum | 109.0333 | 18.86036 | -22982.8 | 271527.7 |
| Daphniphyllum paxianum | 109.2343 | 18.80064 | -2022.54 | 264280.3 |
| Daphniphyllum paxianum | 109.445 | 19.2126 | 21468.7 | 309180.4 |
| Daphniphyllum paxianum | 109.4718 | 19.16737 | 24145.48 | 304100.4 |
| Daphniphyllum paxianum | 109.4865 | 19.15807 | 25651.28 | 303028.8 |
| Daphniphyllum paxianum | 109.6975 | 18.72833 | 46544.54 | 254900 |
| Daphniphyllum paxianum | 109.837 | 18.73456 | 61260.82 | 255199 |
| Daphniphyllum paxianum | 110.2559 | 19.09526 | 106327.7 | 293994.1 |
| Daphniphyllum paxianum | 109.3529 | 19.218 |  |  |
| Daphniphyllum paxianum | 109.3466 | 19.21223 |  |  |
| Daphniphyllum paxianum | 109.3513 | 19.21884 |  |  |
| Daphniphyllum paxianum | 109.3512 | 19.21132 |  |  |
| Daphniphyllum paxianum | 108.8015 | 18.95999 |  |  |
| Daphniphyllum paxianum | 108.7967 | 18.98122 |  |  |
| Daphniphyllum paxianum | 108.7994 | 18.96666 |  |  |
| Daphniphyllum paxianum | 108.7969 | 18.97076 |  |  |
| Daphniphyllum paxianum | 110.4198 | 19.30537 |  |  |
| Daphniphyllum paxianum | 110.4214 | 19.28014 |  |  |
| Daphniphyllum paxianum | 110.3695 | 19.32038 |  |  |
| Daphniphyllum paxianum | 110.3803 | 19.28215 |  |  |
| Daphniphyllum paxianum | 109.7753 | 18.57474 |  |  |
| Daphniphyllum paxianum | 109.7806 | 18.57352 |  |  |
| Daphniphyllum paxianum | 109.7642 | 18.56112 |  |  |
| Daphniphyllum paxianum | 109.5936 | 18.22527 |  |  |
| Daphniphyllum paxianum | 109.5975 | 18.22435 |  |  |
| Daphniphyllum paxianum | 110.0743 | 18.55434 |  |  |
| Daphniphyllum paxianum | 110.1023 | 18.55235 |  |  |
| Daphniphyllum paxianum | 110.0708 | 18.52732 |  |  |
| Dasymaschalon rostratum | 108.6526 | 18.84592 | -63101 | 271225.5 |
| Dasymaschalon rostratum | 109.5425 | 19.03583 | 31162.38 | 289348.8 |
| Dasymaschalon rostratum | 109.6807 | 18.37829 | 43729.57 | 216234.2 |
| Dasymaschalon rostratum | 110.0221 | 18.80753 | 80965.02 | 262772.1 |
| Dasymaschalon rostratum | 110.2238 | 18.67552 | 101866.1 | 247657.4 |
| Dasymaschalon rostratum | 110.0897 | 18.55785 |  |  |
| Dasymaschalon rostratum | 110.0871 | 18.55665 |  |  |
| Dasymaschalon rostratum | 110.0803 | 18.53573 |  |  |
| Dasymaschalon rostratum | 110.0584 | 18.53817 |  |  |
| Dasymaschalon rostratum | 110.0796 | 18.55947 |  |  |
| Dasymaschalon rostratum | 109.6501 | 18.25583 |  |  |
| Dasymaschalon rostratum | 109.659 | 18.25741 |  |  |
| Dasymaschalon rostratum | 109.6588 | 18.25214 |  |  |
| Dasymaschalon rostratum | 109.6493 | 18.25291 |  |  |
| Dasymaschalon rostratum | 109.65 | 18.24931 |  |  |
| Dasymaschalon rostratum | 109.769 | 18.56993 |  |  |
| Dasymaschalon rostratum | 109.7718 | 18.5739 |  |  |
| Dasymaschalon rostratum | 109.7649 | 18.56455 |  |  |
| Dasymaschalon rostratum | 109.7798 | 18.57281 |  |  |
| Dasymaschalon rostratum | 108.7419 | 19.05036 |  |  |
| Dasymaschalon rostratum | 108.7362 | 19.04961 |  |  |
| Dasymaschalon rostratum | 108.7338 | 19.04838 |  |  |
| Dasymaschalon rostratum | 109.0854 | 18.74032 |  |  |
| Dasymaschalon rostratum | 109.0411 | 18.67399 |  |  |
| Dasymaschalon rostratum | 109.0319 | 19.28962 |  |  |
| Dasymaschalon trichophorum | 108.7189 | 19.16737 | -54945.2 | 306528 |
| Dasymaschalon trichophorum | 109.0212 | 19.35345 | -22551.4 | 326078.8 |
| Dasymaschalon trichophorum | 109.3149 | 18.41558 | 5219.958 | 221446.7 |
| Dasymaschalon trichophorum | 109.5425 | 19.03583 | 31162.38 | 289348.8 |
| Dasymaschalon trichophorum | 109.6807 | 18.37829 | 43729.57 | 216234.2 |
| Dasymaschalon trichophorum | 109.1147 | 19.33343 |  |  |
| Dasymaschalon trichophorum | 109.1343 | 19.3275 |  |  |
| Dasymaschalon trichophorum | 109.1351 | 19.32473 |  |  |
| Dasymaschalon trichophorum | 109.1513 | 19.35081 |  |  |
| Dasymaschalon trichophorum | 109.1329 | 19.30763 |  |  |
| Dasymaschalon trichophorum | 109.0898 | 19.34373 |  |  |
| Dasymaschalon trichophorum | 109.1593 | 19.32846 |  |  |
| Dasymaschalon trichophorum | 109.2803 | 18.79295 |  |  |
| Dasymaschalon trichophorum | 109.2904 | 18.78234 |  |  |
| Dasymaschalon trichophorum | 109.275 | 18.77842 |  |  |
| Dasymaschalon trichophorum | 109.2802 | 18.78341 |  |  |
| Dasymaschalon trichophorum | 109.8621 | 18.60339 |  |  |
| Dasymaschalon trichophorum | 109.8673 | 18.60391 |  |  |
| Dasymaschalon trichophorum | 109.8591 | 18.59557 |  |  |
| Dasymaschalon trichophorum | 109.5957 | 18.25909 |  |  |
| Dasymaschalon trichophorum | 109.6013 | 18.26209 |  |  |
| Dasymaschalon trichophorum | 109.5963 | 18.26272 |  |  |
| Dasymaschalon trichophorum | 109.3941 | 19.23305 |  |  |
| Dasymaschalon trichophorum | 109.3938 | 19.22964 |  |  |
| Dasymaschalon trichophorum | 109.3919 | 19.22967 |  |  |
| Debregeasia squamata | 109.0333 | 18.86036 | -22982.8 | 271527.7 |
| Debregeasia squamata | 109.1215 | 19.14164 | -12747.8 | 302339.7 |
| Debregeasia squamata | 109.836 | 19.04117 | 62035.47 | 289106.2 |
| Debregeasia squamata | 109.9578 | 18.76128 | 74063.58 | 257827 |
| Debregeasia squamata | 110.0221 | 18.80753 | 80965.02 | 262772.1 |
| Debregeasia squamata | 108.7446 | 19.06377 |  |  |
| Debregeasia squamata | 108.7467 | 19.06552 |  |  |
| Debregeasia squamata | 108.7437 | 19.0649 |  |  |
| Debregeasia squamata | 108.7462 | 19.06523 |  |  |
| Debregeasia squamata | 108.7475 | 19.06362 |  |  |
| Debregeasia squamata | 108.7471 | 19.06435 |  |  |
| Debregeasia squamata | 109.0062 | 18.68446 |  |  |
| Debregeasia squamata | 109.0104 | 18.66274 |  |  |
| Debregeasia squamata | 109.0422 | 18.66225 |  |  |
| Debregeasia squamata | 109.0536 | 18.75023 |  |  |
| Debregeasia squamata | 109.0591 | 18.7213 |  |  |
| Debregeasia squamata | 109.0354 | 18.6846 |  |  |
| Debregeasia squamata | 109.8312 | 18.99118 |  |  |
| Debregeasia squamata | 109.7927 | 19.11645 |  |  |
| Debregeasia squamata | 109.7964 | 19.0298 |  |  |
| Debregeasia squamata | 110.0961 | 18.51737 |  |  |
| Debregeasia squamata | 110.0537 | 18.52694 |  |  |
| Debregeasia squamata | 109.7847 | 18.56167 |  |  |
| Debregeasia squamata | 109.7758 | 18.56563 |  |  |
| Debregeasia squamata | 109.7718 | 18.5681 |  |  |
| Decaspermum gracilentum | 109.0333 | 18.86036 | -22982.8 | 271527.7 |
| Decaspermum gracilentum | 109.1191 | 19.05985 | -13271 | 293304.6 |
| Decaspermum gracilentum | 109.1215 | 19.14164 | -12747.8 | 302339.7 |
| Decaspermum gracilentum | 109.2248 | 18.51402 | -3968.98 | 232615.4 |
| Decaspermum gracilentum | 109.4044 | 18.88006 | 16149.68 | 272536.9 |
| Decaspermum gracilentum | 109.5425 | 19.03583 | 31162.38 | 289348.8 |
| Decaspermum gracilentum | 109.6401 | 19.49205 | 42797.58 | 339502 |
| Decaspermum gracilentum | 109.6579 | 18.76335 | 42477.13 | 258885.1 |
| Decaspermum gracilentum | 109.6835 | 18.87437 | 45503.17 | 271088.8 |
| Decaspermum gracilentum | 109.6997 | 18.47449 | 46019.61 | 226820.3 |
| Decaspermum gracilentum | 109.7575 | 19.47974 | 55069.1 | 337810.7 |
| Decaspermum gracilentum | 109.837 | 18.73456 | 61260.82 | 255199 |
| Decaspermum gracilentum | 110.2238 | 18.67552 | 101866.1 | 247657.4 |
| Decaspermum gracilentum | 110.4321 | 19.37563 | 125548.1 | 324570.5 |
| Decaspermum gracilentum | 109.1999 | 18.7831 |  |  |
| Decaspermum gracilentum | 109.4386 | 19.15646 |  |  |
| Decaspermum gracilentum | 109.3527 | 19.21144 |  |  |
| Decaspermum gracilentum | 109.8448 | 18.67314 |  |  |
| Decaspermum gracilentum | 109.6186 | 18.80371 |  |  |
| Decaspermum gracilentum | 109.8372 | 19.05782 |  |  |
| Decaspermum gracilentum | 109.4739 | 18.96885 |  |  |
| Decaspermum gracilentum | 109.6798 | 18.82247 |  |  |
| Decaspermum gracilentum | 109.4951 | 18.83664 |  |  |
| Decaspermum gracilentum | 109.8519 | 19.74209 |  |  |
| Decaspermum gracilentum | 110.5118 | 19.65406 |  |  |
| Decaspermum gracilentum | 110.3024 | 19.40153 |  |  |
| Decaspermum gracilentum | 109.7785 | 19.76479 |  |  |
| Decaspermum gracilentum | 109.9038 | 19.703 |  |  |
| Decaspermum gracilentum | 109.6609 | 19.4662 |  |  |
| Decaspermum gracilentum | 110.2577 | 19.77287 |  |  |
| Decaspermum gracilentum | 110.123 | 19.33433 |  |  |
| Decaspermum gracilentum | 110.3405 | 19.81951 |  |  |
| Decaspermum gracilentum | 110.2708 | 19.23994 |  |  |
| Decaspermum gracilentum | 109.987 | 19.36811 |  |  |
| Deeringia amaranthoides | 109.0333 | 18.86036 | -22982.8 | 271527.7 |
| Deeringia amaranthoides | 109.1328 | 18.39072 | -14084.9 | 219272.7 |
| Deeringia amaranthoides | 109.1741 | 18.37558 | -9781.56 | 217466.3 |
| Deeringia amaranthoides | 109.3683 | 19.19189 | 13342.08 | 307122.7 |
| Deeringia amaranthoides | 109.5153 | 18.61925 | 27012 | 243364.4 |
| Deeringia amaranthoides | 109.6835 | 18.87437 | 45503.17 | 271088.8 |
| Deeringia amaranthoides | 109.777 | 19.17887 | 56233.56 | 304494 |
| Deeringia amaranthoides | 110.1924 | 18.76787 | 98795.13 | 257948.6 |
| Deeringia amaranthoides | 110.295 | 19.62074 |  |  |
| Deeringia amaranthoides | 110.3026 | 19.56351 |  |  |
| Deeringia amaranthoides | 110.216 | 19.59759 |  |  |
| Deeringia amaranthoides | 110.3051 | 19.67685 |  |  |
| Deeringia amaranthoides | 109.1464 | 19.33821 |  |  |
| Deeringia amaranthoides | 109.0907 | 19.30775 |  |  |
| Deeringia amaranthoides | 109.0688 | 19.32519 |  |  |
| Deeringia amaranthoides | 109.1612 | 19.33501 |  |  |
| Deeringia amaranthoides | 109.1006 | 19.31923 |  |  |
| Deeringia amaranthoides | 109.1632 | 19.36716 |  |  |
| Deeringia amaranthoides | 109.2956 | 18.79744 |  |  |
| Deeringia amaranthoides | 109.2995 | 18.80283 |  |  |
| Deeringia amaranthoides | 109.2888 | 18.79886 |  |  |
| Deeringia amaranthoides | 109.2981 | 18.77721 |  |  |
| Deeringia amaranthoides | 108.7426 | 19.04804 |  |  |
| Deeringia amaranthoides | 108.7329 | 19.0433 |  |  |
| Deeringia amaranthoides | 109.6526 | 18.25231 |  |  |
| Deeringia amaranthoides | 109.6513 | 18.25775 |  |  |
| Deeringia amaranthoides | 109.467 | 19.21806 |  |  |
| Deeringia amaranthoides | 109.4658 | 19.21961 |  |  |
| Dehaasia hainanensis | 109.0333 | 18.86036 | -22982.8 | 271527.7 |
| Dehaasia hainanensis | 109.0365 | 18.87698 | -22580.2 | 273355 |
| Dehaasia hainanensis | 109.1215 | 19.14164 | -12747.8 | 302339.7 |
| Dehaasia hainanensis | 109.3579 | 18.2994 | 9382.59 | 208464.8 |
| Dehaasia hainanensis | 109.4273 | 19.01928 | 18993.09 | 287860.9 |
| Dehaasia hainanensis | 109.4401 | 18.69801 | 19324.7 | 252298.9 |
| Dehaasia hainanensis | 109.2601 | 19.0354 |  |  |
| Dehaasia hainanensis | 109.2557 | 19.03689 |  |  |
| Dehaasia hainanensis | 109.2716 | 19.03958 |  |  |
| Dehaasia hainanensis | 109.2636 | 19.03792 |  |  |
| Dehaasia hainanensis | 109.2739 | 19.04439 |  |  |
| Dehaasia hainanensis | 109.2653 | 19.04305 |  |  |
| Dehaasia hainanensis | 109.1239 | 19.08581 |  |  |
| Dehaasia hainanensis | 109.121 | 19.08835 |  |  |
| Dehaasia hainanensis | 109.1194 | 19.0867 |  |  |
| Dehaasia hainanensis | 109.1181 | 19.08814 |  |  |
| Dehaasia hainanensis | 109.1184 | 19.08771 |  |  |
| Dehaasia hainanensis | 109.866 | 19.0963 |  |  |
| Dehaasia hainanensis | 109.869 | 19.09645 |  |  |
| Dehaasia hainanensis | 109.866 | 19.09689 |  |  |
| Dehaasia hainanensis | 109.8636 | 19.09397 |  |  |
| Dehaasia hainanensis | 109.5425 | 19.03607 |  |  |
| Dehaasia hainanensis | 109.548 | 19.02888 |  |  |
| Dehaasia hainanensis | 109.5439 | 19.03781 |  |  |
| Dehaasia hainanensis | 109.5476 | 19.02995 |  |  |
| Dehaasia hainanensis | 109.5491 | 19.03212 |  |  |
| Dendrocalamus latiflorus | 109.5153 | 18.61925 | 27012 | 243364.4 |
| Dendrocalamus latiflorus | 109.5153 | 18.61925 | 27012.11 | 243364.5 |
| Dendrocalamus latiflorus | 109.5153 | 18.61925 | 27012.22 | 243364.6 |
| Dendrocalamus latiflorus | 109.5153 | 18.61925 | 27012.32 | 243364.7 |
| Dendrocalamus latiflorus | 109.5153 | 18.61925 | 27012.43 | 243364.8 |
| Dendrocalamus latiflorus | 110.5398 | 19.22195 |  |  |
| Dendrocalamus latiflorus | 110.5752 | 19.26136 |  |  |
| Dendrocalamus latiflorus | 110.5869 | 19.22952 |  |  |
| Dendrocalamus latiflorus | 110.5429 | 19.25329 |  |  |
| Dendrocalamus latiflorus | 110.5997 | 19.2461 |  |  |
| Dendrocalamus latiflorus | 110.5752 | 19.23881 |  |  |
| Dendrocalamus latiflorus | 109.7707 | 19.03309 |  |  |
| Dendrocalamus latiflorus | 109.7547 | 19.0567 |  |  |
| Dendrocalamus latiflorus | 109.8779 | 18.98227 |  |  |
| Dendrocalamus latiflorus | 109.7383 | 18.98541 |  |  |
| Dendrocalamus latiflorus | 109.8987 | 19.02299 |  |  |
| Dendrocalamus latiflorus | 109.89 | 19.09737 |  |  |
| Dendrocalamus latiflorus | 109.8338 | 18.58049 |  |  |
| Dendrocalamus latiflorus | 109.8272 | 18.58153 |  |  |
| Dendrocalamus latiflorus | 109.8264 | 18.57432 |  |  |
| Dendrocalamus latiflorus | 109.8325 | 18.58099 |  |  |
| Dendrocalamus latiflorus | 109.3861 | 19.23157 |  |  |
| Dendrocalamus latiflorus | 109.3916 | 19.23037 |  |  |
| Dendrocalamus latiflorus | 109.3876 | 19.23037 |  |  |
| Dendrocalamus latiflorus | 109.3959 | 19.23459 |  |  |
| Dendrocnide sinuata | 110.0305 | 18.90546 | 82120.94 | 273579.6 |
| Dendrocnide sinuata | 110.0412 | 18.90005 |  |  |
| Dendrocnide sinuata | 110.0124 | 18.87276 |  |  |
| Dendrocnide sinuata | 110.0368 | 18.89847 |  |  |
| Dendrocnide sinuata | 110.0341 | 18.88841 |  |  |
| Dendrocnide sinuata | 110.0203 | 18.89639 |  |  |
| Dendrocnide sinuata | 110.0284 | 18.87804 |  |  |
| Dendrocnide sinuata | 110.0282 | 18.89542 |  |  |
| Dendrocnide sinuata | 110.0126 | 18.87865 |  |  |
| Dendrocnide sinuata | 110.0377 | 18.87852 |  |  |
| Dendrocnide sinuata | 110.0198 | 18.89931 |  |  |
| Dendrocnide sinuata | 110.0378 | 18.88038 |  |  |
| Dendrocnide sinuata | 110.0149 | 18.89048 |  |  |
| Dendrocnide sinuata | 110.0339 | 18.87503 |  |  |
| Dendrocnide sinuata | 110.0297 | 18.89845 |  |  |
| Dendrocnide sinuata | 110.028 | 18.8818 |  |  |
| Dendrocnide sinuata | 110.0337 | 18.89094 |  |  |
| Dendrocnide sinuata | 110.0356 | 18.87983 |  |  |
| Dendrocnide sinuata | 110.0333 | 18.87404 |  |  |
| Dendrocnide sinuata | 110.0215 | 18.88237 |  |  |
| Dendrocnide sinuata | 110.0245 | 18.87692 |  |  |
| Dendrolobium lanceolatum | 109.0367 | 19.17511 | -21544.3 | 306313.7 |
| Dendrolobium lanceolatum | 109.0609 | 18.7429 | -20480.1 | 258450.9 |
| Dendrolobium lanceolatum | 109.1191 | 19.05985 | -13271 | 293304.6 |
| Dendrolobium lanceolatum | 109.1741 | 18.37558 | -9781.56 | 217466.3 |
| Dendrolobium lanceolatum | 109.3011 | 18.85155 | 5180.119 | 269701.6 |
| Dendrolobium lanceolatum | 109.5146 | 18.9427 | 27935.88 | 279133.3 |
| Dendrolobium lanceolatum | 110.1338 | 18.56051 | 92062.33 | 235164.3 |
| Dendrolobium lanceolatum | 110.1876 | 19.13499 | 99259.69 | 298557.2 |
| Dendrolobium lanceolatum | 110.0333 | 19.77435 |  |  |
| Dendrolobium lanceolatum | 110.0751 | 19.70913 |  |  |
| Dendrolobium lanceolatum | 110.0526 | 19.80918 |  |  |
| Dendrolobium lanceolatum | 109.9718 | 19.75611 |  |  |
| Dendrolobium lanceolatum | 109.6482 | 19.4809 |  |  |
| Dendrolobium lanceolatum | 109.7191 | 19.46162 |  |  |
| Dendrolobium lanceolatum | 109.5715 | 19.57368 |  |  |
| Dendrolobium lanceolatum | 109.6871 | 19.49652 |  |  |
| Dendrolobium lanceolatum | 109.6235 | 19.54607 |  |  |
| Dendrolobium lanceolatum | 110.0861 | 18.52863 |  |  |
| Dendrolobium lanceolatum | 110.0745 | 18.55178 |  |  |
| Dendrolobium lanceolatum | 110.0547 | 18.52711 |  |  |
| Dendrolobium lanceolatum | 109.0679 | 19.32588 |  |  |
| Dendrolobium lanceolatum | 109.1026 | 19.32002 |  |  |
| Dendrolobium lanceolatum | 109.1381 | 19.35582 |  |  |
| Dendrolobium lanceolatum | 108.7472 | 19.06345 |  |  |
| Dendrolobium lanceolatum | 108.7455 | 19.06669 |  |  |
| Dendrolobium lanceolatum | 109.7771 | 18.57256 |  |  |
| Dendrolobium lanceolatum | 109.6568 | 18.24902 |  |  |
| Dendrolobium lanceolatum | 109.6506 | 18.25551 |  |  |
| Dendrolobium triangulare | 109.1741 | 18.37558 | -9781.56 | 217466.3 |
| Dendrolobium triangulare | 109.5146 | 18.9427 | 27935.88 | 279133.3 |
| Dendrolobium triangulare | 109.6807 | 18.37829 | 43729.57 | 216234.2 |
| Dendrolobium triangulare | 109.837 | 18.73456 | 61260.82 | 255199 |
| Dendrolobium triangulare | 110.2125 | 18.73421 | 100828.3 | 254175.6 |
| Dendrolobium triangulare | 109.637 | 19.90318 |  |  |
| Dendrolobium triangulare | 109.6431 | 19.87139 |  |  |
| Dendrolobium triangulare | 109.6437 | 19.8708 |  |  |
| Dendrolobium triangulare | 109.6153 | 19.90021 |  |  |
| Dendrolobium triangulare | 109.6151 | 19.8895 |  |  |
| Dendrolobium triangulare | 109.7147 | 19.48405 |  |  |
| Dendrolobium triangulare | 109.63 | 19.42522 |  |  |
| Dendrolobium triangulare | 109.5947 | 19.53325 |  |  |
| Dendrolobium triangulare | 109.5679 | 19.54701 |  |  |
| Dendrolobium triangulare | 109.6024 | 19.49532 |  |  |
| Dendrolobium triangulare | 109.6357 | 19.55436 |  |  |
| Dendrolobium triangulare | 110.2095 | 19.56831 |  |  |
| Dendrolobium triangulare | 110.2054 | 19.65208 |  |  |
| Dendrolobium triangulare | 110.2542 | 19.52476 |  |  |
| Dendrolobium triangulare | 109.0959 | 19.3625 |  |  |
| Dendrolobium triangulare | 109.1686 | 19.34273 |  |  |
| Dendrolobium triangulare | 109.4713 | 19.21842 |  |  |
| Dendrolobium triangulare | 109.4656 | 19.21692 |  |  |
| Dendrolobium triangulare | 109.815 | 18.57839 |  |  |
| Dendrolobium triangulare | 109.902 | 18.98073 |  |  |
| Dendropanax dentiger | 108.9795 | 18.89322 | -28525.9 | 275338 |
| Dendropanax dentiger | 110.0914 | 18.76565 | 88154.38 | 257960.3 |
| Dendropanax dentiger | 109.2828 | 18.77899 |  |  |
| Dendropanax dentiger | 109.2832 | 18.79946 |  |  |
| Dendropanax dentiger | 109.2852 | 18.78448 |  |  |
| Dendropanax dentiger | 109.2961 | 18.79948 |  |  |
| Dendropanax dentiger | 109.2862 | 18.79208 |  |  |
| Dendropanax dentiger | 109.2993 | 18.79436 |  |  |
| Dendropanax dentiger | 109.2961 | 18.79879 |  |  |
| Dendropanax dentiger | 109.2788 | 18.78601 |  |  |
| Dendropanax dentiger | 109.2888 | 18.7778 |  |  |
| Dendropanax dentiger | 109.8707 | 18.59977 |  |  |
| Dendropanax dentiger | 109.8587 | 18.59646 |  |  |
| Dendropanax dentiger | 109.8554 | 18.5968 |  |  |
| Dendropanax dentiger | 109.8546 | 18.59626 |  |  |
| Dendropanax dentiger | 109.8635 | 18.59966 |  |  |
| Dendropanax dentiger | 109.859 | 18.60828 |  |  |
| Dendropanax dentiger | 109.8626 | 18.60385 |  |  |
| Dendropanax dentiger | 109.854 | 18.6086 |  |  |
| Dendropanax dentiger | 109.8618 | 18.6022 |  |  |
| Dendropanax dentiger | 109.8685 | 18.60589 |  |  |
| Dendropanax dentiger | 109.8656 | 18.59675 |  |  |
| Dendropanax hainanensis | 108.9795 | 18.89322 | -28525.9 | 275338 |
| Dendropanax hainanensis | 109.0333 | 18.86036 | -22982.8 | 271527.7 |
| Dendropanax hainanensis | 109.6835 | 18.87437 | 45503.17 | 271088.8 |
| Dendropanax hainanensis | 109.6835 | 18.87437 | 45503.28 | 271088.9 |
| Dendropanax hainanensis | 109.6835 | 18.87437 | 45503.38 | 271089.1 |
| Dendropanax hainanensis | 109.3947 | 19.233 |  |  |
| Dendropanax hainanensis | 109.3842 | 19.23068 |  |  |
| Dendropanax hainanensis | 109.3877 | 19.23372 |  |  |
| Dendropanax hainanensis | 109.3886 | 19.23492 |  |  |
| Dendropanax hainanensis | 109.3932 | 19.23314 |  |  |
| Dendropanax hainanensis | 109.3906 | 19.23148 |  |  |
| Dendropanax hainanensis | 109.3915 | 19.23609 |  |  |
| Dendropanax hainanensis | 109.3929 | 19.23507 |  |  |
| Dendropanax hainanensis | 109.2864 | 18.78753 |  |  |
| Dendropanax hainanensis | 109.2762 | 18.79705 |  |  |
| Dendropanax hainanensis | 109.2912 | 18.78526 |  |  |
| Dendropanax hainanensis | 109.2841 | 18.78567 |  |  |
| Dendropanax hainanensis | 109.2952 | 18.77739 |  |  |
| Dendropanax hainanensis | 109.2793 | 18.78987 |  |  |
| Dendropanax hainanensis | 109.2934 | 18.78423 |  |  |
| Dendropanax hainanensis | 109.2841 | 18.79863 |  |  |
| Dendropanax hainanensis | 109.2806 | 18.782 |  |  |
| Dendropanax hainanensis | 109.2746 | 18.77783 |  |  |
| Dendropanax hainanensis | 109.2834 | 18.79363 |  |  |
| Dendropanax hainanensis | 109.2924 | 18.7801 |  |  |
| Dendropanax oligodontus | 109.4401 | 18.69801 | 19324.7 | 252298.9 |
| Dendropanax oligodontus | 109.6835 | 18.87437 | 45503.17 | 271088.8 |
| Dendropanax oligodontus | 109.8795 | 18.7193 | 65691.55 | 253395.3 |
| Dendropanax oligodontus | 109.9249 | 18.56104 | 70034.61 | 235770.5 |
| Dendropanax oligodontus | 110.3487 | 19.26196 | 116503.7 | 312200.6 |
| Dendropanax oligodontus | 109.7764 | 18.56571 |  |  |
| Dendropanax oligodontus | 109.7676 | 18.57311 |  |  |
| Dendropanax oligodontus | 109.7772 | 18.57245 |  |  |
| Dendropanax oligodontus | 109.7825 | 18.56196 |  |  |
| Dendropanax oligodontus | 109.7796 | 18.5739 |  |  |
| Dendropanax oligodontus | 109.7665 | 18.55903 |  |  |
| Dendropanax oligodontus | 109.8318 | 18.57533 |  |  |
| Dendropanax oligodontus | 109.8136 | 18.57864 |  |  |
| Dendropanax oligodontus | 109.8161 | 18.57298 |  |  |
| Dendropanax oligodontus | 109.8245 | 18.58197 |  |  |
| Dendropanax oligodontus | 109.8324 | 18.57365 |  |  |
| Dendropanax oligodontus | 109.8645 | 18.60495 |  |  |
| Dendropanax oligodontus | 109.8513 | 18.6087 |  |  |
| Dendropanax oligodontus | 109.8618 | 18.60125 |  |  |
| Dendropanax oligodontus | 109.8511 | 18.59782 |  |  |
| Dendropanax oligodontus | 109.855 | 18.60702 |  |  |
| Dendropanax oligodontus | 109.868 | 18.606 |  |  |
| Dendropanax oligodontus | 109.8664 | 18.60216 |  |  |
| Dendropanax oligodontus | 109.8577 | 18.5997 |  |  |
| Dendropanax oligodontus | 109.8568 | 18.60003 |  |  |
| Dendropanax proteus | 110.0914 | 18.76565 | 88154.38 | 257960.3 |
| Dendropanax proteus | 110.0915 | 18.76565 | 88154.6 | 257960.4 |
| Dendropanax proteus | 110.0915 | 18.76565 | 88154.7 | 257960.5 |
| Dendropanax proteus | 110.0915 | 18.76565 | 88154.81 | 257960.7 |
| Dendropanax proteus | 110.0915 | 18.76566 | 88154.92 | 257960.8 |
| Dendropanax proteus | 109.5349 | 18.72689 |  |  |
| Dendropanax proteus | 108.959 | 19.18519 |  |  |
| Dendropanax proteus | 109.6674 | 18.69037 |  |  |
| Dendropanax proteus | 109.1534 | 19.0214 |  |  |
| Dendropanax proteus | 109.3303 | 18.98093 |  |  |
| Dendropanax proteus | 109.7923 | 19.06489 |  |  |
| Dendropanax proteus | 109.1257 | 19.06374 |  |  |
| Dendropanax proteus | 109.4099 | 18.98592 |  |  |
| Dendropanax proteus | 109.2881 | 19.23852 |  |  |
| Dendropanax proteus | 109.6647 | 19.65435 |  |  |
| Dendropanax proteus | 109.6687 | 19.54997 |  |  |
| Dendropanax proteus | 109.8834 | 19.57295 |  |  |
| Dendropanax proteus | 110.415 | 19.53823 |  |  |
| Dendropanax proteus | 110.0476 | 19.16381 |  |  |
| Dendropanax proteus | 109.9396 | 19.38903 |  |  |
| Dendropanax proteus | 109.8143 | 19.31042 |  |  |
| Dendropanax proteus | 109.5924 | 19.20169 |  |  |
| Dendropanax proteus | 110.2953 | 19.51983 |  |  |
| Dendropanax proteus | 109.7983 | 19.62352 |  |  |
| Dendropanax proteus | 110.2284 | 19.40979 |  |  |
| Dendrophthoe pentandra | 109.5146 | 18.9427 | 27935.88 | 279133.3 |
| Dendrophthoe pentandra | 109.8563 | 18.6009 |  |  |
| Dendrophthoe pentandra | 109.8642 | 18.60068 |  |  |
| Dendrophthoe pentandra | 109.8573 | 18.60458 |  |  |
| Dendrophthoe pentandra | 109.5955 | 18.22232 |  |  |
| Dendrophthoe pentandra | 109.593 | 18.22281 |  |  |
| Dendrophthoe pentandra | 109.5989 | 18.22225 |  |  |
| Dendrophthoe pentandra | 109.5921 | 18.22639 |  |  |
| Dendrophthoe pentandra | 109.352 | 19.21249 |  |  |
| Dendrophthoe pentandra | 109.355 | 19.21991 |  |  |
| Dendrophthoe pentandra | 109.3457 | 19.21639 |  |  |
| Dendrophthoe pentandra | 109.3462 | 19.21572 |  |  |
| Dendrophthoe pentandra | 110.4069 | 18.76113 |  |  |
| Dendrophthoe pentandra | 110.428 | 18.74623 |  |  |
| Dendrophthoe pentandra | 110.4103 | 18.78193 |  |  |
| Dendrophthoe pentandra | 110.0786 | 18.55723 |  |  |
| Dendrophthoe pentandra | 110.0944 | 18.54869 |  |  |
| Dendrophthoe pentandra | 110.0806 | 18.51261 |  |  |
| Dendrophthoe pentandra | 109.2972 | 18.78574 |  |  |
| Dendrophthoe pentandra | 109.2938 | 18.79092 |  |  |
| Dendrophthoe pentandra | 109.5819 | 19.57362 |  |  |
| Dendrotrophe varians | 109.416 | 18.58215 | 16423.77 | 239559.1 |
| Dendrotrophe varians | 109.4718 | 19.16737 | 24145.48 | 304100.4 |
| Dendrotrophe varians | 109.7233 | 19.12658 |  |  |
| Dendrotrophe varians | 109.4006 | 18.65555 |  |  |
| Dendrotrophe varians | 109.1827 | 19.03637 |  |  |
| Dendrotrophe varians | 109.2789 | 18.865 |  |  |
| Dendrotrophe varians | 109.1073 | 19.1729 |  |  |
| Dendrotrophe varians | 109.926 | 19.01475 |  |  |
| Dendrotrophe varians | 109.2568 | 18.97332 |  |  |
| Dendrotrophe varians | 109.8273 | 18.9941 |  |  |
| Dendrotrophe varians | 109.9483 | 18.66966 |  |  |
| Dendrotrophe varians | 110.2286 | 19.64539 |  |  |
| Dendrotrophe varians | 110.5051 | 19.78625 |  |  |
| Dendrotrophe varians | 109.5897 | 19.79793 |  |  |
| Dendrotrophe varians | 109.6543 | 19.31674 |  |  |
| Dendrotrophe varians | 109.8199 | 19.65175 |  |  |
| Dendrotrophe varians | 109.9912 | 19.45625 |  |  |
| Dendrotrophe varians | 110.4339 | 19.62558 |  |  |
| Dendrotrophe varians | 110.152 | 19.47037 |  |  |
| Dendrotrophe varians | 110.3543 | 19.31455 |  |  |
| Dendrotrophe varians | 109.6653 | 19.21194 |  |  |
| Dendrotrophe varians | 109.7535 | 19.62952 |  |  |
| Derris alborubra | 109.5153 | 18.61925 | 27012 | 243364.4 |
| Derris alborubra | 109.5409 | 18.34239 | 28849.41 | 212669.7 |
| Derris alborubra | 110.2256 | 19.86031 | 105147.4 | 378650.1 |
| Derris alborubra | 110.2549 | 18.71143 | 105232.9 | 251551.4 |
| Derris alborubra | 110.4321 | 19.37563 | 125548.1 | 324570.5 |
| Derris alborubra | 110.4275 | 19.27682 |  |  |
| Derris alborubra | 110.4334 | 19.33198 |  |  |
| Derris alborubra | 110.3844 | 19.29292 |  |  |
| Derris alborubra | 110.4313 | 19.32153 |  |  |
| Derris alborubra | 110.3573 | 19.30506 |  |  |
| Derris alborubra | 110.3587 | 19.31136 |  |  |
| Derris alborubra | 110.389 | 19.28478 |  |  |
| Derris alborubra | 109.8305 | 18.57969 |  |  |
| Derris alborubra | 109.8286 | 18.5786 |  |  |
| Derris alborubra | 109.8139 | 18.57532 |  |  |
| Derris alborubra | 109.8278 | 18.58542 |  |  |
| Derris alborubra | 109.8258 | 18.58233 |  |  |
| Derris alborubra | 109.8249 | 18.57855 |  |  |
| Derris alborubra | 110.3816 | 18.7753 |  |  |
| Derris alborubra | 110.3752 | 18.77756 |  |  |
| Derris alborubra | 110.3738 | 18.77219 |  |  |
| Derris alborubra | 110.3753 | 18.77637 |  |  |
| Derris alborubra | 110.3719 | 18.7707 |  |  |
| Derris alborubra | 110.3797 | 18.7755 |  |  |
| Derris alborubra | 110.3814 | 18.77255 |  |  |
| Derris fordii | 109.1544 | 19.01211 | -9728.08 | 287914.8 |
| Derris fordii | 109.1544 | 19.01212 | -9727.97 | 287914.9 |
| Derris fordii | 109.1544 | 19.01212 | -9727.86 | 287915 |
| Derris fordii | 109.1544 | 19.01212 | -9727.75 | 287915.1 |
| Derris fordii | 109.1544 | 19.01212 | -9727.65 | 287915.2 |
| Derris fordii | 109.0787 | 19.35358 |  |  |
| Derris fordii | 109.0822 | 19.33085 |  |  |
| Derris fordii | 109.1417 | 19.34695 |  |  |
| Derris fordii | 109.1598 | 19.3149 |  |  |
| Derris fordii | 109.1214 | 19.34607 |  |  |
| Derris fordii | 109.164 | 19.34541 |  |  |
| Derris fordii | 109.7313 | 19.43135 |  |  |
| Derris fordii | 109.6965 | 19.48537 |  |  |
| Derris fordii | 109.7095 | 19.50496 |  |  |
| Derris fordii | 109.5758 | 19.46884 |  |  |
| Derris fordii | 109.6276 | 19.57421 |  |  |
| Derris fordii | 109.5794 | 19.56947 |  |  |
| Derris fordii | 108.7394 | 19.04185 |  |  |
| Derris fordii | 108.7411 | 19.05294 |  |  |
| Derris fordii | 108.7446 | 19.04483 |  |  |
| Derris fordii | 108.7342 | 19.04965 |  |  |
| Derris fordii | 109.7702 | 18.56922 |  |  |
| Derris fordii | 109.7848 | 18.55841 |  |  |
| Derris fordii | 109.7836 | 18.5591 |  |  |
| Derris fordii | 109.7839 | 18.57388 |  |  |
| Derris trifoliata | 110.2332 | 18.6893 | 102892.4 | 249157.1 |
| Derris trifoliata | 110.2549 | 18.71143 | 105232.9 | 251551.4 |
| Derris trifoliata | 110.6146 | 19.99826 | 146153.1 | 392988.4 |
| Derris trifoliata | 109.653 | 18.25067 |  |  |
| Derris trifoliata | 109.6525 | 18.2572 |  |  |
| Derris trifoliata | 109.6522 | 18.25468 |  |  |
| Derris trifoliata | 109.6562 | 18.25231 |  |  |
| Derris trifoliata | 109.6523 | 18.25318 |  |  |
| Derris trifoliata | 109.655 | 18.25219 |  |  |
| Derris trifoliata | 109.6501 | 18.25266 |  |  |
| Derris trifoliata | 110.0914 | 18.54473 |  |  |
| Derris trifoliata | 110.0874 | 18.52819 |  |  |
| Derris trifoliata | 110.0891 | 18.54686 |  |  |
| Derris trifoliata | 110.0681 | 18.55837 |  |  |
| Derris trifoliata | 110.1007 | 18.53924 |  |  |
| Derris trifoliata | 110.087 | 18.56189 |  |  |
| Derris trifoliata | 110.84 | 19.56469 |  |  |
| Derris trifoliata | 110.916 | 19.55566 |  |  |
| Derris trifoliata | 110.8024 | 19.65919 |  |  |
| Derris trifoliata | 110.8688 | 19.70178 |  |  |
| Derris trifoliata | 110.7936 | 19.69906 |  |  |
| Derris trifoliata | 110.8509 | 19.54503 |  |  |
| Derris trifoliata | 110.7683 | 19.70482 |  |  |
| Desmodium gangeticum | 108.9122 | 18.99917 | -35232.9 | 287275.8 |
| Desmodium gangeticum | 109.0367 | 19.17511 | -21544.3 | 306313.7 |
| Desmodium gangeticum | 109.0799 | 19.00987 | -17563.9 | 287906.3 |
| Desmodium gangeticum | 109.1741 | 18.37558 | -9781.56 | 217466.3 |
| Desmodium gangeticum | 109.4865 | 19.15807 | 25651.28 | 303028.8 |
| Desmodium gangeticum | 109.4916 | 18.78671 | 25037.28 | 261953.3 |
| Desmodium gangeticum | 109.7426 | 19.4888 | 53537.07 | 338853.4 |
| Desmodium gangeticum | 109.837 | 18.73456 | 61260.82 | 255199 |
| Desmodium gangeticum | 110.2332 | 18.6893 | 102892.4 | 249157.1 |
| Desmodium gangeticum | 109.7178 | 19.53523 |  |  |
| Desmodium gangeticum | 109.6009 | 19.56129 |  |  |
| Desmodium gangeticum | 109.593 | 19.5605 |  |  |
| Desmodium gangeticum | 109.6494 | 19.53713 |  |  |
| Desmodium gangeticum | 109.9947 | 19.79084 |  |  |
| Desmodium gangeticum | 109.9491 | 19.78656 |  |  |
| Desmodium gangeticum | 110.0074 | 19.73139 |  |  |
| Desmodium gangeticum | 110.0729 | 19.8097 |  |  |
| Desmodium gangeticum | 109.5993 | 18.25701 |  |  |
| Desmodium gangeticum | 109.5992 | 18.26098 |  |  |
| Desmodium gangeticum | 109.8629 | 18.60144 |  |  |
| Desmodium gangeticum | 109.8512 | 18.60722 |  |  |
| Desmodium gangeticum | 109.8634 | 18.6043 |  |  |
| Desmodium gangeticum | 109.8655 | 18.59814 |  |  |
| Desmodium gangeticum | 109.1345 | 19.31191 |  |  |
| Desmodium gangeticum | 109.1505 | 19.36259 |  |  |
| Desmodium gangeticum | 109.1354 | 19.35943 |  |  |
| Desmodium gangeticum | 108.7463 | 19.06594 |  |  |
| Desmodium gangeticum | 108.7458 | 19.06602 |  |  |
| Desmodium gangeticum | 109.4685 | 19.21887 |  |  |
| Desmodium heterocarpon | 109.4916 | 18.78671 | 25037.28 | 261953.3 |
| Desmodium heterocarpon | 109.6579 | 18.76335 | 42477.13 | 258885.1 |
| Desmodium heterocarpon | 110.1756 | 18.76435 | 97019.43 | 257602 |
| Desmodium heterocarpon | 110.2238 | 18.67552 | 101866.1 | 247657.4 |
| Desmodium heterocarpon | 110.6819 | 19.92734 | 153022.5 | 385001.2 |
| Desmodium heterocarpon | 109.7703 | 18.57018 |  |  |
| Desmodium heterocarpon | 109.7645 | 18.57305 |  |  |
| Desmodium heterocarpon | 109.7697 | 18.56431 |  |  |
| Desmodium heterocarpon | 109.8203 | 18.576 |  |  |
| Desmodium heterocarpon | 109.8341 | 18.57923 |  |  |
| Desmodium heterocarpon | 109.8256 | 18.58256 |  |  |
| Desmodium heterocarpon | 109.8606 | 18.60339 |  |  |
| Desmodium heterocarpon | 109.8659 | 18.59979 |  |  |
| Desmodium heterocarpon | 109.8542 | 18.60204 |  |  |
| Desmodium heterocarpon | 109.8616 | 18.59918 |  |  |
| Desmodium heterocarpon | 109.862 | 18.60446 |  |  |
| Desmodium heterocarpon | 110.29 | 19.59149 |  |  |
| Desmodium heterocarpon | 110.2949 | 19.63795 |  |  |
| Desmodium heterocarpon | 110.3065 | 19.58276 |  |  |
| Desmodium heterocarpon | 110.2384 | 19.55415 |  |  |
| Desmodium heterocarpon | 110.3096 | 19.58557 |  |  |
| Desmodium heterocarpon | 110.2051 | 19.55381 |  |  |
| Desmodium heterocarpon | 110.253 | 19.60724 |  |  |
| Desmodium heterocarpon | 110.3205 | 19.64124 |  |  |
| Desmodium heterocarpon | 110.3089 | 19.67244 |  |  |
| Desmodium laxiflorum | 109.3116 | 18.96725 | 6666.857 | 282460.4 |
| Desmodium laxiflorum | 109.4273 | 19.01928 | 18993.09 | 287860.9 |
| Desmodium laxiflorum | 109.4273 | 19.01928 | 18993.19 | 287860.6 |
| Desmodium laxiflorum | 109.4273 | 19.01928 | 18993.29 | 287860.5 |
| Desmodium laxiflorum | 109.4273 | 19.01927 | 18993.39 | 287860.3 |
| Desmodium laxiflorum | 109.3917 | 19.23154 |  |  |
| Desmodium laxiflorum | 109.3892 | 19.23614 |  |  |
| Desmodium laxiflorum | 109.3942 | 19.23001 |  |  |
| Desmodium laxiflorum | 109.3953 | 19.23416 |  |  |
| Desmodium laxiflorum | 109.3891 | 19.23101 |  |  |
| Desmodium laxiflorum | 109.3918 | 19.2338 |  |  |
| Desmodium laxiflorum | 109.3958 | 19.23024 |  |  |
| Desmodium laxiflorum | 109.393 | 19.23179 |  |  |
| Desmodium laxiflorum | 109.3474 | 19.21979 |  |  |
| Desmodium laxiflorum | 109.3486 | 19.22032 |  |  |
| Desmodium laxiflorum | 109.3493 | 19.21967 |  |  |
| Desmodium laxiflorum | 109.3536 | 19.21657 |  |  |
| Desmodium laxiflorum | 109.3504 | 19.21911 |  |  |
| Desmodium laxiflorum | 109.3453 | 19.21738 |  |  |
| Desmodium laxiflorum | 109.3471 | 19.21694 |  |  |
| Desmodium laxiflorum | 109.3529 | 19.21965 |  |  |
| Desmodium laxiflorum | 109.347 | 19.21919 |  |  |
| Desmodium laxiflorum | 109.3546 | 19.217 |  |  |
| Desmodium laxiflorum | 109.3517 | 19.21748 |  |  |
| Desmodium laxiflorum | 109.3471 | 19.21586 |  |  |
| Desmodium velutinum | 109.3149 | 18.41558 | 5219.958 | 221446.7 |
| Desmodium velutinum | 109.4401 | 18.69801 | 19324.7 | 252298.9 |
| Desmodium velutinum | 109.4916 | 18.78671 | 25037.28 | 261953.3 |
| Desmodium velutinum | 109.5064 | 18.29956 | 25081.02 | 208034 |
| Desmodium velutinum | 109.8362 | 19.95414 | 64688.88 | 390034 |
| Desmodium velutinum | 110.0498 | 19.79725 |  |  |
| Desmodium velutinum | 109.9881 | 19.75997 |  |  |
| Desmodium velutinum | 110.0013 | 19.72563 |  |  |
| Desmodium velutinum | 110.0169 | 19.74342 |  |  |
| Desmodium velutinum | 110.0168 | 19.74163 |  |  |
| Desmodium velutinum | 109.9848 | 19.73522 |  |  |
| Desmodium velutinum | 109.6815 | 19.95183 |  |  |
| Desmodium velutinum | 109.6786 | 19.95963 |  |  |
| Desmodium velutinum | 109.6804 | 19.96004 |  |  |
| Desmodium velutinum | 109.6848 | 19.96358 |  |  |
| Desmodium velutinum | 109.6018 | 19.88014 |  |  |
| Desmodium velutinum | 109.6415 | 19.87059 |  |  |
| Desmodium velutinum | 109.5983 | 19.87115 |  |  |
| Desmodium velutinum | 109.6367 | 19.87104 |  |  |
| Desmodium velutinum | 110.2107 | 19.5786 |  |  |
| Desmodium velutinum | 110.2624 | 19.53511 |  |  |
| Desmodium velutinum | 110.3034 | 19.60799 |  |  |
| Desmodium velutinum | 110.2945 | 19.61549 |  |  |
| Desmodium velutinum | 110.2805 | 19.60927 |  |  |
| Desmodium velutinum | 110.247 | 19.53107 |  |  |
| Desmos chinensis | 108.7989 | 18.69619 | -48238.4 | 254162.2 |
| Desmos chinensis | 109.0799 | 19.00987 | -17563.9 | 287906.3 |
| Desmos chinensis | 109.142 | 19.12428 | -10653 | 300354.3 |
| Desmos chinensis | 109.5259 | 19.13355 | 29723.31 | 300201.6 |
| Desmos chinensis | 109.5425 | 19.03583 | 31162.38 | 289348.8 |
| Desmos chinensis | 109.2389 | 18.74242 |  |  |
| Desmos chinensis | 109.9399 | 18.98349 |  |  |
| Desmos chinensis | 109.6059 | 19.15074 |  |  |
| Desmos chinensis | 109.5334 | 19.18936 |  |  |
| Desmos chinensis | 108.9428 | 19.22046 |  |  |
| Desmos chinensis | 110.0467 | 18.88751 |  |  |
| Desmos chinensis | 109.8229 | 19.11475 |  |  |
| Desmos chinensis | 109.8179 | 18.81975 |  |  |
| Desmos chinensis | 109.7172 | 18.9078 |  |  |
| Desmos chinensis | 110.2893 | 19.44501 |  |  |
| Desmos chinensis | 110.039 | 19.21545 |  |  |
| Desmos chinensis | 109.7169 | 19.46825 |  |  |
| Desmos chinensis | 110.2372 | 19.81945 |  |  |
| Desmos chinensis | 109.6196 | 19.83061 |  |  |
| Desmos chinensis | 109.9186 | 19.59196 |  |  |
| Desmos chinensis | 109.9975 | 19.35778 |  |  |
| Desmos chinensis | 109.5968 | 19.46239 |  |  |
| Desmos chinensis | 109.8728 | 19.81769 |  |  |
| Desmos chinensis | 109.9673 | 19.36319 |  |  |
| Desmos chinensis | 109.8488 | 19.19893 |  |  |
| Dichapetalum gelonioides | 109.0799 | 19.00987 | -17563.9 | 287906.3 |
| Dichapetalum gelonioides | 109.6006 | 18.43923 | 35449.67 | 223205.2 |
| Dichapetalum gelonioides | 109.6036 | 18.37017 | 35565.66 | 215558.3 |
| Dichapetalum gelonioides | 109.837 | 18.73456 | 61260.82 | 255199 |
| Dichapetalum gelonioides | 110.2356 | 18.69662 | 103162.5 | 249961.2 |
| Dichapetalum gelonioides | 110.0862 | 18.54226 |  |  |
| Dichapetalum gelonioides | 110.0812 | 18.55514 |  |  |
| Dichapetalum gelonioides | 110.0747 | 18.51401 |  |  |
| Dichapetalum gelonioides | 110.0666 | 18.53441 |  |  |
| Dichapetalum gelonioides | 110.094 | 18.52284 |  |  |
| Dichapetalum gelonioides | 110.4241 | 18.81069 |  |  |
| Dichapetalum gelonioides | 110.4251 | 18.79399 |  |  |
| Dichapetalum gelonioides | 110.4266 | 18.81194 |  |  |
| Dichapetalum gelonioides | 110.4294 | 18.79379 |  |  |
| Dichapetalum gelonioides | 110.4371 | 18.79846 |  |  |
| Dichapetalum gelonioides | 108.7992 | 18.9832 |  |  |
| Dichapetalum gelonioides | 108.8006 | 18.989 |  |  |
| Dichapetalum gelonioides | 108.8103 | 18.97665 |  |  |
| Dichapetalum gelonioides | 108.7974 | 18.96121 |  |  |
| Dichapetalum gelonioides | 109.8515 | 18.6029 |  |  |
| Dichapetalum gelonioides | 109.8567 | 18.60752 |  |  |
| Dichapetalum gelonioides | 109.8707 | 18.59878 |  |  |
| Dichapetalum gelonioides | 109.595 | 18.26208 |  |  |
| Dichapetalum gelonioides | 109.5997 | 18.25814 |  |  |
| Dichapetalum gelonioides | 109.6003 | 18.26145 |  |  |
| Dichroa febrifuga | 109.0333 | 18.86036 | -22982.8 | 271527.7 |
| Dichroa febrifuga | 109.0799 | 19.00987 | -17563.9 | 287906.3 |
| Dichroa febrifuga | 109.5146 | 18.9427 | 27935.88 | 279133.3 |
| Dichroa febrifuga | 109.3515 | 19.21408 |  |  |
| Dichroa febrifuga | 109.3457 | 19.20988 |  |  |
| Dichroa febrifuga | 109.3455 | 19.21948 |  |  |
| Dichroa febrifuga | 109.3511 | 19.21949 |  |  |
| Dichroa febrifuga | 109.3523 | 19.21249 |  |  |
| Dichroa febrifuga | 109.7273 | 19.5131 |  |  |
| Dichroa febrifuga | 109.7227 | 19.48123 |  |  |
| Dichroa febrifuga | 109.6345 | 19.57677 |  |  |
| Dichroa febrifuga | 110.0071 | 19.80218 |  |  |
| Dichroa febrifuga | 109.9961 | 19.73561 |  |  |
| Dichroa febrifuga | 109.9548 | 19.71688 |  |  |
| Dichroa febrifuga | 110.3951 | 19.30197 |  |  |
| Dichroa febrifuga | 110.3805 | 19.29182 |  |  |
| Dichroa febrifuga | 110.3855 | 19.31409 |  |  |
| Dichroa febrifuga | 108.9921 | 18.70017 |  |  |
| Dichroa febrifuga | 108.9988 | 18.71067 |  |  |
| Dichroa febrifuga | 109.0067 | 18.70509 |  |  |
| Dichroa febrifuga | 108.7459 | 19.06629 |  |  |
| Dichroa febrifuga | 109.8144 | 18.58337 |  |  |
| Dichroa febrifuga | 109.8252 | 19.02557 |  |  |
| Diospyros cathayensis | 109.0333 | 18.86036 | -22982.8 | 271527.7 |
| Diospyros cathayensis | 109.1215 | 19.14164 | -12747.8 | 302339.7 |
| Diospyros cathayensis | 109.9566 | 18.66988 | 73677.56 | 247722.8 |
| Diospyros cathayensis | 110.001 | 18.75027 | 78585.68 | 256495.3 |
| Diospyros cathayensis | 110.3082 | 19.17786 | 112041.1 | 302999.2 |
| Diospyros cathayensis | 109.8594 | 18.59741 |  |  |
| Diospyros cathayensis | 109.858 | 18.60474 |  |  |
| Diospyros cathayensis | 109.8633 | 18.59699 |  |  |
| Diospyros cathayensis | 109.8577 | 18.60148 |  |  |
| Diospyros cathayensis | 109.8712 | 18.60131 |  |  |
| Diospyros cathayensis | 109.856 | 18.60841 |  |  |
| Diospyros cathayensis | 109.8584 | 18.59528 |  |  |
| Diospyros cathayensis | 109.8695 | 18.60535 |  |  |
| Diospyros cathayensis | 109.8506 | 18.60837 |  |  |
| Diospyros cathayensis | 109.8869 | 18.79442 |  |  |
| Diospyros cathayensis | 109.8727 | 18.79822 |  |  |
| Diospyros cathayensis | 109.8694 | 18.79294 |  |  |
| Diospyros cathayensis | 109.8719 | 18.79995 |  |  |
| Diospyros cathayensis | 109.8749 | 18.79616 |  |  |
| Diospyros cathayensis | 109.8861 | 18.78924 |  |  |
| Diospyros cathayensis | 109.8882 | 18.78954 |  |  |
| Diospyros cathayensis | 109.8694 | 18.79959 |  |  |
| Diospyros cathayensis | 109.8833 | 18.79944 |  |  |
| Diospyros cathayensis | 109.8691 | 18.79137 |  |  |
| Diospyros cathayensis | 109.8815 | 18.78671 |  |  |
| Diospyros diversilimba | 108.794 | 18.69511 | -48760 | 254058.8 |
| Diospyros diversilimba | 109.1191 | 19.05985 | -13271 | 293304.6 |
| Diospyros diversilimba | 109.2747 | 19.85903 | 5691.175 | 381153.9 |
| Diospyros diversilimba | 109.6535 | 18.2575 | 40487.08 | 202951.5 |
| Diospyros diversilimba | 109.7326 | 19.59786 | 52815.33 | 350936.9 |
| Diospyros diversilimba | 110.2738 | 18.67292 | 107120.4 | 247246.1 |
| Diospyros diversilimba | 109.6312 | 19.43335 |  |  |
| Diospyros diversilimba | 109.5821 | 19.54418 |  |  |
| Diospyros diversilimba | 109.7102 | 19.53365 |  |  |
| Diospyros diversilimba | 109.5777 | 19.54407 |  |  |
| Diospyros diversilimba | 109.7297 | 19.52858 |  |  |
| Diospyros diversilimba | 109.699 | 19.47236 |  |  |
| Diospyros diversilimba | 109.7324 | 19.53483 |  |  |
| Diospyros diversilimba | 110.049 | 19.73108 |  |  |
| Diospyros diversilimba | 109.9636 | 19.72766 |  |  |
| Diospyros diversilimba | 109.6814 | 19.96444 |  |  |
| Diospyros diversilimba | 109.6693 | 19.94549 |  |  |
| Diospyros diversilimba | 109.6798 | 19.95731 |  |  |
| Diospyros diversilimba | 109.6723 | 19.95562 |  |  |
| Diospyros diversilimba | 109.5992 | 18.22453 |  |  |
| Diospyros diversilimba | 109.5952 | 18.22275 |  |  |
| Diospyros diversilimba | 109.5936 | 18.22409 |  |  |
| Diospyros diversilimba | 109.0358 | 18.58933 |  |  |
| Diospyros diversilimba | 109.1472 | 18.56819 |  |  |
| Diospyros diversilimba | 109.0949 | 18.62236 |  |  |
| Diospyros diversilimba | 109.0859 | 18.56112 |  |  |
| Diospyros ehretioides | 109.1191 | 19.05985 | -13271 | 293304.6 |
| Diospyros ehretioides | 109.1215 | 19.14164 | -12747.8 | 302339.7 |
| Diospyros ehretioides | 109.3163 | 18.42284 | 5389.832 | 222245.7 |
| Diospyros ehretioides | 109.3163 | 18.42284 | 5389.94 | 222245.8 |
| Diospyros ehretioides | 109.6975 | 18.72833 | 46544.54 | 254900 |
| Diospyros ehretioides | 108.7437 | 19.06414 |  |  |
| Diospyros ehretioides | 108.7445 | 19.0641 |  |  |
| Diospyros ehretioides | 108.746 | 19.06502 |  |  |
| Diospyros ehretioides | 108.7459 | 19.06657 |  |  |
| Diospyros ehretioides | 108.746 | 19.06546 |  |  |
| Diospyros ehretioides | 109.6575 | 18.25081 |  |  |
| Diospyros ehretioides | 109.6539 | 18.25725 |  |  |
| Diospyros ehretioides | 109.6535 | 18.24914 |  |  |
| Diospyros ehretioides | 109.6573 | 18.25442 |  |  |
| Diospyros ehretioides | 109.8308 | 18.58315 |  |  |
| Diospyros ehretioides | 109.8145 | 18.57402 |  |  |
| Diospyros ehretioides | 109.8222 | 18.58079 |  |  |
| Diospyros ehretioides | 109.8197 | 18.58477 |  |  |
| Diospyros ehretioides | 109.3525 | 19.21385 |  |  |
| Diospyros ehretioides | 109.3511 | 19.21386 |  |  |
| Diospyros ehretioides | 109.3487 | 19.21596 |  |  |
| Diospyros ehretioides | 109.3463 | 19.21027 |  |  |
| Diospyros ehretioides | 109.073 | 18.74409 |  |  |
| Diospyros ehretioides | 109.0479 | 18.74317 |  |  |
| Diospyros ehretioides | 109.0211 | 18.71425 |  |  |
| Diospyros hainanensis | 109.0333 | 18.86036 | -22982.8 | 271527.7 |
| Diospyros hainanensis | 109.6975 | 18.72833 | 46544.54 | 254900 |
| Diospyros hainanensis | 109.8073 | 18.71975 | 58090.63 | 253642.9 |
| Diospyros hainanensis | 109.8188 | 19.14345 | 60528.46 | 300462.2 |
| Diospyros hainanensis | 109.837 | 18.73456 | 61260.82 | 255199 |
| Diospyros hainanensis | 109.9249 | 18.56104 | 70034.61 | 235770.5 |
| Diospyros hainanensis | 109.869 | 19.096 |  |  |
| Diospyros hainanensis | 109.8654 | 19.09313 |  |  |
| Diospyros hainanensis | 109.8692 | 19.09481 |  |  |
| Diospyros hainanensis | 109.8656 | 19.0934 |  |  |
| Diospyros hainanensis | 109.5443 | 19.03404 |  |  |
| Diospyros hainanensis | 109.5503 | 19.03744 |  |  |
| Diospyros hainanensis | 109.5467 | 19.029 |  |  |
| Diospyros hainanensis | 109.5441 | 19.03097 |  |  |
| Diospyros hainanensis | 109.5441 | 19.03534 |  |  |
| Diospyros hainanensis | 109.8659 | 18.79527 |  |  |
| Diospyros hainanensis | 109.8718 | 18.79684 |  |  |
| Diospyros hainanensis | 109.8726 | 18.79628 |  |  |
| Diospyros hainanensis | 109.8651 | 18.79629 |  |  |
| Diospyros hainanensis | 109.8879 | 18.79834 |  |  |
| Diospyros hainanensis | 109.884 | 18.79529 |  |  |
| Diospyros hainanensis | 109.8709 | 18.78501 |  |  |
| Diospyros hainanensis | 109.8737 | 18.79008 |  |  |
| Diospyros hainanensis | 109.8786 | 18.79814 |  |  |
| Diospyros hainanensis | 109.8884 | 18.79459 |  |  |
| Diospyros hainanensis | 109.8808 | 18.79953 |  |  |
| Diospyros howii | 109.6538 | 18.29652 | 40638.73 | 207266.3 |
| Diospyros howii | 110.001 | 18.75027 | 78585.68 | 256495.3 |
| Diospyros howii | 110.0558 | 18.52812 | 83749.62 | 231782.9 |
| Diospyros howii | 110.0914 | 18.76565 | 88154.38 | 257960.3 |
| Diospyros howii | 110.1756 | 18.76435 | 97019.43 | 257602 |
| Diospyros howii | 109.8691 | 18.78877 |  |  |
| Diospyros howii | 109.8844 | 18.78878 |  |  |
| Diospyros howii | 109.888 | 18.79778 |  |  |
| Diospyros howii | 109.8861 | 18.78772 |  |  |
| Diospyros howii | 109.8853 | 18.79689 |  |  |
| Diospyros howii | 109.8748 | 18.79295 |  |  |
| Diospyros howii | 109.8829 | 18.79833 |  |  |
| Diospyros howii | 109.8654 | 18.79885 |  |  |
| Diospyros howii | 109.8801 | 18.80161 |  |  |
| Diospyros howii | 109.8837 | 18.78813 |  |  |
| Diospyros howii | 109.8741 | 18.79972 |  |  |
| Diospyros howii | 109.8769 | 18.79346 |  |  |
| Diospyros howii | 109.8878 | 18.78959 |  |  |
| Diospyros howii | 109.8671 | 18.79156 |  |  |
| Diospyros howii | 109.8866 | 18.79227 |  |  |
| Diospyros howii | 109.8861 | 18.80136 |  |  |
| Diospyros howii | 109.8792 | 18.79606 |  |  |
| Diospyros howii | 109.8781 | 18.78916 |  |  |
| Diospyros howii | 109.8789 | 18.79364 |  |  |
| Diospyros howii | 109.8784 | 18.79723 |  |  |
| Diospyros kaki | 109.1191 | 19.05985 | -13271 | 293304.6 |
| Diospyros kaki | 109.6401 | 19.49205 | 42797.58 | 339502 |
| Diospyros kaki | 110.2542 | 18.78692 | 105355.2 | 259902.1 |
| Diospyros kaki | 110.4309 | 18.7955 |  |  |
| Diospyros kaki | 110.374 | 18.77099 |  |  |
| Diospyros kaki | 110.4087 | 18.78214 |  |  |
| Diospyros kaki | 110.427 | 18.7483 |  |  |
| Diospyros kaki | 110.4317 | 18.73256 |  |  |
| Diospyros kaki | 110.4046 | 18.74369 |  |  |
| Diospyros kaki | 110.4258 | 18.75021 |  |  |
| Diospyros kaki | 110.2159 | 20.01222 |  |  |
| Diospyros kaki | 110.215 | 20.01158 |  |  |
| Diospyros kaki | 110.2153 | 20.01221 |  |  |
| Diospyros kaki | 110.2161 | 20.01178 |  |  |
| Diospyros kaki | 110.4616 | 19.8562 |  |  |
| Diospyros kaki | 110.4624 | 19.85365 |  |  |
| Diospyros kaki | 110.4552 | 19.8585 |  |  |
| Diospyros kaki | 110.4633 | 19.85811 |  |  |
| Diospyros kaki | 110.4609 | 19.84955 |  |  |
| Diospyros kaki | 110.419 | 19.84852 |  |  |
| Diospyros kaki | 110.412 | 19.85511 |  |  |
| Diospyros kaki | 110.4136 | 19.85664 |  |  |
| Diospyros kaki | 110.4129 | 19.85153 |  |  |
| Diospyros longibracteata | 109.0333 | 18.86036 | -22982.8 | 271527.7 |
| Diospyros longibracteata | 109.869 | 18.69141 | 64504.44 | 250339.5 |
| Diospyros longibracteata | 110.0284 | 18.90832 | 81905.25 | 273901 |
| Diospyros longibracteata | 110.1756 | 18.76435 | 97019.43 | 257602 |
| Diospyros longibracteata | 108.7461 | 19.04265 |  |  |
| Diospyros longibracteata | 108.7356 | 19.03996 |  |  |
| Diospyros longibracteata | 108.7419 | 19.04434 |  |  |
| Diospyros longibracteata | 108.7351 | 19.04272 |  |  |
| Diospyros longibracteata | 109.3941 | 19.23448 |  |  |
| Diospyros longibracteata | 109.3907 | 19.23152 |  |  |
| Diospyros longibracteata | 109.3868 | 19.23594 |  |  |
| Diospyros longibracteata | 109.3896 | 19.2325 |  |  |
| Diospyros longibracteata | 109.7732 | 18.56509 |  |  |
| Diospyros longibracteata | 109.7847 | 18.57473 |  |  |
| Diospyros longibracteata | 109.7816 | 18.56994 |  |  |
| Diospyros longibracteata | 109.7663 | 18.57474 |  |  |
| Diospyros longibracteata | 109.6587 | 18.25329 |  |  |
| Diospyros longibracteata | 109.6511 | 18.25212 |  |  |
| Diospyros longibracteata | 109.6499 | 18.25059 |  |  |
| Diospyros longibracteata | 110.0555 | 18.55274 |  |  |
| Diospyros longibracteata | 110.0965 | 18.51557 |  |  |
| Diospyros longibracteata | 109.0346 | 18.52259 |  |  |
| Diospyros longibracteata | 109.0909 | 18.55084 |  |  |
| Diospyros longibracteata | 109.1008 | 18.52977 |  |  |
| Diospyros maclurei | 109.2292 | 18.32242 | -4135.79 | 211411.6 |
| Diospyros maclurei | 109.6828 | 18.3841 | 43968.29 | 216870.9 |
| Diospyros maclurei | 109.837 | 18.73456 | 61260.82 | 255199 |
| Diospyros maclurei | 110.001 | 18.75027 | 78585.68 | 256495.3 |
| Diospyros maclurei | 110.0914 | 18.76565 | 88154.38 | 257960.3 |
| Diospyros maclurei | 109.8676 | 18.79402 |  |  |
| Diospyros maclurei | 109.8675 | 18.7926 |  |  |
| Diospyros maclurei | 109.8691 | 18.79273 |  |  |
| Diospyros maclurei | 109.8845 | 18.80226 |  |  |
| Diospyros maclurei | 109.8714 | 18.80003 |  |  |
| Diospyros maclurei | 109.8706 | 18.79863 |  |  |
| Diospyros maclurei | 109.8656 | 18.79177 |  |  |
| Diospyros maclurei | 109.8792 | 18.78951 |  |  |
| Diospyros maclurei | 109.266 | 19.03589 |  |  |
| Diospyros maclurei | 109.272 | 19.03515 |  |  |
| Diospyros maclurei | 109.2619 | 19.03578 |  |  |
| Diospyros maclurei | 109.2644 | 19.03603 |  |  |
| Diospyros maclurei | 109.2706 | 19.03559 |  |  |
| Diospyros maclurei | 109.2705 | 19.04146 |  |  |
| Diospyros maclurei | 109.1215 | 19.0899 |  |  |
| Diospyros maclurei | 109.122 | 19.09043 |  |  |
| Diospyros maclurei | 109.1196 | 19.08955 |  |  |
| Diospyros maclurei | 109.119 | 19.08953 |  |  |
| Diospyros maclurei | 109.1205 | 19.08578 |  |  |
| Diospyros maclurei | 109.1209 | 19.08837 |  |  |
| Diospyros morrisiana | 109.0333 | 18.86036 | -22982.8 | 271527.7 |
| Diospyros morrisiana | 109.68 | 18.68397 | 44567.71 | 250043.4 |
| Diospyros morrisiana | 109.7715 | 18.5608 |  |  |
| Diospyros morrisiana | 109.7812 | 18.57038 |  |  |
| Diospyros morrisiana | 109.7723 | 18.56751 |  |  |
| Diospyros morrisiana | 109.7673 | 18.56215 |  |  |
| Diospyros morrisiana | 109.7753 | 18.56478 |  |  |
| Diospyros morrisiana | 109.7646 | 18.55812 |  |  |
| Diospyros morrisiana | 109.7685 | 18.57113 |  |  |
| Diospyros morrisiana | 109.7766 | 18.56344 |  |  |
| Diospyros morrisiana | 109.8303 | 18.58326 |  |  |
| Diospyros morrisiana | 109.8146 | 18.57647 |  |  |
| Diospyros morrisiana | 109.8244 | 18.5764 |  |  |
| Diospyros morrisiana | 109.8153 | 18.57768 |  |  |
| Diospyros morrisiana | 109.825 | 18.58218 |  |  |
| Diospyros morrisiana | 109.8252 | 18.58528 |  |  |
| Diospyros morrisiana | 109.8691 | 18.5994 |  |  |
| Diospyros morrisiana | 109.8604 | 18.60475 |  |  |
| Diospyros morrisiana | 109.8635 | 18.60588 |  |  |
| Diospyros morrisiana | 109.8565 | 18.60271 |  |  |
| Diospyros morrisiana | 109.8505 | 18.60461 |  |  |
| Diospyros morrisiana | 109.8609 | 18.59518 |  |  |
| Diospyros strigosa | 108.9122 | 18.99917 | -35232.9 | 287275.8 |
| Diospyros strigosa | 109.3011 | 18.85155 | 5180.119 | 269701.6 |
| Diospyros strigosa | 109.5106 | 18.24137 | 25337.66 | 201585.1 |
| Diospyros strigosa | 109.6828 | 18.3841 | 43968.29 | 216870.9 |
| Diospyros strigosa | 109.869 | 18.69141 | 64504.44 | 250339.5 |
| Diospyros strigosa | 109.467 | 19.22019 |  |  |
| Diospyros strigosa | 109.3927 | 19.23474 |  |  |
| Diospyros strigosa | 109.3554 | 19.21082 |  |  |
| Diospyros strigosa | 110.0666 | 18.53438 |  |  |
| Diospyros strigosa | 110.0719 | 18.52683 |  |  |
| Diospyros strigosa | 110.0723 | 18.52776 |  |  |
| Diospyros strigosa | 110.0655 | 18.54469 |  |  |
| Diospyros strigosa | 109.9471 | 19.77873 |  |  |
| Diospyros strigosa | 109.9884 | 19.71169 |  |  |
| Diospyros strigosa | 110.0391 | 19.73167 |  |  |
| Diospyros strigosa | 109.9902 | 19.76826 |  |  |
| Diospyros strigosa | 109.991 | 19.76763 |  |  |
| Diospyros strigosa | 109.7788 | 18.57255 |  |  |
| Diospyros strigosa | 109.8259 | 18.58253 |  |  |
| Diospyros strigosa | 109.8554 | 18.60399 |  |  |
| Diospyros strigosa | 109.6525 | 18.25606 |  |  |
| Diospyros strigosa | 109.5911 | 18.26256 |  |  |
| Diospyros strigosa | 109.5958 | 18.22585 |  |  |
| Diospyros strigosa | 108.7859 | 18.95889 |  |  |
| Diospyros strigosa | 109.0099 | 19.28734 |  |  |
| Diospyros vaccinioides | 109.9578 | 18.76128 | 74063.58 | 257827 |
| Diospyros vaccinioides | 109.7758 | 18.56917 |  |  |
| Diospyros vaccinioides | 109.8256 | 18.58572 |  |  |
| Diospyros vaccinioides | 109.8663 | 18.59783 |  |  |
| Diospyros vaccinioides | 109.8646 | 18.59659 |  |  |
| Diospyros vaccinioides | 109.8637 | 18.60587 |  |  |
| Diospyros vaccinioides | 109.8628 | 18.60126 |  |  |
| Diospyros vaccinioides | 109.8494 | 18.59832 |  |  |
| Diospyros vaccinioides | 109.8512 | 18.59841 |  |  |
| Diospyros vaccinioides | 109.7611 | 19.00194 |  |  |
| Diospyros vaccinioides | 109.7335 | 19.03526 |  |  |
| Diospyros vaccinioides | 109.7314 | 19.09625 |  |  |
| Diospyros vaccinioides | 109.8786 | 19.03217 |  |  |
| Diospyros vaccinioides | 109.7531 | 18.94208 |  |  |
| Diospyros vaccinioides | 109.7291 | 18.97657 |  |  |
| Diospyros vaccinioides | 109.773 | 19.11172 |  |  |
| Diospyros vaccinioides | 109.7422 | 19.09556 |  |  |
| Diospyros vaccinioides | 109.8157 | 19.09611 |  |  |
| Diospyros vaccinioides | 109.7762 | 19.05847 |  |  |
| Diospyros vaccinioides | 109.81 | 19.05686 |  |  |
| Diospyros vaccinioides | 109.7571 | 19.0633 |  |  |
| Diplodiscus trichospermus | 109.0212 | 19.35345 | -22551.4 | 326078.8 |
| Diplodiscus trichospermus | 109.0333 | 18.86036 | -22982.8 | 271527.7 |
| Diplodiscus trichospermus | 109.0609 | 18.7429 | -20480.1 | 258450.9 |
| Diplodiscus trichospermus | 108.7424 | 19.05101 |  |  |
| Diplodiscus trichospermus | 108.7452 | 19.06454 |  |  |
| Diplodiscus trichospermus | 108.7903 | 18.98575 |  |  |
| Diplodiscus trichospermus | 108.7889 | 18.95146 |  |  |
| Diplodiscus trichospermus | 108.7981 | 18.9594 |  |  |
| Diplodiscus trichospermus | 108.8079 | 18.9685 |  |  |
| Diplodiscus trichospermus | 108.7824 | 18.97056 |  |  |
| Diplodiscus trichospermus | 108.8083 | 18.96163 |  |  |
| Diplodiscus trichospermus | 109.6548 | 18.25045 |  |  |
| Diplodiscus trichospermus | 109.591 | 18.26145 |  |  |
| Diplodiscus trichospermus | 109.6558 | 18.24897 |  |  |
| Diplodiscus trichospermus | 109.5904 | 18.26107 |  |  |
| Diplodiscus trichospermus | 109.6512 | 18.25678 |  |  |
| Diplodiscus trichospermus | 109.5991 | 18.26115 |  |  |
| Diplodiscus trichospermus | 109.6588 | 18.24987 |  |  |
| Diplodiscus trichospermus | 109.5927 | 18.26143 |  |  |
| Diplodiscus trichospermus | 109.6576 | 18.25503 |  |  |
| Diplodiscus trichospermus | 109.5974 | 18.25592 |  |  |
| Diplodiscus trichospermus | 109.6515 | 18.25584 |  |  |
| Diplodiscus trichospermus | 109.5918 | 18.25734 |  |  |
| Diplospora dubia | 108.9122 | 18.99917 | -35232.9 | 287275.8 |
| Diplospora dubia | 109.0333 | 18.86036 | -22982.8 | 271527.7 |
| Diplospora dubia | 109.0609 | 18.7429 | -20480.1 | 258450.9 |
| Diplospora dubia | 109.1191 | 19.05985 | -13271 | 293304.6 |
| Diplospora dubia | 109.2343 | 18.80064 | -2022.54 | 264280.3 |
| Diplospora dubia | 109.5377 | 19.09747 | 30846.03 | 296177.5 |
| Diplospora dubia | 109.5409 | 18.34239 | 28849.41 | 212669.7 |
| Diplospora dubia | 109.6975 | 18.72833 | 46544.54 | 254900 |
| Diplospora dubia | 109.869 | 18.69141 | 64504.44 | 250339.5 |
| Diplospora dubia | 110.1756 | 18.76435 | 97019.43 | 257602 |
| Diplospora dubia | 109.8242 | 18.57502 |  |  |
| Diplospora dubia | 109.8259 | 18.58357 |  |  |
| Diplospora dubia | 109.8343 | 18.58064 |  |  |
| Diplospora dubia | 109.8167 | 18.57926 |  |  |
| Diplospora dubia | 109.6589 | 18.25328 |  |  |
| Diplospora dubia | 109.6564 | 18.25592 |  |  |
| Diplospora dubia | 109.6539 | 18.25234 |  |  |
| Diplospora dubia | 109.6544 | 18.24999 |  |  |
| Diplospora dubia | 110.0999 | 18.51705 |  |  |
| Diplospora dubia | 110.0648 | 18.55974 |  |  |
| Diplospora dubia | 110.1042 | 18.52029 |  |  |
| Diplospora dubia | 109.2957 | 18.79726 |  |  |
| Diplospora dubia | 109.2876 | 18.79841 |  |  |
| Diplospora dubia | 108.9878 | 19.27958 |  |  |
| Diplospora dubia | 109.0265 | 19.31055 |  |  |
| Diplospora dubia | 108.9867 | 19.29335 |  |  |
| Diplospora dubia | 109.7431 | 19.01361 |  |  |
| Diplospora dubia | 109.8172 | 19.04893 |  |  |
| Diplospora dubia | 109.4714 | 19.21705 |  |  |
| Diplospora dubia | 109.6693 | 19.96481 |  |  |
| Disepalum plagioneurum | 108.8278 | 19.25573 | -43193.9 | 315921.6 |
| Disepalum plagioneurum | 109.0333 | 18.86036 | -22982.8 | 271527.7 |
| Disepalum plagioneurum | 109.2333 | 18.38237 | -3508.87 | 218029 |
| Disepalum plagioneurum | 109.3163 | 18.42284 | 5389.832 | 222245.7 |
| Disepalum plagioneurum | 109.429 | 18.50349 | 17547.73 | 230821.1 |
| Disepalum plagioneurum | 108.7372 | 19.04975 |  |  |
| Disepalum plagioneurum | 108.7445 | 19.06467 |  |  |
| Disepalum plagioneurum | 108.7829 | 18.95864 |  |  |
| Disepalum plagioneurum | 109.0555 | 18.7569 |  |  |
| Disepalum plagioneurum | 109.2979 | 18.78544 |  |  |
| Disepalum plagioneurum | 109.1012 | 18.58227 |  |  |
| Disepalum plagioneurum | 109.146 | 18.55556 |  |  |
| Disepalum plagioneurum | 109.0782 | 18.61485 |  |  |
| Disepalum plagioneurum | 109.7683 | 18.57276 |  |  |
| Disepalum plagioneurum | 109.8276 | 18.58632 |  |  |
| Disepalum plagioneurum | 109.8493 | 18.59625 |  |  |
| Disepalum plagioneurum | 109.4706 | 19.21954 |  |  |
| Disepalum plagioneurum | 109.3946 | 19.23276 |  |  |
| Disepalum plagioneurum | 109.3494 | 19.21911 |  |  |
| Disepalum plagioneurum | 109.6591 | 18.25097 |  |  |
| Disepalum plagioneurum | 109.5921 | 18.25626 |  |  |
| Disepalum plagioneurum | 109.5975 | 18.2261 |  |  |
| Disepalum plagioneurum | 109.5931 | 18.22515 |  |  |
| Disepalum plagioneurum | 109.5943 | 18.22691 |  |  |
| Disepalum plagioneurum | 109.5976 | 18.22186 |  |  |
| Distylium racemosum | 109.1191 | 19.05985 | -13271 | 293304.6 |
| Distylium racemosum | 109.1544 | 19.01211 | -9728.08 | 287914.8 |
| Distylium racemosum | 109.1544 | 19.01212 | -9727.97 | 287914.9 |
| Distylium racemosum | 109.1544 | 19.01212 | -9727.86 | 287915 |
| Distylium racemosum | 109.1544 | 19.01212 | -9727.75 | 287915.1 |
| Distylium racemosum | 109.1231 | 19.08566 |  |  |
| Distylium racemosum | 109.1234 | 19.09088 |  |  |
| Distylium racemosum | 109.1235 | 19.08851 |  |  |
| Distylium racemosum | 109.1185 | 19.08899 |  |  |
| Distylium racemosum | 109.1201 | 19.08601 |  |  |
| Distylium racemosum | 109.1236 | 19.09069 |  |  |
| Distylium racemosum | 109.1217 | 19.08934 |  |  |
| Distylium racemosum | 109.1233 | 19.08724 |  |  |
| Distylium racemosum | 109.1191 | 19.08819 |  |  |
| Distylium racemosum | 109.2595 | 19.03366 |  |  |
| Distylium racemosum | 109.2661 | 19.04543 |  |  |
| Distylium racemosum | 109.2588 | 19.03394 |  |  |
| Distylium racemosum | 109.2742 | 19.04199 |  |  |
| Distylium racemosum | 109.2557 | 19.03807 |  |  |
| Distylium racemosum | 109.2607 | 19.0456 |  |  |
| Distylium racemosum | 109.2721 | 19.0409 |  |  |
| Distylium racemosum | 109.2649 | 19.03301 |  |  |
| Distylium racemosum | 109.2618 | 19.04454 |  |  |
| Distylium racemosum | 109.2711 | 19.03702 |  |  |
| Distylium racemosum | 109.2598 | 19.03839 |  |  |
| Dracaena cambodiana | 108.8974 | 18.5739 | -38293.2 | 240304.7 |
| Dracaena cambodiana | 109.0365 | 18.87698 | -22580.2 | 273355 |
| Dracaena cambodiana | 109.3163 | 18.42284 | 5389.832 | 222245.7 |
| Dracaena cambodiana | 109.3163 | 18.42284 | 5389.94 | 222245.8 |
| Dracaena cambodiana | 109.3163 | 18.42284 | 5390.049 | 222245.9 |
| Dracaena cambodiana | 110.0789 | 18.51346 |  |  |
| Dracaena cambodiana | 110.0683 | 18.52007 |  |  |
| Dracaena cambodiana | 110.0872 | 18.52505 |  |  |
| Dracaena cambodiana | 110.0683 | 18.51692 |  |  |
| Dracaena cambodiana | 110.1001 | 18.53757 |  |  |
| Dracaena cambodiana | 109.006 | 19.29175 |  |  |
| Dracaena cambodiana | 109.1045 | 19.30776 |  |  |
| Dracaena cambodiana | 109.073 | 19.30388 |  |  |
| Dracaena cambodiana | 109.1523 | 19.3041 |  |  |
| Dracaena cambodiana | 109.1608 | 19.36335 |  |  |
| Dracaena cambodiana | 109.6584 | 18.25716 |  |  |
[truncated: 657,173 more chars]
