# Supplementary material for: Spatial phylogenetics of the native woody plant species in Hainan, China
Source: Ecol Evol. 2021 Feb 1;11(5):2100–9. doi: 10.1002/ece3.7180 (PMC7920777; doi:10.1002/ece3.7180)
Supplement: Supplementary file 5 — Appendix S3 [file ECE3-11-2100-s005.docx]

| Region | Observed Richness/PD | PD_Significant | RPD_Significant |
| --- | --- | --- | --- |
| NE | 1.92 | Not Sig | Not Sig |
| NE | 2.63 | Not Sig | Not Sig |
| NE | 2.96 | Not Sig | Not Sig |
| NE | 1.96 | Not Sig | Not Sig |
| NE | 1.28 | Not Sig | Not Sig |
| NE | 5.42 | Not Sig | Not Sig |
| NE | 3.85 | Not Sig | Not Sig |
| NE | 0.88 | Not Sig | Not Sig |
| NE | 3.64 | Not Sig | Not Sig |
| NE | 2.14 | Not Sig | Not Sig |
| NE | 1.31 | Not Sig | Not Sig |
| NE | 9.48 | Not Sig | Not Sig |
| NE | 1.26 | Not Sig | Not Sig |
| NE | 0.87 | Not Sig | Not Sig |
| NE | 6.48 | Not Sig | Not Sig |
| NE | 2.22 | Not Sig | Not Sig |
| NE | 2.02 | Not Sig | Sig Low |
| NE | 3.59 | Not Sig | Not Sig |
| NE | 1.08 | Not Sig | Not Sig |
| NE | 16.28 | Not Sig | Not Sig |
| NW | 1.53 | Not Sig | Not Sig |
| NW | 2.95 | Not Sig | Not Sig |
| NW | 0.97 | Not Sig | Not Sig |
| NW | 2.33 | Not Sig | Not Sig |
| NW | 0.88 | Not Sig | Not Sig |
| NW | 2.07 | Not Sig | Not Sig |
| NW | 0.84 | Not Sig | Not Sig |
| NW | 0.86 | Sig Low | Sig Low |
| NW | 4.72 | Sig Low | Sig Low |
| NW | 1.01 | Very Sig Low | Very Sig Low |
| SE | 5.47 | Not Sig | Not Sig |
| SE | 3.56 | Not Sig | Not Sig |
| SE | 38.15 | Not Sig | Not Sig |
| SE | 0.99 | Not Sig | Highly Sig |
| SE | 1.37 | Not Sig | Not Sig |
| SE | 1.33 | Not Sig | Not Sig |
| SE | 2.19 | Not Sig | Not Sig |
| SE | 7.66 | Not Sig | Not Sig |
| SE | 10.84 | Sig Low | Sig Low |
| SE | 14.17 | Sig Low | Sig Low |
| SE | 2.25 | Very Sig Low | Very Sig Low |
| SE | 5.33 | Very Sig Low | Very Sig Low |
| SE | 1.74 | Not Sig | Not Sig |
| SE | 3.32 | Not Sig | Not Sig |
| SE | 17.69 | Not Sig | Not Sig |
| SW | 4.67 | Not Sig | Not Sig |
| SW | 5.1 | Not Sig | Not Sig |
| SW | 34.99 | Not Sig | Not Sig |
| SW | 1.07 | Not Sig | Highly Sig |
| SW | 15.44 | Not Sig | Not Sig |
| SW | 0.87 | Not Sig | Not Sig |
| SW | 2.57 | Not Sig | Not Sig |
| SW | 19.44 | Not Sig | Not Sig |
| SW | 21.64 | Not Sig | Very Highly Sig |
| SW | 2.79 | Not Sig | Highly Sig |
| SW | 1.16 | Not Sig | Highly Sig |
| SW | 10.7 | Sig Low | Sig Low |
| SW | 1.38 | Sig Low | Sig Low |
| SW | 3.03 | Sig Low | Sig Low |
| SW | 3.76 | Sig Low | Sig Low |
| SW | 22.57 | Sig Low | Sig Low |
| SW | 11.77 | Sig Low | Sig Low |
| SW | 15.63 | Sig Low | Sig Low |
| SW | 7.91 | Very Sig Low | Very Sig Low |
| SW | 42.83 | Very Sig Low | Very Sig Low |
| SW | 11.11 | Very Sig Low | Very Sig Low |
| SW | 24.24 | Very Sig Low | Very Sig Low |
| SW | 10.89 | Not Sig | Not Sig |
| SW | 1.91 | Not Sig | Not Sig |
