# Supplementary material for: Spatial phylogenetics of the native woody plant species in Hainan, China
Source: Ecol Evol. 2021 Feb 1;11(5):2100–9. doi: 10.1002/ece3.7180 (PMC7920777; doi:10.1002/ece3.7180)

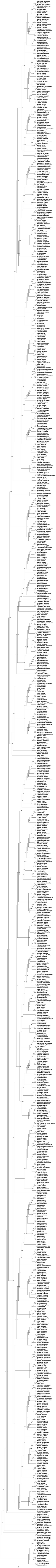

Supplement: Supplementary file 1 — Fig S1 [file ECE3-11-2100-s001.pdf]
